# Fertility is a key predictor of the double burden of malnutrition among women of child-bearing age in sub-Saharan Africa

**DOI:** 10.7189/jogh.10.020423

**Published:** 2020-12

**Authors:** Jason Mulimba Were, Saverio Stranges, Irena F Creed

**Affiliations:** 1 Department of Epidemiology and Biostatistics, Western University, London, Ontario, Canada; 2 Department of Family Medicine, Western University, London, Ontario, Canada; 3 Department of Population Health, Luxembourg Institute of Health, Strassen, Luxembourg; 4 The Africa Institute, Western University, London, Ontario, Canada; 5 Department of Biology, Western University, London, Ontario, Canada; 6 Department of Geography, Western University, London, Ontario, Canada; 7 School of Environment and Sustainability, University of Saskatchewan, Saskatoon, Saskatchewan, Canada

## Abstract

**Background:**

The ongoing nutrition transition in sub-Saharan Africa (SSA) is exhibiting spatial heterogeneity and temporal variability leading to different forms of malnutrition burden across SSA, with some regions exhibiting the double burden of malnutrition. This study aimed to develop a predictive understanding of the malnutrition burden among women of child-bearing age.

**Methods:**

Data from 34 SSA countries were acquired from the Demographic and Health Survey, World Bank, and Swiss Federal Institute of Technology. The SSA countries were classified into malnutrition classes based on their national prevalence of underweight, overweight, and obesity using a 10% threshold. Next, random forest analysis was used to examine the association between country-level demographic variables and the national prevalence of underweight, overweight and obesity. Finally, random forest analysis and multinomial logistic regression models were utilized to investigate the association between individual-level social and demographic variables and Body Mass Index (BMI) categories of underweight, normal weight, and combined overweight and obesity.

**Results:**

Four malnutrition classes were identified: Class A had 5 countries with ≥10% of the women underweight; Class B had 11 countries with ≥10% each of underweight and overweight; Class C1 had 7 countries with ≥10% overweight; and Class C2 had 11 countries with ≥10% obesity. At the country-level, fertility rate predicted underweight, overweight and obesity prevalence, but economic indicators were also important, including the gross domestic product per capita – a measure of economic opportunity that predicted both overweight and obesity prevalence, and the GINI coefficient – a measure of economic inequality that predicted both underweight and overweight prevalence. At the individual-level, parity was a risk factor for underweight in underweight burdened countries and a risk factor for overweight/obesity in overweight/obesity burdened countries, whereas age and wealth were protective factors for underweight but risk factors for overweight/obesity.

**Conclusions:**

Beyond the effect of economic indicators, this study revealed the important role of fertility rate and parity, which may represent risk factors for both underweight and combined overweight and obesity among women of child-bearing age. Health professionals should consider combining reproductive health services with nutritional programs when addressing the challenge of malnutrition in SSA.

The global burden of overweight and obesity have reached epidemic proportions. The World Health Organization (WHO) estimates that approximately 1.9 billion adults (18 years and above) were overweight, with 650 million of them being obese in 2016 worldwide [1]. These changes in nutritional status may be explained by “nutrition and epidemiological transition theories”, theories that describes shifts in nutrition, lifestyle and disease patterns, with a progressive surge in non-communicable diseases (NCDs) [2,3]. A large proportion of this burden is concentrated in High-Income-Countries (HICs), but worrisome trends towards higher overweight and obesity have been reported in Low- and Middle-Income Countries (LMICs), due to the rapid socio-demographic and epidemiological transition occurring in these settings [4,5].

Nutrition transition theory, which is described by historical shifts in dietary and physical activity patterns through five stages (collecting food, famine, receding famine, degenerative disease and behavioral change) [2], exhibits a large degree of spatial heterogeneity and temporal variability with regards to the nature and the pace of the transition [6]. Spatially, most countries are encountering stage 4, degenerative disease, which is characterised by increased consumption of high calorie foods, typically high in fat, sugar and refined carbohydrates [2,4]. This dietary culture is often accompanied by low levels of physical activity and thus high incidence and prevalence of obesity, hypertension, diabetes and other nutrition related NCDs are reported [2]. Temporally, HICs, except those in East Asia, started the transition from stage 3, receding famine, to stage 4, degenerative disease in the early twentieth century and progressed slowly up to 1980s, where they appear to remain in most countries [6]. In contrast, LMICs, started the transition from stage 3 to stage 4 in the 1980s and have since rapidly accelerated to stage 4 [6,7]. The patterns in transitions have been uneven in LMICs, with stage 4 being more visible in urban than in rural areas [6]. Consequently, some LMICs are encountering a double burden of malnutrition (DBM), where sub-groups of the population are underweight and others are overweight [4].

Sub-Saharan Africa (SSA) is a region where many countries have been grappling with the DBM. In 1975, 18% of SSA adults were underweight while 15.5% were either overweight or obese [5]. Among these, men were more often underweight (males 19.3% vs females 17.4%), whereas women were more often overweight/obese (males 7.5% vs females 14.7%) [5]. Forty years later, 9.5% of SSA adults were underweight and 38.4% were overweight or obese [5]. Once again, men were more often underweight (males 11.5% vs females 8.9%), whereas women were more often overweight/obese (males 22.2% vs females 39.7%) [5]. In SSA, the DBM is exacerbated by existing inequities in food security [8-11].

In SSA, over the last 40 years, the transition towards over-nutrition occurred rapidly, with women being more affected by this transition than men [5,9,12]. However, no study has examined the DBM among women of child-bearing age both among and within SSA countries. Given the socio-demographic diversity that exists in the SSA, understanding the patterns and predictors of malnutrition is important in designing context specific health interventions. Here, we conduct a comprehensive study of the DBM among women of child-bearing age in SSA. The objectives are: (1) to classify countries by malnutrition prevalence of women of child-bearing age; and (2) to determine key country-level indicators associated with the underweight, overweight and obesity prevalence among women of child-bearing age; and (3) to determine key individual-level risk factors for underweight and combined overweight and obesity among women of child-bearing age. The novelty of this study is 2-fold. First, our use of conditional inference random forest analysis in this study aims to contribute to methodological approaches for examining malnutrition predictors. Second, our findings can be used to inform the “double-duty” policy objectives, that seek to leverage existing policies and interventions to simultaneously address DBM [11]. This study is timely as the WHO is currently advancing the discourse on reframing nutritional policies to tackle the new nutritional reality within this region [11].

## METHODS

Data were obtained from the Demographic and Health Survey (DHS) program, a publicly available public health-oriented data (https://www.dhsprogram.com/). We used the most recent surveys from SSA countries (from 2007 to 2016). DHS uses a two-stage stratified sampling design, where households are sampled from administratively defined Primary Sampling Units (PSU) in each country [13]. Individuals aged 15 years and above from the selected households are eligible to participate [13]. An *a priori* criterion was applied to limit the study to participants with valid anthropometric measurements [14]. Participants lacking height and weight data, pregnant women, and women who had given birth two months prior to the survey were excluded, as were participants missing data on covariates of interest. Initial and the final samples sizes for each country are presented in **Table 1**.

**Table 1:** DHS survey information and country-level indicators from 34 countries in sub-Saharan Africa

Country	Survey year	Initial sample size	Final sample size	GDP per capita (Current US$)	Urbanization (%)	GINI coefficient	Globalization index	Fertility rate	Life expectancy (Years)
Benin	2011\12	16 599	14 060	837	42.67	0.48	42.6	5.25	59.8
Burkina Faso	2010	17 087	7212	575	25.66	0.35	43.62	5.87	57.01
Burundi	2010	9389	3929	231	10.64	0.33	33.07	6.26	54.84
Cameroon	2011	15 426	6813	1296	50.1	0.47	42.9	5.05	55.81
Chad	2014\15	17719	8873	777	22.47	0.43	39.57	6.05	52.55
Comoros	2012	5329	4479	789	28.02	0.45	30.84	4.63	62.58
Congo	2011\12	10 819	4822	2952	64.1	0.49	45.19	4.88	62.15
D R Congo	2013\14	18 827	7650	461	41.98	0.42	39.98	6.29	58.75
Ethiopia	2016	15 683	13 222	707	19.92	0.33	-	4.32	65
Gabon	2012	8422	4213	9774	86.36	0.42	49.37	4.01	64.1
Gambia	2013	10 233	3886	486	58.37	0.47	49.72	5.6	60.46
Ghana	2014	9396	4238	1432	53.39	0.42	54.17	4.1	62.11
Guinea	2012	9142	4038	502	35.75	0.34	42.54	5.18	57.76
Ivory Coast	2011\12	10 060	4057	1263	52.04	0.42	48.42	5.16	51.49
Kenya	2014	31 079	12 962	1335	25.2	0.48	46.64	3.99	66.19
Lesotho	2014	6621	3157	1175	26.79	0.54	45.94	3.19	53.09
Liberia	2013	9239	3998	454	48.92	0.33	39.28	4.79	61.04
Madagascar	2008\09	17 375	7379	416	31.29	0.43	41.26	4.69	62.92
Malawi	2015\16	24 562	7179	301	16.45	0.46	-	4.65	62.54
Mali	2012\13	10 424	4397	778	38.36	0.33	45.11	6.32	56.53
Mozambique	2011	13 745	11 617	527	31.18	0.46	44.7	5.52	55.19
Namibia	2013	10 018	3921	5488	44.68	0.61	52.63	3.56	61.85
Niger	2012	11 160	4104	392	17.98	0.34	45.33	7.42	58.17
Nigeria	2013	38 948	32 078	2997	46.9	0.43	52.49	5.71	52.11
Rwanda	2014\15	13 497	5995	710	28.81	0.5	45.56	3.97	66.62
Sao Tome and Principe	2008\09	2615	2078	1100	61.18	0.31	31.36	4.84	65.64
Senegal	2010\11	15 688	5020	1080	42.49	0.4	52.38	5.04	64.71
Sierra Leone	2013	16 658	6971	711	39.27	0.34	44.79	4.79	50.39
Eswatini	2006\07	4987	4402	3047	21.78	0.51	42.83	3.76	47.68
Tanzania	2015\16	13 266	11 535	879	31.61	0.38	-	5.08	64.9
Togo	2013\14	9480	4238	620	39.47	0.43	53.7	4.59	59.58
Uganda	2011	8674	2308	584	14.8	0.41	44.6	6.06	57.72
Zambia	2013\14	16 411	14 129	1738	40.47	0.56	52.47	5.1	60.72
Zimbabwe	2015	9955	8721	1019	32.38	0.43	44.04	3.84	60.67

### Nutritional status

For DHS participants, weight and height data were objectively measured, using standardized solar-powered scales and height/length boards, respectively [15]. Body Mass Index (BMI) is the individual’s weight in kilograms by height in metres squared (kg/m^2^) [16]. Our BMI categories were in accordance with WHO classification as follows: underweight <18.5 kg/m^2^; normal weight 18.5 to 24.9 kg/m^2^; overweight 25 to 29.9 kg/m^2^ and obesity ≥30 kg/m^2^.

### Predictors of nutritional status

Country-level characteristics that could be associated with nutritional status were considered, based on available evidence, including: demographics [ie, fertility rate (average number of live births per woman for the duration of her reproductive years) and life expectancy at birth (average number of years a female is expected to live if mortality rates remain the same)] [14,17-19]; urbanization (represented by percentage of people living in urban areas) [17]; economic opportunity (represented by the gross domestic product (GDP) per capita, in current US$) [17,18]; economic inequality (represented by the GINI coefficient) [14,20,21]; and the KOF globalization index (reflecting the degree of a country’s interdependence in both social, political and economic dimensions) [22]. These data were acquired from the World Bank (https://data.worldbank.org/) and Swiss Federal Institute of Technology (https://kof.ethz.ch/en/data.html) for years corresponding to the DHS survey year, except for the GINI coefficient, for which values for the closest year to the DHS surveys were used.

Individual-level characteristics that could be associated with the nutritional status of women were also considered, including the following from DHS data: age (5-year age groups); marital status (single, married/partnered, formerly married/partnered); hormonal contraceptive use (yes or no); parity (0, 1, 2, 3, 4, 5 and 6+); ethnicity (tribe); religion (Catholics, other Christians, Islam, Traditionalists, others, and no religion); administrative region of residence (country-specific); residential setting (rural and urban); education (no education, primary, secondary, and higher); occupation (not working, non-manual/service workers, manual workers, agricultural workers and others); wealth quintile (poorest, poorer, middle, richer and richest); and media exposure (not exposed, exposed to one media source, exposed to two media sources and exposed to three media sources) [8,9,12,21,23-26].

### Statistical analysis

Descriptive statistics were calculated to determine the population-weighted prevalence for underweight, overweight and obesity. For country-level analyses, the three BMI categories were used, but for individual-level analyses, overweight and obesity were combined to form one overweight/obesity category (BMI≥25 kg/m^2^) for analytical purpose due to small proportions of the obesity category across most of the countries [21,23-25].

**Figure 1:**
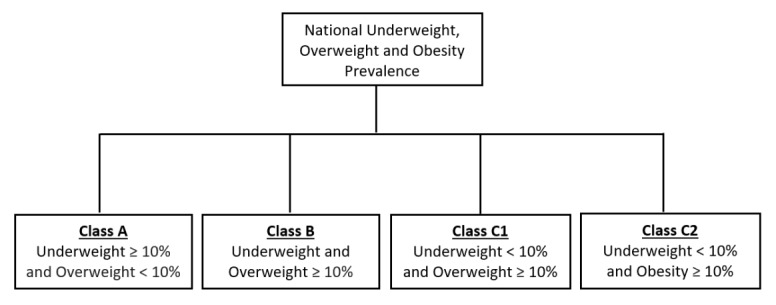
Flowchart summary for the creation of malnutrition classes.

For objective 1, a 10% prevalence was chosen as a threshold that would signal a public health problem. There are no known population-based thresholds for malnutrition that would signal a public health concern. However, WHO suggests that a prevalence of 10% and above for adult underweight (BMI<18.5 kg/m^2^) signifies a poor nutrition situation [16]. Furthermore, prior studies conducted in LMICs suggest that a prevalence of 10% or more for both underweight and overweight deserves public health attention [8,9,20,21,23-25,27]. Therefore, a 10% threshold was used to classify SSA countries into four malnutrition classes: Class A consisted of countries with an underweight prevalence ≥10% and an overweight prevalence <10%; Class B countries had an underweight and an overweight prevalence ≥10%; Class C countries had an underweight prevalence <10% and an overweight prevalence ≥10% (C1) or obesity prevalence ≥10% (C2) (**Figure 1**).

For objective 2, a random forest analysis using a conditional inference approach was performed to determine the relative importance of each country-level indicator in predicting the underweight, overweight and obesity prevalence [28,29]. The forest size was set to 1000 and the number of variables considered for splitting at each node was set to the square root of the total number of variables [29-31]. The threshold for assessing variable importance was set to the absolute value of the lowest ranked variable [28]. Since decision tree analysis involves a random process, multiple runs (7) were conducted using different seed numbers to ensure the robustness of the results [28,29]. A cross validation analysis, a synonymous testing procedure often used in prediction models to assess goodness-of-fit, was not conducted because the built-in bootstrap aggregating (bagging) process in the random forest framework allows for testing of the model fitness through the out-of-bag samples [28]. After repeated re-runs of the analyses, models with the smallest out-of-bag error estimates were selected as the final models for this study.

For objective 3, a random forest analysis was used to determine the relative importance of each individual-level variable in predicting women’s nutritional status for each country. The forest size for each country was set at 500 since all countries had a large sample size (>2000). Considering that random forest and variable importance analyses are computationally intensive algorithms, using the default setting for forest size (500) provided the best balance between precision and computation times [28]. To confirm the appropriateness of the forest size, five countries (Ethiopia, Burundi, Ghana, Nigeria and Sao Tome and Principe) with varying sample sizes, were selected at random and the random forest analyses were conducted with forest sizes of 500, 750 and 1000 for each country. In addition to random forest analyses, multinomial logistic regression models were fitted to examine the risk of being overweight/obese and underweight as compared to normal weight. To ensure our estimates were representative of the population, unequal sample weights and stratification were accounted for in all logistic regression models [17]. Goodness-of-fit was assessed using root mean square error (random forest regression analysis) [32], out-of-bag error (random forest classification analysis) [32,33], and F-test (logistic regression analyses) [34]. Statistical analyses were conducted using R (version 3.3.2) (R Foundation for Statistical Computing, Vienna, Austria) and STATA (version 14) (StataCorp, College Station, TX, USA) software.

## RESULTS

**Figure 2:**
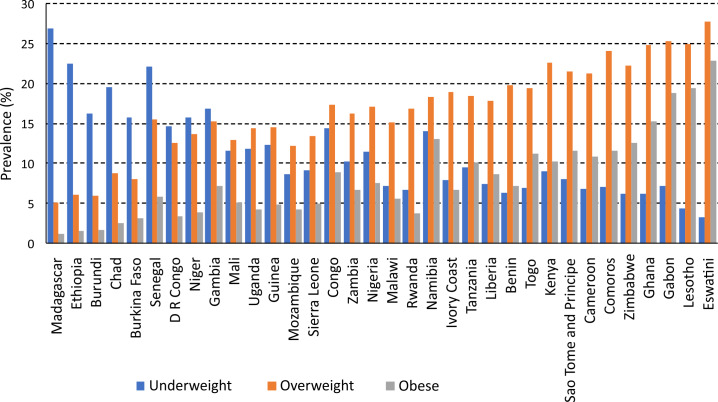
Prevalence of underweight (BMI<18.5 kg/m2), overweight (25 to 29.9 kg/m2) and obesity (BMI≥30.0 kg/m^2^) among women of reproductive age in SSA.

Descriptive statistics of malnutrition in the 34 SSA countries are presented in **Table 1** and **Figure 2**. Underweight (BMI<18.5 kg/m^2^) prevalence exceeded 10% in 16 countries, with the highest observed in Madagascar (26.9%). Overweight/obesity (BMI>25.0 kg/m^2^) prevalence exceeded 10% in 31 countries, with 12 of these countries with obesity (BMI>30.0 kg/m^2^) prevalence exceeding 10%. Country-specific descriptive statistics are presented in Tables S1-S34 in the **Online Supplementary Document**.

There were four classes of malnutrition in SSA based on the 10% threshold (**Table 2**). Five countries were characterized by ≥10% burden of underweight (Class A). Eleven countries were characterized by ≥10% prevalence of underweight and ≥10% overweight (Class B, representing a DBM). Eighteen countries were characterized by ≥10% overweight or obese [Class C, with seven countries with ≥10% overweight (C1) and eleven countries with ≥10% obese (C2)].

**Table 2:** Malnutrition classes in sub-Saharan Africa (SSA)

Class	Countries
**Class A**	Madagascar, Ethiopia, Burundi, Chad, Burkina Faso
**Class B**	Niger, Democratic Republic of Congo, Mali, Uganda, Guinea, Gambia, Senegal, Namibia, Zambia, Nigeria, Congo
**Class C1**	Mozambique, Sierra Leone, Rwanda, Malawi, Ivory Coast, Benin, Liberia
**Class C2**	Cameroon, Comoros, Togo, Sao Tome and Principe, Kenya, Tanzania, Gabon, Zimbabwe, Eswatini, Lesotho, Ghana

SSA – sub-Saharan Africa

At the country-level, fertility rate was the strongest predictor of malnutrition, followed by the economic indicators of opportunity (GDP) and equality (GINI coefficient). Specifically, for underweight prevalence, fertility rate followed by the GINI coefficient were the strongest predictors (Figure S1 in the **Online Supplementary Document**). For overweight prevalence, fertility rate followed by GDP and the GINI coefficient were the strongest predictors (Figure S2 in Online Supplementary Document). For obesity, fertility rate followed by GDP were the strongest predictors (Figure S3 in **Online Supplementary Document**). Fertility rate was highest in countries with significant prevalence of underweight (Class A, median fertility rate = 5.9) and countries with the double burden of underweight and overweight (Class B, median = 5.6). Fertility rate was lowest in countries with significant prevalence of overweight (Class C1, median = 4.8) and obesity (Class C2, median = 4.1). The GINI coefficient was lowest in countries with significant prevalence of underweight (Class A, median = 0.35), followed by countries with the double burden of underweight and overweight (Class B, median = 0.42), and then by countries with overweight and obesity – Class C countries (Class C1, median = 0.46 and Class C2, median = 0.43). The GDP showed a non-systematic trend, with GDP lowest in countries with significant prevalence of underweight (Class A, median = US$575), followed by countries with high prevalence of overweight (Class C1, median = US$710), followed by countries with high prevalence of the double burden of underweight and overweight (Class B, median = US$778), and finally countries with high prevalence of obesity (Class C2, median = US$1175) (**Table 3**).

**Table 3:** Distribution of the informative predictors of underweight, overweight and obesity prevalence

Class	Fertility rate	GINI coefficient	GDP per capita, current US$
**A** Mean (sd)	5.5 ± 0.90	0.37 ± 0.05	541 ± 221.4
Median	5.9	0.35	575
Quartile coefficient of dispersion	0.13	0.13	0.26
**B** Mean (sd)	5.6 ± 0.99	0.44 ± 0.09	1587 ± 1615.4
Median	5.6	0.42	778
Quartile coefficient of dispersion	0.12	0.18	0.72
**C1** Mean (sd)	4.9 ± 0.50	0.43 ± 0.05	686 ± 312.1
Median	4.8	0.46	710
Quartile coefficient of dispersion	0.06	0.17	0.30
**C2** Mean (sd)	4.3 ± 0.60	0.44 ± 0.06	2042 ± 2642.9
Median	4.1	0.43	1175
Quartile coefficient of dispersion	0.12	0.07	0.24

sd – standard deviation

At the individual-level, parity was a stronger predictor but had a more nuanced association with malnutrition in the within-country analysis (See **Table 4** for random forest results and Tables S35-S68 in Online Supplementary Document). Among Class A countries, multinomial logistic regression analyses adjusted for confounding variables showed that higher parity was associated with a higher risk of underweight for mothers who had at least one child compared to a childless woman in Burundi and Ethiopia. However, higher parity was associated with a lower risk of overweight/obesity relative to normal weight for mothers with six or more children in Burkina Faso. In Class B countries, higher parity was associated with mixed effects in terms of malnutrition. Higher parity had a protective effect against underweight in Niger, Senegal, Guinea, Namibia, and Nigeria, while also having a protective effect against overweight/obesity in Uganda, Niger, and Guinea among women with three or more children. However, higher parity was associated with a higher risk of overweight/obesity in Zambia and Namibia. In Class C countries (C1 and C2), higher parity was generally associated with a lower risk of underweight and a higher risk of overweight/obesity compared to a childless woman. Malawi and Mozambique were exceptions to this pattern with higher parity associated with a reduction in risk of being overweight/obese relative to normal weight.

**Table 4:** A summary of the most important within country predictors of nutritional status in sub-Saharan Africa (SSA)

Country	Variables by rank of importance		
**First**	**Second**	**Third**
**Class A:**			
Madagascar	Region	Wealth Quintile	Age Group
Ethiopia	Region	Age Group	Wealth Quintile
Burundi	Age group	Marital status	Residential setting
Burkina Faso	Age group	Ethnicity	Wealth quintile
Chad	Ethnicity	Region	Age Group
**Class B:**			
D R Congo	Wealth Quintile	Age Group	Ethnicity
Niger	Wealth Quintile	Region	Age Group
Mali	Age Group	Wealth Quintile	Residential Setting
Gambia	Age Group	Region	Wealth Quintile
Uganda	Wealth Quintile	Age Group	Marital Status
Guinea	Age Group	Region	Wealth Quintile
Senegal	Age Group	Residential Setting	Region
Namibia	Age Group	Wealth Quintile	Region
Congo	Age Group	Wealth Quintile	Region
Nigeria	Age Group	Wealth Quintile	Parity
Zambia	Wealth Quintile	Age Group	Parity
**Class C1:**			
Sierra Leone	Age Group	Wealth Quintile	Parity
Mozambique	Age Group	Wealth Quintile	Parity
Malawi	Age Group	Wealth Quintile	Parity
Ivory Coast	Age Group	Wealth Quintile	Residential Setting
Rwanda	Wealth Quintile	Parity	Occupation
Liberia	Age Group	Wealth Quintile	Occupation
Benin	Age Group	Region	Wealth Quintile
**Class C2:**			
Kenya	Wealth Quintile	Age Group	Ethnicity
Sao Tome and Principe	Age Group	Wealth Quintile	Marital Status
Togo	Age Group	Wealth Quintile	Region
Cameroon	Age Group	Ethnicity	Wealth Quintile
Comoros	Age Group	Parity	Wealth Quintile
Tanzania	Age Group	Wealth Quintile	Region
Zimbabwe	Age Group	Wealth Quintile	Parity
Gabon	Age Group	Wealth Quintile	Parity
Ghana	Age Group	Wealth Quintile	Occupation
Lesotho	Age Group	Wealth Quintile	Parity
Eswatini	Age Group	Parity	Wealth Quintile

SSA – sub-Saharan Africa

Multinomial logistic regression analyses also revealed a strong dose-response effect of wealth on malnutrition; regardless of the malnutrition classes, women classified in wealthier quintiles had a lower risk of being underweight and a higher risk of being overweight/obese, relative to normal weight. Age and region were also found as strong predictors of malnutrition within countries regardless of the class of malnutrition (Tables S35-S68 in **Online Supplementary Document**). Like wealth, age had a risk effect for overweight/obesity and a protective effect for underweight in a dose-response manner. Region had a relatively strong effect among Class A and B countries and a relatively weak effect among Class C1 and C2 countries (**Table 4**).

## DISCUSSION

Globally, countries are undergoing an epidemiological and nutrition transition from high prevalence of underweight towards overweight/obesity because of shifts in diet, lifestyles, and socio-demographic profiles [2,4,5]. Most HICs are in a nutrition state characterized by a huge burden of chronic-degenerative disease, with a surge in lifestyle related NCDs such as stroke, hypertension, and obesity, attributed to high consumption of foods rich in cholesterol, fats, refined starch and sugars, and sedentary lifestyles [6]. LMICs are following a similar nutrition transition, but one that is happening so quickly that some countries are facing the DBM – with high prevalence of both underweight and overweight/obesity [4,6,18]. We conducted a comprehensive study of the malnutrition among women of child-bearing age to gain insight to the factors leading to a DBM in SSA.

Our use of a subjective 10% threshold in prevalence of BMI to define underweight, overweight and obesity revealed that SSA countries fell within one of four malnutrition classes. A previous study using a scoring system [18] was not able to distinguish these four malnutrition classes. Specifically, Abrahams et al. (2011) found that out of 40 SSA countries, 65% of the countries were classified as being in the receding famine stage [stage 3] and 35% of the countries were classified as being in the degenerative diseases stage [stage 4] [18] of the nutrition transition. In contrast, our study found that out of 34 SSA countries (we did not include 6 of the countries in Abrahams et al. study as we relied on DHS data only), 15% of the countries continue to face significant undernutrition, 32% of the countries are grappling with the DBM where women are at a greater risk of developing undernutrition related conditions such as anemia, osteoporosis and birth complications as well as at a greater risk of developing cardiovascular diseases and other related NCDs [12,35], and 53% of the countries have transitioned to high prevalence of overweight/obesity, suggesting the emergence of NCDs, including hypertension, coronary heart disease, stroke and cancer [35]. The results of our approach reveal a complex scenario with high heterogeneity across SSA, with countries experiencing high prevalence of undernutrition, DBM, or over-nutrition, suggesting the need for a “customized” approach to nutritional policies and programs.

Our use of random forest analyses revealed a unique relationship between fertility indicators and malnutrition, both across and within countries.

*Across* countries, a strong association was observed between fertility rate and all malnutrition burdens (ie, prevalence of underweight, overweight and obesity). Countries with high fertility rates had the highest underweight burden, and vice versa, countries with low fertility rates had the lowest underweight burden. Countries with high fertility rates include Madagascar, Chad and Niger, which are among the most food insecure countries in the world [36]. In the context of large family sizes and food insufficiencies, underweight is a likely reflection of inadequate food intake for women, with feeding priorities directed towards infants and children [37,38]. On the other hand, countries with low fertility rates had the highest overweight and obesity burden, which is consistent with the nutrition and epidemiological transition theories [3,6,10]. Relative to other countries, lower fertility rates in countries such as Ghana [39] and Sao Tome and Principe [40] could be associated with higher female participation in the formal labour force, which is historically known to be incompatible with childcare and thus discouraging high parity [19]. Therefore, the link between low fertility rates and overweight prevalence could be explained by increased engagement of women in sedentary labour markets.

*Within* countries, parity was found to be a risk factor for underweight in countries with high prevalence of underweight, and a risk factor for overweight/obesity in countries with a high burden of overweight and obesity. Parity as a risk factor of underweight in women of child-bearing age could reflect multiple reproductive cycles within short intervals that does not allow for sufficient replenishment of the body’s nutrient stock [38]. Women are physiologically vulnerable to malnutrition, with reproductive functions such as pregnancies and breastfeeding often increasing nutritional requirements [38,41]. Women in poverty-stricken settings where food insecurity is endemic are often engaged in energy demanding agricultural occupations that often leaves them nutritionally depleted [24]. In contrast, parity as a risk factor of overweight/obesity in obesogenic settings could be as a result of pre-partum weight gain and postpartum weight retention for women with an adequate albeit nutrient-deficient food supply [9]. Given that fertility rate was found to be a strong predictor of the prevalence of underweight, overweight and obesity at the country-level, this study highlights the importance of considering women’s reproductive cycles (the frequency of pregnancies and the recovery in between pregnancies) in addressing the malnutrition challenge. Policy makers and public health practitioners should consider linking pre- *and* post-natal health services that are highly prioritised in many SSA countries to nutritional programs (eg, nutrition education and physical activity campaigns).

Fertility and parity were key predictors of malnutrition class, but previously reported relationships between economic development indicators and malnutrition [14,17,20,21,42] were also observed. Our study found the GINI coefficient to be a significant predictive factor for both underweight and overweight (but not obesity) prevalence. As a marker of income inequality, studies in India [20] and Indonesia [21] have found the GINI coefficient to be associated with both underweight and overweight/obesity prevalence. A higher GINI coefficient is likely linked to an uneven distribution of food and other resources that are crucial to an individual’s nutritional well-being [20]. Therefore, in countries with substantial income inequality, the socio-economic elites are more likely to indulge in overconsumption whereas the socially and economically disadvantaged are more likely to be afflicted by food insecurity [20,21]. Furthermore, income inequality could be a marker for social dissolution and inefficiencies in public policies and governance [20,43]. Hence, in countries with high income inequality, corruption and manipulation of public policies by vested interests are likely to exacerbate the DBM by interfering with the equal provision of amenities essential for improving individual’s nutritional status [20,21,43]. Our study finding of GDP as a predictor of both overweight and obesity prevalence is not surprising, as this is well established in the literature [17,20,22,42]. Increases in national income is often accompanied by a shift from traditional subsistence farming to modern industrial economies [42,44]. Therefore, with the migration to urban areas in search of economic opportunities, there is reduced access to locally produced healthy foods and a reduction in other nutritionally healthy activities such as exercise and breastfeeding [44].

Regardless of the malnutrition classes, wealth and age were found to be the strongest predictors of a women’s nutritional status at the individual-level, consistent with other studies conducted in developing countries [8,21,23,24,26]. At the individual-level, wealth reflects an increase in disposable income; wealthier individuals are more likely to adopt a ‘Western’ dietary pattern [21,24,25]. Age is associated with a decrease in physical activity, lower metabolism and loss of muscle tissues, which in the long run contributes to an increase in body weight [45]. In SSA countries, age is an important determinant of a woman’s social position in the community. Older women are revered within homesteads and are often tasked with sedentary supervisory roles, while the younger ones perform the most rigorous domestic chores [46]. This may contribute to lower physical activity levels and therefore higher likelihoods of being overweight/obese as one ages.

Based on the nutrition and epidemiologic transition theory, we expect that SSA populations will transition to a nutrition state characterized by overweight/obesity and chronic-degenerative diseases [2,3]. However, it is possible that this nutrition state could be “leap frogged”, if economic opportunities were provided to enable the population to adopt healthy food choices and active lifestyles and avoid some of the poor side-effects of westernization. Policy makers should consider developing food policies that promote equitable access to healthy foods.

Our study had several limitations worth noting. First, BMI is a relative measure of body weight and therefore may not adequately measure one’s nutritional status as it does not distinguish between body fat and lean mass [14,47]. Furthermore, body fat distribution differs by ethnicity and therefore, the WHO recommended BMI thresholds may not be an accurate measure of nutritional status for women of African decent [47]. However, BMI is shown to highly correlate with gold standard measures for nutritional status, therefore making it the only proxy measure for many large-scale population studies [14]. Second, our modelling and analyses were limited to correlates of malnutrition that were available in the DHS data set; we could not examine the effect of behavioral and diet related predictors [12,14]. Third, random forests are limited in terms of predictions beyond the input data [32,48]. However, our study used random forests to determine the relative importance of predictor variables, offering important insights into the data that are not available from other conventional analytical techniques. Furthermore, our random forests analyses were complemented with logistic regression models to determine the relative risk ratio estimates of the predictor’s variables. Finally, our study was based on cross-sectional rather than longitudinal data that limited our interpretations to associations rather than causal links.

## CONCLUSION

SSA countries are facing a significant public health challenge. Specifically, out of the 34 countries studies, five countries continue to face significant undernutrition, 11 countries face the double burden of malnutrition (with greater than 10% prevalence of underweight and overweight/obesity), and 18 countries face significant over-nutrition [with greater than 10% prevalence of overweight (7 countries) or obesity (11 countries)]. Fertility rate, GINI coefficient and GDP are key predictors of underweight, overweight and/or obesity among women of child-bearing age at the country-level, whereas parity, age and wealth are key predictors at the individual-level. Beyond the focus on economic indicators, health policies need to consider the complex relationship between fertility and nutrition status of women of child-bearing age, and health professionals should consider combining reproductive health services with nutritional programs when addressing the complex challenge of malnutrition in SSA countries.

## Additional material

Online Supplementary Document
