# Supplementary material for: Fertility is a key predictor of the double burden of malnutrition among women of child-bearing age in sub-Saharan Africa
Source: J Glob Health. 2020 Oct 8;10(2):020423. doi: 10.7189/jogh.10.020423 (PMC7568927; doi:10.7189/jogh.10.020423)
Supplement: Online Supplementary Document [file jogh-10-020423-s001.pdf]

ONLINE SUPPLEMENTARY DOCUMENT

Appendix S1: Random forest Box plots for country level Analysis (Figures S1-S3).

1. The box plots present a summary of country-level predictors for underweight (BMI <18.5 kg/m^2), overweight (BMI 25.0 – 29.9 kg/m^2) and obesity (BMI > 29.9 kg/m^2) prevalence.
2. Variables to the right of the dashed line (absolute value of the lowest ranking variable) are the most influential predictors for underweight, overweight and obesity prevalence, ranked based on the relative importance score.
3. Presented alongside the figure caption is the root mean square error (RMSE), used as goodness of fit statistic for the model.
4. Variable Definitions:
    a. **GDP:** Gross domestic product divided by midyear population (Current USSD).
    b. **Urbanization:** Proportion of the total population living in urban areas (% of the total).
    c. **GINI Index (GINI Coefficient):** Measure of deviation of individual's income from a perfectly equal distribution (0=Perfect Equality while 1=Perfect Inequality).
    d. **Globalization (KOF Index of Globalization):** Measure of the degree of Globalization incorporating, the social, political, and economic dimensions.
    e. **Fertility:** Average number of live births per woman until the end of her child-bearing years.
    a. **Life Expectancy:** Average number of years a newborn female is expected to live if mortality patterns remain the same.

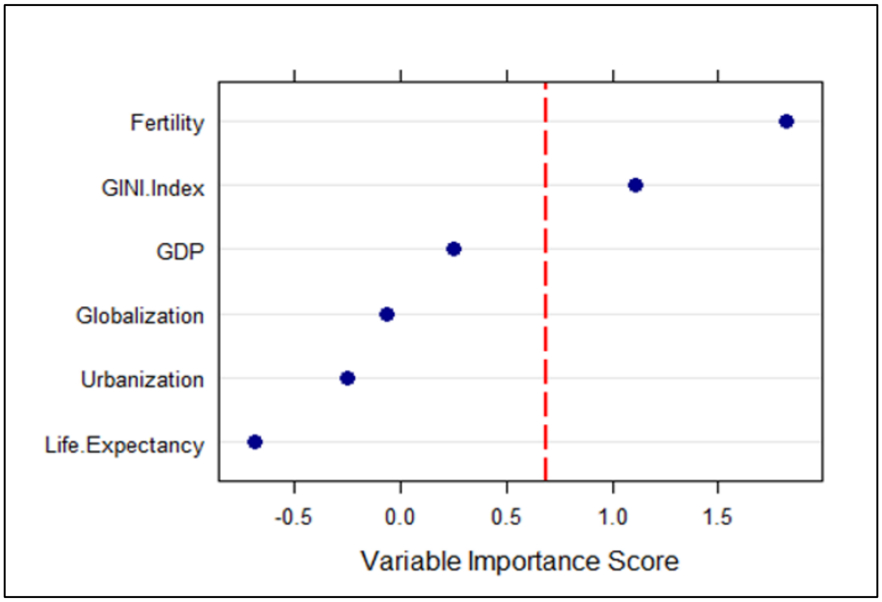

Figure S1: Predictors for underweight prevalence (RMSE = 5.6417).

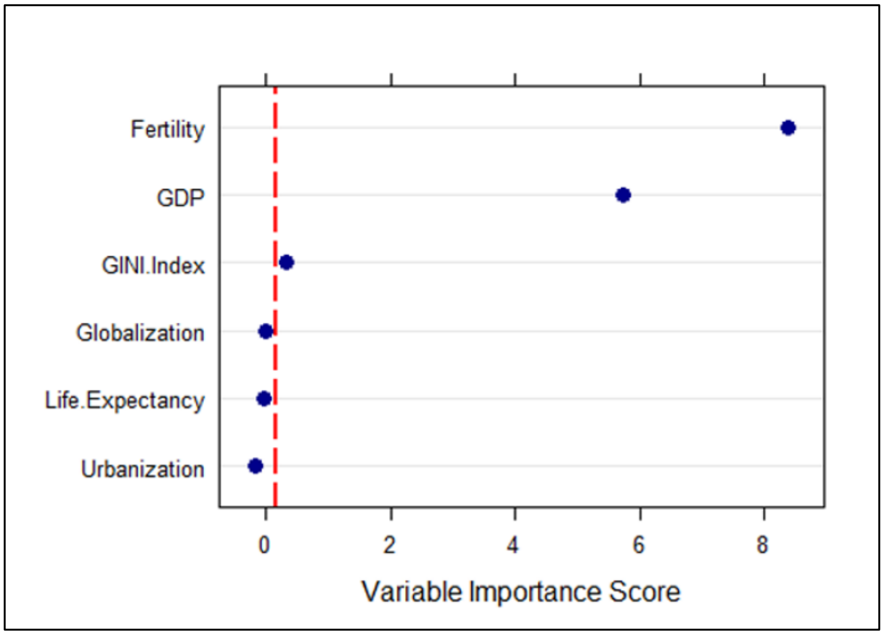

Figure S2: Predictors for overweight prevalence (RMSE=4.9637).

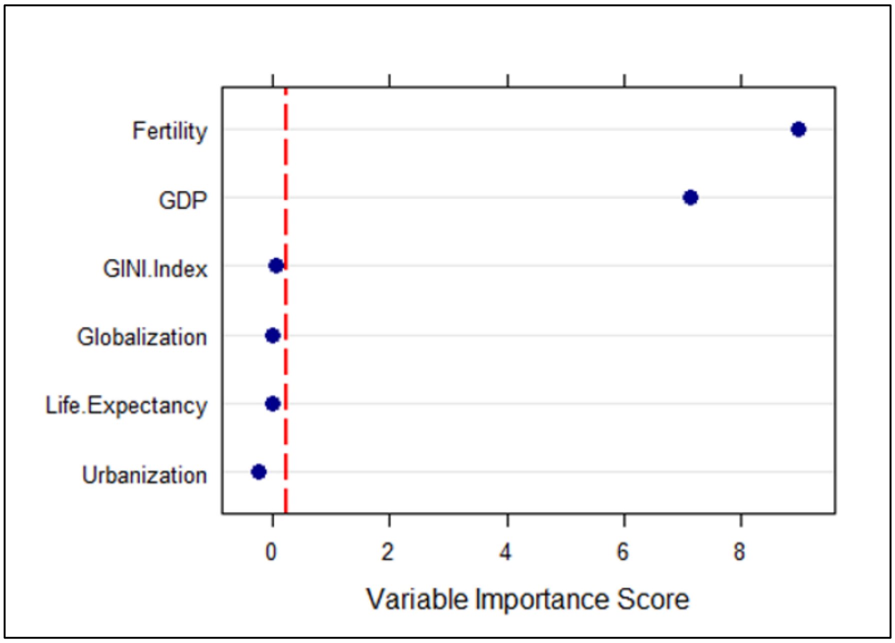

Figure S3: Predictors for obesity prevalence (RMSE = 4.2199).

Appendix 2: Descriptive statistics for individual-level data for each country included in the study (Tables S1 – S34).

Table S1: Descriptive statistics for the study population in Madagascar.

Variable	Sample Size n (%)
**Total Sample Size**	7,379
**Nutritional status**	
Underweight	1,985 (26.9)
Normal Weight	4,937 (66.9)
Overweight/Obesity	457 (6.2)
**Age**	
15-19	1,682 (22.8)
20-24	1,092 (14.8)
25-29	1,159 (15.7)
30-34	1,018 (13.8)
35-39	893 (12.1)
40-44	834 (11.3)
45-49	701 (9.5)
**Religion**	
Catholics	2,597 (35.2)
Other Christians	2,634 (35.7)
Islam	44 (0.6)
Traditionalist	162 (2.2)
Others	443 (6.0)
No Religion	1,498 (20.3)
**Parity**	
0	1,874 (25.4)
1	1,063 (14.4)
2	996 (13.5)
3	893 (12.1)
4	671 (9.1)
5	576 (7.8)
6+	1,306 (17.7)
**Marital Status**	
Single	1,468 (19.9)
Married	4,929 (66.8)
Formerly Married	981 (13.3)
**Residential Setting**	
Rural	6,088 (82.5)
Urban	1,291 (17.5)

Variable	Sample Size n (%)
**Region**	
Analamanga	1,299 (17.6)
Vakinankaratra	649 (8.8)
Itasy	303 (4.1)
Bongolava	214 (2.9)
Haute Matsiatra	391 (5.3)
Anamoroni'i mania	251 (3.4)
Vatovavy Fitovinany	362 (4.9)
Ihorombe	140 (1.9)
Atsimo Atsinanana	229 (3.1)
Atsinanana	472 (6.4)
Analanjirofo	369 (5.0)
Alaotra Mangoro	369 (5.0)
Boeny	258 (3.5)
Sofia	450 (6.1)
Betsiboka	103 (1.4)
Melaky	74 (1.0)
Atsimo Andrefana	354 (4.8)
Androy	170 (2.3)
Anosy	214 (2.9)
Menabe	170 (2.3)
Diana	199 (2.7)
Sava	339 (4.6
**Level of Education**	
No Education	1,358 (18.4)
Primary	3,594 (48.7)
Secondary	2,251 (30.5)
Higher	177 (2.4)
**Wealth Quintile**	
Poorest	1,254 (17.0)
Poorer	1,350 (18.3)
Middle	1,350 (18.3)
Richer	1,557 (21.1)
Richest	1,867 (25.3)
**Occupation**	
Not Working	1,173 (15.9)
Nonmanual	1,011 (13.7)
Manual	635 (8.6)
Agricultural	4,545 (61.6)
Others	15 (0.2)
**Media Exposure**	
Not Exposed	2,302 (31.2)
Exposed to one media source	2,885 (39.1)
Exposed to two media sources	1,181 (16.0)
Exposed to three media sources	1,011 (13.7)
**Hormonal Contraceptives use**	
No	5,726 (77.6)
Yes	1,653 (22.4)

Table S2: Descriptive statistics for the study population in Ethiopia.

Variable	Sample Size n (%)
**Total Sample Size**	13,222 (100)
**Nutritional status**	
Underweight	2,988 (20.6)
Normal Weight	9,229 (69.8)
Overweight/Obesity	1,005 (7.6)
**Age**	
15-19	3,015 (22.8)
20-24	2,182 (16.5)
25-29	2,367 (17.9)
30-34	1,904 (14.4)
35-39	1,626 (12.3)
40-44	1,164 (8.8)
45-49	965 (7.3)
**Ethnicity**	
Amhara	4,112 (31.1)
Affar	79 (0.6)
Guragie	383 (2.9)
Hadiye	304 (2.3)
Oromo	4,350 (32.9)
Sidama	542 (4.1)
Somali	331 (2.5)
Tigray	1,045 (7.9)
Welaita	410 (3.1)
Others	1,666 (12.6)
**Religion**	
Catholic	106 (0.8)
Other Christians	9,070 (68.6)
Islam	3,900 (29.5)
Others	145 (1.1)
**Parity**	
0	4,535 (34.3)
1	1,454 (11.0)
2	1,283 (9.7)
3	1,203 (9.1)
4	1,018 (7.7)
5	1,031 (7.8)
6+	2,697 (20.4)
**Marital Status**	
Single	3,755 (28.4)
Married	8,145 (61.6)
Formerly Married	1,322 (10.0)
**Residential Setting**	
Rural	10,207 (77.2)
Urban	3,015 (22.8)

Variable	Sample Size n (%)
**Region**	
Tigray	978 (7.4)
Afar	106 (0.8)
Amhara	3,279 (24.8)
Oromia	4,667 (35.3)
Somali	344 (2.6)
Benishangul	132 (1.0)
Snnpr	2,763 (20.9)
Gambela	40 (0.3)
Harari	26 (0.2)
Addis Adaba	820 (6.2)
Dire Dawa	66 (0.5)
**Level of Education**	
No Education	6,241 (47.2)
Primary	4,667 (35.3)
Secondary	1,560 (11.8)
Higher	754 (5.7)
**Wealth Quintile**	
Poorest	2,129 (16.1)
Poorer	2,274 (17.2)
Middle	2,565 (19.4)
Richer	2,671 (20.2)
Richest	3,583 (27.1)
**Occupation**	
Not Working	6,426 (48.6)
Nonmanual	2,935 (22.2)
Manual	740 (5.6)
Agricultural	2,790 (21.1)
Others	331 (2.5)
**Media Exposure**	
Not Exposed	7,351 (55.6)
Exposed to one media source	2,724 (20.6)
Exposed to two media sources	2,116 (16.0)
Exposed to three media sources	1,031 (7.8)
**Hormonal Contraceptives use**	
No	9,824 (74.3)
Yes	3,398 (25.7)

Table S3: Descriptive statistics for the study population in Burundi.

Variable	Sample Size n (%)
**Total Sample Size**	3,929 (100)
**Nutritional status**	
Underweight	636 (16.2)
Normal Weight	2,998 (76.3)
Overweight/Obesity	295 (7.5)
**Age**	
15-19	1,096 (27.9)
20-24	699 (17.8)
25-29	617 (15.7)
30-34	416 (10.6)
35-39	416 (10.6)
40-44	334 (8.5)
45-49	350 (8.9)
**Religion**	
Catholics	2,428 (61.8)
Other Christians	1,320 (33.6)
Islam	83 (2.1)
Others	98 (2.5)
**Parity**	
0	1,521 (38.7)
1	389 (9.9)
2	369 (9.4)
3	326 (8.3)
4	283 (7.2)
5	271 (6.9)
6+	770 (19.6)
**Marital Status**	
Single	1,493 (38.0)
Married	2,055 (52.3)
Formerly Married	381 (9.7)
**Residential Setting**	
Rural	3,509 (89.3)
Urban	420 (10.7)
**Region**	
Bujumbura	287 (7.3)
North	1,163 (29.6)
Centre East	963 (24.5)
West	648 (16.5)
South	868 (22.1)
**Level of Education**	
No Education	1,752 (44.6)
Primary	1,654 (42.1)
Secondary	491 (12.5)
Higher	31 (0.8)
**Wealth Quintile**	
Poorest	786 (20.0)
Poorer	778 (19.8)
Middle	758 (19.3)
Richer	790 (20.1)
Richest	817 (20.8)

Variable	Sample Size n (%)
**Occupation**	
Not Working	805 (20.5)
Nonmanual	251 (6.4)
Agricultural	2,833 (72.1)
Others	39 (1.0)
**Media Exposure**	
Not Exposed	723 (18.4)
Exposed to one media source	2,090 (53.2)
Exposed to two media sources	860 (21.9)
Exposed to three media sources	255 (6.5)
**Hormonal Contraceptives use**	
No	3,564 (90.7)
Yes	365 (9.3)

Table S4: Descriptive statistics for the study population in Burkina Faso.

Variable	Sample Size n (%)
**Total Sample Size**	7,212 (100)
**Nutritional status**	
Underweight	1,139 (15.8)
Normal Weight	5,265 (73.0)
Overweight/Obesity	808 (11.2)
**Age**	
15-19	1,500 (20.8)
20-24	1,298 (18.0)
25-29	1,161 (16.1)
30-34	1,067 (14.8)
35-39	858 (11.9)
40-44	692 (9.6)
45-49	635 (8.8)
**Ethnicity**	
Bobo	368 (5.1)
Fulfuldé / Peul	534 (7.4)
Gourmantché	454 (6.3)
Gourouns	325 (4.5)
Lobi	173 (2.4)
Mossi	3,765 (52.2)
Sénoufo	346 (4.8)
Dagara	195 (2.7)
Bissa	303 (4.2)
Others	750 (10.4)
**Religion**	
Catholic	1,709 (23.7)
Other Christians	512 (7.1)
Islam	4,421 (61.3)
Traditionalists	512 (7.1)
No Religion	58 (0.8)
**Parity**	
0	1,753 (24.3)
1	801 (11.1)
2	801 (11.1)
3	793 (11.0)
4	728 (10.1)
5	635 (8.8)
6+	1,702 (23.6)
**Marital Status**	
Single	1,457 (20.2)
Married	5,496 (76.2)
Formerly Married	260 (3.6)
**Residential Setting**	
Rural	5,171 (71.7)
Urban	2,041 (28.3)

Variable	Sample Size n (%)
**Region**	
Boucle de mouhoun	772 (10.7)
Cascades	274 (3.8)
Centre	1,118 (15.5)
Centre-Est	534 (7.4)
Centre-Nord	483 (6.7)
Centre-Ouest	555 (7.7)
Centre-Sud	317 (4.4)
Est	584 (8.1)
Hauts Basins	916 (12.7)
Nord	512 (7.1)
Plateau Central	339 (4.7)
Sahel	498 (6.9)
Sud-Ouest	310 (4.3)
**Level of Education**	
No Education	5,243 (72.7)
Primary	1,024 (14.2)
Secondary	865 (12.0)
Higher	79 (1.1)
**Wealth Quintile**	
Poorest	1,298 (18.0)
Poorer	1,320 (18.3)
Middle	1,320 (18.3)
Richer	1,414 (19.6)
Richest	1,861 (25.8)
**Occupation**	
Not Working	1,522 (21.1)
Nonmanual	1,760 (24.4)
Manual	4,76 (6.6)
Agricultural	3,296 (45.7)
Others	1,59 (2.2)
**Media Exposure**	
Not Exposed	1,846 (25.6)
Exposed to one media source	3,152 (43.7)
Exposed to two media sources	1,630 (22.6)
Exposed to three media sources	584 (8.1)
**Hormonal Contraceptives use**	
No	6,354 (88.1)
Yes	858 (11.9)

Table S5: Descriptive statistics for the study population in Chad.

Variable	Sample Size n (%)
**Total Sample Size**	8,873 (100)
**Nutritional status**	
Underweight	1,730 (19.5)
Normal Weight	6,131 (69.1)
Overweight/Obesity	1,012 (11.4)
**Age**	
15-19	2,032 (22.9)
20-24	1,366 (15.4)
25-29	1,544 (17.4)
30-34	1,224 (13.8)
35-39	1,065 (12.0)
40-44	816 (9.2)
45-49	825 (9.3)
**Ethnicity**	
Toupouri/Kéra	186 (2.1)
Gorane	515 (5.8)
Arab	816 (9.2)
Baguirmi/Barma	89 (1.0)
Kanembou/Bornou/Boudouma	896 (10.1)
Boulala/Médégo/Kouka	328 (3.7)
Ouadaï/Maba/Massalit/Mimi	710 (8.0)
Zaghawa/Bideyat/Kobé	106 (1.2)
Dadajo/Kibet/Mouro	204 (2.3)
Bidio/Migami/Kenga/Dangléat	213 (2.4)
Moundang	248 (2.8)
Massa/Mousseye/Mousgoume	444 (5.0)
Sara (Ngambaye/Sara Madjin- Gaye/Mbaye)	2,839 (32.0)
Peul/Foulbé/Bodoré	
Tama/Assongori/Mararit	142 (1.6)
Gabri/Kabalaye/Nangtchéré/ Soumraye	98 (1.1)
Marba/Lélé/Mesmé	160 (1.8)
Karo/Zimé/Pévé	328 (3.7)
Others	115 (1.3)
	435 (4.9)
**Religion**	
Catholic	1,872 (21.1)
Other Christians	2,227 (25.1)
Islam	4,507 (50.8)
Traditionalists	27 (0.3)
No Religion	240 (2.7)
**Parity**	
0	1,917 (21.6)
1	852 (9.6)
2	790 (8.9)
3	843 (9.5)
4	816 (9.2)
5	736 (8.3)
6+	2,919 (32.9)
**Marital Status**	
Single	1,686 (19.0)
Married	6,309 (71.1)
Formerly Married	878 (9.9)

Variable	Sample Size n (%)
**Residential Setting**	
Rural	6,752 (76.1)
Urban	2,121 (23.9)
**Region**	
Batha	319 (3.6)
Borkou, Tibesti	44 (0.5)
Chari Baguirmi	355 (4.0)
Guéra	453 (5.1)
Hadjer-Lamis	586 (6.6)
Kanem	346 (3.9)
Lac	506 (5.7)
Logone Occidental	612 (6.9)
Logone Oriental	896 (10.1)
Mandoul	621 (7.0)
Mayo Kebbi Est	665 (7.5)
Mayo Kebbi Ouest	479 (5.4)
Moyen Chari	506 (5.7)
Ouaddaï	479 (5.4)
Salamat	142 (1.6)
Tandjilé	532 (6.0)
Wadi Fira	240 (2.7)
N'Djaména	799 (9.0)
Barh El Gazal	115 (1.3)
Ennedi Est, Ennedi Ouest	44 (0.5)
Sila	133 (1.5)
**Level of Education**	
No Education	5,448 (61.4)
Primary	2,067 (23.3)
Secondary	1,278 (14.4)
Higher	80 (0.9)
**Wealth Quintile**	
Poorest	1,757 (19.8)
Poorer	1,677 (18.9)
Middle	1,712 (19.3)
Richer	1,810 (20.4)
Richest	1,917 (21.6)
**Occupation**	
Not Working	4,321 (48.7)
Nonmanual	3,106 (35.0)
Manual	115 (1.3)
Agricultural	1,269 (14.3)
Others	62 (0.7)
**Media Exposure**	
Not Exposed	6,273 (70.7)
Exposed to one media source	1,331 (15.0)
Exposed to two media sources	772 (8.7)
Exposed to three media sources	497 (5.6)
**Hormonal Contraceptives use**	
No	8,580 (96.7)
Yes	293 (3.3)

Table S6: Descriptive statistics for the study population in Democratic Republic of Congo.

Variable	Sample Size n (%)
**Total Sample Size**	7,655 (100)
**Nutritional status**	
Underweight	1,125 (14.7)
Normal Weight	5,305 (69.3)
Overweight/Obesity	1,225 (16.0)
**Age**	
15-19	1,782 (23.3)
20-24	1,323 (17.3)
25-29	1.301 (17.0)
30-34	1,008 (13.2)
35-39	900 (11.8)
40-44	733 (9.6)
45-49	608 (7.9)
**Ethnicity**	
Bakongo Nord & Sud	710 (9.3)
Bas Kasaï et Kwilu-Kwango	1,331 (17.4)
Cuvette central	707 (9.2)
Ubangi et Itimbiri	854 (11.2)
Uele Lac Albert	559 (7.3)
Basele-K, Man. et Kivu	1,509 (19.7)
Kasai, Katanga, Tanganyika	1,884 (24.6)
Others	100 (1.3)
**Religion**	
Catholic	2,339 (30.6)
Other Christians	5,088 (66.5)
Islam	73 (1.0)
Traditionalists	41 (0.5)
No Religion	115 (1.5)
**Parity**	
0	2,162 (28.2)
1	987 (12.9)
2	805 (10.5)
3	684 (8.9)
4	673 (8.8)
5	611 (8.0)
6+	1,733 (22.6)
**Marital Status**	
Single	2,287 (29.9)
Married	4,488 (58.6)
Formerly Married	880 (11.5)
**Residential Setting**	
Rural	4,749 (62.0)
Urban	2,905 (38.0)

Variable	Sample Size n (%)
**Region**	
Kinshasa	934 (12.2)
Bandundu	1,238 (16.2)
Bas-Congo	374 (4.9)
Equateur	987 (12.9)
Kasai-Occidental	480 (6.3)
Kasai-Oriental	781 (10.2)
Katanga	682 (8.9)
Maniema	234 (3.1)
Nord-Kivu	701 (9.2)
Orientale	726 (9.5)
Sud-Kivu	517 (6.8)
**Level of Education**	
No Education	1,210 (15.8)
Primary	2,771 (36.2)
Secondary	3,372 (44.1)
Higher	302 (4.0)
**Wealth Quintile**	
Poorest	1,409 (18.4)
Poorer	1,424 (18.6)
Middle	1,472 (19.2)
Richer	1,484 (19.4)
Richest	1,866 (24.4)
**Occupation**	
Not Working	2,169 (28.3)
Nonmanual	2,307 (30.1)
Manual	27 (0.4)
Agricultural	3,152 (41.2)
**Media Exposure**	
Not Exposed	3,841 (50.2)
Exposed to one media source	1,924 (25.1)
Exposed to two media sources	1,357 (17.7)
Exposed to three media sources	534 (7.0)
**Hormonal Contraceptives use**	
No	7,462 (97.5)
Yes	193 (2.5)

Table S7: Descriptive statistics for the study population in Niger.

Variable	Sample Size n (%)
**Total Sample Size**	4,104 (100)
**Nutritional status**	
Underweight	648 (15.8)
Normal Weight	2,733 (66.6)
Overweight/Obesity	722 (17.6)
**Age**	
15-19	718 (17.5)
20-24	669 (16.3)
25-29	743 (18.1)
30-34	657 (16.0)
35-39	550 (13.4)
40-44	402 (9.8)
45-49	365 (8.9)
**Parity**	
0	702 (17.1)
1	394 (9.6)
2	386 (9.4)
3	402 (9.8)
4	415 (10.1)
5	382 (9.3)
6+	1,424 (34.7)
**Marital Status**	
Single	435 (10.6)
Married	3,497 (85.2)
Formerly Married	172 (4.2)
**Residential Setting**	
Rural	3,316 (80.8)
Urban	788 (19.2)
**Region**	
Agadez	86 (2.1)
Diffa	127 (3.1)
Dosso	570 (13.9)
Maradi	841 (20.5)
Tahoua	936 (22.8)
Tillaberi	521 (12.7)
Zinder	681 (16.6)
Niamey	341 (8.3)
**Level of Education**	
No Education	3,279 (79.9)
Primary	476 (11.6)
Secondary	328 (8.0)
Higher	21 (0.5)
**Wealth Quintile**	
Poorest	718 (17.5)
Poorer	780 (19.0)
Middle	817 (19.9)
Richer	870 (21.2)
Richest	919 (22.4)
**Occupation**	
Not Working	2,963 (72.2)
Nonmanual	923 (22.5)
Manual	107 (2.6)
Agricultural	111 (2.7)

Variable	Sample Size n (%)
**Media Exposure**	
Not Exposed	1,395 (34.0)
Exposed to one media source	1,736 (42.3)
Exposed to two media sources	825 (20.1)
Exposed to three media sources	148 (3.6)
**Hormonal Contraceptives use**	
No	3,739 (91.1)
Yes	365 (8.9)

Table S8: Descriptive statistics for the study population in Mali.

Variable	Sample Size n (%)	
**Total Sample Size**	4,397	(100)
**Nutritional status**		
Underweight	510	(11.6)
Normal Weight	3,095	(70.4)
Overweight/Obesity	791	(18.0)
**Age**		
15-19	805	(18.3)
20-24	739	(16.8)
25-29	818	(18.6)
30-34	660	(15.0)
35-39	536	(12.2)
40-44	470	(10.7)
45-49	369	(8.4)
**Ethnicity**		
Bambara	1,508	(34.3)
Malinke	317	(7.2)
Peulh	651	(14.8)
Sarakole/Soninke/Marka	506	(11.5)
Dogon	413	(9.4)
Sénoufo/Minianka	466	(10.6)
Others	536	(12.2)
**Religion**		
Catholics	119	(2.7)
Other Christians	75	(1.7)
Islam	4,063	(92.4)
Traditionalist	31	(0.7)
No Religion	110	(2.5)
**Parity**		
0	835	(19.0)
1	541	(12.3)
2	563	(12.8)
3	506	(11.5)
4	497	(11.3)
5	470	(10.7)
6+	985	(22.4)
**Marital Status**		
Single	682	(15.5)
Married	3,619	(82.3)
Formerly Married	97	(2.2)
**Residential Setting**		
Rural	3,258	(74.1)
Urban	1,139	(25.9)
**Region**		
Kayes	563	(12.8)
Sikasso	923	(21.0)
Koulikoro	1,011	(23.0)
Segou	791	(18.0)
Mopti	519	(11.8)
Bamako	589	(13.4)

Variable	Sample Size n (%)	
**Level of Education**		
No Education	3,280	(74.6)
Primary	413	(9.4)
Secondary	642	(14.6)
Higher	62	(1.4)
**Wealth Quintile**		
Poorest	835	(19.0)
Poorer	796	(18.1)
Middle	831	(18.9)
Richer	871	(19.8)
Richest	1,064	(24.2)
**Occupation**		
Not Working	2,220	(50.5)
Nonmanual	893	(20.3)
Manual	616	(14.0)
Agricultural	668	(15.2)
**Media Exposure**		
Not Exposed	1,148	(26.1)
Exposed to one media source	1,161	(26.4)
Exposed to two media sources	1,732	(39.4)
Exposed to three media sources	356	(8.1)
**Hormonal Contraceptives use**		
No	3,927	(89.3)
Yes	470	(10.7)

Table S9: Descriptive statistics for the study population in Gambia.

Variable	Sample Size n (%)
**Total Sample Size**	3,886 (100)
**Nutritional status**	
Underweight	653 (16.8)
Normal Weight	2,359 (60.7)
Overweight/Obesity	874 (22.5)
**Age**	
15-19	940 (24.2)
20-24	797 (20.5)
25-29	665 (17.1)
30-34	567 (14.6)
35-39	389 (10.0)
40-44	299 (7.7)
45-49	229 (5.9)
**Ethnicity**	
Mandinka/Jahanka	1,348 (34.7)
Wollof	536 (13.8)
Jola/Karoninka	400 (10.3)
Fula/Tukulur/Lorobo	878 (22.6)
Serere	109 (2.8)
Serahuleh	241 (6.2)
Others	190 (4.9)
Non-Gambian	183 (4.7)
**Religion**	
Other Christians	144 (3.7)
Islam	3,742 (96.3)
**Parity**	
0	1364 (35.1)
1	497 (12.8)
2	427 (11.0)
3	354 (9.1)
4	303 (7.8)
5	295 (7.6)
6+	645 (16.6)
**Marital Status**	
Single	1,209 (31.1)
Married	2,456 (63.2)
Formerly Married	222 (5.7)
**Residential Setting**	
Rural	1,714 (44.1)
Urban	2,172 (55.9)
**Region**	
Banjul	85 (2.2)
Kanifing	902 (23.2)
Brikama	1,310 (33.7)
Mansakonko	190 (4.9)
Kerewan	443 (11.4)
Kuntaur	194 (5.0)
Janjanbureh	284 (7.3)
Base	478 (12.3)

Variable	Sample Size n (%)	
**Level of Education**		
No Education	1,791	(46.1)
Primary	521	(13.4)
Secondary	1,368	(35.2)
Higher	206	(5.3)
**Wealth Quintile**		
Poorest	645	(16.6)
Poorer	754	(19.4)
Middle	653	(16.8)
Richer	851	(21.9)
Richest	983	(25.3)
**Occupation**		
Not Working	1,892	(48.7)
Nonmanual	1,053	(27.1)
Manual	89	(2.3)
Agricultural	851	(21.9)
**Media Exposure**		
Not Exposed	330	(8.5)
Exposed to one media source	968	(24.9)
Exposed to two media sources	1,943	(50.0)
Exposed to three media sources	645	(16.6)
**Hormonal Contraceptives use**		
No	3,633	(93.5)
Yes	253	(6.5)

Table S10: Descriptive statistics for the study population in Uganda.

Variable	Sample Size n (%)	
**Total Sample Size**	2,308	(100)
**Nutritional status**		
Underweight	275	(11.9)
Normal Weight	1,606	(69.6)
Overweight/Obesity	429	(18.6)
**Age**		
15-19	586	(25.4)
20-24	372	(16.1)
25-29	406	(17.6)
30-34	275	(11.9)
35-39	291	(12.6)
40-44	194	(8.4)
45-49	185	(8.0)
**Ethnicity**		
Baganda	411	(17.8)
Banyankole	226	(9.8)
Basoga	185	(8.0)
Bakiga	180	(7.8)
Iteso	164	(7.1)
Others	1142	(49.5)
**Religion**		
Catholic	972	(42.1)
Other Christians	1,022	(44.3)
Islam	284	(12.3)
Others	30	(1.3)
**Parity**		
0	655	(28.4)
1	201	(8.7)
2	238	(10.3)
3	217	(9.4)
4	231	(10.0)
5	173	(7.5)
6+	593	(25.7)
**Marital Status**		
Single	676	(29.3)
Married	1,322	(57.3)
Formerly Married	309	(13.4)
**Residential Setting**		
Rural	1,807	(78.3)
Urban	501	(21.7)
**Region**		
Kampala	242	(10.5)
Central 1	245	(10.6)
Central 2	231	(10.0)
East Central	219	(9.5)
Eastern	337	(14.6)
North	192	(8.3)
Karamoja	65	(2.8)
West-Nile	138	(6.0)
Western	332	(14.4)
South West	307	(13.3)

Variable	Sample Size n (%)	
**Level of Education**		
No Education	270	(11.7)
Primary	1,376	(59.6)
Secondary	531	(23.0)
Higher	132	(5.7)
**Wealth Quintile**		
Poorest	379	(16.4)
Poorer	383	(16.6)
Middle	420	(18.2)
Richer	503	(21.8)
Richest	623	(27.0)
**Occupation**		
Not Working	632	(27.4)
Nonmanual	554	(24.0)
Agricultural	1,122	(48.6)
**Media Exposure**		
Not Exposed	291	(12.6)
Exposed to one media source	1,013	(43.9)
Exposed to two media sources	619	(26.8)
Exposed to three media sources	385	(16.7)
**Hormonal Contraceptives use**		
No	1,872	(81.1)
Yes	436	(18.9)

Table S11: Descriptive statistics for the study population in Guinea.

Variable	Sample Size n (%)
**Total Sample Size**	4,038 (100)
**Nutritional status**	
Underweight	497 (12.3)
Normal Weight	2,758 (68.3)
Overweight/Obesity	783 (19.4)
**Age**	
15-19	969 (24.0)
20-24	690 (17.1)
25-29	630 (15.6)
30-34	509 (12.6)
35-39	501 (12.4)
40-44	396 (9.8)
45-49	343 (8.5)
**Ethnicity**	
Soussou	820 (20.3)
Peulh	1,300 (32.2)
Malinké	1,244 (30.8)
Kissi	190 (4.7)
Toma	113 (2.8)
Guerzé	258 (6.4)
Other	113 (2.8)
**Religion**	
Other Christians	371 (9.2)
Islam	3,477 (86.1)
No Religion	190 (4.7)
**Parity**	
0	1,094 (27.1)
1	553 (13.7)
2	472 (11.7)
3	359 (8.9)
4	384 (9.5)
5	363 (9.0)
6+	812 (20.1)
**Marital Status**	
Single	1,078 (26.7)
Married	2,774 (68.7)
Formerly Married	186 (4.6)
**Residential Setting**	
Rural	2,556 (63.3)
Urban	1,482 (36.7)
**Region**	
Boké	412 (10.2)
Conakry	852 (21.1)
Faranah	335 (8.3)
Kankan	521 (12.9)
Kindia	557 (13.8)
Labé	363 (9.0)
Mamou	258 (6.4)
N'Zérékoré	739 (18.3)

Variable	Sample Size n (%)	
**Level of Education**		
No Education	2,625	(65.0)
Primary	573	(14.2)
Secondary	711	(17.6)
Higher	129	(3.2)
**Wealth Quintile**		
Poorest	690	(17.1)
Poorer	763	(18.9)
Middle	755	(18.7)
Richer	848	(21.0)
Richest	981	(24.3)
**Occupation**		
Not Working	1,070	(26.5)
Nonmanual	1,175	(29.1)
Manual	258	(6.4)
Agricultural	1,534	(38.0)
**Media Exposure**		
Not Exposed	1,175	(29.1)
Exposed to one media source	1,195	(29.6)
Exposed to two media sources	1,333	(33.0)
Exposed to three media sources	335	(8.3)
**Hormonal Contraceptives use**		
No	3,881	(96.1)
Yes	157	(3.9)

Table S12: Descriptive statistics for the study population in Senegal.

Variable	Sample Size n (%)
**Total Sample Size**	5,020 (100)
**Nutritional status**	
Underweight	1,109 (22.1)
Normal Weight	2,841 (56.6)
Overweight/Obesity	1,069 (21.3)
**Age**	
15-19	1,195 (23.8)
20-24	974 (19.4)
25-29	813 (16.2)
30-34	648 (12.9)
35-39	532 (10.6)
40-44	502 (10.0)
45-49	356 (7.1)
**Ethnicity**	
Wolof	2,018 (40.2)
Poular	1,205 (24.0)
Serer	738 (14.7)
Mandingue	186 (3.7)
Diola	216 (4.3)
Soninke	141 (2.8)
Not a Senegalese	126 (2.5)
Others	392 (7.8)
**Religion**	
Islam	4,774 (95.1)
Other	246 (4.9)
**Parity**	
0	1,888 (37.6)
1	633 (12.6)
2	527 (10.5)
3	427 (8.5)
4	366 (7.3)
5	326 (6.5)
6+	853 (17.0)
**Marital Status**	
Single	1,642 (32.7)
Married	3,132 (62.4)
Formerly Married	246 (4.9)
**Residential Setting**	
Rural	2,480 (49.4)
Urban	2,540 (50.6)

Variable	Sample Size n (%)
**Region**	
Dakar	1,335 (26.6)
Ziguinchor	201 (4.0)
Diourbel	592 (11.8)
Saint-Louis	301 (6.0)
Tambacounda	206 (4.1)
Kaolack	382 (7.6)
Thiès	673 (13.4)
Louga	361 (7.2)
Fatick	236 (4.7)
Kolda	196 (3.9)
Matam	186 (3.7)
Kaffrine	186 (3.7)
Kedougou	35 (0.7)
Sedhiou	131 (2.6)
**Level of Education**	
No Education	2,791 (55.6)
Primary	1,089 (21.7)
Secondary	1,049 (20.9)
Higher	90 (1.8)
**Wealth Quintile**	
Poorest	768 (15.3)
Poorer	838 (16.7)
Middle	1,029 (20.5)
Richer	1,130 (22.5)
Richest	1,255 (25.0)
**Occupation**	
Not Working	2666 (53.1)
Nonmanual	1,822 (36.3)
Manual	85 (1.7)
Agricultural	447 (8.9)
**Media Exposure**	
Not Exposed	361 (7.2)
Exposed to one media source	1,064 (21.2)
Exposed to two media sources	2,535 (50.5)
Exposed to three media sources	1,059 (21.1)
**Hormonal Contraceptives use**	
No	4,598 (91.6)
Yes	422 (8.4)

Table S13: Descriptive statistics for the study population in Namibia.

Variable	Sample Size n (%)
**Total Sample Size**	3,921 (100)
**Nutritional status**	
Underweight	549 (14.0)
Normal Weight	2,137 (54.5)
Overweight/Obesity	1,235 (31.5)
**Age**	
15-19	823 (21.0)
20-24	753 (19.2)
25-29	612 (15.6)
30-34	514 (13.1)
35-39	498 (12.7)
40-44	392 (10.0)
45-49	329 (8.4)
**Religion**	
Catholic	772 (19.7)
Other Christians	2,776 (70.8)
Others	329 (8.4)
No Religion	43 (1.1)
**Parity**	
0	1,274 (32.5)
1	769 (19.6)
2	698 (17.8)
3	478 (12.2)
4	294 (7.5)
5	176 (4.5)
6+	231 (5.9)
**Marital Status**	
Single	2,368 (60.4)
Married	1,259 (32.1)
Formerly Married	294 (7.5)
**Residential Setting**	
Rural	1,792 (45.7)
Urban	2,129 (54.3)
**Region**	
Caprivi	208 (5.3)
Erongo	333 (8.5)
Hardap	149 (3.8)
Karas	157 (4.0)
Kavango	341 (8.7)
Khomas	823 (21.0)
Kunene	110 (2.8)
Ohangwena	412 (10.5)
Omaheke	102 (2.6)
Omusati	388 (9.9)
Oshana	353 (9.0)
Oshikoto	310 (7.9)
Otjozondjupa	235 (6.0)
**Level of Education**	
No Education	184 (4.7)
Primary	788 (20.1)
Secondary	2,604 (66.4)
Higher	345 (8.8)

Variable	Sample Size n (%)	
**Wealth Quintile**		
Poorest	623	(15.9)
Poorer	706	(18.0)
Middle	765	(19.5)
Richer	949	(24.2)
Richest	878	(22.4)
**Occupation**		
Not Working	2,180	(55.6)
Nonmanual	1,557	(39.7)
Manual	125	(3.2)
Agricultural	59	(1.5)
**Media Exposure**		
Not Exposed	314	(8.0)
Exposed to one media source	772	(19.7)
Exposed to two media sources	1,243	(31.7)
Exposed to three media sources	1,592	(40.6)
**Hormonal Contraceptives use**		
No	2,843	(72.5)
Yes	1,078	(27.5)

Table S14: Descriptive statistics for the study population in Congo.

Variable	Sample Size n (%)
**Total Sample Size**	4,822 (100)
**Nutritional status**	
Underweight	694 (14.4)
Normal Weight	2,864 (59.4)
Overweight/Obesity	1,263 (26.2)
**Age**	
15-19	1,022 (21.2)
20-24	853 (17.7)
25-29	815 (16.9)
30-34	709 (14.7)
35-39	617 (12.8)
40-44	424 (8.8)
45-49	381 (7.9)
**Ethnicity**	
Kongo	2,667 (55.3)
Punu	222 (4.6)
Duma	43 (0.9)
Mbéré/Mbéti/Kélé	101 (2.1)
Téké	632 (13.1)
Mbochi	526 (10.9)
Sangha	121 (2.5)
Oubanguiens	39 (0.8)
Pygmée	29 (0.6)
Etranger	391 (8.1)
Others	53 (1.1)
**Religion**	
Catholic	1,524 (31.6)
Other Christians	2,831 (58.7)
Islam	53 (1.1)
Traditionalists	227 (4.7)
Other	29 (0.6)
No Religion	159 (3.3)
**Parity**	
0	1,133 (23.5)
1	767 (15.9)
2	815 (16.9)
3	694 (14.4)
4	501 (10.4)
5	328 (6.8)
6+	583 (12.1)
**Marital Status**	
Single	1,297 (26.9)
Married	2,676 (55.5)
Formerly Married	849 (17.6)
**Residential Setting**	
Rural	1,533 (31.8)
Urban	3,289 (68.2)

Variable	Sample Size n (%)	
**Region**		
Kouilou	130	(2.7)
Niari	246	(5.1)
Lekoumou	87	(1.8)
Bouenza	530	(11.0)
Pool	280	(5.8)
Plateaux	140	(2.9)
Cuvette	149	(3.1)
Cuvette - Ouest	58	(1.2)
Sangha	77	(1.6)
Likouala	193	(4.0)
Brazzaville	1,606	(33.3)
Pointe-noire	1,326	(27.5)
**Level of Education**		
No Education	265	(5.5)
Primary	1,186	(24.6)
Secondary	3,139	(65.1)
Higher	231	(4.8)
**Wealth Quintile**		
Poorest	810	(16.8)
Poorer	979	(20.3)
Middle	1,032	(21.4)
Richer	964	(20.0)
Richest	1,037	(21.5)
**Occupation**		
Not Working	1,639	(34.0)
Nonmanual	1,967	(40.8)
Manual	236	(4.9)
Agricultural	935	(19.4)
Other	43	(0.9)
**Media Exposure**		
Not Exposed	1,259	(26.1)
Exposed to one media source	1,331	(27.6)
Exposed to two media sources	1,379	(28.6)
Exposed to three media sources	853	(17.7)
**Hormonal Contraceptives use**		
No	4,576	(94.9)
Yes	246	(5.1)

Table S15: Descriptive statistics for the study population in Nigeria.

Variable	Sample Size n (%)
**Total Sample Size**	32,078 (100)
**Nutritional status**	
Underweight	3,689 (11.5)
Normal Weight	20,466 (63.8)
Overweight/Obesity	7,923 (24.7)
**Age**	
15-19	6,833 (21.3)
20-24	5,229 (16.3)
25-29	5,357 (16.7)
30-34	4,202 (13.1)
35-39	3,914 (12.2)
40-44	3,240 (10.1)
45-49	3,304 (10.3)
**Ethnicity**	
Ekoi	32 (0.1)
Fulani	1,989 (6.2)
Hausa	8,372 (26.1)
Ibibio	770 (2.4)
Igala	321 (1.0)
Igbo	4,876 (15.2)
Ijaw/Izon	642 (2.0)
Kanuri/Beriberi	577 (1.8)
Tiv	642 (2.0)
Yoruba	4,812 (15.0)
Others	9,046 (28.2)
**Religion**	
Other Christians	12,061 (37.6)
Catholics	3,721 (11.6)
Islam	16,007 (49.9)
Traditionalists	289 (0.9)
**Parity**	
0	10,105 (31.5)
1	3,272 (10.2)
2	3,079 (9.6)
3	3,047 (9.5)
4	2,983 (9.3)
5	2,598 (8.1)
6+	6,993 (21.8)
**Marital Status**	
Single	8,950 (27.9)
Married	21,460 (66.9)
Formerly Married	1,668 (5.2)
**Residential Setting**	
Rural	18,060 (56.3)
Urban	14,018 (43.7)

Variable	Sample Size n (%)
**Region**	
Sokoto	866 (2.7)
Zamfara	1.026 (3.2)
Katsina	1,155 (3.6)
Jigawa	1,091 (3.4)
Yobe	770 (2.4)
Borno	1,123 (3.5)
Adamawa	642 (2.0)
Gombe	417 (1.3)
Bauchi	866 (2.7)
Kano	2,598 (8.1)
Kaduna	1,604 (5.0)
Kebbi	962 (3.0)
Niger	1,123 (3.5)
Fct-Abuja	289 (0.9)
Nasarawa	481 (1.5)
Plateau	545 (1.7)
Taraba	706 (2.2)
Benue	994 (3.1)
Kogi	609 (1.9)
Kwara	545 (1.7)
Oyo	1,315 (4.1)
Osun	706 (2.2)
Ekiti	289 (0.9)
Ondo	706 (2.2)
Edo	674 (2.1)
Anambra	898 (2.8)
Enugu	834 (2.6)
Ebonyi	962 (3.0)
Cross River	609 (1.9)
Akwa Ibom	770 (2.4)
Abia	449 (1.4)
Imo	738 (2.3)
Rivers	1,091 (3.4)
Bayelsa	289 (0.9)
Delta	866 (2.7)
Lagos	1,732 (5.4)
Ogun	738 (2.3)
**Level of Education**	
No Education	11,484 (35.8)
Primary	5,517 (17.2)
Secondary	11,965 (37.3)
Higher	3,112 (9.7)
**Wealth Quintile**	
Poorest	5,485 (17.1)
Poorer	5,870 (18.3)
Middle	6,223 (19.4)
Richer	6,768 (21.1)
Richest	7,731 (24.1)
**Occupation**	
Not Working	11,805 (36.8)
Nonmanual	14,211 (44.3)
Manual	2,855 (8.9)
Agricultural	3,176 (9.9)
Others	32 (0.1)

Variable	Sample Size n (%)
**Media Exposure**	
Not Exposed	8,565 (26.7)
Exposed to one media source	6,833 (21.3)
Exposed to two media sources	10,201 (31.8)
Exposed to three media sources	6,480 (20.2)
**Hormonal Contraceptives use**	
No	30,314 (94.5)
Yes	1,764 (5.5)

Table S16: Descriptive statistics for the study population in Zambia.

Variable	Sample Size n (%)
**Total Sample Size**	14,129 (100)
**Nutritional status**	
Underweight	1,455 (10.3)
Normal Weight	9,424 (66.7)
Overweight/Obesity	3,250 (23.0)
**Age**	
15-19	3,221 (22.8)
20-24	2,458 (17.4)
25-29	2,303 (16.3)
30-34	2,049 (14.5)
35-39	1,766 (12.5)
40-44	1,356 (9.6)
45-49	975 (6.9)
**Ethnicity**	
Bemba	3,447 (24.4)
Tonga	1,950 (13.8)
Chewa	1,046 (7.4)
Lozi	819 (5.8)
Nsenga	763 (5.4)
Tumbuka	706 (5.0)
Ngoni	622 (4.4)
Lala	438 (3.1)
Kaonde	353 (2.5)
Namwanga	438 (3.1)
Mambwe	410 (2.9)
Lunda(North-Western)	283 (2.0)
Luvale	325 (2.3)
Others	2,529 (17.9)
**Religion**	
Catholic	2,600 (18.4)
Other Christians	11,374 (80.5)
Islam	85 (0.6)
Other	71 (0.5)
**Parity**	
0	3,561 (25.2)
1	1,936 (13.7)
2	1,794 (12.7)
3	1,597 (11.3)
4	1,342 (9.5)
5	1,116 (7.9)
6+	2,783 (19.7)
**Marital Status**	
Single	4,253 (30.1)
Married	8,054 (57.0)
Formerly Married	1,823 (12.9)
**Residential Setting**	
Rural	7,404 (52.4)
Urban	6,725 (47.6)

Variable	Sample Size n (%)	
**Region**		
Central	1,257	(8.9)
Copperbelt	2,487	(17.6)
Eastern	1,653	(11.7)
Luapula	933	(6.6)
Lusaka	2,896	(20.5)
Muchinga	735	(5.2)
Northern	989	(7.0)
North Western	608	(4.3)
Southern	1,724	(12.2)
Western	848	(6.0)
**Level of Education**		
No Education	1,116	(7.9)
Primary	6,542	(46.3)
Secondary	5,736	(40.6)
Higher	735	(5.2)
**Wealth Quintile**		
Poorest	2,360	(16.7)
Poorer	2,374	(16.8)
Middle	2,642	(18.7)
Richer	3,052	(21.6)
Richest	3,702	(26.2)
**Occupation**		
Not Working	6,796	(48.1)
Nonmanual	3,546	(25.1)
Manual	170	(1.2)
Agricultural	3,419	(24.2)
Other	198	(1.4)
**Media Exposure**		
Not Exposed	3,405	(24.1)
Exposed to one media source	3,914	(27.7)
Exposed to two media sources	3,716	(26.3)
Exposed to three media sources	3,094	(21.9)
**Hormonal Contraceptives use**		
No	9,989	(70.7)
Yes	4,140	(29.3)

Table S17: Descriptive statistics for the study population in Sierra Leone.

Variable	Sample Size n (%)
**Sample Size**	6,971 (100)
**Nutritional status**	
Underweight	641 (9.2)
Normal Weight	5,047 (72.4)
Overweight/Obesity	1,283 (18.4)
**Age**	
15-19	1,555 (22.3)
20-24	1,087 (15.6)
25-29	1,150 (16.5)
30-34	941 (13.5)
35-39	997 (14.3)
40-44	606 (8.7)
45-49	634 (9.1)
**Ethnicity**	
Creole	77 (1.1)
Fullah	230 (3.3)
Kono	293 (4.2)
Limba	432 (6.2)
Loko	230 (3.3)
Mandigo	174 (2.5)
Mende	2,307 (33.1)
Sherbro	174 (2.5)
Temne	2,489 (35.7)
Koranko	195 (2.8)
Others	369 (5.3)
**Religion**	
Other Christians	1,506 (21.6)
Islam	5,451 (78.2)
Other	14 (0.2)
**Parity**	
0	1,743 (25.0)
1	941 (13.5)
2	857 (12.3)
3	753 (10.8)
4	753 (10.8)
5	641 (9.2)
6+	1,283 (18.4)
**Marital Status**	
Single	2,036 (29.2)
Married	4,434 (63.6)
Formerly Married	502 (7.2)
**Residential Setting**	
Rural	4,392 (63.0)
Urban	2,579 (37.0)
**Region**	
Eastern	1,471 (21.1)
Northern	2,642 (37.9)
Southern	1,443 (20.7)
Western	1,415 (20.3)

Variable	Sample Size n (%)	
**Level of Education**		
No Education	3,834	(55.0)
Primary	955	(13.7)
Secondary	1,952	(28.0)
Higher	230	(3.3)
**Wealth Quintile**		
Poorest	1,234	(17.7)
Poorer	1,283	(18.4)
Middle	1,331	(19.1)
Richer	1,387	(19.9)
Richest	1,736	(24.9)
**Occupation**		
Not Working	1,980	(28.4)
Nonmanual	383	(5.5)
Manual	1,903	(27.3)
Agricultural	2,705	(38.8)
**Media Exposure**		
Not Exposed	2,363	(33.9)
Exposed to one media source	3,046	(43.7)
Exposed to two media sources	1,074	(15.4)
Exposed to three media sources	488	(7.0)
**Hormonal Contraceptives use**		
No	5556	(79.7)
Yes	1415	(20.3)

Table S18: Descriptive statistics for the study population in Mozambique.

Variable	Sample Size n (%)
**Total Sample Size**	11,617 (100)
**Nutritional status**	
Underweight	999 (8.6)
Normal Weight	8,713 (75.0)
Overweight/Obesity	1,905 (16.4)
**Age**	
15-19	2,591 (22.3)
20-24	1,917 (16.5)
25-29	1,859 (16.0)
30-34	1,626 (14.0)
35-39	1,464 (12.6)
40-44	1,080 (9.3)
45-49	1,080 (9.3)
**Ethnicity**	
Emakhuwa	2,416 (20.8)
Português	1,104 (9.5)
Xichangana	1,626 (14.0)
Cisena	1,150 (9.9)
Elomwe	871 (7.5)
Echuwabo	732 (6.3)
Cinyanja	883 (7.6)
Cindau	569 (4.9)
Xitswa	488 (4.2)
Cinyungwe	372 (3.2)
Ciyao	256 (2.2)
Others	1,150 (9.9)
**Religion**	
Catholic	3,415 (29.4)
Other Christians	4,891 (42.1)
Islam	1,975 (17.0)
Other	256 (2.2)
No Religion	1,080 (9.3)
**Parity**	
0	2,602 (22.4)
1	1,801 (15.5)
2	1,603 (13.8)
3	1,382 (11.9)
4	1,255 (10.8)
5	918 (7.9)
6+	2,056 (17.7)
**Marital Status**	
Single	2,335 (20.1)
Married	7,551 (65.0)
Formerly Married	1,731 (14.9)
**Residential Setting**	
Rural	7,458 (64.2)
Urban	4,159 (35.8)

Variable	Sample Size n (%)	
**Region**		
Niassa	534	(4.6)
Cabo Delgado	836	(7.2)
Nampula	1,580	(13.6)
Zambezia	2,068	(17.8)
Tete	1,336	(11.5)
Manica	802	(6.9)
Sofala	1,220	(10.5)
Inhambane	755	(6.5)
Gaza	720	(6.2)
Maputo Provincia	953	(8.2)
Maputo Cidade	813	(7.0)
**Level of Education**		
No Education	3,590	(30.9)
Primary	5,762	(49.6)
Secondary	2,091	(18.0)
Higher	174	(1.5)
**Wealth Quintile**		
Poorest	2,103	(18.1)
Poorer	2,091	(18.0)
Middle	2,161	(18.6)
Richer	2,358	(20.3)
Richest	2,904	(25.0)
**Occupation**		
Not Working	6,145	(52.9)
Nonmanual	1,928	(16.6)
Manual	128	(1.1)
Agricultural	3,415	(29.4)
**Media Exposure**		
Not Exposed	3,288	(28.3)
Exposed to one media source	4,531	(39.0)
Exposed to two media sources	2,451	(21.1)
Exposed to three media sources	1,348	(11.6)
**Hormonal Contraceptives use**		
No	10,455	(90.0)
Yes	1.162	(10.0)

Table S19: Descriptive statistics for the study population in Malawi.

Variable	Sample Size n (%)
**Sample Size**	7,179 (100)
**Nutritional status**	
Underweight	517 (7.2)
Normal Weight	5,176 (72.1)
Overweight/Obesity	1,486 (20.7)
**Age**	
15-19	1,543 (21.5)
20-24	1,407 (19.6)
25-29	1,149 (16.0)
30-34	1,084 (15.1)
35-39	847 (11.8)
40-44	646 (9.0)
45-49	503 (7.0)
**Ethnicity**	
Chewa	2,462 (34.3)
Tombuka	646 (9.0)
Lomwe	1,436 (20.0)
Tonga	122 (1.7)
Yao	969 (13.5)
Sena	244 (3.4)
Nkhonde	65 (0.9)
Ngoni	840 (11.7)
Mang'anja	194 (2.7)
Nyanja	79 (1.1)
Others	122 (1.7)
**Religion**	
Catholic	1,299 (18.1)
Other Christians	4,946 (68.9)
Islam	890 (12.4)
Other	7 (0.1)
No Religion	36 (0.5)
**Parity**	
0	1,608 (22.4)
1	1,041 (14.5)
2	1,055 (14.7)
3	948 (13.2)
4	768 (10.7)
5	632 (8.8)
6+	1,127 (15.7)
**Marital Status**	
Single	1,622 (22.6)
Married	4,551 (63.4)
Formerly Married	1,005 (14.0)
**Residential Setting**	
Rural	5,851 (81.5)
Urban	1,328 (18.5)
**Region**	
Northern	818 (11.4)
Central	3,044 (42.4)
Southern	3,317 (46.2)

Variable	Sample Size n (%)	
**Level of Education**		
No Education	883	(12.3)
Primary	4,365	(60.8)
Secondary	1,701	(23.7)
Higher	230	(3.2)
**Wealth Quintile**		
Poorest	1,328	(18.5)
Poorer	1,386	(19.3)
Middle	1,357	(18.9)
Richer	1,371	(19.1)
Richest	1,737	(24.2)
**Occupation**		
Not Working	2,290	(31.9)
Nonmanual	948	(13.2)
Manual	1,084	(15.1)
Agricultural	2,857	(39.8)
**Media Exposure**		
Not Exposed	3,152	(43.9)
Exposed to one media source	2,125	(29.6)
Exposed to two media sources	1,228	(17.1)
Exposed to three media sources	675	(9.4)
**Hormonal Contraceptives use**		
No	4,530	(63.1)
Yes	2,649	(36.9)

Table S20: Descriptive statistics for the study population in Ivory Coast.

Variable	Sample Size n (%)	
**Sample Size**	4,057	(100)
**Nutritional status**		
Underweight	316	(7.8)
Normal Weight	2,698	(66.5)
Overweight/Obesity	1,043	(25.7)
**Age**		
15-19	876	(21.6)
20-24	759	(18.7)
25-29	722	(17.8)
30-34	548	(13.5)
35-39	471	(11.6)
40-44	361	(8.9)
45-49	321	(7.9)
**Ethnicity**		
Akan	1,266	(31.2)
Krou	430	(10.6)
Mandé du nord	519	(12.8)
Mandé du sud	369	(9.1)
Voltaïque/Gur	609	(15.0)
Autres nationalités	864	(21.3)
**Religion**		
Catholic	755	(18.6)
Other Christians	1,067	(26.3)
Islam	1,639	(40.4)
Traditionalist	89	(2.2)
Other	53	(1.3)
No Religion	454	(11.2)
**Parity**		
0	1,124	(27.7)
1	621	(15.3)
2	540	(13.3)
3	442	(10.9)
4	337	(8.3)
5	316	(7.8)
6+	678	(16.7)
**Marital Status**		
Single	1,335	(32.9)
Married	2,418	(59.6)
Formerly Married	304	(7.5)
**Residential Setting**		
Rural	1,972	(48.6)
Urban	2,085	(51.4)

Variable	Sample Size n (%)	
**Region**		
Centre	272	(6.7)
Centre-Est	110	(2.7)
Centre-Nord	329	(8.1)
Centre-Ouest	552	(13.6)
Nord	207	(5.1)
Nord-Est	166	(4.1)
Nord-Ouest	166	(4.1)
Ouest	475	(11.7)
Sud sans Abidjan	527	(13.0)
Sud-Ouest	312	(7.7)
Ville d'Abidjan	941	(23.2)
**Level of Education**		
No Education	2,170	(53.5)
Primary	1,018	(25.1)
Secondary	771	(19.0)
Higher	97	(2.4)
**Wealth Quintile**		
Poorest	690	(17.0)
Poorer	730	(18.0)
Middle	779	(19.2)
Richer	856	(21.1)
Richest	1,002	(24.7)
**Occupation**		
Not Working	1,160	(28.6)
Nonmanual	1,740	(42.9)
Manual	231	(5.7)
Agricultural	925	(22.8)
**Media Exposure**		
Not Exposed	1,091	(26.9)
Exposed to one media source	1,245	(30.7)
Exposed to two media sources	1,116	(27.5)
Exposed to three media sources	604	(14.9)
**Hormonal Contraceptives use**		
No	3,684	(90.8)
Yes	373	(9.2)

Table S21: Descriptive statistics for the study population in Rwanda.

Variable	Sample Size n (%)
**Sample Size**	5,995 (100)
**Nutritional status**	
Underweight	402 (6.7)
Normal Weight	4,358 (72.7)
Overweight/Obesity	1,235 (20.6)
**Age**	
15-19	1,349 (22.5)
20-24	1,061 (17.7)
25-29	977 (16.3)
30-34	863 (14.4)
35-39	695 (11.6)
40-44	570 (9.5)
45-49	480 (8.0)
**Religion**	
Catholic	2,410 (40.2)
Other Christians	3,447 (57.5)
Islam	120 (2.0)
No Religion	18 (0.3)
**Parity**	
0	2,206 (36.8)
1	791 (13.2)
2	743 (12.4)
3	594 (9.9)
4	498 (8.3)
5	426 (7.1)
6+	737 (12.3)
**Marital Status**	
Single	2,482 (41.4)
Married	2,848 (47.5)
Formerly Married	665 (11.1)
**Residential Setting**	
Rural	4,802 (80.1)
Urban	1,193 (19.9)
**Region**	
Kigali city	797 (13.3)
South	1,445 (24.1)
West	1.307 (21.8)
North	1,001 (16.7)
East	1,445 (24.1)
**Level of Education**	
No Education	719 (12.0)
Primary	3,855 (64.3)
Secondary	1,271 (21.2)
Higher	150 (2.5)
**Wealth Quintile**	
Poorest	1,169 (19.5)
Poorer	1,181 (19.7)
Middle	1,127 (18.8)
Richer	1,115 (18.6)
Richest	1,403 (23.4)

Variable	Sample Size n (%)	
**Occupation**		
Not Working	917	(15.3)
Nonmanual	971	(16.2)
Manual	234	(3.9)
Agricultural	3,873	(64.6)
**Media Exposure**		
Not Exposed	833	(13.9)
Exposed to one media source	2,236	(37.3)
Exposed to two media sources	1,834	(30.6)
Exposed to three media sources	1,091	(18.2)
**Hormonal Contraceptives use**		
No	4,448	(74.2)
Yes	1,547	(25.8)

Table S22: Descriptive statistics for the study population in Liberia.

Variable	Sample Size n (%)
**Sample Size**	3,998 (100)
**Nutritional status**	
Underweight	300 (7.5)
Normal Weight	2,639 (66.0)
Overweight/Obesity	1,059 (26.5)
**Age**	
15-19	864 (21.6)
20-24	720 (18.0)
25-29	676 (16.9)
30-34	496 (12.4)
35-39	508 (12.7)
40-44	396 (9.9)
45-49	340 (8.5)
**Ethnicity**	
Bassa	452 (11.3)
Gio	276 (6.9)
Gola	148 (3.7)
Grebo	332 (8.3)
Kissi	136 (3.4)
Kpelle	1,003 (25.1)
Krahn	116 (2.9)
Kru	272 (6.8)
Lorma	188 (4.7)
Mano	320 (8.0)
Vai	196 (4.9)
Others	560 (14.0)
**Religion**	
Other Christians	3,454 (86.4)
Islam	448 (11.2)
No Religion	96 (2.4)
**Parity**	
0	884 (22.1)
1	684 (17.1)
2	584 (14.6)
3	440 (11.0)
4	352 (8.8)
5	304 (7.6)
6+	752 (18.8)
**Marital Status**	
Single	1,323 (33.1)
Married	2,231 (55.8)
Formerly Married	444 (11.1)
**Residential Setting**	
Rural	1,567 (39.2)
Urban	2,431 (60.8)
**Region**	
North Western	360 (9.0)
South Central	2,123 (53.1)
South Eastern A	204 (5.1)
South Eastern B	252 (6.3)
North Central	1,059 (26.5)

Variable	Sample Size n (%)	
**Level of Education**		
No Education	1,327	(33.2)
Primary	1,159	(29.0)
Secondary	1,383	(34.6)
Higher	128	(3.2)
**Wealth Quintile**		
Poorest	692	(17.3)
Poorer	716	(17.9)
Middle	704	(17.6)
Richer	956	(23.9)
Richest	932	(23.3)
**Occupation**		
Not Working	1,687	(42.2)
Nonmanual	1,211	(30.3)
Manual	104	(2.6)
Agricultural	940	(23.5)
Others	56	(1.4)
**Media Exposure**		
Not Exposed	872	(21.8)
Exposed to one media source	1,431	(35.8)
Exposed to two media sources	1,051	(26.3)
Exposed to three media sources	644	(16.1)
**Hormonal Contraceptives use**		
No	3,142	(78.6)
Yes	856	(21.4)

Table S23: Descriptive statistics for the study population in Benin.

Variable	Sample Size n (%)
**Sample Size**	14,060 (100)
**Nutritional status**	
Underweight	886 (6.3)
Normal Weight	9,392 (66.8)
Overweight/Obesity	3,782 (26.9)
**Age**	
15-19	2,643 (18.8)
20-24	2,278 (16.2)
25-29	2,503 (17.8)
30-34	2,179 (15.5)
35-39	1,884 (13.4)
40-44	1,504 (10.7)
45-49	1,069 (7.6)
**Ethnicity**	
Adja	2,207 (15.7)
Bariba	1,195 (8.5)
Dendi	436 (3.1)
Fon	6,468 (46.0)
Yoa	408 (2.9)
Betamaribe	858 (6.1)
Peulh	436 (3.1)
Yoruba	1,772 (12.6)
Other Béninois	56 (0.4)
Other Nationalities	225 (1.6)
**Religion**	
Catholic	4,710 (33.5)
Other Christians	3,529 (25.1)
Islam	3,093 (22.0)
Traditionalists	1,772 (12.6)
Other	267 (1.9)
No Religion	689 (4.9)
**Parity**	
0	3,726 (26.5)
1	1,715 (12.2)
2	1,870 (13.3)
3	1,772 (12.6)
4	1,603 (11.4)
5	1,195 (8.5)
6+	2,179 (15.5)
**Marital Status**	
Single	3,698 (26.3)
Married	9,491 (67.5)
Formerly Married	872 (6.2)
**Residential Setting**	
Rural	7,466 (53.1)
Urban	6,594 (46.9)

Variable	Sample Size n (%)
**Region**	
Alibori	703 (5.0)
Atacora	1,153 (8.2)
Atlantique	1,758 (12.5)
Borgou	1,012 (7.2)
Collines	942 (6.7)
Couffo	970 (6.9)
Donga	576 (4.1)
Littoral	2,221 (15.8)
Mono	787 (5.6)
Ouémé	1.687 (12.0)
Plateau	984 (7.0)
Zou	1,265 (9.0)
**Level of Education**	
No Education	8,211 (58.4)
Primary	2,475 (17.6)
Secondary	3,107 (22.1)
Higher	267 (1.9)
**Wealth Quintile**	
Poorest	2,362 (16.8)
Poorer	2,531 (18.0)
Middle	2,615 (18.6)
Richer	3,051 (21.7)
Richest	3,501 (24.9)
**Occupation**	
Not Working	5,329 (37.9)
Nonmanual	5,202 (37.0)
Manual	830 (5.9)
Agricultural	1,997 (14.2)
Others	703 (5.0)
**Media Exposure**	
Not Exposed	4,063 (28.9)
Exposed to one media source	3,698 (26.3)
Exposed to two media sources	4,499 (32.0)
Exposed to three media sources	1,800 (12.8)
**Hormonal Contraceptives use**	
No	13,455 (95.7)
Yes	605 (4.3)

Table S24: Descriptive statistics for the study population in Kenya.

Variable	Sample Size n (%)
**Total Sample Size**	12,967 (100)
**Nutritional status**	
Underweight	1,167 (9.0)
Normal Weight	7,547 (58.2)
Overweight/Obesity	4,253 (32.8)
**Age**	
15-19	2,451 (18.9)
20-24	2,230 (17.2)
25-29	2,516 (19.4)
30-34	1,893 (14.6)
35-39	1,647 (12.7)
40-44	1,219 (9.4)
45-49	1,011 (7.8)
**Ethnicity**	
Embu	130 (1.0)
Kalenjin	1,595 (12.3)
Kamba	1,504 (11.6)
Kikuyu	2,749 (21.2)
Kisii	791 (6.1)
Luhya	2,049 (15.8)
Luo	1,375 (10.6)
Maasai	246 (1.9)
Meru	765 (5.9)
Mijikenda/Swahili	674 (5.2)
Somali	272 (2.1)
Taita/Taveta	117 (0.9)
Turkana	156 (1.2)
Samburu	52 (0.4)
Other	493 (3.8)
**Religion**	
Catholic	2,593 (20.0)
Other Christians	9,362 (72.2)
Islam	765 (5.9)
Others	39 (0.3)
No Religion	207 (1.6)
**Parity**	
0	3,410 (26.3)
1	1,971 (15.2)
2	2,075 (16.0)
3	1,712 (13.2)
4	1,297 (10.0)
5	882 (6.8)
6+	1,621 (12.5)
**Marital Status**	
Single	3,955 (30.5)
Married	7,469 (57.6)
Formerly Married	1,543 (11.9)
**Residential Setting**	
Rural	7,780 (60.0)
Urban	5,187 (40.0)

Variable	Sample Size n (%)
**Region**	
Coast	1,245 (9.6)
North Eastern	233 (1.8)
Eastern	1,893 (14.6)
Central	1,673 (12.9)
Rift Valley	3,307 (25.5)
Western	1,413 (10.9)
Nyanza	1,699 (13.1)
Nairobi	1,504 (11.6)
**Level of Education**	
No Education	843 (6.5)
Primary	6,509 (50.2)
Secondary	4,227 (32.6)
Higher	1,387 (10.7)
**Wealth Quintile**	
Poorest	1,906 (14.7)
Poorer	2,308 (17.8)
Middle	2,580 (19.9)
Richer	2,775 (21.4)
Richest	3,397 (26.2)
**Occupation**	
Not Working	4,357 (33.6)
Nonmanual	4,940 (38.1)
Manual	934 (7.2)
Agricultural	2,736 (21.1)
**Media Exposure**	
Not Exposed	1,504 (11.6)
Exposed to one media source	3,786 (29.2)
Exposed to two media sources	4,240 (32.7)
Exposed to three media sources	3,436 (26.5)
**Hormonal Contraceptives use**	
No	8,454 (65.2)
Yes	4,513 (34.8)

Table S25: Descriptive statistics for the study population in Sao Tome and Principe.

Variable	Sample Size n (%)
**Sample Size**	2,078 (100)
**Nutritional status**	
Underweight	160 (7.7)
Normal Weight	1,226 (59.0)
Overweight/Obesity	692 (33.3)
**Age**	
15-19	453 (21.8)
20-24	347 (16.7)
25-29	330 (15.9)
30-34	310 (14.9)
35-39	204 (9.8)
40-44	247 (11.9)
45-49	187 (9.0)
**Religion**	
Catholic	1,459 (70.2)
Other Christians	301 (14.5)
Other	102 (4.9)
No Religion	216 (10.4)
**Parity**	
0	544 (26.2)
1	274 (13.2)
2	291 (14.0)
3	276 (13.3)
4	220 (10.6)
5	160 (7.7)
6+	312 (15.0)
**Marital Status**	
Single	524 (25.2)
Married	1,299 (62.5)
Formerly Married	256 (12.3)
**Region**	
Região Centro	1,216 (58.5)
Região Sul	305 (14.7)
Região Norte	449 (21.6)
Região do Príncipe	108 (5.2)
**Residential Setting**	
Rural	756 (36.4)
Urban	1,322 (63.6)
**Level of Education**	
No Education	125 (6.0)
Primary	1,189 (57.2)
Secondary	765 (36.8)
**Wealth Quintile**	
Poorest	362 (17.4)
Poorer	387 (18.6)
Middle	349 (16.8)
Richer	455 (21.9)
Richest	526 (25.3)
**Occupation**	
Not Working	860 (41.4)
Nonmanual	1,056 (50.8)
Manual	162 (7.8)

Variable	Sample Size n (%)	
**Media Exposure**		
Not Exposed	77	(3.7)
Exposed to one media source	87	(4.2)
Exposed to two media sources	794	(38.2)
Exposed to three media sources	1,120	(53.9)
**Hormonal Contraceptives use**		
No	1,644	(79.1)
Yes	434	(20.9)

Table S26: Descriptive statistics for the study population in Togo.

Variable	Sample Size n (%)
**Sample Size**	4,238 (100)
**Nutritional status**	
Underweight	297 (7.0)
Normal Weight	2,640 (62.3)
Overweight/Obesity	1,301 (30.7)
**Age**	
15-19	831 (19.6)
20-24	729 (17.2)
25-29	661 (15.6)
30-34	585 (13.8)
35-39	576 (13.6)
40-44	470 (11.1)
45-49	386 (9.1)
**Ethnicity**	
Adja-Ewé/Mina	1,784 (42.1)
Kabye/Tem	1,110 (26.2)
Akposso/Akebou	174 (4.1)
Ana-Ife	123 (2.9)
Para-Gourma/Akan	737 (17 .4)
Other Togolese	68 (1.6)
Stranger	242 (5.7)
**Religion**	
Other Christians	2,623 (61.9)
Islam	716 (16.9)
Traditionalist	602 (14.2)
No Religion	297 (7.0)
**Parity**	
0	1,259 (29.7)
1	615 (14.5)
2	538 (12.7)
3	487 (11.5)
4	407 (9.6)
5	301 (7.1)
6+	631 (14.9)
**Marital Status**	
Single	1,263 (29.8)
Married	2,649 (62.5)
Formerly Married	326 (7.7)
**Residential Setting**	
Rural	2,263 (53.4)
Urban	1,975 (46.6)
**Region**	
Grande Agglomération de Lomé	1,322 (31.2)
Maritime (Sans Agglomération de Lomé)	661 (15.6)
Plateaux	
Centrale	915 (21.6)
Kara	403 (9.5)
Savenes	470 (11.1)
	466 (11.0)

Variable	Sample Size n (%)	
**Level of Education**		
No Education	1,280	(30.2)
Primary	1,441	(34.0)
Secondary	1373	(32.4)
Higher	144	(3.4)
**Wealth Quintile**		
Poorest	708	(16.7)
Poorer	665	(15.7)
Middle	767	(18.1)
Richer	1,004	(23.7)
Richest	1,093	(25.8)
**Occupation**		
Not Working	1,144	(27.0)
Nonmanual	1,831	(43.2)
Manual	483	(11.4)
Agricultural	780	(18.4)
**Media Exposure**		
Not Exposed	1,144	(27.0)
Exposed to one media source	1,301	(30.7)
Exposed to two media sources	1,322	(31.2)
Exposed to three media sources	470	(11.1)
**Hormonal Contraceptives use**		
No	3,746	(88.4)
Yes	492	(11.6)

Table S27: Descriptive statistics for the study population in Cameroon.

Variable	Sample Size n (%)	
**Sample Size**	6,813	(100)
**Nutritional status**		
Underweight	470	(6.9)
Normal Weight	4,156	(61.0)
Overweight/Obesity	2,187	(32.1)
**Age**		
15-19	1,669	(24.5)
20-24	1,308	(19.2)
25-29	1,145	(16.8)
30-34	804	(11.8)
35-39	736	(10.8)
40-44	572	(8.4)
45-49	579	(8.5)
**Ethnicity**		
Arabes-Choa/Peulh/Haoussa/Kanuri	579	(8.5)
Biu-Mandara		
Adamaoua-Oubangui	899	(13.2)
Bantoïde Sud-Ouest	620	(9.1)
Grassfields	89	(1.3)
Bamilike/Bamoun	1,022	(15.0)
Côtier/Ngoe/Oroko	1,703	(25.0)
Beti/Bassa/Mbam	293	(4.3)
Kako/Meka/Pygmé	1,254	(18.4)
Stranger/Others	191	(2.8)
	164	(2.4)
**Religion**		
Catholic	2,541	(37.3)
Other Christians	2,569	(37.7)
Islam	1,301	(19.1)
Traditionalist	184	(2.7)
Others	82	(1.2)
No Religion	136	(2.0)
**Parity**		
0	2,119	(31.1)
1	940	(13.8)
2	838	(12.3)
3	681	(10.0)
4	559	(8.2)
5	477	(7.0)
6+	1,199	(17.6)
**Marital Status**		
Single	2,092	(30.7)
Married	4,095	(60.1)
Formerly Married	627	(9.2)
**Residential Setting**		
Rural	3,025	(44.4)
Urban	3,788	(55.6)

Variable	Sample Size n (%)	
**Region**		
Adamaoua	334	(4.9)
Centre (sans Yaoundé)	463	(6.8)
Douala	824	(12.1)
Est	266	(3.9)
Extrême-Nord	1,036	(15.2)
Littoral (sans Douala)	286	(4.2)
Nord	640	(9.4)
Nord-Ouest	722	(10.6)
Ouest	749	(11.0)
Sud	184	(2.7)
Sud-Ouest	518	(7.6)
Yaoundé	790	(11.6)
**Level of Education**		
No Education	1,213	(17.8)
Primary	2,330	(34.2)
Secondary	2,868	(42.1)
Higher	402	(5.9)
**Wealth Quintile**		
Poorest	1,015	(14.9)
Poorer	1,158	(17.0)
Middle	1,274	(18.7)
Richer	1,642	(24.1)
Richest	1,724	(25.3)
**Occupation**		
Not Working	2,112	(31.0)
Nonmanual	2,119	(31.1)
Manual	831	(12.2)
Agricultural	1,751	(25.7)
**Media Exposure**		
Not Exposed	1,676	(24.6)
Exposed to one media source	1,587	(23.3)
Exposed to two media sources	2,044	(30.0)
Exposed to three media sources	1,506	(22.1)
**Hormonal Contraceptives use**		
No	6,459	(94.8)
Yes	354	(5.2)

Table S28: Descriptive statistics for study population in Comoros.

Variable	Sample Size n (%)	
**Total Sample Size**	4,479	(100)
**Nutritional status**		
Underweight	318	(7.1)
Normal Weight	2,562	(57.2)
Overweight/Obesity	1,599	(35.7)
**Age**		
15-19	1,178	(26.3)
20-24	806	(18.0)
25-29	690	(15.4)
30-34	596	(13.3)
35-39	529	(11.8)
40-44	412	(9.2)
45-49	269	(6.0)
**Parity**		
0	2,145	(47.9)
1	394	(8.8)
2	385	(8.6)
3	376	(8.4)
4	331	(7.4)
5	237	(5.3)
6+	609	(13.6)
**Marital Status**		
Single	1,608	(35.9)
Married	2,558	(57.1)
Formerly Married	314	(7.0)
**Residential Setting**		
Rural	2,938	(65.6)
Urban	1,541	(34.4)
**Region**		
Ngazidja	2,159	(48.2)
Mwali	269	(6.0)
Ndzuwani	2,051	(45.8)
**Level of Education**		
No Education	1,380	(30.8)
Primary	833	(18.6)
Secondary	1,841	(41.1)
Higher	426	(9.5)
**Wealth Quintile**		
Poorest	708	(15.8)
Poorer	882	(19.7)
Middle	941	(21.0)
Richer	950	(21.2)
Richest	999	(22.3)
**Occupation**		
Not Working	2,737	(61.1)
Nonmanual	873	(19.5)
Manual	434	(9.7)
Agricultural	434	(9.7)
**Media Exposure**		
Not Exposed	815	(18.2)
Exposed to one media source	1,026	(22.9)
Exposed to two media sources	1,774	(39.6)
Exposed to three media sources	864	(19.3)

Variable	Sample Size n (%)	
**Hormonal Contraceptives use**		
No	4,143	(92.5)
Yes	336	(7.5)

Table S29: Descriptive statistics for the study population in Tanzania.

Variable	Sample Size n (%)	
**Sample Size**	11,535	(100)
**Nutritional status**		
Underweight	1,096	(9.5)
Normal Weight	7,152	(62.0)
Overweight/Obesity	3,287	(28.5)
**Age**		
15-19	2,549	(22.1)
20-24	2,042	(17.7)
25-29	1,742	(15.1)
30-34	1,488	(12.9)
35-39	1,442	(12.5)
40-44	1,292	(11.2)
45-49	980	(8.5)
**Parity**		
0	3,045	(26.4)
1	1,776	(15.4)
2	1,569	(13.6)
3	1,292	(11.2)
4	1,073	(9.3)
5	865	(7.5)
6+	1,915	(16.6)
**Marital Status**		
Single	3,161	(27.4)
Married	6,806	(59.0)
Formerly Married	1,569	(13.6)
**Residential Setting**		
Rural	7,256	(62.9)
Urban	4,279	(37.1)
**Level of Education**		
No Education	1,638	(14.2)
Primary	7,094	(61.5)
Secondary	2,642	(22.9)
Higher	161	(1.4)

Variable	Sample Size n (%)	
**Region**		
Dodoma	496	(4.3)
Arusha	450	(3.9)
Kilimanjaro	335	(2.9)
Tanga	623	(5.4)
Morogoro	577	(5.0)
Pwani	254	(2.2)
Dar es salaam	1,384	(12.0)
Lindi	265	(2.3)
Mtwara	381	(3.3)
Ruvuma	323	(2.8)
Iringa	219	(1.9)
Mbeya	715	(6.2)
Singida	311	(2.7)
Tabora	588	(5.1)
Rukwa	242	(2.1)
Kigoma	473	(4.1)
Shinyanga	415	(3.6)
Kagera	542	(4.7)
Mwanza	715	(6.2)
Mara	427	(3.7)
Manyara	335	(2.9)
Njombe	185	(1.6)
Katavi	104	(0.9)
Simiyu	415	(3.6)
geita	404	(3.5)
Kaskazini Unguja	46	(0.4)
Kusini Unguja	35	(0.3)
Mjini Magharibi	185	(1.6)
Kaskazini Pemba	46	(0.4)
Kusini Pemba	46	(0.4)
**Wealth Quintile**		
Poorest	1,823	(15.8)
Poorer	1,938	(16.8)
Middle	2,030	(17.6)
Richer	2,492	(21.6)
Richest	3,253	(28.2)
**Occupation**		
Not Working	2,665	(23.1)
Nonmanual	1,615	(14.0)
Manual	2,353	(20.4)
Agricultural	4,902	(42.5)
**Media Exposure**		
Not Exposed	1,661	(14.4)
Exposed to one media source	2,930	(25.4)
Exposed to two media sources	3,380	(29.3)
Exposed to three media sources	3,564	(30.9)
**Hormonal Contraceptives use**		
No	8,963	(77.7)
Yes	2,572	(22.3)

Table S30: Descriptive statistics for the study population in Zimbabwe.

Variable	Sample Size n (%)
**Sample Size**	8,721 (100)
**Nutritional status**	
Underweight	541 (6.2)
Normal Weight	5,145 (59.0)
Overweight/Obesity	3,035 (34.8)
**Age**	
15-19	1,980 (22.7)
20-24	1,404 (16.1)
25-29	1,395 (16.0)
30-34	1,378 (15.8)
35-39	1,108 (12.7)
40-44	916 (10.5)
45-49	541 (6.2)
**Religion**	
Catholic	602 (6.9)
Other Christians	7,622 (87.4)
Islam	26 (0.3)
Traditionalists	52 (0.6)
No Religion	419 (4.8)
**Parity**	
0	2,390 (27.4)
1	1,282 (14.7)
2	1,544 (17.7)
3	1,456 (16.7)
4	985 (11.3)
5	523 (6.0)
6+	541 (6.2)
**Marital Status**	
Single	2,381 (27.3)
Married	5,137 (58.9)
Formerly Married	1,203 (13.8)
**Residential Setting**	
Rural	5,381 (61.7)
Urban	3,340 (38.3)
**Region**	
Manicaland	1,099 (12.6)
Mashonaland Central	759 (8.7)
Mashonaland East	846 (9.7)
Mashonaland West	1,020 (11.7)
Matabeleland North	419 (4.8)
Matabeleland South	366 (4.2)
Midlands	1,099 (12.6)
Masvingo	1,047 (12.0)
Harare	1,552 (17.8)
Bulawayo	515 (5.9)
**Level of Education**	
No Education	105 (1.2)
Primary	2,250 (25.8)
Secondary	5,730 (65.7)
Higher	637 (7.3)

Variable	Sample Size n (%)	
**Wealth Quintile**		
Poorest	1,474	(16.9)
Poorer	1,474	(16.9)
Middle	1,570	(18.0)
Richer	1,980	(22.7)
Richest	2,224	(25.5)
**Occupation**		
Not Working	4,247	(48.7)
Nonmanual	3,305	(37.9)
Manual	253	(2.9)
Agricultural	820	(9.4)
Others	96	(1.1)
**Media Exposure**		
Not Exposed	2,058	(23.6)
Exposed to one media source	2,398	(27.5)
Exposed to two media sources	2,363	(27.1)
Exposed to three media sources	1,901	(21.8)
**Hormonal Contraceptives use**		
No	4,718	(54.1)
Yes	4,003	(45.9)

Table S31: Descriptive statistics for the study population in Gabon.

Variable	Sample Size n (%)	
**Total Sample Size**	4,213	(100)
**Nutritional status**		
Underweight	333	(7.9)
Normal Weight	2,157	(51.2)
Overweight/Obesity	1,723	(40.9)
**Age**		
15-19	948	(22.5)
20-24	750	(17.8)
25-29	653	(15.5)
30-34	594	(14.1)
35-39	510	(12.1)
40-44	417	(9.9)
45-49	341	(8.1)
**Ethnicity**		
Shira-Punu/Vili	994	(23.6)
Fang	1,222	(29.0)
Kota-Kele	286	(6.8)
Mbede-Teke	354	(8.4)
Myene	303	(7.2)
Nzabi-Duma	598	(14.2)
Okande-Tsogho	105	(2.5)
Pygmee	21	(0.5)
Other	329	(7.8)
**Religion**		
Catholic	1,786	(42.4)
Other Christians	2,132	(50.6)
Others	72	(1.7)
No Religion	223	(5.3)
**Parity**		
0	1192	(28.3)
1	809	(19.2)
2	632	(15.0)
3	447	(10.6)
4	362	(8.6)
5	206	(4.9)
6+	565	(13.4)
**Marital Status**		
Single	1,622	(38.5)
Married	2,052	(48.7)
Formerly Married	539	(12.8)
**Residential Setting**		
Rural	543	(12.9)
Urban	3,670	(87.1)

Variable	Sample Size n (%)	
**Region**		
Estuaire	472	(11.2)
Libreville-Port-Gentil	2,313	(54.9)
Haut-Ogooué	409	(9.7)
Moyen-Ogooué	135	(3.2)
Ngounié	265	(6.3)
Nyanga	105	(2.5)
Ogooué Maritime	46	(1.1)
Ogooué-Ivindo	139	(3.3)
Ogooué-Lolo	143	(3.4)
Woleu-N'tem	185	(4.4)
**Level of Education**		
No Education	55	(1.3)
Primary	944	(22.4)
Secondary	2,802	(66.5)
Higher	413	(9.8)
**Wealth Quintile**		
Poorest	674	(16.0)
Poorer	784	(18.6)
Middle	923	(21.9)
Richer	817	(19.4)
Richest	1,015	(24.1)
**Occupation**		
Not Working	2,212	(52.5)
Nonmanual	1,584	(37.6)
Manual	185	(4.4)
Agricultural	232	(5.5)
**Media Exposure**		
Not Exposed	177	(4.2)
Exposed to one media source	881	(20.9)
Exposed to two media sources	1,496	(35.5)
Exposed to three media sources	1,660	(39.4)
**Hormonal Contraceptives use**		
No	4,036	(95.8)
Yes	177	(4.2)

Table S32: Descriptive statistics for the study population in Ghana.

Variable	Sample Size n (%)	
**Total Sample Size**	4,238	(100)
**Nutritional status**		
Underweight	263	(6.2)
Normal Weight	2,276	(53.7)
Overweight/Obesity	1,699	(40.1)
**Age**		
15-19	780	(18.4)
20-24	699	(16.5)
25-29	674	(15.9)
30-34	602	(14.2)
35-39	576	(13.6)
40-44	492	(11.6)
45-49	415	(9.8)
**Ethnicity**		
Akan	2,170	(51.2)
Ga/Dangme	335	(7.9)
Ewe	559	(13.2)
Guan	106	(2.5)
Mole-Dagbani	593	(14.0)
Grusi	119	(2.8)
Gurma	242	(5.7)
Mande	34	(0.8)
Others	81	(1.9)
**Religion**		
Catholics	445	(10.5)
Other Christians	2,971	(70.1)
Islam	636	(15.0)
Traditionalist	89	(2.1)
No Religion	97	(2.3)
**Parity**		
0	1,382	(32.6)
1	555	(13.1)
2	559	(13.2)
3	504	(11.9)
4	411	(9.7)
5	314	(7.4)
6+	513	(12.1)
**Marital Status**		
Single	1,458	(34.4)
Married	2,293	(54.1)
Formerly Married	487	(11.5)
**Residential Setting**		
Rural	1907	(45.0)
Urban	2331	(55.0)

Variable	Sample Size n (%)	
**Region**		
Western	500	(11.8)
Central	428	(10.1)
Greater Accra	877	(20.7)
Volta	322	(7.6)
Eastern	373	(8.8)
Ashanti	767	(18.1)
Brong Ahafo	348	(8.2)
Northern	364	(8.6)
Upper East	161	(3.8)
Upper West	97	(2.3)
**Level of Education**		
No Education	797	(18.8)
Primary	771	(18.2)
Secondary	2,420	(57.1)
Higher	250	(5.9)
**Wealth Quintile**		
Poorest	699	(16.5)
Poorer	720	(17.0)
Middle	882	(20.8)
Richer	954	(22.5)
Richest	983	(23.2)
**Occupation**		
Not Working	987	(23.3)
Nonmanual	1,924	(45.4)
Manual	542	(12.8)
Agricultural	784	(18.5)
**Media Exposure**		
Not Exposed	318	(7.5)
Exposed to one media source	949	(22.4)
Exposed to two media sources	2,272	(53.6)
Exposed to three media sources	699	(16.5)
**Hormonal Contraceptives use**		
No	3,585	(84.6)
Yes	653	(15.4)

Table S33: Descriptive statistics for study population in Lesotho.

Variable	Sample Size n (%)
**Total Sample Size**	3,157 (100)
**Nutritional status**	
Underweight	136 (4.3)
Normal Weight	1,620 (51.3)
Overweight/Obesity	1,402 (44.4)
**Age**	
15-19	695 (22.0)
20-24	603 (19.1)
25-29	515 (16.3)
30-34	455 (14.4)
35-39	344 (10.9)
40-44	290 (9.2)
45-49	256 (8.1)
**Religion**	
Catholic	1,250 (39.6)
Other Christians	1,856 (58.8)
Other	51 (1.6)
**Parity**	
0	957 (30.3)
1	691 (21.9)
2	606 (19.2)
3	388 (12.3)
4	230 (7.3)
5	139 (4.4)
6+	145 (4.6)
**Marital Status**	
Single	1,070 (33.9)
Married	1,667 (52.8)
Formerly Married	420 (13.3)
**Residential Setting**	
Rural	2,039 (64.6)
Urban	1,118 (35.4)
**Region**	
Maseru	859 (27.2)
Botha-Bothe	186 (5.9)
Leribe	502 (15.9)
Berea	420 (13.3)
Mafeteng	268 (8.5)
Mohale’s Hoek	268 (8.5)
Quthing	161 (5.1)
Qacha’s-Nek	95 (3.0)
Mokhotlong	167 (5.3)
Thaba Tseka	230 (7.3)
**Level of Education**	
No Education	35 (1.1)
Primary	1,241 (39.3)
Secondary	1,635 (51.8)
Higher	246 (7.8)

Variable	Sample Size n (%)	
**Wealth Quintile**		
Poorest	445	(14.1)
Poorer	524	(16.6)
Middle	597	(18.9)
Richer	758	(24.0)
Richest	833	(26.4)
**Media Exposure**		
Not Exposed	537	(17.0)
Exposed to one media source	1,017	(32.2)
Exposed to two media sources	947	(30.0)
Exposed to three media sources	657	(20.8)
**Hormonal Contraceptives use**		
No	2,254	(71.4)
Yes	903	(28.6)

Table S34: Descriptive statistics for the study population in Eswatini.

Variable	Sample Size n (%)
**Sample Size**	4,402 (100)
**Nutritional status**	
Underweight	141 (3.2)
Normal Weight	2,034 (46.2)
Overweight/Obesity	2,227 (50.6)
**Age**	
15-19	1,145 (26.0)
20-24	867 (19.7)
25-29	625 (14.2)
30-34	550 (12.5)
35-39	445 (10.1)
40-44	405 (9.2)
45-49	365 (8.3)
**Religion**	
Catholic	211 (4.8)
Other Christians	4,001 (90.9)
Traditionalists	18 (0.4)
No Religion	172 (3.9)
**Parity**	
0	1,351 (30.7)
1	814 (18.5)
2	643 (14.6)
3	475 (10.8)
4	321 (7.3)
5	242 (5.5)
6+	555 (12.6)
**Marital Status**	
Single	2,227 (50.6)
Married	1,770 (40.2)
Formerly Married	405 (9.2)
**Residential Setting**	
Rural	3,253 (73.9)
Urban	1,149 (26.1)
**Region**	
Hhohho	1,171 (26.6)
Manzini	1,461 (33.2)
Shiselweni	929 (21.1)
Lubombo	841 (19.1)
**Level of Education**	
No Education	352 (8.0)
Primary	1,431 (32.5)
Secondary	2,289 (52.0)
Higher	330 (7.5)
**Wealth Quintile**	
Poorest	687 (15.6)
Poorer	744 (16.9)
Middle	867 (19.7)
Richer	995 (22.6)
Richest	1,109 (25.2)

Variable	Sample Size n (%)	
**Occupation**		
Not Working	2,483	(56.4)
Nonmanual	1,448	(32.9)
Manual	299	(6.8)
Agricultural	172	(3.9)
**Media Exposure**		
Not Exposed	409	(9.3)
Exposed to one media source	1,052	(23.9)
Exposed to two media sources	1,576	(35.8)
Exposed to three media sources	1,365	(31.0)
**Hormonal Contraceptives use**		
No	3,561	(80.9)
Yes	841	(19.1)

Appendix 3: Random forest box plots for individual-level analyses (Figures S4 -S37).

1. The box plots present a summary of the individual level predictors for nutritional status i.e. both underweight (BMI <18.5 kg/m^2) and overweight/obesity (BMI ≥ 25.0 kg/m^2) among women of child-bearing age for each country.
2. Variables to the right of the dashed line (absolute value of the lowest ranking variable) are the most influential predictors for the individual's nutritional status, ranked based on the relative importance score.
3. Presented alongside the figure caption is the Out-of-Bag error (OOB Error), used as goodness of fit statistic for the model.

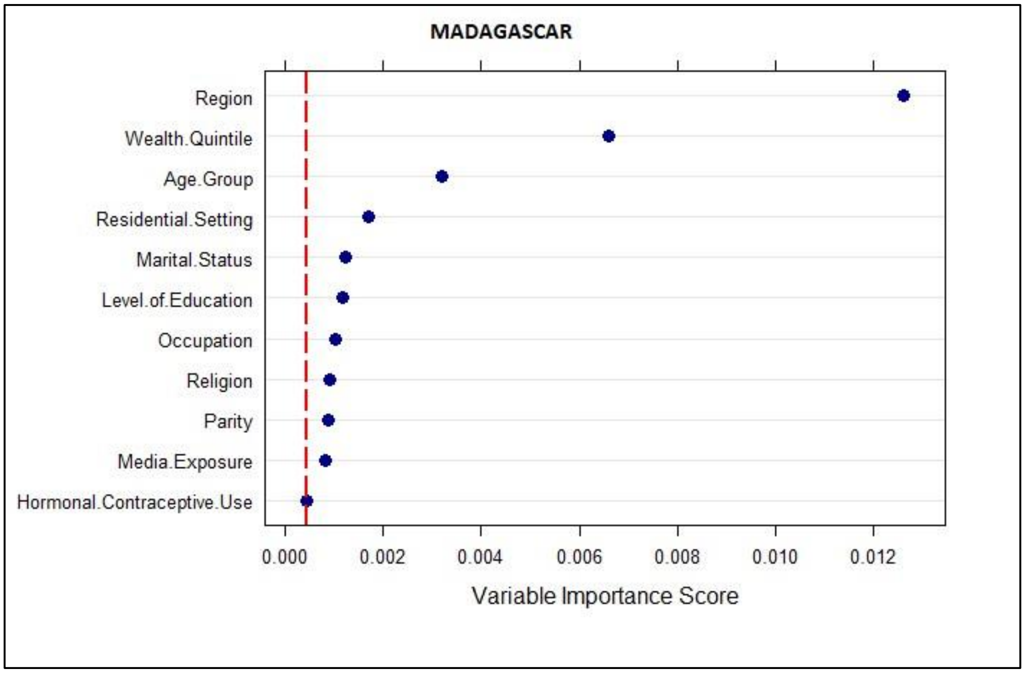

Figure S4: Predictors for nutritional status in Madagascar (OOB error = 29.0%).

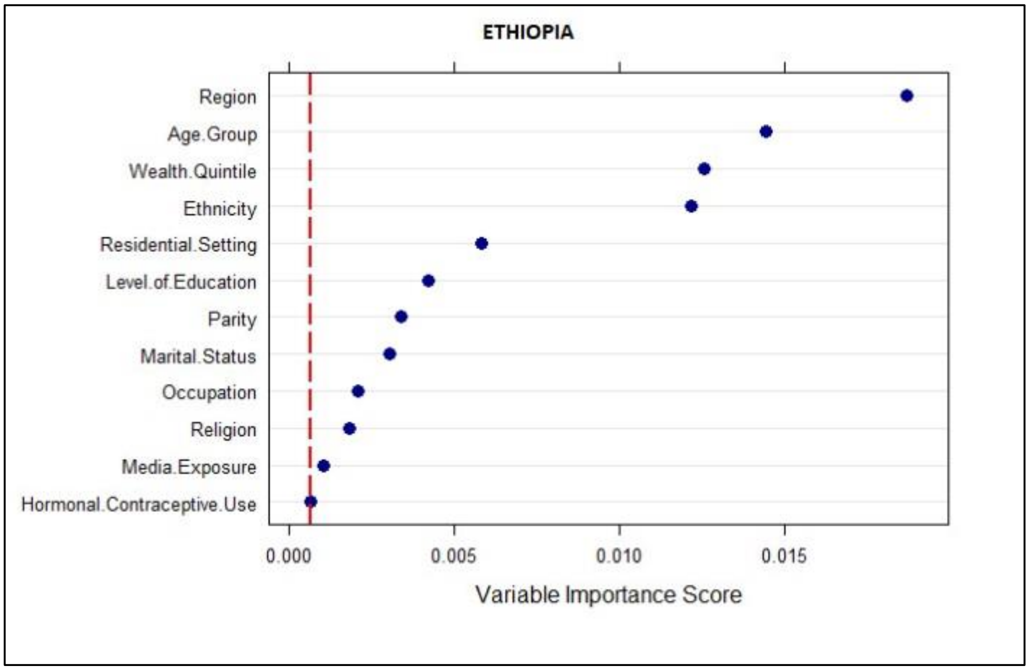

Figure S5: Predictors for nutritional status in Ethiopia (OOB error = 30.5%).

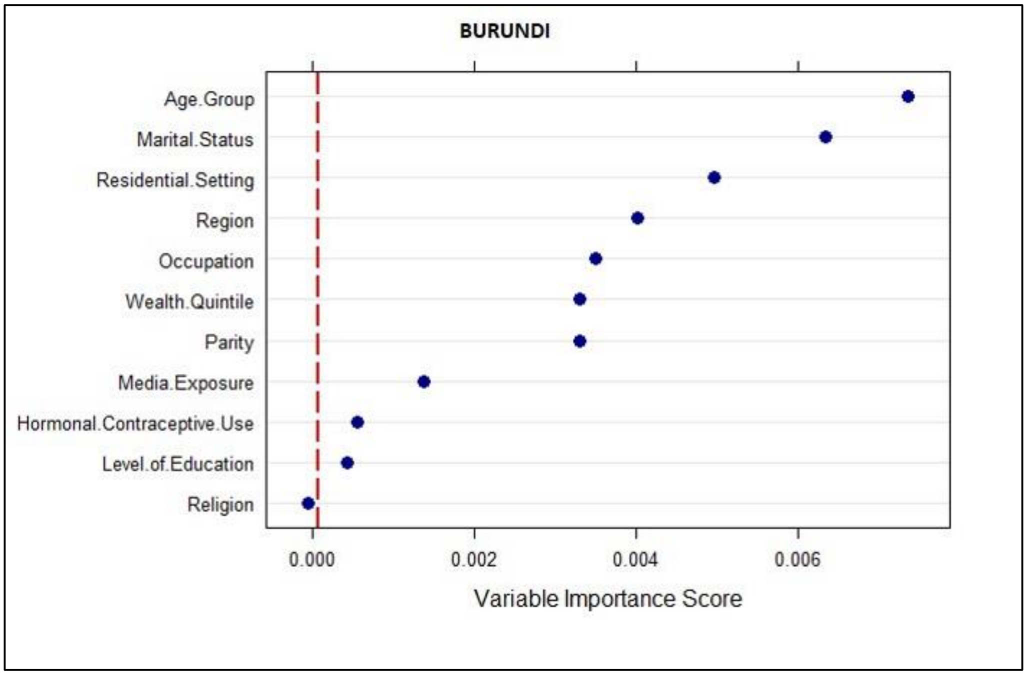

Figure S6: Predictors for nutritional status in Burundi (OOB error = 24.1%).

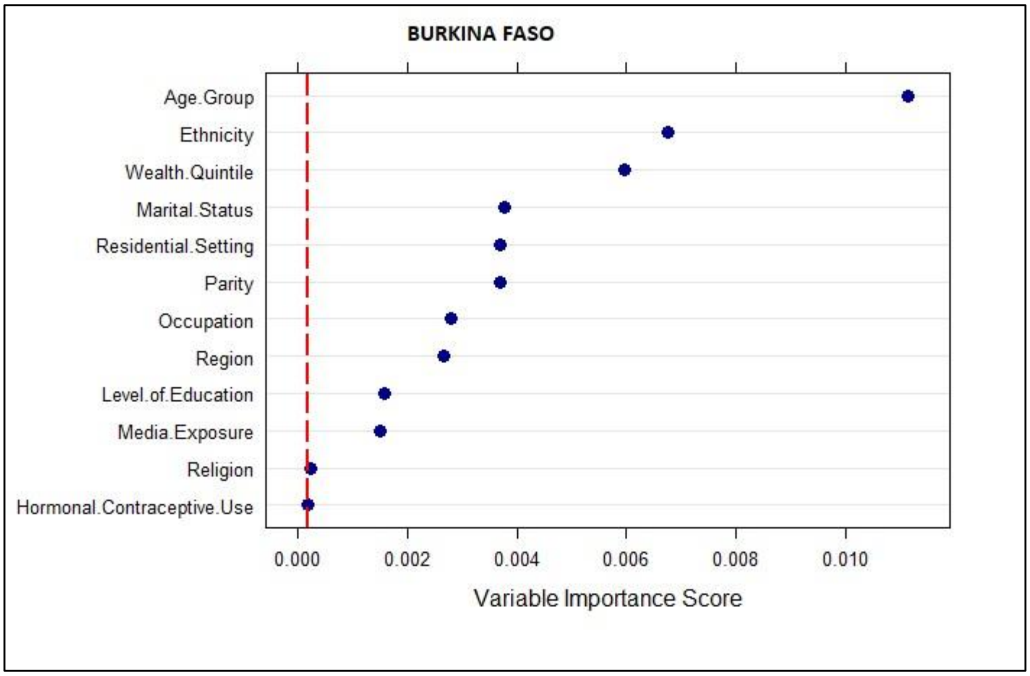

Figure S7: Predictors for nutritional status in Burkina Faso (OOB error = 22.8%).

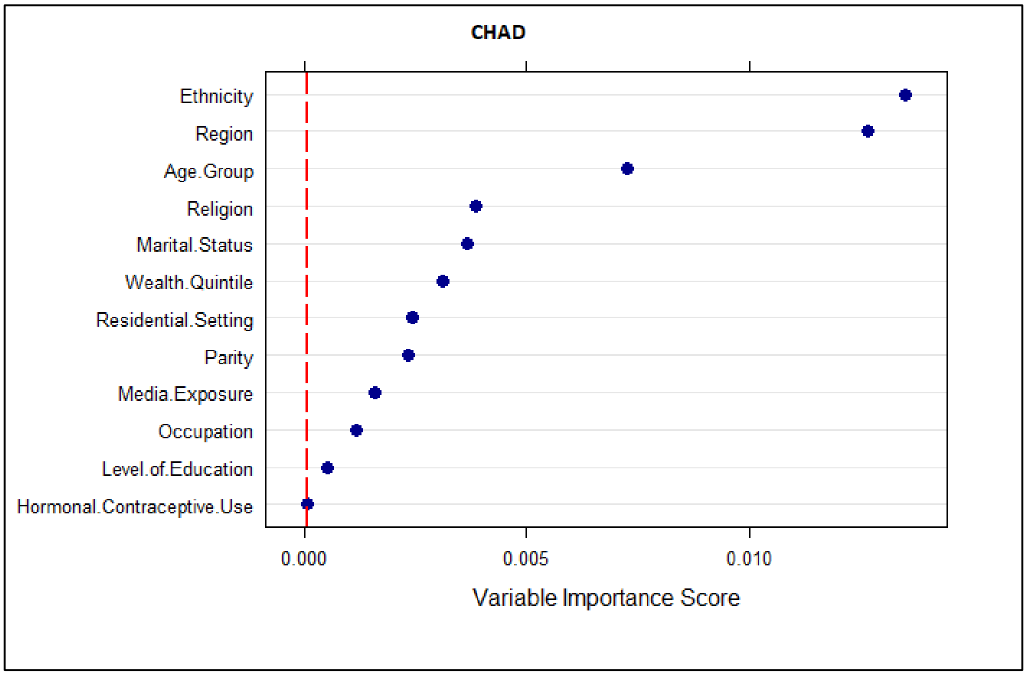

Figure S8: Predictors for nutritional status in Chad (OOB error = 27.4%).

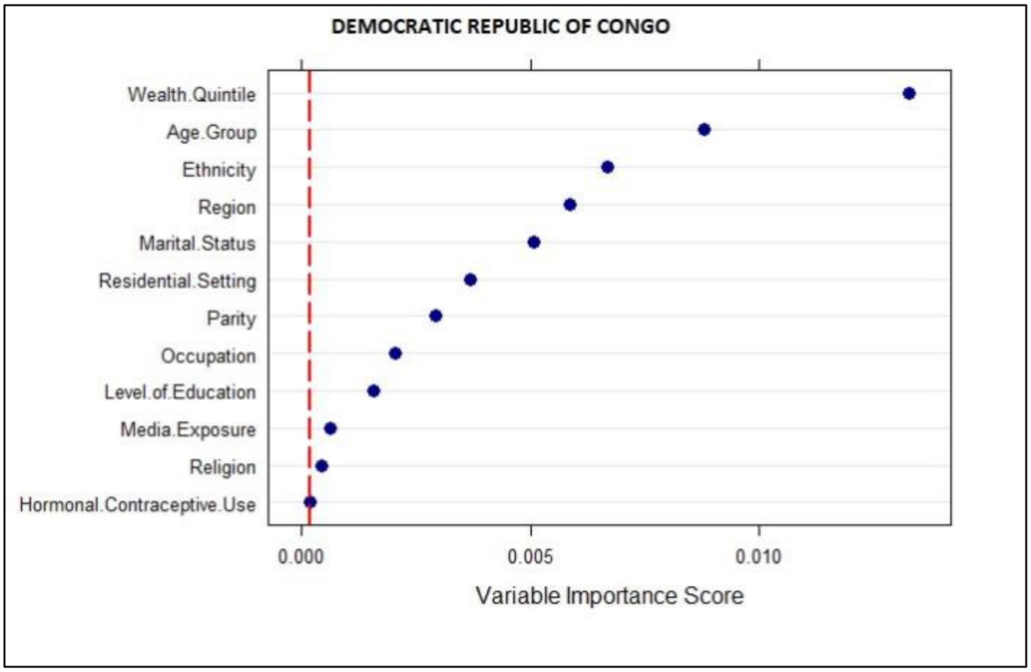

Figure S9: Predictors for nutritional status in D R Congo (OOB error = 29.0%).

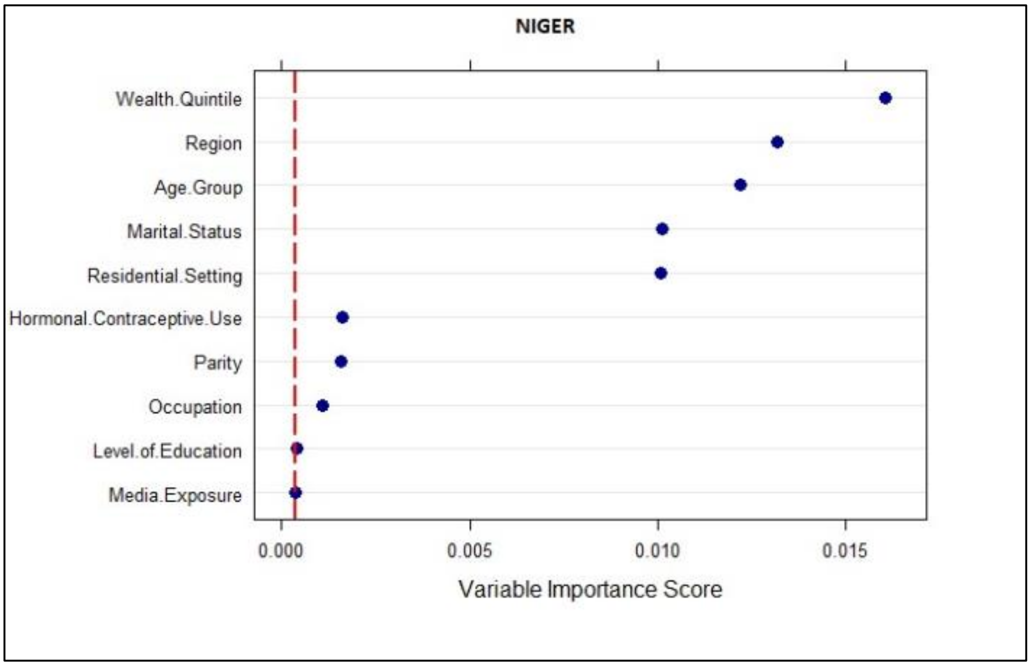

Figure S10: Predictors for nutritional status in Niger (OOB error = 29.3%).

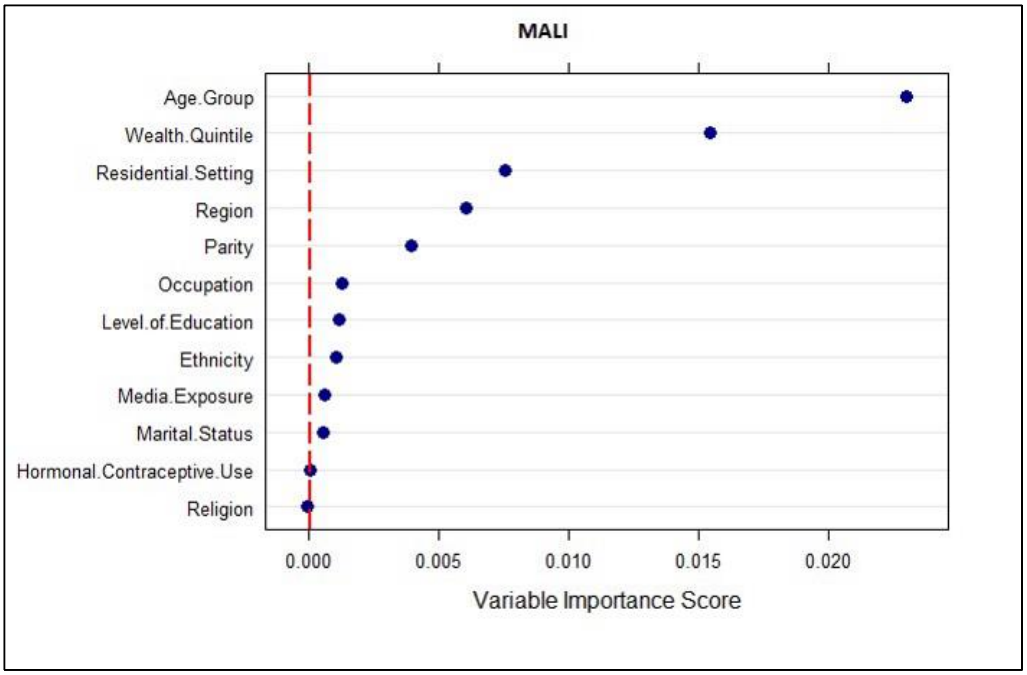

Figure S11: Predictors for nutritional status in Mali (OOB error = 26.4%).

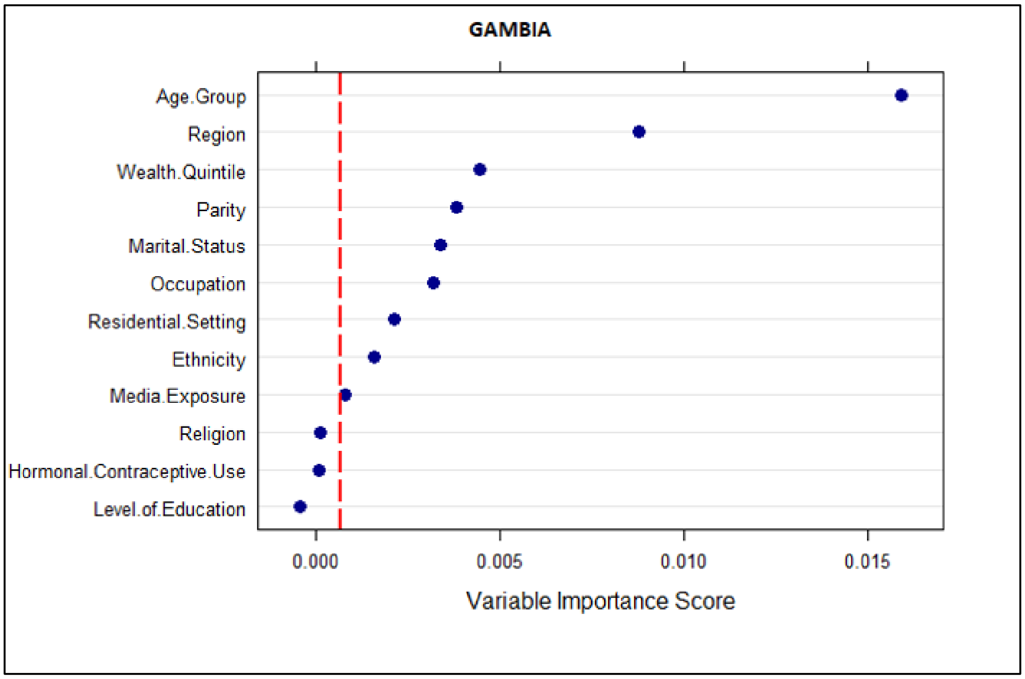

Figure S12: Predictors for nutritional status in Gambia (OOB error = 34.0%).

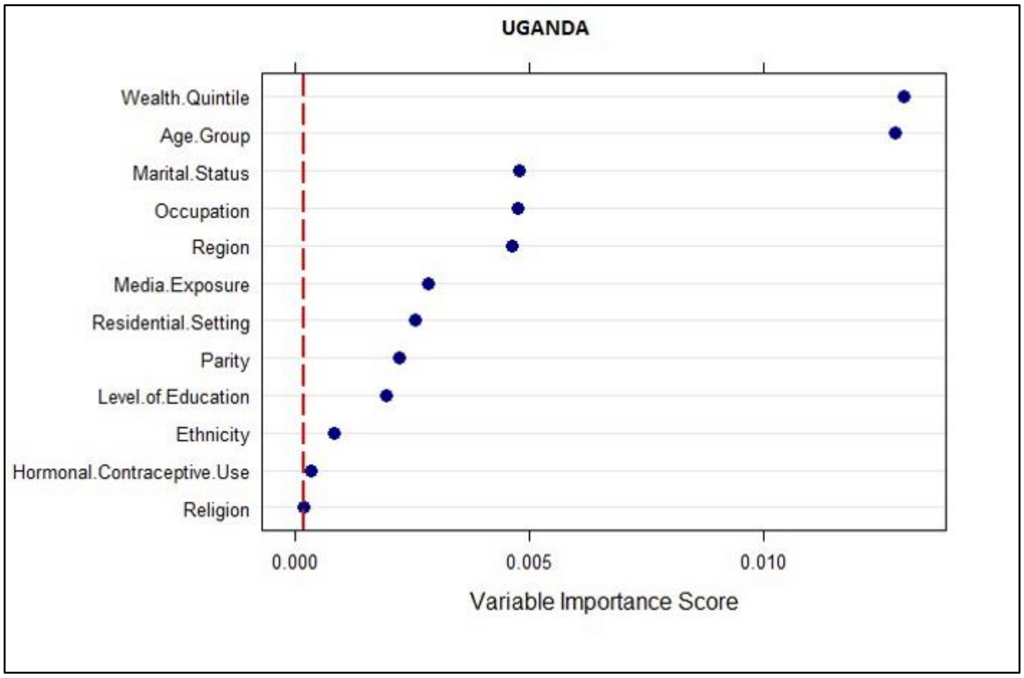

Figure S13: Predictors for nutritional status in Uganda (OOB error = 27.2%).

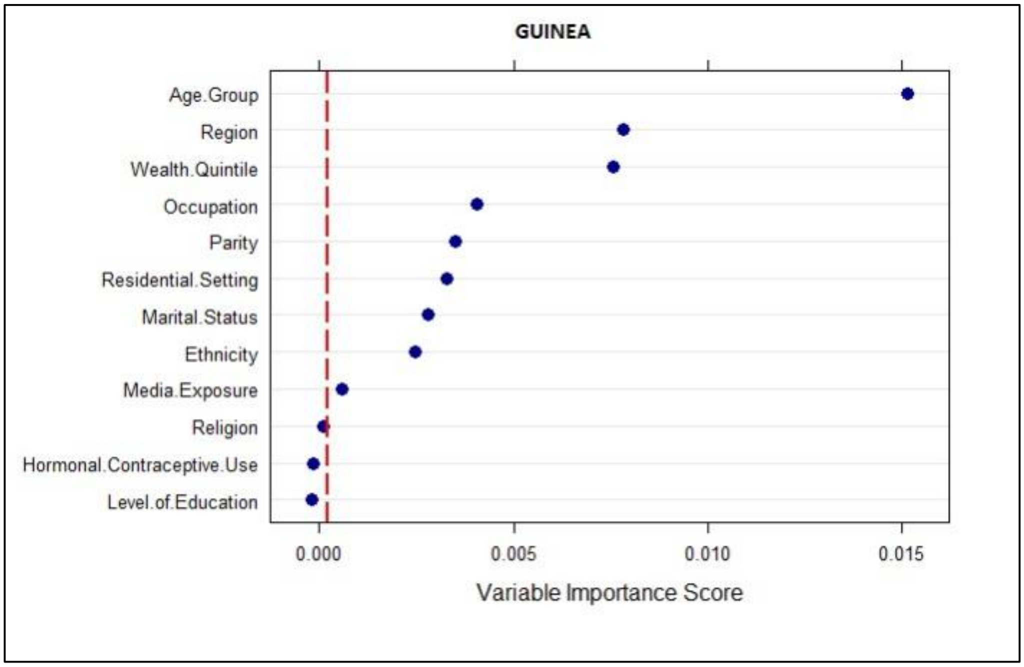

Figure S14: Predictors for nutritional status in Guinea (OOB error = 27.4%).

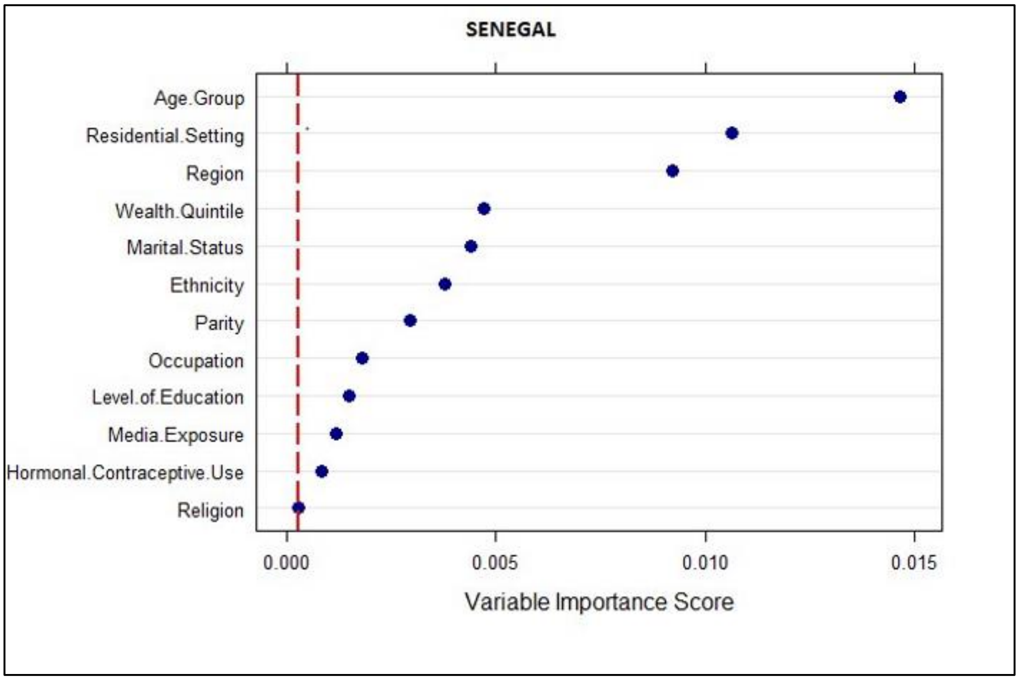

Figure S15: Predictors for nutritional status in Senegal (OOB error = 33.5%).

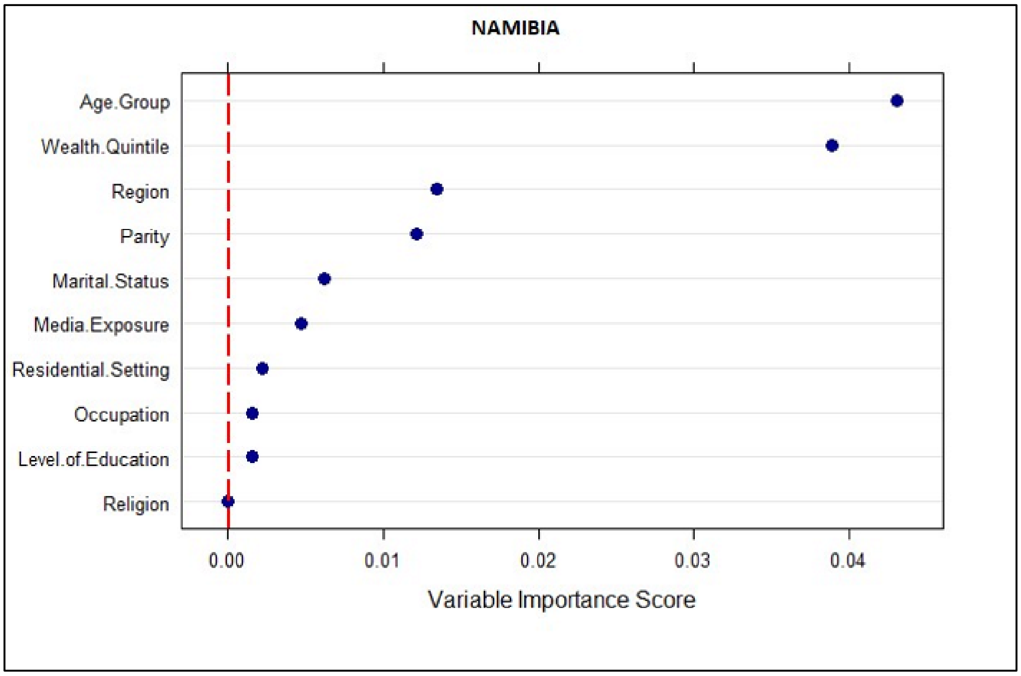

Figure S16: Predictors for nutritional status in Namibia (OOB error = 30.0%).

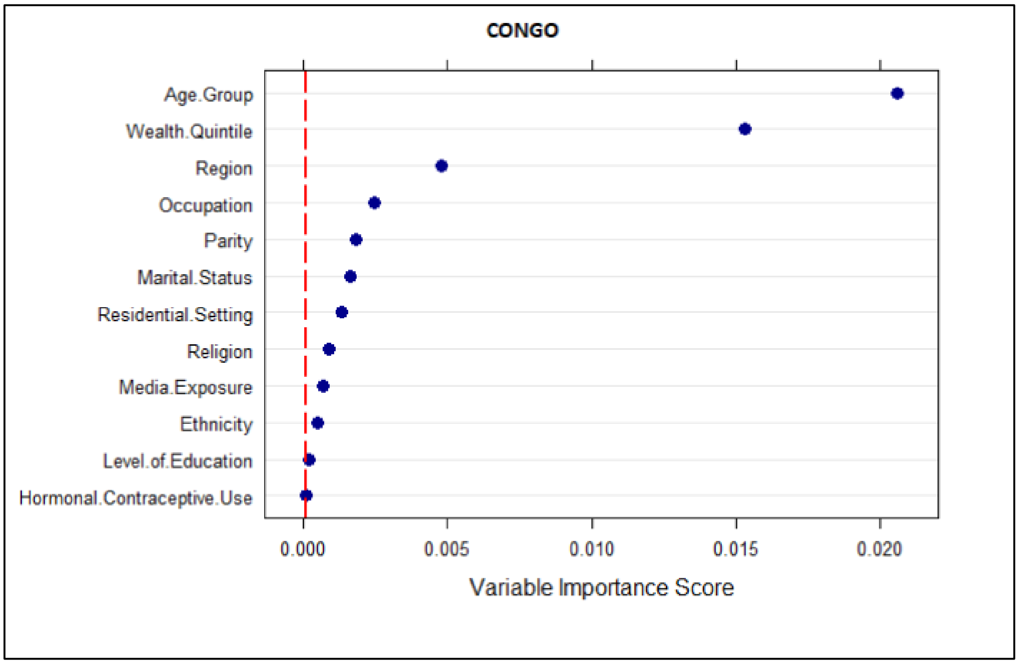

Figure S17: Predictors for nutritional status in Congo (OOB error = 28.6%).

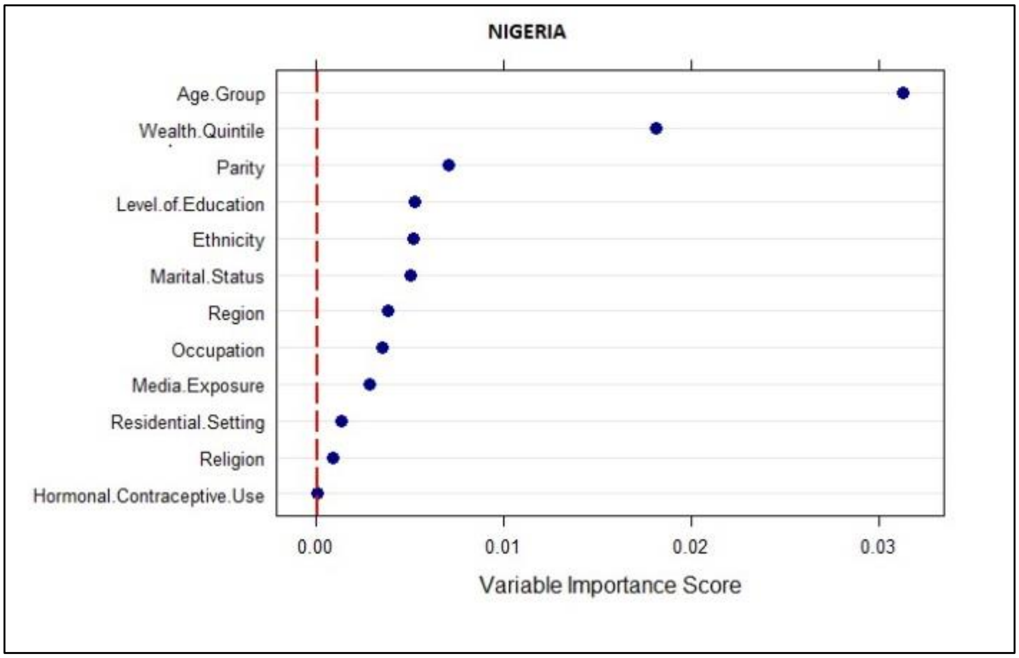

Figure S18: Predictors for nutritional status in Nigeria (OOB error = 27.7%).

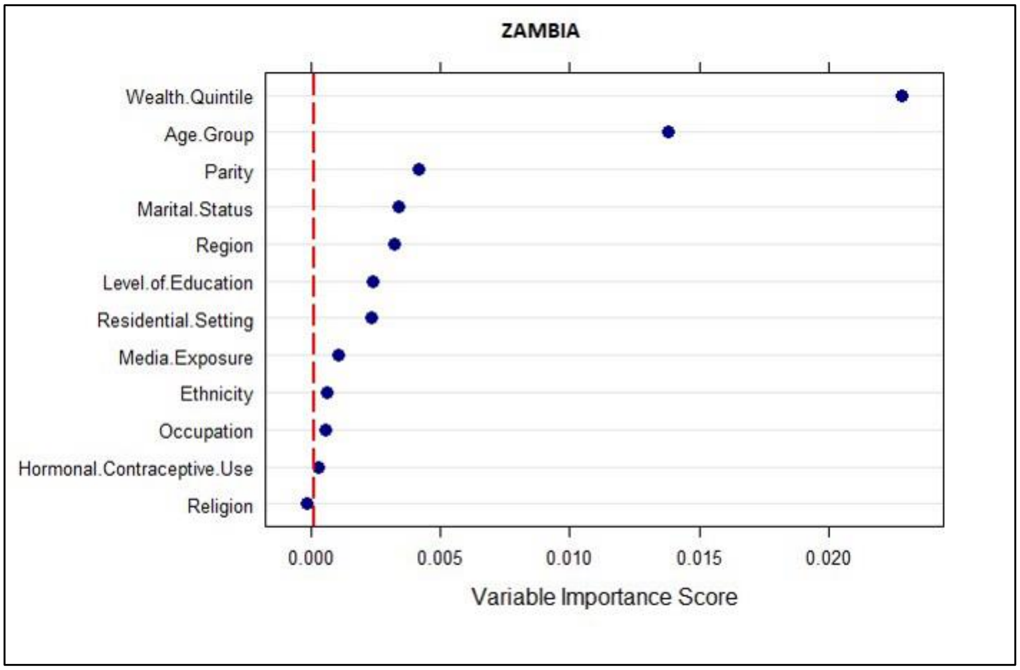

Figure S19: Predictors for nutritional status in Zambia (OOB error = 25.7%).

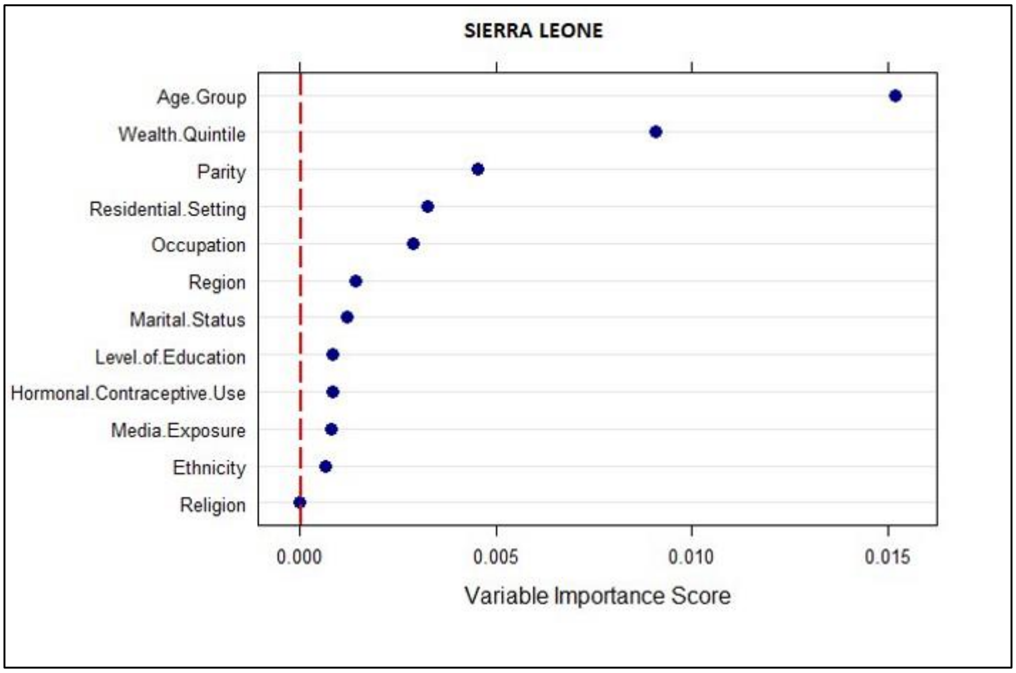

Figure S20: Predictors for nutritional status in Sierra Leone (OOB error = 24.9%).

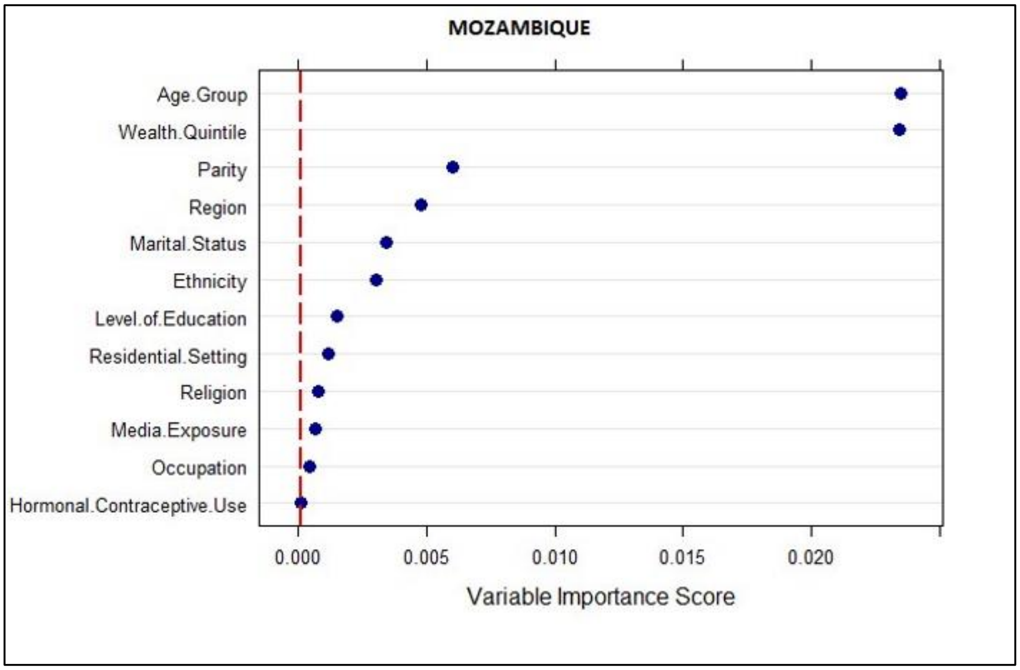

Figure S21: Predictors for nutritional status in Mozambique (OOB error = 22.7%).

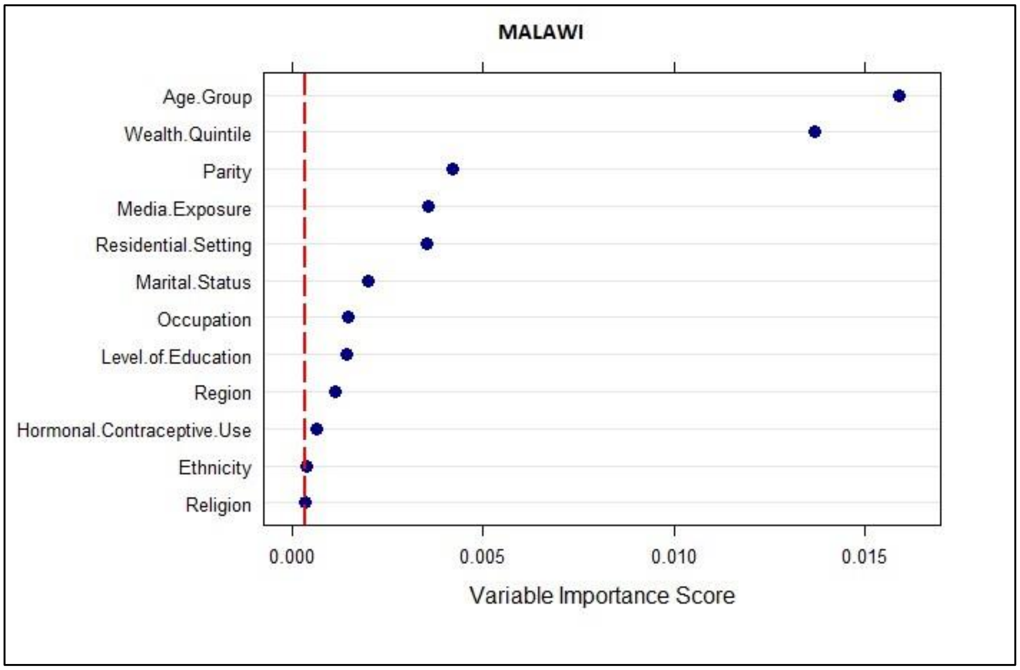

Figure S22: Predictors for nutritional status in Malawi (OOB error = 23.4%).

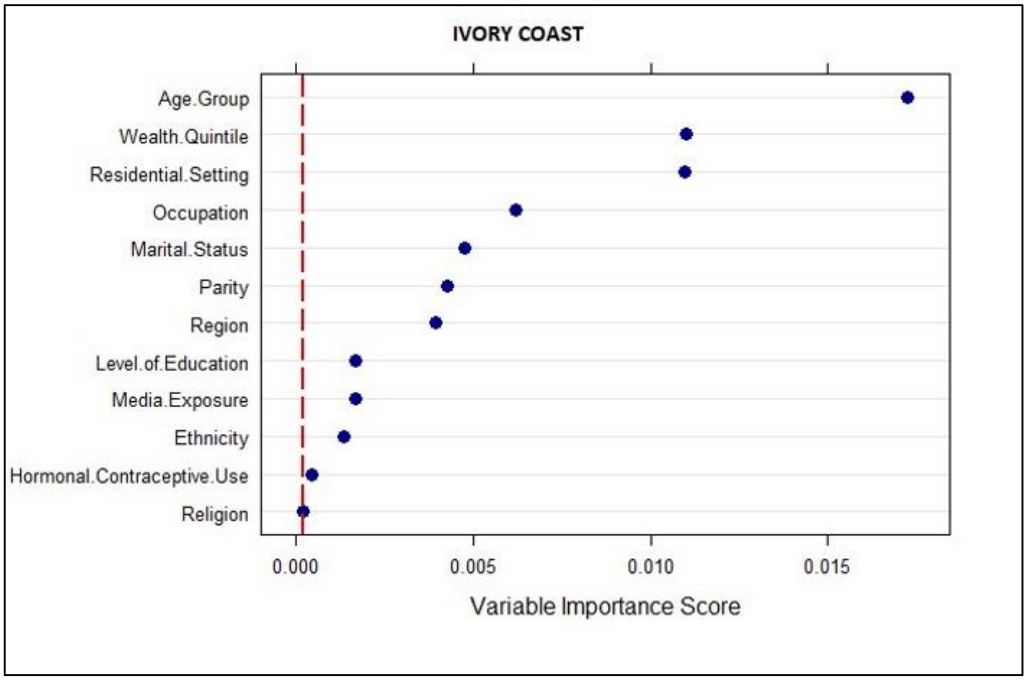

Figure S23: Predictors for nutritional status in Ivory Coast (OOB error = 24.5%).

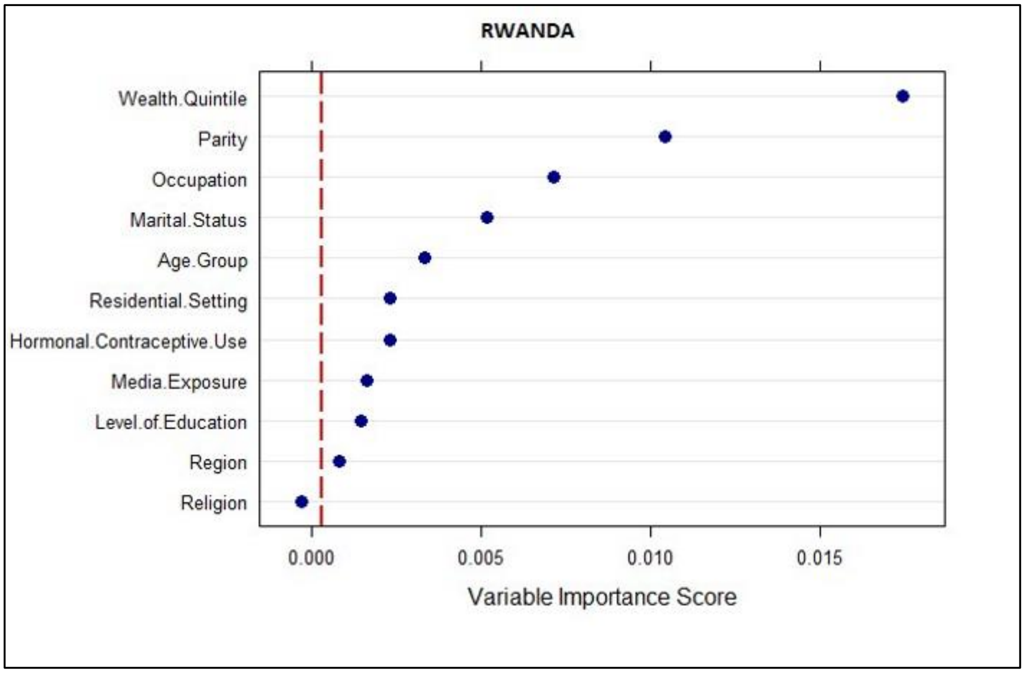

Figure S24: Predictors for nutritional status in Rwanda (OOB error = 24.2%).

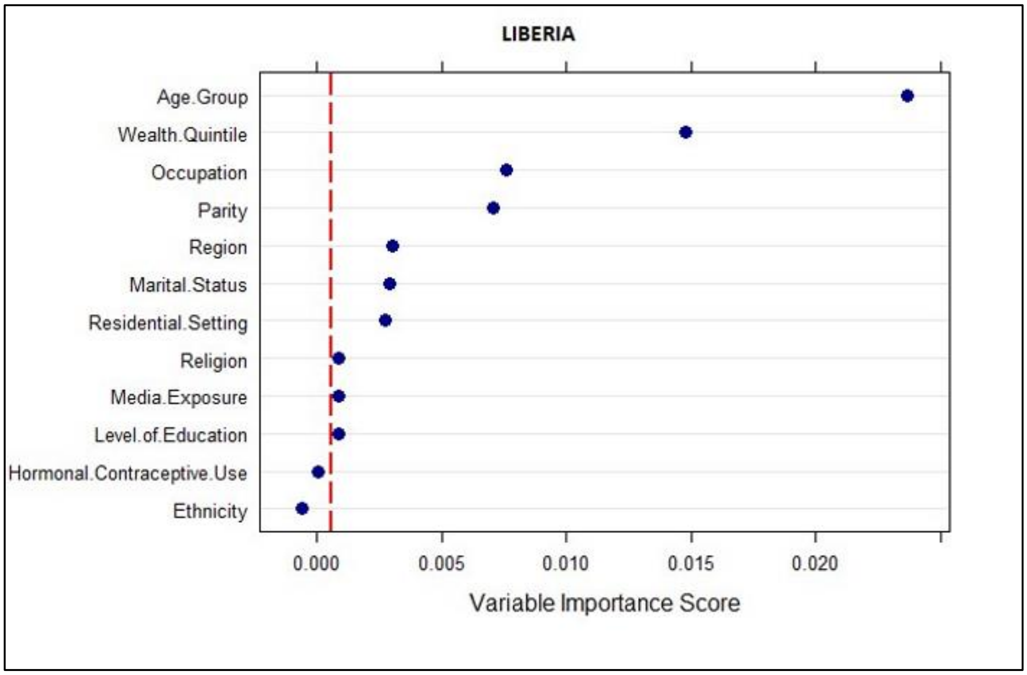

Figure S25: Predictors for nutritional status in Liberia (OOB error = 25.5%).

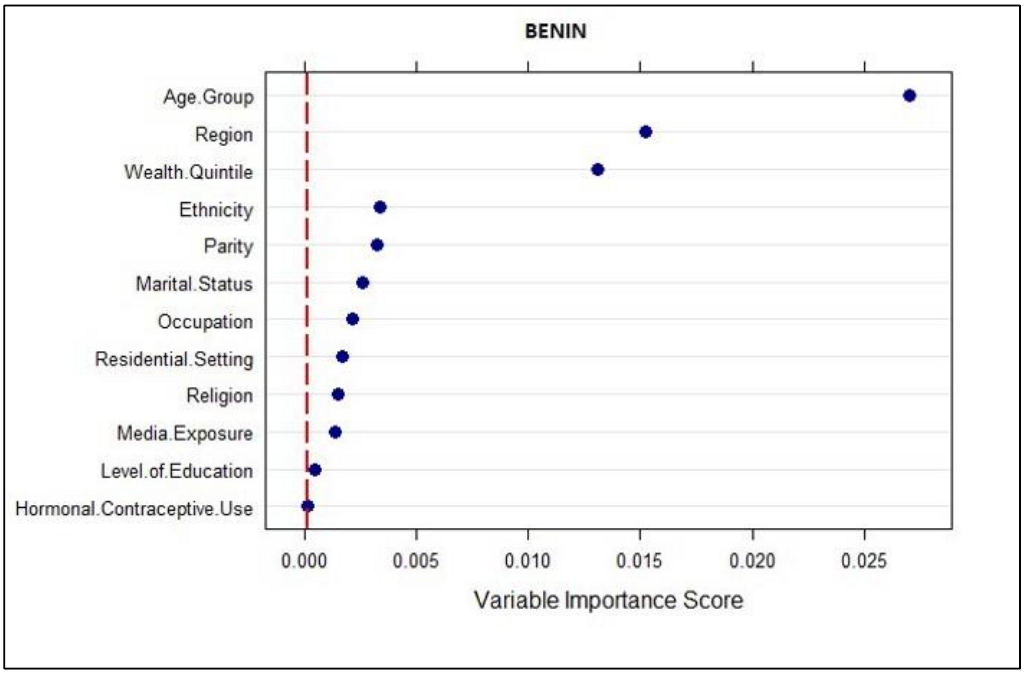

Figure S26: Predictors for nutritional status in Benin (OOB error = 24.5%).

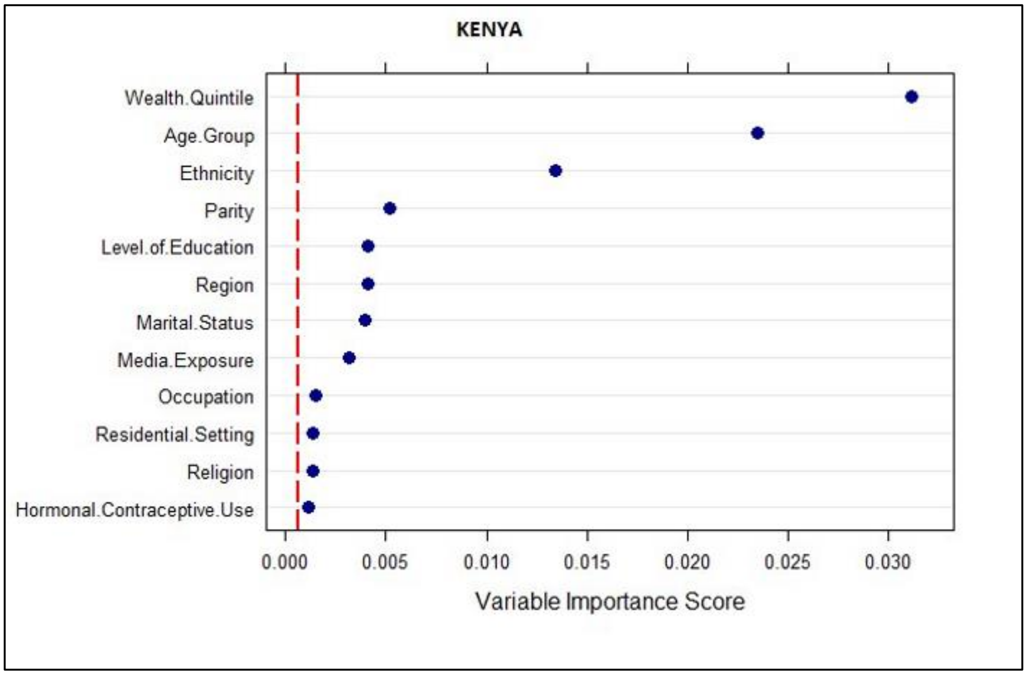

Figure S27: Predictors for nutritional status in Kenya (OOB error = 28.8%).

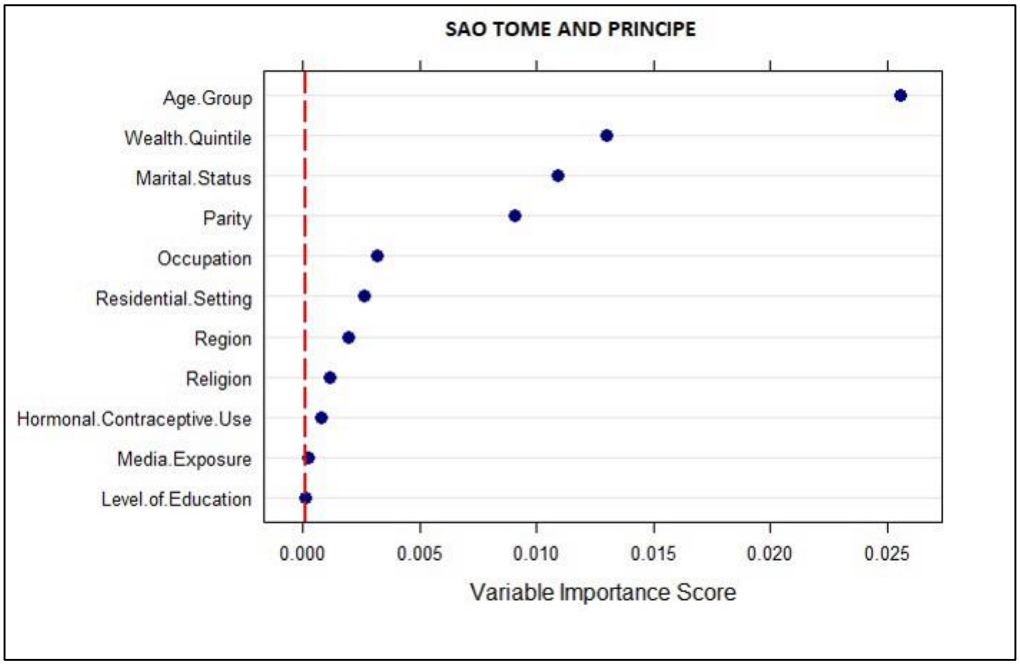

Figure S28: Predictors for nutritional status in Sao Tome and Principe (OOB error = 28.5%).

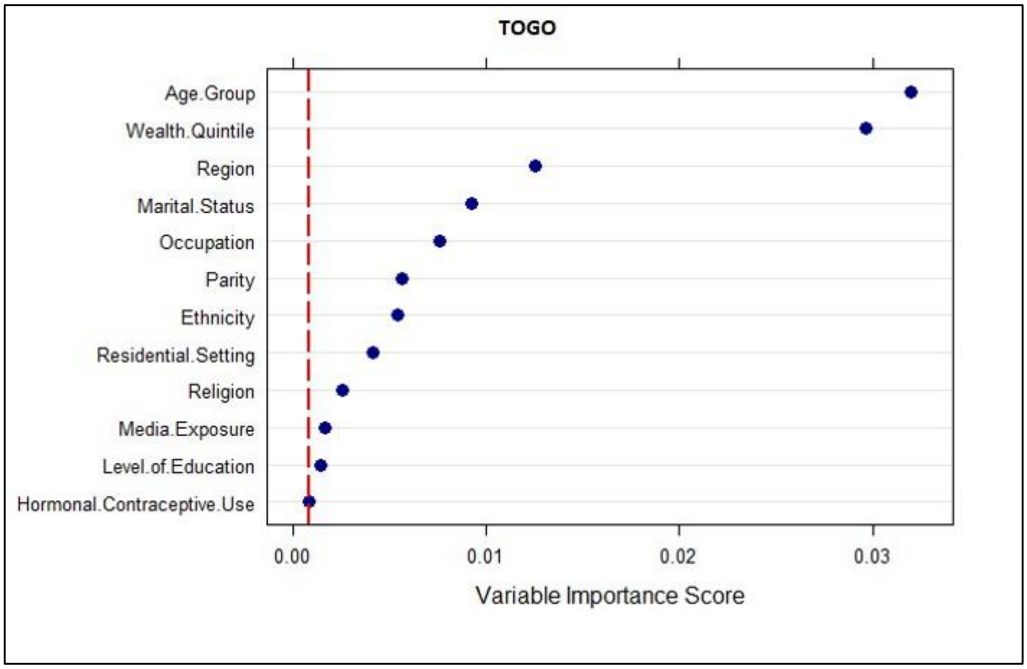

Figure S29: Predictors for nutritional status in Togo (OOB error = 24.7%).

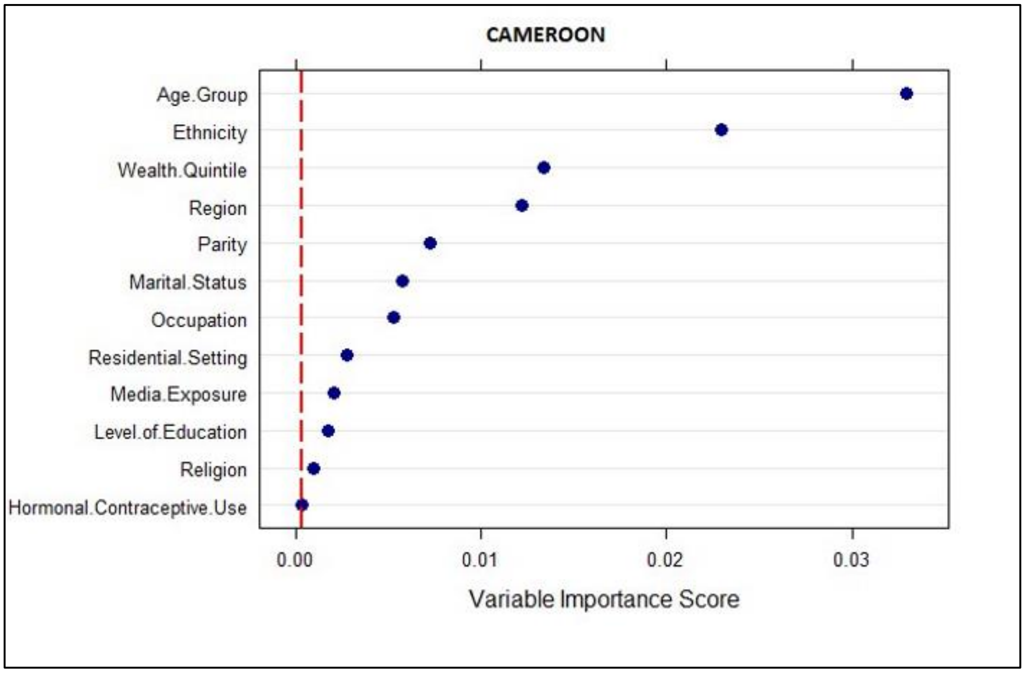

Figure S30: Predictors for nutritional status in Cameroon (OOB error = 24.9%).

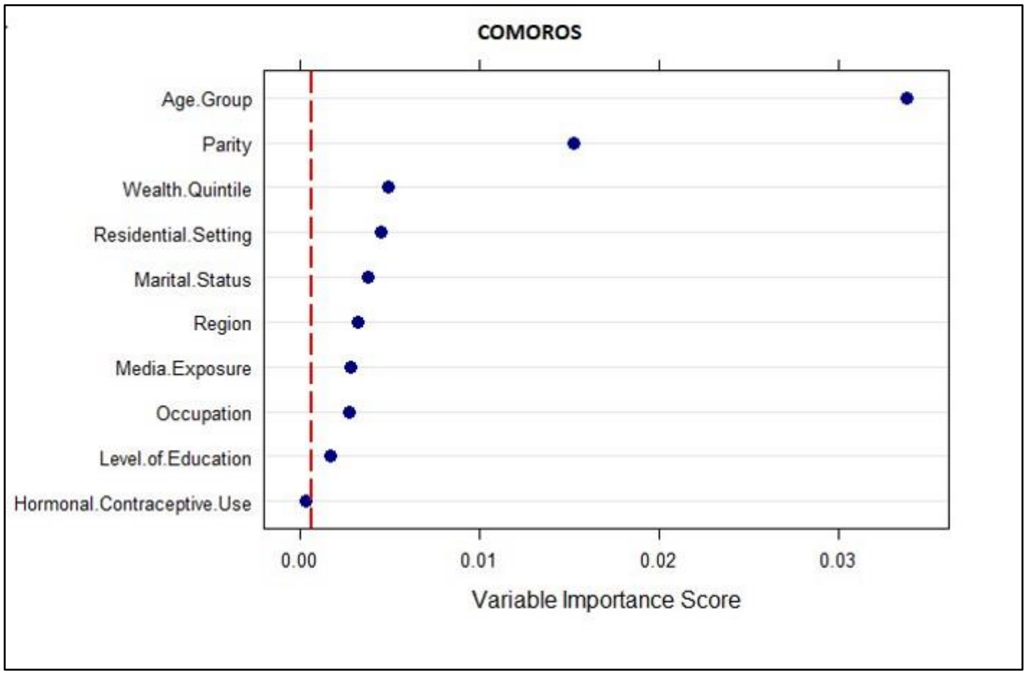

Figure S31: Predictors for nutritional status in Comoros (OOB error = 28.9%).

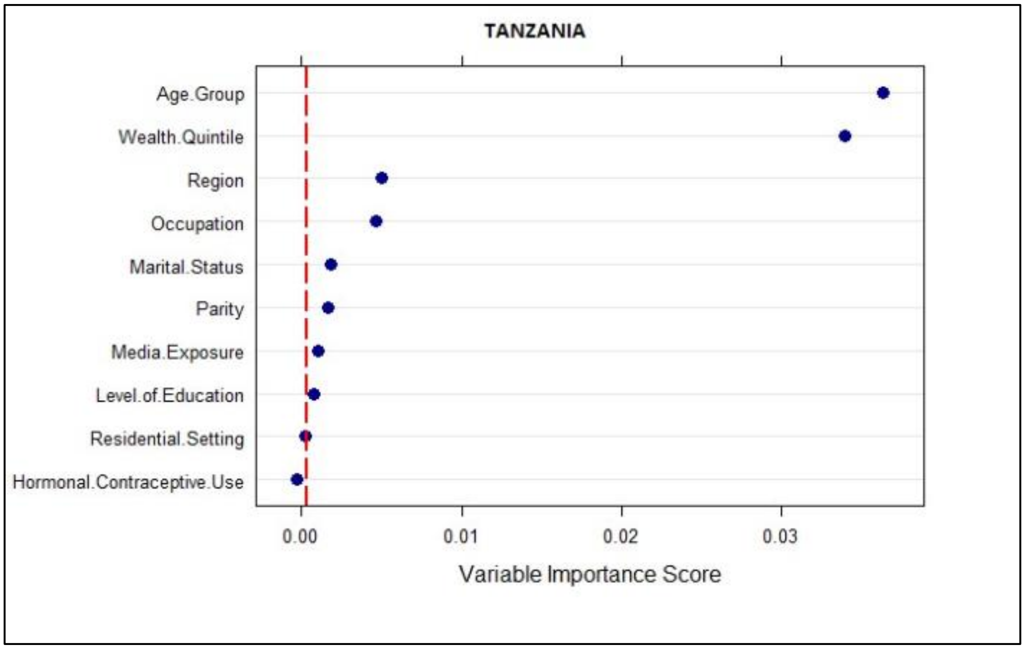

Figure S32: Predictors for nutritional status in Tanzania (OOB error = 26.6%).

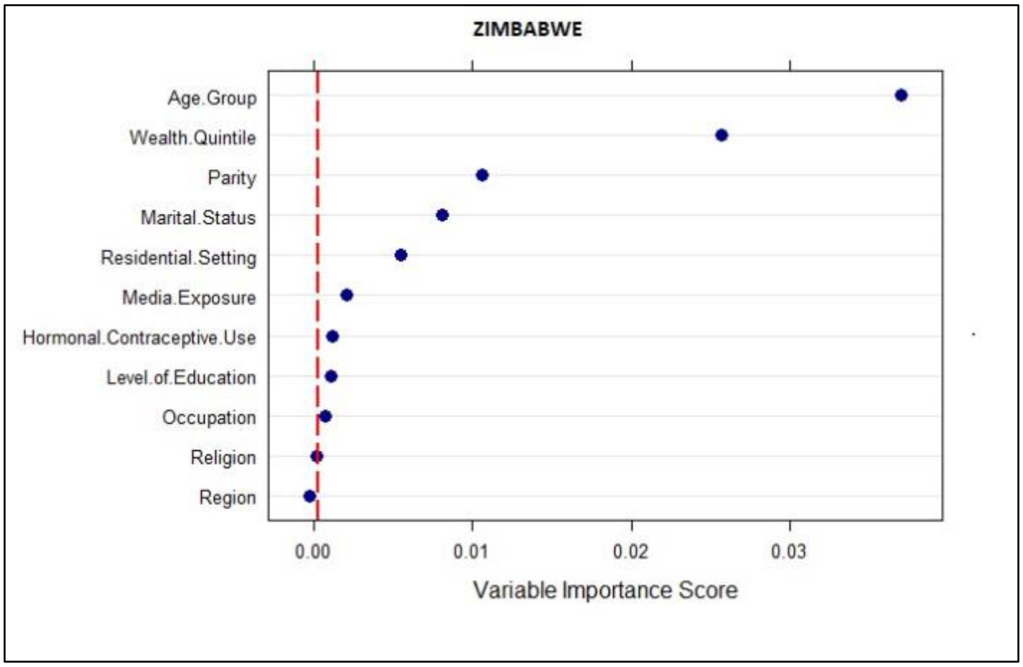

Figure S33: Predictors for nutritional status in Zimbabwe (OOB error = 28.1%).

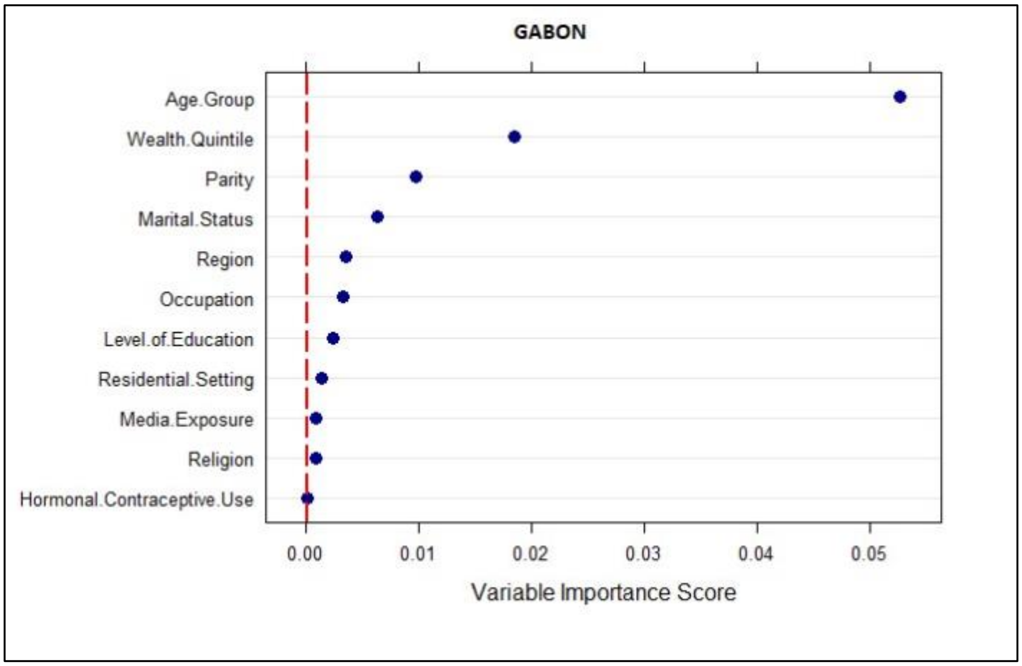

Figure S34: Predictors for nutritional status in Gabon (OOB error = 27.8%).

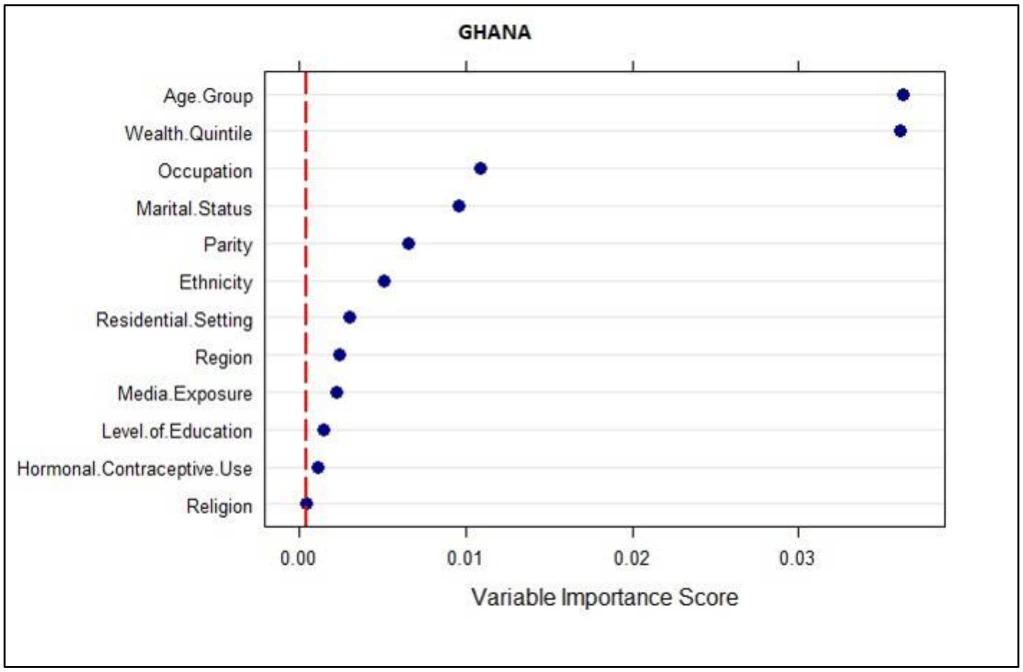

Figure S35: Predictors for nutritional status in Ghana (OOB error = 24.4%).

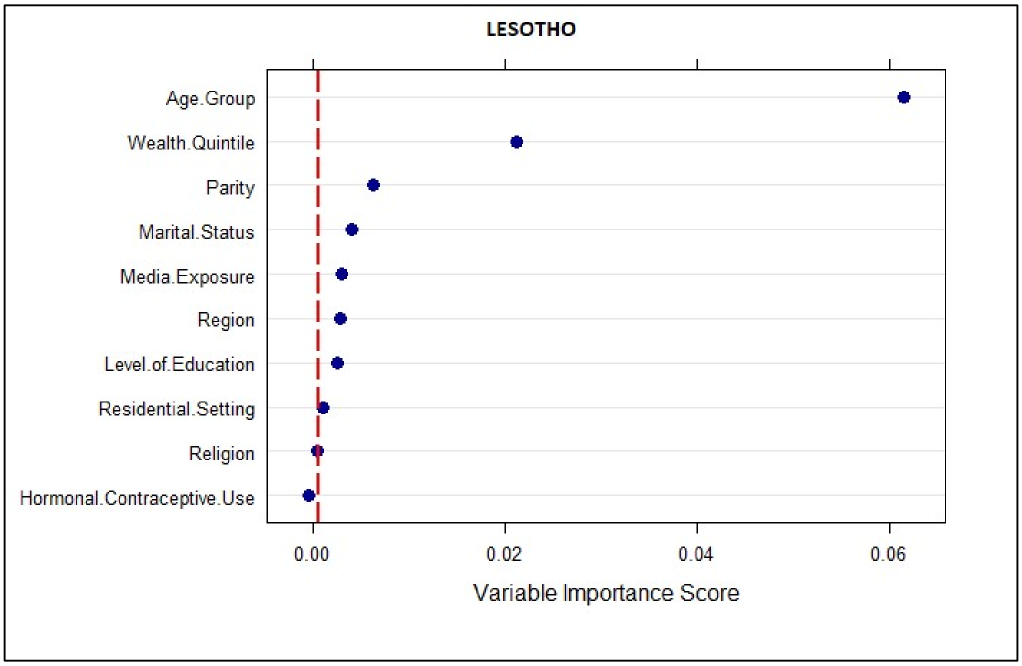

Figure S36: Predictors for nutritional status in Lesotho (OOB error = 27.0%).

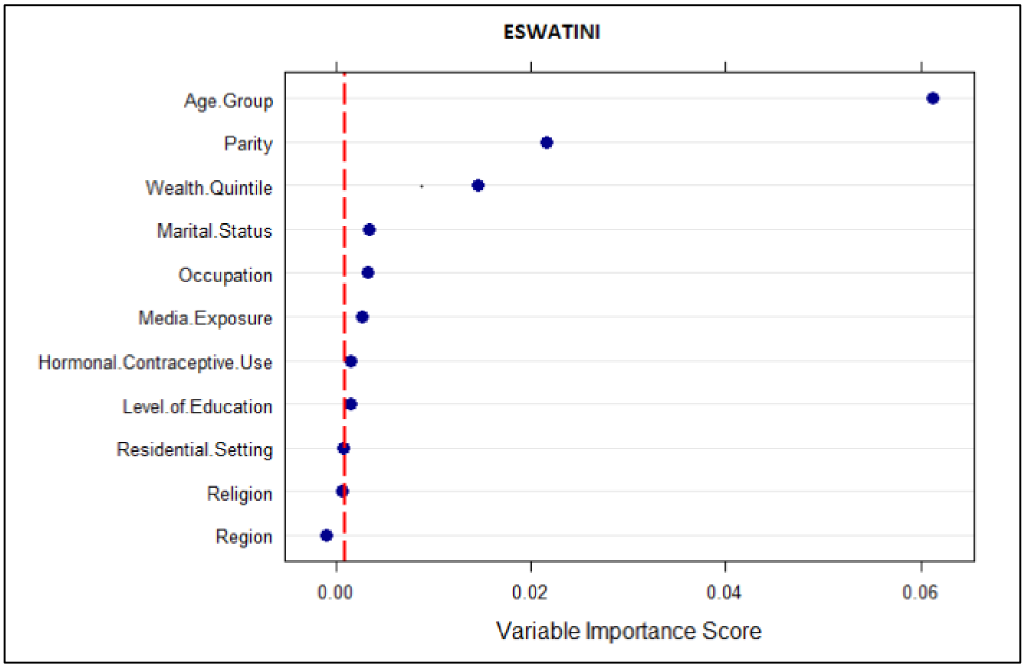

Figure S37: Predictors for nutritional status in Eswatini (OOB error = 27.3%).

Appendix 4: Logistic regression results for individual-level analysis (Table S35-S68).

1. Multivariable multinomial regression models were implemented to calculate the adjusted and unadjusted associations for each country.
2. Normal weight (BMI 18.5 – 24.9 kg/m^2) was used as the reference category for the outcome.
3. Beneath each plot, F-value (P-value) has been presented as the goodness of fit statistic.

Table S35: Unadjusted and adjusted relative risk ratio, of underweight and overweight/obesity among women of child-bearing age in Madagascar.

VARIABLES	UNDERWEIGHT		OVERWEIGHT/OBESITY	
	Unadjusted RRR (95% CI)	Adjusted RRR (95% CI)	Unadjusted RRR (95% CI)	Adjusted RRR (95% CI)
**Age**				
15-19 (Reference)	1.00	1.00	1.00	1.00
20-24	0.80 (0.66, 0.98)	0.84 (0.66, 1.06)	3.93 (2.01, 7.67)	2.78 (1.31, 5.89)
25-29	0.95 (0.75, 1.19)	0.96 (0.71, 1.28)	6.21 (3.26, 11.8)	3.74 (1.67, 8.41)
30-34	0.96 (0.76, 1.22)	1.00 (0.74, 1.34)	10.1 (5.29, 19.2)	5.76 (2.60, 12.8)
35-39	1.11 (0.88, 1.40)	1.16 (0.85, 1.59)	13.1 (7.05, 24.5)	7.32 (3.27, 16.4)
40-44	1.04 (0.83, 1.30)	1.04 (0.77, 1.41)	11.0 (5.72, 21.1)	6.29 (2.76, 14.3)
45-49	1.24 (0.97, 1.58)	1.24 (0.89, 1.73)	10.3 (5.27, 20.1)	6.29 (2.64, 15.0)
**Religion**				
Catholics (Reference)	1.00	1.00	1.00	1.00
Other Christians	0.89 (0.75, 1.06)	0.93 (0.77, 1.12)	1.00 (0.77, 1.29)	1.01 (0.77, 1.31)
Islam	1.24 (0.66, 2.33)	1.24 (0.67, 2.27)	1.93 (0.66, 5.59)	1.08 (0.46, 2.52)
Traditionalist	1.06 (0.64, 1.75)	1.04 (0.65, 1.69)	0.75 (0.30, 1.87)	2.04 (0.65, 6.43)
Others	0.87 (0.66, 1.13)	0.96 (0.73, 1.26)	1.41 (0.91, 2.17)	1.19 (0.78, 1.83)
No Religion	1.15 (0.96, 1.39)	1.02 (0.81, 1.27)	0.49 (0.33, 0.72)	1.26 (0.77, 2.06)
**Parity**				
0 (Reference)	1.00	1.00	1.00	1.00
1	0.94 (0.76, 1.18)	1.03 (0.77, 1.36)	2.32 (1.49, 3.63)	1.23 (0.71, 2.16)
2	0.95 (0.77, 1.18)	1.06 (0.80, 1.40)	4.01 (2.43, 6.62)	1.68 (0.90, 3.15)
3	1.04 (0.80, 1.34)	1.12 (0.81, 1.55)	4.04 (2.59, 6.30)	1.40 (0.74, 2.63)
4	1.19 (0.94, 1.50)	1.23 (0.88, 1.73)	3.13 (1.86, 5.26)	1.22 (0.59, 2.52)
5	1.02 (0.78, 1.35)	0.99 (0.68, 1.43)	3.19 (1.89, 5.36)	1.42 (0.67, 3.01)
6+	1.34 (1.09, 1.65)	1.14 (0.83, 1.57)	2.83 (1.85, 4.33)	1.45 (0.73, 2.88)
**Marital Status**				
Single (Reference)	1.00	1.00	1.00	1.00
Married	0.96 (0.81, 1.14)	0.86 (0.66, 1.12)	5.34 (3.42, 8.36)	1.65 (0.92, 2.96)
Formerly Married	1.13 (0.89, 1.43)	0.92 (0.67, 1.26)	4.84 (2.84, 8.27)	1.57 (0.83, 2.96)
**Residential Setting**				
Rural (Reference)	1.00	1.00	1.00	1.00
Urban	0.80 (0.66, 0.95)	1.11 (0.85, 1.45)	2.73 (2.10, 3.55)	0.84 (0.61, 1.17)

VARIABLES	UNDERWEIGHT		OVERWEIGHT/OBESITY	
	Unadjusted RRR (95% CI)	Adjusted RRR (95% CI)	Unadjusted RRR (95% CI)	Adjusted RRR (95% CI)
**Region**				
Analamanga (Reference)	1.00	1.00	1.00	1.00
Vakinankarata	1.29 (0.88, 1.88)	1.01 (0.65, 1.56)	0.45 (0.22, 0.94)	1.26 (0.67, 2.35)
Itasy	1.53 (1.03, 2.27)	1.25 (0.79, 2.00)	0.40 (0.20, 0.80)	1.21 (0.61, 2.38)
Bongolava	0.18 (0.09, 0.36)	0.14 (0.07, 0.30)	0.58 (0.29, 1.18)	1.85 (0.79, 4.34)
Haute Matsiatra	2.35 (1.56, 3.53)	1.89 (1.19, 2.98)	1.01 (0.57, 1.81)	2.51 (1.45, 4.36)
Anamoronn Mania	2.63 (1.76, 3.95)	2.10 (1.32, 3.36)	0.64 (0.27, 1.53)	1.56 (0.78, 3.14)
Vatovavy Fitovinany	2.11 (1.37, 3.24)	1.39 (0.85, 2.30)	0.59 (0.32, 1.10)	2.53 (1.33, 4.78)
Ihorombe	1.23 (0.80, 1.89)	0.82 (0.50, 1.34)	0.53 (0.25, 1.11)	1.99 (0.90, 4.38)
Atsimo Atsinanana	2.10 (1.29, 3.43)	1.34 (0.78, 2.28)	0.25 (0.08, 0.79)	1.59 (0.54, 4.64)
Atsinanana	1.63 (1.15, 2.32)	1.27 (0.86, 1.86)	0.78 (0.42, 1.47)	1.38 (0.74, 2.58)
Ananlanjirofo	2.14 (1.37, 3.36)	1.53 (0.93, 2.50)	0.41 (0.16, 1.06)	1.44 (0.56, 3.67)
Alaotra Mangoro	1.08 (0.72, 1.63)	0.91 (0.57, 1.43)	0.63 (0.32, 1.22)	1.11 (0.58, 2.12)
Boeny	1.71 (1.11, 2.66)	1.33 (0.81, 2.19)	2.27 (1.32, 3.91)	4.22 (2.52, 7.05)
Sofia	1.01 (0.62, 1.65)	0.67 (0.39, 1.15)	0.26 (0.10, 0.69)	0.88 (0.33, 2.39)
Betsiboka	0.91 (0.60, 1.38)	0.65 (0.39, 1.06)	0.21 (0.08, 0.57)	0.61 (0.30, 1.26)
Melaky	0.85 (0.54, 1.33)	0.53 (0.31, 0.91)	0.33 (0.14, 0.80)	1.48 (0.64, 3.40)
Atsimo Andrefana	1.44 (0.86, 2.39)	0.99 (0.57, 1.73)	1.11 (0.62, 1.98)	3.32 (1.74, 6.30)
Androy	1.53 (1.07, 2.20)	0.91 (0.58, 1.43)	0.41 (0.19, 0.88)	2.56 (1.09, 5.99)
Anosy	1.13 (0.68, 1.86)	0.74 (0.43, 1.29)	0.40 (0.15, 1.07)	1.65 (0.70, 3.93)
Menabe	1.15 (0.68, 1.94)	0.78 (0.46, 1.35)	1.32 (0.80, 2.17)	4.90 (2.69, 8.92)
Diana	1.37 (0.87, 2.17)	1.06 (0.65, 1.74)	0.98 (0.53, 1.81)	2.15 (1.08, 4.27)
Sava	1.03 (0.64, 1.65)	0.81 (0.48, 1.38)	0.59 (0.27, 1.29)	1.88 (0.79, 4.47)
**Level of Education**				
No Education (Reference)	1.00	1.00	1.00	1.00
Primary	0.79 (0.66, 0.93)	0.88 (0.72, 1.07)	1.40 (0.93, 2.10)	1.02 (0.62, 1.68)
Secondary	0.67 (0.56, 0.80)	0.85 (0.66, 1.09)	3.17 (2.11, 4.76)	1.02 (0.59, 1.75)
Higher	0.39 (0.21, 0.72)	0.59 (0.30, 1.15)	4.69 (2.35, 9.36)	0.85 (0.35, 2.04)
**Wealth Quintile**				
Poorest (Reference)	1.00	1.00	1.00	1.00
Poorer	1.03 (0.83, 1.27)	0.97 (0.78, 1.21)	1.23 (0.65, 2.32)	1.44 (0.72, 2.88)
Middle	0.77 (0.61, 0.97)	0.76 (0.58, 0.98)	1.40 (0.73, 2.68)	1.65 (0.81, 3.36)
Richer	0.66 (0.51, 0.85)	0.67 (0.49, 0.91)	2.73 (1.58, 4.74)	3.20 (1.61, 6.37)
Richest	0.57 (0.45, 0.72)	0.55 (0.38, 0.80)	7.87 (4.78, 12.9)	7.11 (3.47, 14.6)
**Occupation**				
Not Working (Reference)	1.00	1.00	1.00	1.00
Nonmanual	0.71 (0.55, 0.91)	0.74 (0.56, 0.98)	4.18 (2.86, 6.11)	1.70 (1.13, 2.55)
Manual	0.85 (0.64, 1.13)	0.83 (0.61, 1.13)	1.84 (1.22, 2.79)	1.06 (0.66, 1.68)
Agricultural	1.11 (0.94, 1.32)	0.87 (0.66, 1.13)	0.73 (0.51, 1.02)	0.90 (0.56, 1.44)
Others	0.62 (0.08, 4.85)	0.65 (0.07, 5.69)	0.52 (0.07, 4.20)	0.27 (0.03, 2.20)
**Media Exposure**				
Not Exposed (Reference)	1.00	1.00	1.00	1.00
Exposed to one media source	0.85 (0.73, 1.00)	1.06 (0.89, 1.27)	1.69 (1.18, 2.43)	1.27 (0.81, 1.98)
Exposed to two media sources	0.68 (0.56, 0.83)	1.06 (0.81, 1.38)	3.87 (2.69, 5.56)	1.73 (1.07, 2.79)
Exposed to three media sources	0.63 (0.50, 0.80)	1.10 (0.78, 1.55)	6.08 (4.20, 8.81)	2.24 (1.30, 3.87)
**Hormonal Contraceptive Use**				
No (Reference)	1.00	1.00	1.00	1.00
Yes	0.87 (0.74, 1.04)	0.92 (0.76, 1.11)	1.41 (1.08, 1.83)	1.12 (0.86, 1.47)

Overall Model Fit: F (112, 440) = 6.49, *P*=0.0000.

Table S36: Unadjusted and adjusted relative risk ratio, of underweight and overweight/obesity among women of child-bearing age in Ethiopia.

VARIABLES	UNDERWEIGHT		OVERWEIGHT/OBESITY	
	Unadjusted RRR (95% CI)	Adjusted RRR (95% CI)	Unadjusted RRR (95% CI)	Adjusted RRR (95% CI)
**Age**				
15-19 (Reference)	1.00	1.00	1.00	1.00
20-24	0.66 (0.55, 0.79)	0.68 (0.55, 0.84)	1.37 (0.90, 2.09)	1.19 (0.76, 1.88)
25-29	0.55 (0.45, 0.66)	0.56 (0.43, 0.73)	2.15 (1.58, 2.94)	1.99 (1.30, 3.05)
30-34	0.64 (0.53, 0.79)	0.62 (0.45, 0.85)	3.26 (2.27, 4.67)	3.79 (2.31, 6.24)
35-39	0.75 (0.62, 0.90)	0.70 (0.51, 0.97)	2.90 (1.95, 4.31)	3.66 (2.18, 6.14)
40-44	0.78 (0.62, 0.97)	0.73 (0.51, 1.05)	3.35 (2.20, 5.09)	5.15 (2.87, 9.25)
45-49	0.88 (0.68, 1.14)	0.81 (0.56, 1.18)	2.90 (1.86, 4.53)	4.40 (2.43, 7.96)
**Ethnicity**				
Oromo (Reference)	1.00	1.00	1.00	1.00
Affar	2.12 (1.56, 2.89)	1.05 (0.58, 1.90)	0.65 (0.36, 1.20)	0.90 (0.42, 1.90)
Amhara	0.81 (0.69, 0.95)	0.89 (0.64, 1.24)	1.00 (0.74, 1.35)	1.07 (0.82, 1.38)
Guragie	1.12 (0.72, 1.75)	1.73 (1.01, 2.98)	2.33 (1.37, 3.98)	0.97 (0.63, 1.50)
Hadiye	0.81 (0.54, 1.23)	1.23 (0.65, 2.30)	0.67 (0.35, 1.28)	0.90 (0.45, 1.80)
Sidama	0.73 (0.47, 1.13)	1.17 (0.65, 2.10)	0.38 (0.21, 0.72)	0.80 (0.35, 1.86)
Somali	1.62 (1.32, 2.00)	1.36 (0.57, 3.25)	2.64 (1.78, 3.90)	2.39 (1.30, 4.38)
Tigray	1.55 (1.28, 1.88)	1.77 (1.05, 2.98)	1.21 (0.82, 1.80)	1.13 (0.79, 1.61)
Welaita	0.44 (0.37, 0.53)	0.70 (0.47, 1.05)	0.64 (0.22, 1.86)	0.68 (0.31, 1.50)
Others	0.42 (0.33, 0.54)	0.61 (0.41, 0.90)	0.79 (0.55, 1.14)	1.10 (0.70, 1.73)
**Religion**				
Other Christians (Reference)	1.00	1.00	1.00	1.00
Catholics	0.53 (0.25, 1.11)	0.60 (0.30, 1.22)	0.85 (0.26, 2.73)	0.84 (0.14, 5.10)
Islam	1.34 (1.17, 1.55)	1.06 (0.91, 1.25)	0.88 (0.65, 1.19)	1.08 (0.80, 1.48)
Others	1.09 (0.48, 2.45)	1.15 (0.67, 1.97)	0.70 (0.30, 1.64)	1.34 (0.46, 3.94)
**Parity**				
0 (Reference)	1.00	1.00	1.00	1.00
1	0.86 (0.70, 1.06)	1.26 (0.94, 1.67)	1.45 (1.06, 2.00)	0.88 (0.54, 1.43)
2	0.75 (0.58, 0.96)	1.21 (0.85, 1.72)	1.81 (1.28, 2.55)	0.93 (0.55, 1.56)
3	0.91 (0.74, 1.11)	1.53 (1.09, 2.14)	2.15 (1.57, 2.96)	1.39 (0.84, 2.32)
4	0.72 (0.56, 0.91)	1.18 (0.82, 1.71)	1.18 (0.82, 1.70)	0.73 (0.42, 1.26)
5	0.99 (0.80, 1.23)	1.54 (1.05, 2.24)	0.87 (0.55, 1.38)	0.68 (0.35, 1.31)
6+	0.96 (0.81, 1.13)	1.33 (0.93, 1.90)	0.91 (0.68, 1.24)	0.67 (0.38, 1.17)
**Marital Status**				
Single (Reference)	1.00	1.00	1.00	1.00
Married	0.82 (0.72, 0.92)	0.77 (0.58, 1.01)	1.37 (1.10, 1.71)	1.68 (1.09, 2.59)
Formerly Married	0.91 (0.74, 1.12)	0.84 (0.61, 1.14)	2.14 (1.60, 2.86)	1.71 (1.06, 2.74)
**Residential Setting**				
Rural (Reference)	1.00	1.00	1.00	1.00
Urban	0.67 (0.57, 0.80)	0.68 (0.50, 0.93)	6.89 (5.15, 9.23)	1.78 (1.23, 2.58)

VARIABLES	UNDERWEIGHT		OVERWEIGHT/OBESITY	
	Unadjusted RRR (95% CI)	Adjusted RRR (95% CI)	Unadjusted RRR (95% CI)	Adjusted RRR (95% CI)
**Region**				
Addis Ababa (Reference)	1.00	1.00	1.00	1.00
Tigray	2.44 (1.91, 3.11)	1.13 (0.69, 1.85)	0.18 (0.13, 0.26)	0.52 (0.30, 0.90)
Afar	3.23 (2.54, 4.12)	2.13 (1.34, 3.39)	0.32 (0.22, 0.46)	1.08 (0.62, 1.91)
Amhara	1.33 (1.07, 1.65)	1.20 (0.84, 1.71)	0.09 (0.07, 0.12)	0.35 (0.22, 0.53)
Oromia	1.55 (1.26, 1.90)	1.12 (0.78, 1.61)	0.21 (0.14, 0.32)	0.80 (0.53, 1.23)
Somali	2.47 (1.92, 3.18)	1.14 (0.45, 2.84)	0.54 (0.41, 0.72)	1.64 (0.89, 3.02)
Benishangul	1.21 (0.95, 1.54)	1.22 (0.86, 1.74)	0.19 (0.14, 0.25)	0.95 (0.60, 1.50)
Snnpr	0.81 (0.64, 1.03)	0.70 (0.48, 1.01)	0.14 (0.10, 0.19)	0.71 (0.41, 1.22)
Gambela	2.25 (1.66, 3.06)	2.61 (1.73, 3.94)	0.27 (0.20, 0.38)	0.60 (0.39, 0.92)
Harari	1.54 (1.17, 2.04)	1.28 (0.90, 1.84)	0.67 (0.53, 0.84)	1.04 (0.77, 1.42)
Dire Dawa	1.69 (1.33, 2.14)	1.42 (1.02, 1.97)	0.74 (0.60, 0.91)	1.04 (0.78, 1.38)
**Level of Education**				
No Education (Reference)	1.00	1.00	1.00	1.00
Primary	1.06 (0.93, 1.20)	1.05 (0.90, 1.24)	1.64 (1.35, 2.00)	1.49 (1.11, 1.99)
Secondary	0.73 (0.58, 0.91)	0.80 (0.61, 1.04)	2.92 (2.23, 3.84)	1.57 (1.04, 2.39)
Higher	1.03 (0.79, 1.35)	1.65 (1.19, 2.30)	5.88 (3.88, 8.92)	1.75 (1.00, 3.04)
**Wealth Quintile**				
Poorest (Reference)	1.00	1.00	1.00	1.00
Poorer	0.77 (0.63, 0.95)	0.85 (0.68, 1.07)	1.01 (0.64, 1.61)	1.43 (0.87, 2.34)
Middle	0.84 (0.68, 1.04)	0.96 (0.76, 1.21)	0.93 (0.58, 1.49)	1.27 (0.75, 2.16)
Richer	0.79 (0.64, 0.96)	0.92 (0.73, 1.16)	1.60 (1.05, 2.43)	2.12 (1.35, 3.32)
Richest	0.61 (0.49, 0.75)	0.82 (0.58, 1.17)	8.24 (5.66, 12.00)	4.36 (2.73, 6.97)
**Occupation**				
Not Working (Reference)	1.00	1.00	1.00	1.00
Nonmanual	0.68 (0.58, 0.80)	0.79 (0.66, 0.94)	2.77 (2.15, 3.55)	1.23 (0.96, 1.59)
Manual	0.70 (0.53, 0.91)	0.75 (0.56, 0.98)	1.35 (0.90, 2.01)	0.77 (0.52, 1.14)
Agricultural	0.84 (0.73, 0.97)	0.77 (0.66, 0.91)	0.42 (0.28, 0.61)	0.64 (0.42, 0.98)
Others	1.10 (0.77, 1.57)	1.11 (0.76, 1.62)	1.02 (0.61, 1.72)	0.61 (0.33, 1.13)
**Media Exposure**				
Not Exposed (Reference)	1.00	1.00	1.00	1.00
Exposed to one media source	0.97 (0.83, 1.14)	1.04 (0.87, 1.24)	1.88 (1.41, 2.49)	0.96 (0.66, 1.40)
Exposed to two media sources	0.87 (0.73, 1.04)	0.99 (0.80, 1.22)	4.02 (2.97, 5.44)	1.34 (0.94, 1.91)
Exposed to three media sources	0.79 (0.63, 1.00)	0.92 (0.68, 1.25)	6.21 (4.64, 8.31)	1.48 (0.91, 2.39)
**Hormonal Contraceptive Use**				
No (Reference)	1.00	1.00	1.00	1.00
Yes	0.73 (0.63, 0.85)	0.89 (0.75, 1.05)	0.98 (0.81, 1.20)	0.90 (0.70, 1.17)

Overall Model Fit: F (104, 514) = 16.72, *P*=0.0000.

Table S37: Unadjusted and adjusted relative risk ratio, of underweight and overweight/obesity among women of child-bearing age in Burundi.

VARIABLES	UNDERWEIGHT		OVERWEIGHT/OBESITY	
	Unadjusted RRR (95% CI)	Adjusted RRR (95% CI)	Unadjusted RRR (95% CI)	Adjusted RRR (95% CI)
**Age**				
15-19 (Reference)	1.00	1.00	1.00	1.00
20-24	0.36 (0.26, 0.48)	0.48 (0.34, 0.68)	1.03 (0.68, 1.57)	1.02 (0.60, 1.76)
25-29	0.47 (0.34, 0.64)	0.69 (0.44, 1.08)	1.26 (0.82, 1.94)	1.14 (0.61, 2.13)
30-34	0.52 (0.37, 0.72)	0.77 (0.46, 1.27)	1.42 (0.92, 2.19)	1.22 (0.60, 2.48)
35-39	0.82 (0.58, 1.17)	1.31 (0.73, 2.36)	1.46 (0.91, 2.34)	1.64 (0.80, 3.39)
40-44	0.88 (0.63, 1.24)	1.42 (0.81, 2.48)	1.48 (0.92, 2.37)	1.98 (0.88, 4.44)
45-49	1.17 (0.84, 1.63)	1.87 (1.03, 3.40)	1.55 (0.99, 2.45)	2.21 (0.96, 5.06)
**Religion**				
Catholics (Reference)	1.00	1.00	1.00	1.00
Other Christians	0.75 (0.60, 0.94)	0.84 (0.67, 1.06)	1.47 (1.14, 1.89)	1.30 (1.00, 1.69)
Islam	0.65 (0.32, 1.32)	0.86 (0.40, 1.86)	4.44 (2.59, 7.62)	1.84 (1.07, 3.15)
Others	1.14 (0.67, 1.94)	1.20 (0.69, 2.06)	1.10 (0.44, 2.73)	1.42 (0.51, 3.99)
**Parity**				
0 (Reference)	1.00	1.00	1.00	1.00
1	0.68 (0.49, 0.95)	2.12 (1.32, 3.40)	0.94 (0.60, 1.48)	0.73 (0.32, 1.67)
2	0.53 (0.36, 0.78)	1.75 (0.95, 3.23)	1.00 (0.64, 1.54)	0.72 (0.30, 1.73)
3	0.69 (0.46, 1.02)	2.21 (1.18, 4.12)	1.28 (0.85, 1.94)	0.95 (0.39, 2.31)
4	0.86 (0.56, 1.33)	2.17 (1.10, 4.29)	1.34 (0.85, 2.12)	0.89 (0.37, 2.14)
5	0.97 (0.65, 1.46)	2.12 (1.04, 4.32)	1.47 (0.94, 2.31)	0.97 (0.39, 2.41)
6+	0.96 (0.74, 1.25)	1.64 (0.84, 3.20)	1.14 (0.78, 1.66)	0.74 (0.28, 1.93)
**Marital Status**				
Single (Reference)	1.00	1.00	1.00	1.00
Married	0.62 (0.50, 0.77)	0.36 (0.22, 0.59)	1.24 (0.95, 1.62)	1.55 (0.72, 3.34)
Formerly Married	1.16 (0.85, 1.56)	0.51 (0.30, 0.87)	0.93 (0.60, 1.46)	1.03 (0.44, 2.39)
**Residential Setting**				
Rural (Reference)	1.00	1.00	1.00	1.00
Urban	0.72 (0.52, 0.99)	0.87 (0.43, 1.77)	6.62 (5.04, 8.70)	2.20 (1.36, 3.56)
**Region**				
Bujumbura (Reference)	1.00	1.00	1.00	1.00
North	1.46 (0.98, 2.17)	1.11 (0.51, 2.41)	0.12 (0.09, 0.18)	0.69 (0.42, 1.15)
Centre East	1.51 (1.01, 2.26)	1.10 (0.51, 2.38)	0.13 (0.09, 0.20)	0.78 (0.45, 1.36)
West	1.32 (0.87, 2.00)	1.12 (0.50, 2.49)	0.16 (0.11, 0.25)	0.95 (0.54, 1.69)
South	1.24 (0.85, 1.83)	1.08 (0.51, 2.29)	0.18 (0.13, 0.25)	0.94 (0.56, 1.58)
**Level of Education**				
No Education (Reference)	1.00	1.00	1.00	1.00
Primary	1.04 (0.83, 1.30)	0.95 (0.76, 1.20)	1.33 (0.99, 1.77)	1.09 (0.76, 1.55)
Secondary	0.81 (0.59, 1.10)	0.62 (0.40, 0.95)	3.13 (2.22, 4.43)	1.19 (0.71, 1.99)
Higher	0.62 (0.18, 2.07)	0.64 (0.18, 2.31)	9.18 (5.40, 15.6)	1.17 (0.59, 2.33)
**Wealth Quintile**				
Poorest (Reference)	1.00	1.00	1.00	1.00
Poorer	0.61 (0.46, 0.81)	0.58 (0.42, 0.78)	0.77 (0.41, 1.43)	0.69 (0.36, 1.30)
Middle	0.70 (0.51, 0.98)	0.67 (0.47, 0.95)	1.68 (0.95, 2.99)	1.39 (0.77, 2.52)
Richer	0.66 (0.49, 0.89)	0.62 (0.45, 0.86)	1.48 (0.84, 2.61)	1.17 (0.63, 2.18)
Richest	0.54 (0.40, 0.74)	0.57 (0.38, 0.84)	5.79 (3.64, 9.20)	1.80 (0.99, 3.27)
**Occupation**				
Not Working (Reference)	1.00	1.00	1.00	1.00
Nonmanual	0.31 (0.18, 0.52)	0.38 (0.22, 0.68)	3.79 (2.61, 5.51)	1.61 (1.06, 2.43)
Agricultural	0.55 (0.44, 0.70)	0.53 (0.41, 0.69)	0.50 (0.36, 0.68)	0.69 (0.42, 1.14)
Others	0.36 (0.10, 1.27)	0.30 (0.08, 1.08)	1.29 (0.54, 3.05)	0.77 (0.31, 1.94)

VARIABLES	UNDERWEIGHT		OVERWEIGHT/OBESITY	
	Unadjusted RRR (95% CI)	Adjusted RRR (95% CI)	Unadjusted RRR (95% CI)	Adjusted RRR (95% CI)
**Media Exposure**				
Not Exposed (Reference)	1.00	1.00	1.00	1.00
Exposed to one media source	0.99 (0.76, 1.28)	1.14 (0.86, 1.51)	1.35 (0.85, 2.12)	1.15 (0.70, 1.91)
Exposed to two media sources	1.03 (0.76, 1.40)	1.14 (0.81, 1.59)	2.83 (1.73, 4.63)	1.58 (0.88, 2.82)
Exposed to three media sources	1.13 (0.73, 1.75)	1.88 (1.11, 3.18)	6.68 (3.95, 11.3)	1.54 (0.79, 2.99)
**Hormonal Contraceptive Use**				
No (Reference)	1.00	1.00	1.00	1.00
Yes	0.60 (0.41, 0.88)	0.90 (0.59, 1.38)	1.91 (1.37, 2.65)	1.65 (1.11, 2.46)

Overall Model Fit: F (72, 272) = 6.88, *P*=0.0000.

Table S38: Unadjusted and adjusted relative risk ratio, of underweight and overweight/obesity among women of child-bearing age in Burkina Faso.

VARIABLES	UNDERWEIGHT		OVERWEIGHT/OBESITY	
	Unadjusted RRR (95% CI)	Adjusted RRR (95% CI)	Unadjusted RRR (95% CI)	Adjusted RRR (95% CI)
**Age**				
15-19 (Reference)	1.00	1.00	1.00	1.00
20-24	0.48 (0.38, 0.60)	0.75 (0.56, 1.02)	1.43 (0.95, 2.14)	1.14 (0.73, 1.78)
25-29	0.46 (0.35, 0.59)	0.78 (0.51, 1.19)	2.21 (1.51, 3.23)	2.04 (1.27, 3.28)
30-34	0.59 (0.47, 0.76)	1.07 (0.71, 1.62)	3.00 (2.09, 4.32)	3.67 (2.27, 5.94)
35-39	0.65 (0.51, 0.82)	1.19 (0.78, 1.81)	2.95 (2.06, 4.21)	4.49 (2.66, 7.56)
40-44	0.79 (0.62, 1.00)	1.51 (0.98, 2.32)	5.43 (3.80, 7.77)	11.1 (6.56, 18.8)
45-49	0.74 (0.57, 0.96)	1.38 (0.87, 2.18)	4.37 (3.01, 6.33)	10.5 (6.07, 18.1)
**Ethnicity**				
Mossi (Reference)	1.00	1.00	1.00	1.00
Bobo	0.72 (0.49, 1.08)	0.89 (0.54, 1.48)	1.31 (0.80, 2.16)	1.33 (0.78, 2.27)
Fulfuldé / Peul	3.01 (2.31, 3.91)	2.81 (2.10, 3.76)	0.71 (0.45, 1.10)	0.95 (0.57, 1.59)
Gourmantché	2.03 (1.47, 2.80)	0.95 (0.61, 1.49)	0.40 (0.23, 0.70)	0.87 (0.46, 1.65)
Gourouns	0.96 (0.59, 1.56)	0.98 (0.58, 1.66)	0.99 (0.63, 1.56)	0.97 (0.58, 1.62)
Lobi	1.01 (0.68, 1.50)	0.67 (0.31, 1.42)	0.62 (0.36, 1.07)	0.86 (0.40, 1.85)
Sénoufo	0.73 (0.44, 1.22)	1.19 (0.66, 2.15)	1.08 (0.75, 1.57)	1.04 (0.63, 1.71)
Dagara	0.67 (0.46, 0.97)	0.52 (0.31, 0.88)	0.91 (0.47, 1.75)	0.85 (0.37, 1.97)
Bissa	0.73 (0.51, 1.04)	0.69 (0.40, 1.18)	0.88 (0.52, 1.50)	1.16 (0.68, 1.98)
Others	0.89 (0.59, 1.36)	1.06 (0.70, 1.59)	1.73 (1.26, 2.38)	1.50(1.06, 2.14)
**Religion**				
Catholics (Reference)	1.00	1.00	1.00	1.00
Other Christians	1.17 (0.83, 1.65)	0.96 (0.69, 1.33)	0.78 (0.53, 1.15)	0.80 (0.53, 1.20)
Islam	1.18 (0.98, 1.43)	1.02 (0.83, 1.25)	0.97 (0.77, 1.23)	1.41 (1.06, 1.87)
Traditionalists	1.34 (0.97, 1.86)	1.09 (0.78, 1.52)	0.26 (0.15, 0.43)	0.60 (0.32, 1.12)
No Religion	0.87 (0.41, 1.85)	0.62 (0.29, 1.32)	0.39 (0.09, 1.75)	0.92 (0.25, 3.44)
**Parity**				
0 (Reference)	1.00	1.00	1.00	1.00
1	0.50 (0.38, 0.66)	0.76 (0.51, 1.13)	1.59 (1.15, 2.21)	1.10 (0.71, 1.71)
2	0.50 (0.38, 0.67)	0.75 (0.48, 1.18)	1.57 (1.14, 2.16)	0.86 (0.53, 1.37)
3	0.45 (0.34, 0.59)	0.64 (0.40, 1.02)	2.02 (1.51, 2.69)	0.93 (0.58, 1.50)
4	0.79 (0.61, 1.02)	1.00 (0.62, 1.63)	2.01 (1.44, 2.80)	0.88 (0.53, 1.45)
5	0.67 (0.50, 0.89)	0.74 (0.44, 1.23)	1.95 (1.37, 2.77)	0.82 (0.47, 1.43)
6+	0.78 (0.64, 0.95)	0.66 (0.41, 1.07)	1.46 (1.11, 1.92)	0.53 (0.30, 0.93)
**Marital Status**				
Single (Reference)	1.00	1.00	1.00	1.00
Married	0.62 (0.52, 0.73)	0.50 (0.34, 0.75)	1.90 (1.44, 2.50)	1.81 (1.10, 2.98)
Formerly Married	0.53 (0.34, 0.83)	0.50 (0.29, 0.88)	2.77 (1.74, 4.41)	1.27 (0.61, 2.63)
**Residential Setting**				
Rural (Reference)	1.00	1.00	1.00	1.00
Urban	0.47 (0.37, 0.58)	0.62 (0.46, 0.84)	4.65 (3.84, 5.64)	1.51 (1.12, 2.05)

VARIABLES	UNDERWEIGHT		OVERWEIGHT/OBESITY	
	Unadjusted RRR (95% CI)	Adjusted RRR (95% CI)	Unadjusted RRR (95% CI)	Adjusted RRR (95% CI)
**Region**				
Centre (Reference)	1.00	1.00	1.00	1.00
Boucle de Mouhoun	1.49 (0.99, 2.23)	0.86 (0.55, 1.35)	0.29 (0.20, 0.42)	0.64 (0.39, 1.04)
Cascades	0.96 (0.58, 1.61)	0.61 (0.33, 1.15)	0.41 (0.29, 0.57)	0.76 (0.46, 1.24)
Centre-Est	1.93 (1.22, 3.03)	1.36 (0.79, 2.33)	0.21 (0.15, 0.30)	0.46 (0.30, 0.71)
Centre-Nord	1.60 (1.02, 2.49)	1.03 (0.64, 1.65)	0.15 (0.08, 0.28)	0.45 (0.23, 0.87)
Centre-Ouest	2.10 (1.41, 3.13)	1.38 (0.89, 2.14)	0.25 (0.15, 0.41)	0.73 (0.44, 1.21)
Centre-Sud	2.25 (1.43, 3.54)	1.53 (0.95, 2.45)	0.14 (0.08, 0.26)	0.40 (0.21, 0.75)
Est	4.19 (2.85, 6.16)	2.61 (1.64, 4.15)	0.19 (0.12, 0.31)	0.88 (0.52, 1.50)
Hauts Basins	1.44 (0.93, 2.22)	1.06 (0.65, 1.71)	0.60 (0.45, 0.79)	0.85 (0.63, 1.16)
Nord	2.30 (1.51, 3.50)	1.44 (0.94, 2.22)	0.16 (0.10, 0.25)	0.41 (0.26, 0.64)
Plateau Central	1.31 (0.83, 2.08)	0.86 (0.54, 1.39)	0.18 (0.12, 0.27)	0.54 (0.33, 0.88)
Sahel	2.83 (1.77, 4.54)	0.93 (0.56, 1.55)	0.28 (0.19, 0.43)	0.83 (0.49, 1.40)
Sud-Ouest	1.74 (1.20, 2.54)	1.70 (0.90, 3.22)	0.22 (0.16, 0.32)	1.01 (0.55, 1.88)
**Level of Education**				
No Education (Reference)	1.00	1.00	1.00	1.00
Primary	0.70 (0.55, 0.89)	0.87 (0.67, 1.13)	2.04(1.65, 2.54)	1.34 (1.02, 1.75)
Secondary	0.70 (0.54, 0.92)	0.78 (0.55, 1.11)	3.04 (2.44, 3.79)	1.74 (1.25, 2.42)
Higher	0.77 (0.40, 1.51)	1.63 (0.71, 3.75)	3.62 (1.98, 6.62)	0.89 (0.44, 1.81)
**Wealth Quintile**				
Poorest (Reference)	1.00	1.00	1.00	1.00
Poorer	0.82 (0.66, 1.01)	0.88 (0.71, 1.08)	1.36 (0.88, 2.10)	1.45 (0.94, 2.26)
Middle	0.67 (0.53, 0.85)	0.78 (0.61, 1.00)	1.50 (0.97, 2.31)	1.59 (1.00, 2.52)
Richer	0.52 (0.41, 0.65)	0.70 (0.54, 0.92)	2.35 (1.50, 3.69)	1.91 (1.17, 3.12)
Richest	0.34 (0.26, 0.45)	0.47 (0.32, 0.67)	8.25 (5.60, 12.2)	3.58 (2.09, 6.13)
**Occupation**				
Not Working (Reference)	1.00	1.00	1.00	1.00
Nonmanual	0.68 (0.53, 0.85)	0.74 (0.58, 0.96)	1.56 (1.23, 1.98)	1.04 (0.82, 1.32)
Manual	0.51 (0.34, 0.76)	0.57 (0.37, 0.87)	0.78 (0.50, 1.21)	0.80 (0.54, 1.19)
Agricultural	0.86 (0.69, 1.06)	0.75 (0.59, 0.95)	0.31 (0.24, 0.41)	0.61 (0.44, 0.84)
Others	0.76 (0.42, 1.36)	0.69 (0.34, 1.39)	0.59 (0.31, 1.10)	0.60 (0.34, 1.07)
**Media Exposure**				
Not Exposed (Reference)	1.00	1.00	1.00	1.00
Exposed to one media source	0.81 (0.69, 0.96)	0.94 (0.79, 1.12)	1.19 (0.92, 1.53)	0.97 (0.74, 1.27)
Exposed to two media sources	0.68 (0.54, 0.85)	1.26 (0.97, 1.65)	3.17 (2.44, 4.12)	1.11 (0.82, 1.50)
Exposed to three media sources	0.54 (0.37, 0.77)	1.27 (0.77, 2.10)	4.83 (3.56, 6.55)	1.11 (0.73, 1.70)
**Hormonal Contraceptive Use**				
No (Reference)	1.00	1.00	1.00	1.00
Yes	0.59 (0.45, 0.76)	0.87 (0.67, 1.15)	2.33 (1.93, 2.81)	1.54 (1.20, 1.97)

Overall Model Fit: F (110, 438) = 10.81, *P*=0.0000.

Table S39: Unadjusted and adjusted relative risk ratio, of underweight and overweight/obesity among women of child-bearing age in Chad.

VARIABLES	UNDERWEIGHT		OVERWEIGHT/OBESITY	
	Unadjusted RRR (95% CI)	Adjusted RRR (95% CI)	Unadjusted RRR (95% CI)	Adjusted RRR (95% CI)
**Age**				
15-19 (Reference)	1.00	1.00	1.00	1.00
20-24	0.71 (0.57, 0.88)	0.80 (0.62, 1.03)	1.40 (0.98, 1.99)	1.51 (0.90, 2.54)
25-29	0.71 (0.58, 0.85)	0.87 (0.64, 1.18)	2.27 (1.65, 3.12)	3.30 (1.84, 5.92)
30-34	0.59 (0.47, 0.73)	0.83 (0.59, 1.15)	3.13 (2.27, 4.31)	5.31 (2.85, 9.90)
35-39	0.66 (0.52, 0.84)	0.93 (0.63, 1.36)	4.33 (3.08, 6.08)	7.62 (3.86, 15.0)
40-44	0.72 (0.57, 0.92)	1.05 (0.73, 1.51)	4.88 (3.49, 6.82)	9.01 (4.35, 18.6)
45-49	0.68 (0.53, 0.88)	0.97 (0.64, 1.47)	4.33 (3.03, 6.17)	8.40 (4.26, 16.6)
**Ethnicity**				
Sara (Ngambaye/Sara Madjin-Gaye/Mbaye) (Reference)	1.00	1.00	1.00	1.00
Gorane	5.93 (4.03, 8.72)	2.35 (1.18, 4.68)	1.76 (0.88, 3.51)	1.56 (0.60, 4.11)
Arab	4.02 (2.77, 5.84)	2.44 (1.29, 4.61)	1.82 (0.90, 3.69)	1.32 (0.53, 3.30)
Baguirmi/Barma	1.61 (0.79, 3.25)	0.91 (0.40, 2.10)	4.72 (2.24, 9.93)	3.55 (1.39, 9.09)
Kanembou/Bornou/Boudouma	4.43 (3.05, 6.44)	1.81 (0.92, 3.56)	1.30 (0.62, 2.73)	1.70 (0.65, 4.45)
Boulala/Médégo/Kouka	4.19 (2.77, 6.33)	2.17 (1.06, 4.41)	1.58 (0.74, 3.36)	1.10 (0.42, 2.89)
Ouadaï/Maba/Massalit/Mimi	2.65 (1.77, 3.99)	1.75 (0.86, 3.55)	1.21 (0.59, 2.46)	1.09 (0.44, 2.73)
Zaghawa/Bideyat/Kobé	5.64 (3.49, 9.10)	4.07 (1.91, 8.67)	2.80 (1.04, 7.53)	1.81 (0.57, 5.70)
Dadajo/Kibet/Mouro	2.19 (1.35, 3.54)	1.21 (0.58, 2.52)	1.00 (0.36, 2.76)	1.00 (0.33, 3.06)
Bidio/Migami/Kenga/Dangléat	2.23 (1.26, 3.93)	1.38 (0.65, 2.94)	1.08 (0.43, 2.69)	1.08 (0.28, 4.15)
Moundang	0.78 (0.40, 1.50)	1.25 (0.53, 2.98)	1.94 (0.95, 3.96)	1.43 (0.54, 3.78)
Massa/Mousseye/Mousgoume	0.75 (0.40, 1.40)	0.62 (0.31, 1.26)	0.59 (0.25, 1.40)	0.61 (0.26, 1.41)
Toupouri/Kéra	0.79 (0.54, 1.14)	1.03 (0.57, 1.86)	2.43 (1.31, 4.52)	1.90 (0.88, 4.10)
Peul/Foulbé/Bodoré	6.09 (3.54, 10.5)	3.84 (1.89, 7.80)	2.16 (0.90, 5.19)	1.41 (0.48, 4.16)
Tama/Assongori/Mararit	2.16 (1.25, 3.74)	1.64 (0.68, 3.94)	1.26 (0.59, 2.69)	1.49 (0.52, 4.24)
Gabri/Kabalaye/Nangtchéré/Soumraye	0.86 (0.45, 1.63)	1.32 (0.57, 3.06)	2.70 (1.23, 5.93)	2.17 (0.80, 5.89)
Marba/Lélé/Mesmé	0.72 (0.42, 1.23)	1.12 (0.53, 2.33)	0.81 (0.35, 1.87)	0.68 (0.28, 1.66)
Karo/Zimé/Pévé	0.49 (0.17, 1.44)	0.85 (0.24, 3.03)	1.79 (0.87, 3.66)	1.78 (0.67, 4.72)
Others	1.79 (1.09, 2.92)	1.39 (0.72, 2.69)	3.11 (1.55, 6.26)	2.37 (1.04, 5.38)
**Religion**				
Catholics (Reference)	1.00	1.00	1.00	1.00
Other Christians	1.04 (0.75, 1.44)	0.88 (0.62, 1.24)	1.21 (0.99, 1.49)	1.27 (1.02, 1.58)
Islam	4.77 (3.85, 5.91)	2.02 (1.23, 3.31)	0.92 (0.72, 1.18)	1.80 (1.12, 2.90)
Traditionalists	1.88 (0.46, 7.64)	1.23 (0.26, 5.82)	0.30 (0.04, 2.18)	0.70 (0.09, 5.26)
No Religion	0.85 (0.54, 1.33)	0.78 (0.48, 1.27)	0.37 (0.20, 0.69)	0.69 (0.34, 1.41)
**Parity**				
0 (Reference)	1.00	1.00	1.00	1.00
1	0.79 (0.60, 1.04)	1.26 (0.87, 1.83)	0.91 (0.58, 1.45)	0.82 (0.35, 1.91)
2	0.70 (0.55, 0.90)	1.06 (0.72, 1.56)	1.59 (1.11, 2.29)	1.07 (0.51, 2.24)
3	0.74 (0.57, 0.96)	1.09 (0.74, 1.62)	1.41 (0.94, 2.12)	0.74 (0.35, 1.56)
4	0.78 (0.61, 0.99)	1.10 (0.72, 1.69)	1.84 (1.23, 2.75)	0.94 (0.43, 2.04)
5	0.69 (0.54, 0.87)	0.85 (0.57, 1.27)	1.58 (1.08, 2.30)	0.72 (0.35, 1.47)
6+	0.63 (0.53, 0.74)	0.81 (0.56, 1.17)	2.13 (1.62, 2.80)	0.76 (0.39, 1.49)
**Marital Status**				
Single (Reference)	1.00	1.00	1.00	1.00
Married	0.72 (0.61, 0.85)	0.72 (0.51, 1.01)	1.58 (1.20, 2.09)	0.92 (0.38, 2.22)
Formerly Married	0.67 (0.51, 0.87)	0.74 (0.49, 1.13)	2.90 (2.04, 4.11)	1.09 (0.42, 2.81)

VARIABLES	UNDERWEIGHT		OVERWEIGHT/OBESITY	
	Unadjusted RRR (95% CI)	Adjusted RRR (95% CI)	Unadjusted RRR (95% CI)	Adjusted RRR (95% CI)
**Residential Setting**				
Rural (Reference)	1.00	1.00	1.00	1.00
Urban	0.95 (0.79, 1.14)	1.20 (0.84, 1.73)	3.71 (3.10, 4.43)	1.81 (1.33, 2.44)
**Region**				
N'Djaména (Reference)	1.00	1.00	1.00	1.00
Batha	2.31 (1.66, 3.22)	1.24 (0.80, 1.93)	0.30 (0.17, 0.52)	0.64 (0.33, 1.25)
Borkou, Tibesti	1.76 (1.19, 2.62)	0.82 (0.53, 1.29)	0.82 (0.59, 1.14)	1.17 (0.69, 1.96)
Chari Baguirmi	1.77 (1.22, 2.57)	1.12 (0.76, 1.63)	0.26 (0.18, 0.39)	0.53 (0.32, 0.88)
Guéra	1.25 (0.82, 1.89)	0.90 (0.59, 1.40)	0.21 (0.10, 0.45)	0.44 (0.19, 1.01)
Hadjer-Lamis	1.55 (1.08, 2.22)	0.77 (0.52, 1.14)	0.21 (0.12, 0.38)	0.46 (0.26, 0.82)
Kanem	3.38 (2.50, 4.57)	1.65 (1.06, 2.56)	0.14 (0.08, 0.24)	0.25 (0.12, 0.52)
Lac	2.70 (2.09, 3.50)	1.54 (1.00, 2.37)	0.09 (0.05, 0.16)	0.19 (0.09, 0.40)
Logone Occidental	0.45 (0.27, 0.77)	0.93 (0.55, 1.58)	0.57 (0.42, 0.79)	0.95 (0.64, 1.40)
Logone Oriental	0.56 (0.39, 0.82)	1.16 (0.75, 1.78)	0.33 (0.25, 0.45)	0.69 (0.46, 1.02)
Mandoul	0.46 (0.34, 0.62)	1.00 (0.66, 1.52)	0.28 (0.19, 0.41)	0.58 (0.37, 0.92)
Mayo Kebbi Est	0.52 (0.34, 0.80)	1.33 (0.77, 2.27)	0.15 (0.09, 0.26)	0.70 (0.39, 1.28)
Mayo Kebbi Ouest	0.38 (0.24, 0.61)	0.65 (0.33, 1.27)	0.31 (0.23, 0.44)	0.82 (0.47, 1.41)
Moyen Chari	0.40 (0.26, 0.63)	0.65 (0.40, 1.06)	0.46 (0.30, 0.72)	0.75 (0.48, 1.18)
Ouaddaï	1.33 (0.91, 1.94)	0.79 (0.50, 1.25)	0.20 (0.12, 0.33)	0.48 (0.25, 0.91)
Salamat	1.52 (1.02, 2.27)	0.89 (0.56, 1.41)	0.16 (0.09, 0.29)	0.31 (0.17, 0.58)
Tandjilé	0.35 (0.23, 0.53)	0.74 (0.40, 1.40)	0.28 (0.18, 0.42)	0.78 (0.44, 1.38)
Wadi Fira	1.31 (0.95, 1.81)	0.60 (0.37, 0.98)	0.19 (0.13, 0.29)	0.50 (0.26, 0.95)
Barh El Gazal	3.28 (2.19, 4.90)	1.56 (0.96, 2.53)	0.28 (0.15, 0.52)	0.47 (0.23, 0.95)
Ennedi Est, Ennedi Ouest	2.18 (1.21, 3.91)	0.81 (0.40, 1.64)	0.50 (0.27, 0.92)	0.93 (0.44, 1.97)
Sila	1.87 (1.24, 2.82)	1.19 (0.74, 1.92)	0.14 (0.07, 0.28)	0.36 (0.18, 0.73)
**Level of Education**				
No Education (Reference)	1.00	1.00	1.00	1.00
Primary	0.44 (0.36, 0.52)	0.98 (0.80, 1.20)	1.43 (1.14, 1.79)	1.37 (1.01, 1.85)
Secondary	0.55 (0.44, 0.68)	1.22 (0.90, 1.66)	1.69 (1.34, 2.14)	1.48 (1.02, 2.14)
Higher	0.26 (0.08, 0.86)	0.67 (0.18, 2.53)	6.15 (3.63, 10.4)	1.79 (0.96, 3.31)
**Wealth Quintile**				
Poorest (Reference)	1.00	1.00	1.00	1.00
Poorer	1.09 (0.88, 1.35)	0.98 (0.79, 1.22)	1.19 (0.89, 1.59)	1.41 (1.02, 1.94)
Middle	1.41 (1.15, 1.73)	1.07 (0.87, 1.31)	1.02 (0.71, 1.46)	1.15 (0.79, 1.68)
Richer	1.61 (1.29, 2.01)	1.05 (0.79, 1.38)	1.22 (0.87, 1.70)	1.37 (0.94, 1.97)
Richest	1.12 (0.91, 1.37)	0.72 (0.52, 0.99)	4.02 (3.06, 5.26)	1.73 (1.20, 2.49)
**Occupation**				
Not Working (Reference)	1.00	1.00	1.00	1.00
Nonmanual	0.35 (0.29, 0.42)	0.73 (0.61, 0.88)	1.67 (1.35, 2.06)	1.25 (0.99, 1.59)
Manual	0.88 (0.61, 1.27)	0.72 (0.50, 1.03)	0.57 (0.23, 1.43)	0.45 (0.15, 1.36)
Agricultural	0.71 (0.58, 0.86)	1.05 (0.85, 1.30)	0.78 (0.54, 1.11)	1.00 (0.68, 1.47)
Others	0.41 (0.17, 0.99)	0.47 (0.24, 0.95)	0.21 (0.05, 0.91)	0.32 (0.07, 1.49)
**Media Exposure**				
Not Exposed (Reference)	1.00	1.00	1.00	1.00
Exposed to one media source	0.72 (0.59, 0.88)	0.97 (0.77, 1.22)	1.97 (1.50, 2.59)	1.17 (0.85, 1.61)
Exposed to two media sources	0.66 (0.51, 0.85)	0.83 (0.60, 1.13)	2.44 (1.86, 3.21)	1.02 (0.71, 1.46)
Exposed to three media sources	0.72 (0.51, 1.03)	0.90 (0.56, 1.46)	3.80 (2.85, 5.06)	1.25 (0.79, 1.99)
**Hormonal Contraceptive Use**				
No (Reference)	1.00	1.00	1.00	1.00
Yes	0.23 (0.12, 0.46)	0.48 (0.23, 1.01)	1.93 (1.37, 2.74)	0.91 (0.62, 1.33)

Overall Model Fit: F (144, 430) = 14.19, *P*=0.0000.

Table S40: Unadjusted and adjusted relative risk ratio, of underweight and overweight/obesity among women of child-bearing age in Democratic Republic of Congo.

VARIABLES	UNDERWEIGHT		OVERWEIGHT/OBESITY	
	Unadjusted RRR (95% CI)	Adjusted RRR (95% CI)	Unadjusted RRR (95% CI)	Adjusted RRR (95% CI)
**Age**				
15-19 (Reference)	1.00	1.00	1.00	1.00
20-24	0.73 (0.56, 0.94)	0.90 (0.62, 1.30)	1.40 (1.01, 1.92)	1.25 (0.80, 1.93)
25-29	0.58 (0.44, 0.76)	0.64 (0.40, 1.00)	2.05 (1.51, 2.79)	2.01 (1.28, 3.15)
30-34	0.58 (0.42, 0.80)	0.66 (0.38, 1.15)	2.76 (2.07, 3.68)	2.76 (1.75, 4.34)
35-39	0.67 (0.50, 0.92)	0.88 (0.54, 1.45)	3.50 (2.59, 4.73)	3.53 (2.09, 5.96)
40-44	0.66 (0.46, 0.95)	0.85 (0.48, 1.48)	3.29 (2.35, 4.61)	3.51 (2.02, 6.09)
45-49	0.42 (0.29, 0.60)	0.48 (0.27, 0.84)	3.31 (2.40, 4.55)	4.56 (2.76, 7.52)
**Ethnicity**				
Kasai, Katanga, Tanganyika (Reference)	1.00	1.00	1.00	1.00
Bakongo Nord & Sud	1.18 (0.75, 1.84)	1.13 (0.68, 1.86)	1.90 (1.31, 2.76)	1.19 (0.68, 2.08)
Bas Kasaï et Kwilu-Kwango	1.64 (1.33, 2.02)	1.42 (1.01, 2.01)	0.76 (0.50, 1.14)	0.98 (0.56, 1.73)
Cuvette central	1.21 (0.92, 1.59)	0.90 (0.59, 1.37)	0.75 (0.42, 1.34)	1.06 (0.58, 1.92)
Ubangi et Itimbiri	0.49 (0.36, 0.67)	0.34 (0.18, 0.61)	0.98 (0.64, 1.50)	1.25 (0.68, 2.29)
Uele Lac Albert	0.52 (0.36, 0.75)	0.41 (0.16, 1.06)	1.68 (1.15, 2.44)	1.33 (0.70, 2.53)
Basele-K, Man. et Kivu	0.45 (0.33, 0.62)	0.34 (0.08, 1.40)	2.26 (1.53, 3.35)	2.23 (1.14, 4.35)
Others	1.59 (0.71, 3.58)	1.43 (0.74, 2.75)	2.49 (0.97, 6.41)	1.97 (0.70, 5.53)
**Religion**				
Catholics (Reference)	1.00	1.00	1.00	1.00
Other Christians	1.01 (0.81, 1.26)	0.97 (0.78, 1.21)	1.18 (0.97, 1.44)	1.18 (0.97, 1.43)
Islam	0.64 (0.32, 1.27)	0.92 (0.45, 1.91)	1.83 (1.03, 3.24)	1.64 (0.84, 3.20)
Traditionalists	3.68 (1.43, 9.46)	2.22 (0.84, 5.84)	2.06 (0.48, 8.86)	2.74 (0.88, 8.50)
Others	0.97 (0.50, 1.91)	0.85 (0.39, 1.84)	0.80 (0.47, 1.57)	1.13 (0.56, 2.31)
**Parity**				
0 (Reference)	1.00	1.00	1.00	1.00
1	0.85 (0.63, 1.16)	1.02 (0.68, 1.53)	0.92 (0.67, 1.28)	0.67 (0.42, 1.07)
2	0.58 (0.42, 0.78)	0.75 (0.47, 1.19)	1.30 (0.97, 1.75)	0.78 (0.47, 1.29)
3	0.77 (0.56, 1.05)	1.07 (0.65, 1.77)	1.60 (1.19, 2.14)	0.86 (0.51, 1.46)
4	0.95 (0.69, 1.31)	1.33 (0.75, 2.37)	1.34 (0.95, 1.90)	0.69 (0.41, 1.15)
5	0.76 (0.55, 1.07)	0.99 (0.58, 1.68)	1.52 (1.16, 2.01)	0.80 (0.49, 1.33)
6+	0.56 (0.43, 0.73)	0.73 (0.44, 1.19)	1.75 (1.39, 2.20)	0.78 (0.49, 1.25)
**Marital Status**				
Single (Reference)	1.00	1.00	1.00	1.00
Married	0.67 (0.55, 0.82)	0.70 (0.49, 1.02)	1.66 (1.35, 2.03)	2.20 (1.46, 3.32)
Formerly Married	0.75 (0.58, 0.96)	0.87 (0.59, 1.30)	1.72 (1.31, 2.26)	1.63 (1.03, 2.59)
**Residential Setting**				
Rural (Reference)	1.00	1.00	1.00	1.00
Urban	0.59 (0.49, 0.71)	0.77 (0.56, 1.06)	3.03 (2.21, 4.16)	1.03 (0.69, 1.54)

VARIABLES	UNDERWEIGHT		OVERWEIGHT/OBESITY	
	Unadjusted RRR (95% CI)	Adjusted RRR (95% CI)	Unadjusted RRR (95% CI)	Adjusted RRR (95% CI)
**Region**				
Kinshasa (Reference)	1.00	1.00	1.00	1.00
Bandundu	3.08 (2.25, 4.21)	1.29 (0.81, 2.08)	0.11 (0.07, 0.18)	0.49 (0.28, 0.84)
Bas-Congo	3.05 (1.66, 5.59)	1.55 (0.84, 2.89)	0.27 (0.15, 0.48)	0.53 (0.32, 0.89)
Equateur	1.40 (0.98, 1.99)	1.53 (0.83, 2.81)	0.22 (0.14, 0.32)	0.78 (0.48, 1.28)
Kasai-Occidental	1.62 (1.16, 2.27)	0.86 (0.53, 1.41)	0.18 (0.10, 0.33)	0.59 (0.31, 1.14)
Kasai-Oriental	1.97 (1.42, 2.72)	1.16 (0.73, 1.84)	0.25 (0.17, 0.38)	0.68 (0.38, 1.21)
Katanga	2.00 (1.42, 2.83)	1.12 (0.69, 1.81)	0.41 (0.26, 0.65)	1.06 (0.59, 1.88)
Maniema	1.04 (0.64, 1.72)	1.67 (0.40, 7.07)	0.41 (0.30, 0.55)	0.73 (0.35, 1.50)
Nord-Kivu	0.71 (0.47, 1.07)	1.05 (0.27, 4.06)	0.77 (0.54, 1.09)	1.29 (0.68, 2.45)
Orientale	0.92 (0.60, 1.41)	1.15 (0.41, 3.21)	0.57 (0.42, 0.78)	1.74 (1.00, 3.03)
Sud-Kivu	0.91 (0.46, 1.78)	1.45 (0.35, 6.05)	0.76 (0.39, 1.49)	1.32 (0.61, 2.83)
**Level of Education**				
No Education (Reference)	1.00	1.00	1.00	1.00
Primary	1.36 (1.10, 1.67)	1.15 (0.93, 1.43)	1.13 (0.81, 1.58)	1.18 (0.85, 1.64)
Secondary	1.09 (0.84, 1.42)	0.86 (0.62, 1.19)	1.63 (1.23, 2.16)	1.19 (0.85, 1.68)
Higher	0.67 (0.40, 1.12)	0.89 (0.48, 1.66)	3.50 (2.48, 4.93)	1.41 (0.82, 2.43)
**Wealth Quintile**				
Poorest (Reference)	1.00	1.00	1.00	1.00
Poorer	1.13 (0.89, 1.45)	1.11 (0.87, 1.42)	1.02 (0.67, 1.54)	0.98 (0.62, 1.54)
Middle	0.87 (0.66, 1.15)	0.89 (0.66, 1.19)	1.42 (0.98, 2.04)	1.18 (0.78, 1.77)
Richer	0.77 (0.54, 1.10)	0.88 (0.64, 1.21)	2.85 (1.70, 4.78)	2.06 (1.33, 3.19)
Richest	0.51 (0.39, 0.66)	0.61 (0.40, 0.92)	5.97 (4.40, 8.08)	4.34 (2.78, 6.77)
**Occupation**				
Not Working (Reference)	1.00	1.00	1.00	1.00
Nonmanual	0.50 (0.38, 0.64)	0.64 (0.49, 0.85)	1.60 (1.35, 1.91)	1.04 (0.85, 1.26)
Manual	0.14 (0.03, 0.69)	0.25 (0.05, 1.36)	1.08 (0.42, 2.77)	0.66 (0.25, 1.79)
Agricultural	0.95 (0.79, 1.14)	0.93 (0.74, 1.18)	0.49 (0.38, 0.64)	0.56 (0.42, 0.74)
**Media Exposure**				
Not Exposed (Reference)	1.00	1.00	1.00	1.00
Exposed to one media source	0.81 (0.63, 1.03)	1.00 (0.74, 1.35)	1.71 (1.33, 2.20)	0.99 (0.78, 1.25)
Exposed to two media sources	0.68 (0.50, 0.91)	0.92 (0.64, 1.31)	2.65 (2.15, 3.27)	1.15 (0.85, 1.55)
Exposed to three media sources	0.67 (0.42, 1.06)	1.06 (0.62, 1.80)	3.43 (2.54, 4.63)	1.28 (0.92, 1.77)
**Hormonal Contraceptive Use**				
No (Reference)	1.00	1.00	1.00	1.00
Yes	0.28 (0.11, 0.72)	0.45 (0.17, 1.22)	2.49 (1.68, 3.70)	1.33 (0.86, 2.04)

Overall Model Fit: F (100, 371) = 12.92, *P*=0.0000.

Table S41: Unadjusted and adjusted relative risk ratio, of underweight and overweight/obesity among women of child-bearing age in Niger.

VARIABLES	UNDERWEIGHT		OVERWEIGHT/OBESITY	
	Unadjusted RRR (95% CI)	Adjusted RRR (95% CI)	Unadjusted RRR (95% CI)	Adjusted RRR (95% CI)
**Age**				
15-19 (Reference)	1.00	1.00	1.00	1.00
20-24	0.43 (0.32, 0.57)	0.67 (0.46, 0.98)	1.78 (1.16, 2.72)	1.50 (0.89, 2.53)
25-29	0.34 (0.25, 0.46)	0.54 (0.33, 0.89)	3.27 (2.20, 4.86)	3.67 (2.11, 6.36)
30-34	0.36 (0.24, 0.52)	0.52 (0.31, 0.87)	4.96 (3.31, 7.42)	6.72 (3.73, 12.1)
35-39	0.30 (0.21, 0.44)	0.42 (0.23, 0.78)	4.72 (3.20, 6.97)	6.45 (3.67, 11.3)
40-44	0.44 (0.30, 0.65)	0.59 (0.33, 1.05)	5.19 (3.39, 7.96)	7.68 (4.17, 14.1)
45-49	0.54 (0.32, 0.89)	0.72 (0.36, 1.47)	4.91 (3.14, 7.70)	7.68 (4.21, 14.0)
**Parity**				
0 (Reference)	1.00	1.00	1.00	1.00
1	0.42 (0.28, 0.62)	0.61 (0.37, 0.99)	1.72 (1.11, 2.65)	1.02 (0.56, 1.85)
2	0.29 (0.19, 0.45)	0.54 (0.31, 0.93)	2.11 (1.43, 3.14)	0.87 (0.46, 1.64)
3	0.37 (0.25, 0.56)	0.67 (0.37, 1.23)	1.87 (1.27, 2.76)	0.71 (0.40, 1.29)
4	0.36 (0.24, 0.53)	0.71 (0.39, 1.31)	2.20 (1.46, 3.30)	0.66 (0.35, 1.24)
5	0.30 (0.19, 0.47)	0.64 (0.34, 1.22)	1.91 (1.28, 2.84)	0.50 (0.27, 0.93)
6+	0.41 (0.31, 0.55)	0.82 (0.45, 1.50)	2.09 (1.49, 2.93)	0.51 (0.28, 0.92)
**Marital Status**				
Single (Reference)	1.00	1.00	1.00	1.00
Married	0.35 (0.27, 0.46)	0.57 (0.37, 0.88)	2.60 (1.72, 3.92)	3.93 (2.21, 6.99)
Formerly Married	0.39 (0.22, 0.69)	0.62 (0.31, 1.21)	3.15 (1.82, 5.44)	2.69 (1.30, 5.57)
**Residential Setting**				
Rural (Reference)	1.00	1.00	1.00	1.00
Urban	0.90 (0.70, 1.15)	1.04 (0.68, 1.59)	3.99 (3.24, 4.93)	1.57 (1.06, 2.33)
**Region**				
Niamey (Reference)	1.00	1.00	1.00	1.00
Agadez	1.16 (0.72, 1.87)	1.09 (0.65, 1.84)	0.53 (0.37, 0.75)	0.86 (0.55, 1.34)
Diffa	0.63 (0.35, 1.14)	0.73 (0.38, 1.41)	0.14 (0.09, 0.22)	0.29 (0.15, 0.56)
Dosso	0.97 (0.61, 1.55)	0.97 (0.55, 1.70)	0.38 (0.27, 0.52)	1.18 (0.78, 1.78)
Maradi	1.17 (0.75, 1.85)	1.24 (0.72, 2.16)	0.13 (0.09, 0.19)	0.36 (0.23, 0.56)
Tahoua	0.95 (0.58, 1.53)	0.87 (0.50, 1.52)	0.30 (0.22, 0.43)	0.97 (0.63, 1.47)
Tillaberi	0.99 (0.60, 1.64)	0.99 (0.55, 1.77)	0.35 (0.26, 0.48)	1.04 (0.69, 1.58)
Zinder	1.55 (0.99, 2.43)	1.69 (0.99, 2.87)	0.10 (0.07, 0.16)	0.26 (0.16, 0.43)
**Level of Education**				
No Education (Reference)	1.00	1.00	1.00	1.00
Primary	0.99 (0.71, 1.37)	0.84 (0.58, 1.23)	1.68 (1.31, 2.15)	1.22 (0.92, 1.62)
Secondary	1.53 (1.13, 2.08)	0.92 (0.58, 1.44)	1.89 (1.40, 2.55)	1.38 (0.90, 2.11)
Higher	0.30 (0.07, 1.36)	0.33 (0.06, 1.78)	4.63 (2.08, 10.3)	0.89 (0.34, 2.36)
**Wealth Quintile**				
Poorest (Reference)	1.00	1.00	1.00	1.00
Poorer	0.90 (0.65, 1.25)	0.94 (0.67, 1.33)	1.09 (0.72, 1.65)	1.04 (0.69, 1.57)
Middle	0.86 (0.63, 1.18)	0.88 (0.63, 1.22)	1.00 (0.65, 1.56)	0.95 (0.61, 1.49)
Richer	0.67 (0.47, 0.96)	0.69 (0.47, 1.01)	1.54 (1.02, 2.31)	1.46 (0.97, 2.20)
Richest	0.66 (0.48, 0.91)	0.50 (0.31, 0.80)	4.68 (3.23, 6.80)	3.31 (2.07, 5.30)
**Occupation**				
Not Working (Reference)	1.00	1.00	1.00	1.00
Nonmanual	0.83 (0.62, 1.12)	0.93 (0.68, 1.27)	1.69 (1.35, 2.10)	1.23 (0.95, 1.60)
Manual	0.60 (0.31, 1.16)	0.63 (0.31, 1.29)	2.32 (1.52, 3.55)	1.50 (0.88, 2.53)
Agricultural	1.04 (0.51, 2.14)	1.10 (0.53, 2.29)	1.40 (0.75, 2.61)	1.45 (0.74, 2.84)

VARIABLES	UNDERWEIGHT		OVERWEIGHT/OBESITY	
	Unadjusted RRR (95% CI)	Adjusted RRR (95% CI)	Unadjusted RRR (95% CI)	Adjusted RRR (95% CI)
**Media Exposure**				
Not Exposed (Reference)	1.00	1.00	1.00	1.00
Exposed to one media source	0.85 (0.66, 1.09)	0.91 (0.69, 1.19)	1.07 (0.83, 1.38)	0.85 (0.65, 1.11)
Exposed to two media sources	0.90 (0.68, 1.19)	0.97 (0.70, 1.33)	2.50 (1.90, 3.29)	1.08 (0.78, 1.51)
Exposed to three media sources	1.26 (0.81, 1.97)	1.20 (0.66, 2.19)	3.31 (2.21, 4.95)	1.02 (0.59, 1.77)
**Hormonal Contraceptives use**				
No (Reference)	1.00	1.00	1.00	1.00
Yes	0.44 (0.26, 0.74)	0.70 (0.42, 1.17)	2.69 (2.04, 3.54)	1.44 (1.07, 1.95)

Overall Model Fit: F (72, 385) = 10.21, *P*=0.0000.

Table S42: Unadjusted and adjusted relative risk ratio, of underweight and overweight/obesity among women of child-bearing age in Mali.

VARIABLES	UNDERWEIGHT		OVERWEIGHT/OBESITY	
	Unadjusted RRR (95% CI)	Adjusted RRR (95% CI)	Unadjusted RRR (95% CI)	Adjusted RRR (95% CI)
**Age**				
15-19 (Reference)	1.00	1.00	1.00	1.00
20-24	0.60 (0.44, 0.83)	0.77 (0.52, 1.13)	1.40 (0.94, 2.07)	1.65 (1.08, 2.50)
25-29	0.59 (0.44, 0.79)	0.80 (0.52, 1.24)	2.90 (2.07, 4.06)	4.30 (2.76, 6.71)
30-34	0.41 (0.28, 0.61)	0.51 (0.30, 0.89)	3.38 (2.31, 4.94)	4.90 (2.98, 8.07)
35-39	0.42 (0.28, 0.63)	0.54 (0.30, 0.95)	5.21 (3.53, 7.71)	7.84 (4.69, 13.1)
40-44	0.68 (0.46, 1.00)	0.89 (0.51, 1.56)	4.96 (3.50, 7.04)	7.61 (4.58, 12.6)
45-49	0.55 (0.34, 0.89)	0.70 (0.38, 1.32)	4.52 (3.12, 6.55)	7.84 (4.72, 13.0)
**Ethnicity**				
Bambara (Reference)	1.00	1.00	1.00	1.00
Malinke	0.85 (0.56, 1.30)	0.96 (0.60, 1.52)	1.01 (0.71, 1.42)	0.73 (0.51, 1.05)
Peulh	1.37 (0.99, 1.89)	1.31 (0.93, 1.84)	1.10 (0.81, 1.50)	0.99 (0.72, 1.37)
Sarakole/Soninke/Marka	1.19 (0.83, 1.72)	1.34 (0.87, 2.06)	1.25 (0.91, 1.72)	1.03 (0.71, 1.49)
Dogon	0.82 (0.54, 1.24)	0.53 (0.34, 0.83)	0.87 (0.60, 1.26)	1.03 (0.63, 1.68)
Sénoufo/Minianka	0.86 (0.55, 1.34)	0.82 (0.52, 1.29)	0.81 (0.53, 1.23)	0.84 (0.54, 1.29)
Others	0.71 (0.50, 1.02)	0.71 (0.47, 1.06)	1.31 (0.98, 1.76)	1.15 (0.83, 1.59)
**Religion**				
Islam (Reference)	1.00	1.00	1.00	1.00
Catholics	0.50 (0.20, 1.23)	0.61 (0.23, 1.59)	0.85 (0.44, 1.67)	0.85 (0.43, 1.66)
Other Christians	0.47 (0.18, 1.21)	0.57 (0.22, 1.50)	0.52 (0.29, 0.92)	0.70 (0.40, 1.24)
Traditionalists	0.22 (0.05, 1.04)	0.25 (0.05, 1.22)	0.29 (0.08, 1.02)	0.55 (0.17, 1.82)
No Religion	1.33 (0.72, 2.43)	1.40 (0.74, 2.68)	0.46 (0.20, 1.02)	0.89 (0.37, 2.09)
**Parity**				
0 (Reference)	1.00	1.00	1.00	1.00
1	0.88 (0.64, 1.22)	1.08 (0.72, 1.60)	1.10 (0.77, 1.56)	0.82 (0.52, 1.30)
2	0.40 (0.28, 0.57)	0.52 (0.32, 0.84)	1.55 (1.12, 2.15)	0.82 (0.53, 1.28)
3	0.37 (0.24, 0.58)	0.46 (0.25, 0.82)	1.52 (1.06, 2.19)	0.66 (0.41, 1.07)
4	0.64 (0.44, 0.93)	0.82 (0.47, 1.43)	1.93 (1.40, 2.65)	0.80 (0.49, 1.29)
5	0.63 (0.43, 0.92)	0.88 (0.51, 1.50)	1.70 (1.20, 2.40)	0.66 (0.40, 1.10)
6+	0.54 (0.41, 0.73)	0.75 (0.45, 1.26)	2.27 (1.67, 3.08)	0.86 (0.54, 1.37)
**Marital Status**				
Single (Reference)	1.00	1.00	1.00	1.00
Married	0.56 (0.45, 0.70)	0.75 (0.51, 1.10)	2.01 (1.45, 2.78)	1.42 (0.85, 2.38)
Formerly Married	0.66 (0.34, 1.29)	0.88 (0.41, 1.87)	2.67 (1.50, 4.78)	1.20 (0.59, 2.45)
**Residential Setting**				
Rural (Reference)	1.00	1.00	1.00	1.00
Urban	0.99 (0.79, 1.25)	0.98 (0.63, 1.52)	3.25 (2.67, 3.96)	1.55 (1.08, 2.23)
**Region**				
Bamako (Reference)	1.00	1.00	1.00	1.00
Kayes	0.89 (0.59, 1.35)	0.74 (0.39, 1.40)	0.36 (0.26, 0.49)	0.87 (0.60, 1.27)
Sikasso	0.92 (0.66, 1.29)	0.91 (0.58, 1.43)	0.30 (0.21, 0.42)	0.71 (0.48, 1.04)
Koulikoro	1.08 (0.79, 1.49)	1.20 (0.79, 1.83)	0.33 (0.25, 0.44)	0.74 (0.52, 1.06)
Segou	0.84 (0.56, 1.24)	0.94 (0.60, 1.50)	0.33 (0.24, 0.46)	0.89 (0.62, 1.27)
Mopti	1.25 (0.89, 1.75)	1.64 (1.01, 2.66)	0.36 (0.26, 0.49)	1.06 (0.63, 1.77)
**Level of Education**				
No Education (Reference)	1.00	1.00	1.00	1.00
Primary	0.89 (0.61, 1.31)	0.75 (0.52, 1.10)	1.93 (1.52, 2.46)	1.62 (1.23, 2.15)
Secondary	0.98 (0.74, 1.30)	0.57 (0.38, 0.84)	1.45 (1.14, 1.84)	1.37 (0.95, 1.99)
Higher	1.39 (0.62, 3.11)	0.83 (0.28, 2.45)	1.34 (0.72, 2.48)	0.73 (0.38, 1.38)

VARIABLES	UNDERWEIGHT		OVERWEIGHT/OBESITY	
	Unadjusted RRR (95% CI)	Adjusted RRR (95% CI)	Unadjusted RRR (95% CI)	Adjusted RRR (95% CI)
**Wealth Quintile**				
Poorest (Reference)	1.00	1.00	1.00	1.00
Poorer	0.97 (0.71, 1.34)	1.00 (0.72, 1.40)	0.91 (0.65, 1.28)	0.89 (0.63, 1.27)
Middle	1.05 (0.76, 1.46)	1.06 (0.76, 1.48)	0.93 (0.65, 1.33)	0.92 (0.62, 1.35)
Richer	1.02 (0.72, 1.44)	1.15 (0.76, 1.73)	1.72 (1.23, 2.40)	1.57 (1.06, 2.31)
Richest	0.96 (0.71, 1.29)	0.99 (0.58, 1.69)	3.84 (2.86, 5.16)	2.96 (1.85, 4.76)
**Occupation**				
Not Working (Reference)	1.00	1.00	1.00	1.00
Nonmanual	0.53 (0.38, 0.74)	0.57 (0.41, 0.80)	1.90 (1.48, 2.43)	1.17 (0.90, 1.50)
Agricultural	0.82 (0.60, 1.11)	0.87 (0.63, 1.20)	0.69 (0.49, 0.96)	0.89 (0.61, 1.31)
Don’t Know	0.83 (0.59, 1.17)	0.89 (0.63, 1.26)	1.49 (1.17, 1.90)	1.20 (0.93, 1.56)
**Media Exposure**				
Not Exposed (Reference)	1.00	1.00	1.00	1.00
Exposed to one media source	0.96 (0.73, 1.27)	0.97 (0.74, 1.27)	1.14 (0.89, 1.47)	1.02 (0.78, 1.33)
Exposed to two media sources	0.87 (0.66, 1.15)	0.88 (0.65, 1.18)	1.84 (1.44, 2.37)	1.05 (0.79, 1.39)
Exposed to three media sources	1.04 (0.70, 1.56)	1.09 (0.60, 1.99)	2.30 (1.69, 3.13)	0.91 (0.61, 1.37)
**Hormonal Contraceptive Use**				
No (Reference)	1.00	1.00	1.00	1.00
Yes	0.85 (0.60, 1.19)	1.12 (0.79, 1.59)	1.67 (1.30, 2.13)	0.99 (0.76, 1.28)

Overall Model Fit: F (88, 311) = 6.29, *P*=0.0000.

Table S43: Unadjusted and adjusted relative risk ratio, of underweight and overweight/obesity among women of child-bearing age in Gambia.

VARIABLES	UNDERWEIGHT		OVERWEIGHT/OBESITY	
	Unadjusted RRR (95% CI)	Adjusted RRR (95% CI)	Unadjusted RRR (95% CI)	Adjusted RRR (95% CI)
**Age**				
15-19 (Reference)	1.00	1.00	1.00	1.00
20-24	0.61 (0.44, 0.85)	0.92 (0.66, 1.30)	1.44 (1.00, 2.08)	1.44 (0.96, 2.16)
25-29	0.55 (0.37, 0.82)	1.09 (0.65, 1.83)	3.10 (2.06, 4.68)	3.15 (1.89, 5.27)
30-34	0.45 (0.31, 0.65)	1.02 (0.61, 1.69)	2.75 (1.83, 4.14)	3.28 (1.89, 5.68)
35-39	0.38 (0.23, 0.63)	0.92 (0.48, 1.79)	4.97 (3.30, 7.50)	6.57 (3.64, 11.9)
40-44	0.50 (0.30, 0.84)	1.30 (0.64, 2.66)	6.13 (3.88, 9.69)	8.71 (4.55, 16.7)
45-49	0.41 (0.24, 0.69)	0.99 (0.47, 2.07)	5.87 (3.63, 9.48)	8.52 (4.51, 16.1)
**Ethnicity**				
Mandinka/Jahanka (Reference)	1.00	1.00	1.00	1.00
Wollof	0.98 (0.63, 1.50)	0.95 (0.61, 1.47)	1.09 (0.77, 1.55)	1.12 (0.74, 1.70)
Jola/Karoninka	0.61 (0.39, 0.98)	0.63 (0.37, 1.06)	1.35 (0.93, 1.97)	0.96 (0.62, 1.50)
Fula/Tukulur/Lorobo	1.35 (1.00, 1.82)	1.34 (1.00, 1.79)	1.02 (0.78, 1.34)	1.14 (0.86, 1.52)
Serere	1.00 (0.49, 2.02)	1.15 (0.57, 2.33)	1.83 (1.08, 3.11)	1.14 (0.66, 1.99)
Serahuleh	0.86 (0.53, 1.38)	0.99 (0.58, 1.70)	1.12 (0.74, 1.67)	1.15 (0.76, 1.75)
Others	1.07 (0.62, 1.84)	1.03 (0.46, 2.30)	1.27 (0.76, 2.14)	1.10 (0.58, 2.07)
Non-Gambian	0.53 (0.26, 1.09)	0.64 (0.29, 1.40)	1.60 (0.97, 2.63)	1.43 (0.85, 2.41)
**Religion**				
Islam (Reference)	1.00	1.00	1.00	1.00
Christians	0.93 (0.54, 1.61)	1.12 (0.49, 2.57)	1.14 (0.68, 1.90)	0.61 (0.33, 1.13)
**Parity**				
0 (Reference)	1.00	1.00	1.00	1.00
1	0.64 (0.45, 0.93)	1.15 (0.73, 1.81)	1.41 (1.00, 1.98)	1.13 (0.68, 1.88)
2	0.57 (0.38, 0.84)	1.12 (0.62, 2.03)	1.34 (0.93, 1.92)	0.90 (0.49, 1.67)
3	0.57 (0.37, 0.89)	1.00 (0.55, 1.81)	2.35 (1.55, 3.55)	1.47 (0.79, 2.75)
4	0.68 (0.44, 1.05)	1.15 (0.59, 2.26)	3.05 (2.01, 4.62)	1.60 (0.92, 2.76)
5	0.36 (0.20, 0.64)	0.59 (0.29, 1.21)	1.88 (1.17, 3.03)	0.85 (0.43, 1.69)
6+	0.44 (0.33, 0.60)	0.65 (0.35, 1.21)	2.46 (1.74, 3.47)	0.98 (0.52, 1.86)
**Marital Status**				
Single (Reference)	1.00	1.00	1.00	1.00
Married	0.48 (0.39, 0.60)	0.45 (0.31, 0.65)	1.67 (1.26, 2.22)	0.69 (0.42, 1.12)
Formerly Married	0.24 (0.13, 0.46)	0.25 (0.12, 0.49)	3.32 (2.33, 4.73)	0.95 (0.56, 1.61)
**Residential Setting**				
Rural (Reference)	1.00	1.00	1.00	1.00
Urban	0.78 (0.61, 1.00)	1.23 (0.81, 1.88)	1.79 (1.46, 2.19)	0.76 (0.48, 1.20)
**Region**				
Banjul (Reference)	1.00	1.00	1.00	1.00
Kanifing	0.80 (0.53, 1.21)	0.77 (0.49, 1.20)	0.72 (0.54, 0.96)	0.85 (0.62, 1.18)
Brikama	1.05 (0.69, 1.60)	0.99 (0.59, 1.64)	0.42 (0.31, 0.56)	0.56 (0.40, 0.78)
Mansakonko	1.50 (0.99, 2.28)	1.22 (0.68, 2.21)	0.36 (0.25, 0.53)	0.58 (0.34, 0.98)
Kerewan	1.44 (0.99, 2.08)	1.10 (0.64, 1.90)	0.30 (0.21, 0.42)	0.50 (0.29, 0.87)
Kuntaur	1.16 (0.77, 1.76)	0.88 (0.48, 1.61)	0.27 (0.18, 0.41)	0.44 (0.24, 0.81)
Janjanbureh	1.77 (1.14, 2.73)	1.30 (0.71, 2.39)	0.41 (0.30, 0.58)	0.71 (0.43, 1.18)
Base	1.03 (0.67, 1.58)	0.79 (0.41, 1.50)	0.27 (0.18, 0.40)	0.41 (0.23, 0.76)
**Level of Education**				
No Education (Reference)	1.00	1.00	1.00	1.00
Primary	0.88 (0.63, 1.22)	0.70 (0.49, 1.01)	0.83 (0.64, 1.09)	1.18 (0.86, 1.62)
Secondary	1.32 (1.01, 1.72)	0.95 (0.67, 1.35)	0.90 (0.72, 1.14)	1.24 (0.85, 1.82)
Higher	0.51 (0.27, 0.96)	0.42 (0.20, 0.90)	1.26 (0.80, 1.99)	1.24 (0.69, 2.24)

VARIABLES	UNDERWEIGHT		OVERWEIGHT/OBESITY	
	Unadjusted RRR (95% CI)	Adjusted RRR (95% CI)	Unadjusted RRR (95% CI)	Adjusted RRR (95% CI)
**Wealth Quintile**				
Poorest (Reference)	1.00	1.00	1.00	1.00
Poorer	1.22 (0.92, 1.61)	1.23 (0.92, 1.64)	1.01 (0.68, 1.49)	0.99 (0.66, 1.48)
Middle	1.14 (0.81, 1.59)	1.13 (0.80, 1.59)	1.55 (1.14, 2.12)	1.64 (1.15, 2.34)
Richer	0.80 (0.56, 1.14)	0.79 (0.48, 1.29)	2.01 (1.47, 2.74)	1.88 (1.25, 2.83)
Richest	0.70 (0.49, 1.01)	0.67 (0.38, 1.19)	2.18 (1.59, 2.97)	1.95 (1.20, 3.16)
**Occupation**				
Not Working (Reference)	1.00	1.00	1.00	1.00
Nonmanual	0.59 (0.43, 0.82)	0.86 (0.60, 1.24)	2.32 (1.82, 2.95)	1.41 (1.07, 1.87)
Manual	0.38 (0.13, 1.06)	0.53 (0.18, 1.58)	2.77 (1.70, 4.52)	1.88 (1.16, 3.04)
Agricultural	0.91 (0.71, 1.18)	1.13 (0.82, 1.57)	0.93 (0.74, 1.17)	0.88 (0.66, 1.17)
**Media Exposure**				
Not Exposed (Reference)	1.00	1.00	1.00	1.00
Exposed to one media source	0.81 (0.58, 1.14)	0.88 (0.63, 1.24)	1.09 (0.68, 1.73)	1.11 (0.69, 1.79)
Exposed to two media sources	0.68 (0.46, 1.00)	0.77 (0.52, 1.12)	1.34 (0.84, 2.14)	1.12 (0.66, 1.89)
Exposed to three media sources	0.78 (0.50, 1.22)	0.96 (0.56, 1.65)	1.54 (0.94, 2.50)	0.96 (0.50, 1.87)
**Hormonal Contraceptive Use**				
No (Reference)	1.00	1.00	1.00	1.00
Yes	0.31 (0.15, 0.64)	0.48 (0.22, 1.03)	1.57 (1.09, 2.26)	1.03 (0.72, 1.48)

Overall Model Fit: F (88, 180) = 8.63, *P*=0.0000.

Table S44: Unadjusted and adjusted relative risk ratio, of underweight and overweight/obesity among women of child-bearing age in Uganda.

VARIABLES	UNDERWEIGHT		OVERWEIGHT/OBESITY	
	Unadjusted RRR (95% CI)	Adjusted RRR (95% CI)	Unadjusted RRR (95% CI)	Adjusted RRR (95% CI)
**Age**				
15-19 (Reference)	1.00	1.00	1.00	1.00
20-24	0.77 (0.46, 1.27)	0.81 (0.45, 1.43)	1.20 (0.74, 1.95)	0.93 (0.53, 1.62)
25-29	0.64 (0.42, 0.99)	0.67 (0.34, 1.30)	1.45 (0.95, 2.20)	1.27 (0.72, 2.25)
30-34	0.77 (0.45, 1.30)	0.85 (0.38, 1.89)	2.39 (1.59, 3.58)	2.55 (1.34, 4.84)
35-39	0.85 (0.49, 1.47)	0.83 (0.34, 2.05)	2.25 (1.45, 3.48)	3.10 (1.48, 6.52)
40-44	1.22 (0.72, 2.07)	1.24 (0.55, 2.78)	2.53 (1.53, 4.19)	4.04 (1.89, 8.64)
45-49	1.13 (0.67, 1.90)	0.96 (0.35, 2.63)	3.40 (2.11, 5.47)	4.99 (2.31, 10.8)
**Ethnicity**				
Baganda (Reference)	1.00	1.00	1.00	1.00
Banyankole	0.64 (0.25, 1.62)	0.87 (0.33, 2.30)	0.59 (0.36, 0.97)	0.99 (0.52, 1.86)
Basoga	0.96 (0.44, 2.09)	0.55 (0.22, 1.35)	0.41 (0.24, 0.70)	0.55 (0.25, 1.21)
Bakiga	1.33 (0.63, 2.80)	2.06 (0.84, 5.04)	0.80 (0.47, 1.35)	1.21 (0.60, 2.41)
Iteso	3.63 (1.88, 7.01)	2.01 (0.82, 4.97)	0.33 (0.18, 0.62)	0.94 (0.39, 2.29)
Others	2.14 (1.27, 3.59)	1.45 (0.74, 2.85)	0.35 (0.24, 0.51)	0.66 (0.40, 1.09)
**Religion**				
Catholics (Reference)	1.00	1.00	1.00	1.00
Other Christians	0.84 (0.61, 1.15)	1.03 (0.72, 1.49)	1.31 (0.99, 1.72)	1.13 (0.83, 1.53)
Islam	0.61 (0.37, 0.99)	0.76 (0.45, 1.28)	1.67 (1.13, 2.48)	1.29 (0.82, 2.02)
Others	0.68 (0.15, 3.06)	0.88 (0.16, 4.89)	1.88 (0.54, 6.58)	1.30 (0.36, 4.76)
**Parity**				
0 (Reference)	1.00	1.00	1.00	1.00
1	0.59 (0.33, 1.05)	0.55 (0.22, 1.34)	1.73 (1.12, 2.67)	1.07 (0.63, 1.79)
2	0.87 (0.50, 1.49)	0.76 (0.26, 2.19)	1.57 (0.98, 2.51)	0.75 (0.39, 1.44)
3	0.82 (0.44, 1.51)	0.70 (0.21, 2.28)	1.10 (0.68, 1.78)	0.42 (0.21, 0.86)
4	0.94 (0.54, 1.63)	0.73 (0.23, 2.29)	1.91 (1.24, 2.96)	0.77 (0.36, 1.68)
5	0.94 (0.52, 1.70)	0.74 (0.23, 2.38)	1.86 (1.13, 3.05)	0.54 (0.24, 1.22)
6+	0.93 (0.63, 1.37)	0.56 (0.18, 1.71)	1.40 (0.99, 1.98)	0.39 (0.19, 0.81)
**Marital Status**				
Single (Reference)	1.00	1.00	1.00	1.00
Married	0.88 (0.63, 1.22)	1.10 (0.42, 2.87)	1.58 (1.17, 2.14)	2.03 (1.11, 3.70)
Formerly Married	1.21 (0.76, 1.92)	1.51 (0.57, 3.97)	1.80 (1.20, 2.69)	1.72 (0.85, 3.47)
**Residential Setting**				
Rural (Reference)	1.00	1.00	1.00	1.00
Urban	0.74 (0.52, 1.05)	1.10 (0.66, 1.82)	3.04 (2.33, 3.96)	1.57 (1.00, 2.46)
**Region**				
Kampala (Reference)	1.00	1.00	1.00	1.00
Central 1	0.71 (0.27, 1.86)	0.46 (0.17, 1.26)	0.44 (0.25, 0.76)	0.86 (0.47, 1.57)
Central 2	0.79 (0.37, 1.69)	0.48 (0.21, 1.12)	0.36 (0.21, 0.61)	0.70 (0.39, 1.23)
East central	1.15 (0.60, 2.20)	0.99 (0.39, 2.46)	0.29 (0.17, 0.48)	0.86 (0.40, 1.86)
Eastern	1.95 (1.08, 3.51)	0.61 (0.24, 1.52)	0.17 (0.10, 0.30)	0.65 (0.28, 1.50)
North	1.45 (0.78, 2.70)	0.45 (0.17, 1.16)	0.12 (0.07, 0.22)	0.51 (0.24, 1.05)
Karamoja	3.32 (1.57, 7.04)	0.90 (0.29, 2.82)	0.02 (0.01, 0.09)	0.10 (0.02, 0.50)
West Nile	1.84 (1.00, 3.40)	0.57 (0.23, 1.43)	0.07 (0.04, 0.14)	0.25 (0.11, 0.57)
Western	0.76 (0.38, 1.52)	0.34 (0.14, 0.84)	0.42 (0.26, 0.68)	1.30 (0.72, 2.37)
South west	0.41 (0.18, 0.95)	0.19 (0.07, 0.50)	0.38 (0.24, 0.60)	1.01 (0.49, 2.10)

VARIABLES	UNDERWEIGHT		OVERWEIGHT/OBESITY	
	Unadjusted RRR (95% CI)	Adjusted RRR (95% CI)	Unadjusted RRR (95% CI)	Adjusted RRR (95% CI)
**Level of Education**				
No Education (Reference)	1.00	1.00	1.00	1.00
Primary	0.58 (0.37, 0.91)	0.64 (0.38, 1.06)	0.89 (0.57, 1.39)	0.84 (0.50, 1.43)
Secondary	0.28 (0.16, 0.50)	0.43 (0.21, 0.89)	1.20 (0.75, 1.92)	0.63 (0.33, 1.20)
Higher	0.41 (0.18, 0.90)	0.73 (0.29, 1.89)	3.18 (1.74, 5.84)	0.96 (0.47, 1.96)
**Wealth Quintile**				
Poorest (Reference)	1.00	1.00	1.00	1.00
Poorer	0.80 (0.52, 1.24)	1.04 (0.63, 1.72)	1.61 (0.78, 3.30)	1.21 (0.58, 2.54)
Middle	0.37 (0.22, 0.62)	0.58 (0.31, 1.09)	2.29 (1.23, 4.28)	1.39 (0.72, 2.68)
Richer	0.36 (0.22, 0.59)	0.61 (0.32, 1.16)	4.57 (2.52, 8.29)	2.66 (1.37, 5.16)
Richest	0.31 (0.19, 0.51)	0.47 (0.19, 1.16)	7.61 (4.28, 13.5)	3.12 (1.43, 6.81)
**Occupation**				
Not Working (Reference)	1.00	1.00	1.00	1.00
Nonmanual	0.87 (0.57, 1.32)	0.93 (0.58, 1.49)	2.28 (1.64, 3.17)	1.39 (0.94, 2.05)
Agricultural	0.96 (0.66, 1.39)	0.86 (0.57, 1.30)	0.93 (0.66, 1.32)	0.79 (0.53, 1.17)
**Media Exposure**				
Not Exposed (Reference)	1.00	1.00	1.00	1.00
Exposed to one media source	0.69 (0.46, 1.02)	0.91 (0.60, 1.39)	1.63 (0.99, 2.68)	1.27 (0.76, 2.14)
Exposed to two media sources	0.56 (0.35, 0.90)	0.83 (0.48, 1.45)	2.28 (1.31, 3.96)	1.25 (0.69, 2.24)
Exposed to three media sources	0.36 (0.20, 0.66)	0.65 (0.30, 1.41)	4.13 (2.39, 7.13)	1.36 (0.74, 2.52)
**Hormonal Contraceptive Use**				
No (Reference)	1.00	1.00	1.00	1.00
Yes	0.44 (0.27, 0.70)	0.58 (0.34, 0.97)	1.06 (0.78, 1.45)	0.82 (0.56, 1.20)

Overall Model Fit: F (90, 304) = 5.07, *P*=0.0000.

Table S45: Unadjusted and adjusted relative risk ratio, of underweight and overweight/obesity among women of child-bearing age in Guinea.

VARIABLES	UNDERWEIGHT		OVERWEIGHT/OBESITY	
	Unadjusted RRR (95% CI)	Adjusted RRR (95% CI)	Unadjusted RRR (95% CI)	Adjusted RRR (95% CI)
**Age**				
15-19 (Reference)	1.00	1.00	1.00	1.00
20-24	0.66 (0.48, 0.91)	0.86 (0.59, 1.25)	1.82 (1.33, 2.48)	2.01 (1.39, 2.91)
25-29	0.47 (0.32, 0.69)	0.69 (0.44, 1.07)	2.56 (1.73, 3.77)	3.59 (2.06, 6.26)
30-34	0.49 (0.33, 0.74)	0.82 (0.48, 1.40)	2.88 (1.95, 4.24)	4.42 (2.38, 8.20)
35-39	0.52 (0.36, 0.76)	0.91 (0.55, 1.50)	3.79 (2.69, 5.35)	6.62 (3.73, 11.73)
40-44	0.72 (0.49, 1.05)	1.23 (0.71, 2.13)	3.86 (2.66, 5.62)	7.74 (4.24, 14.13)
45-49	0.59 (0.38, 0.90)	0.90 (0.50, 1.62)	3.98 (2.75, 5.75)	9.20 (4.82, 17.56)
**Ethnicity**				
Peulh (Reference)	1.00	1.00	1.00	1.00
Soussou	0.54 (0.39, 0.76)	0.63 (0.42, 0.96)	2.27 (1.67, 3.10)	1.80 (1.32, 2.46)
Malinké	0.78 (0.60, 1.01)	0.83 (0.57, 1.22)	1.41 (1.11, 1.81)	1.54 (1.10, 2.15)
Kissi	0.54 (0.31, 0.94)	0.22 (0.08, 0.66)	0.87 (0.54, 1.41)	1.95 (0.81, 4.69)
Toma	0.31 (0.14, 0.68)	0.08 (0.02, 0.41)	0.88 (0.27, 2.84)	1.84 (0.39, 8.64)
Guerzé	0.31 (0.12, 0.83)	0.14 (0.04, 0.45)	1.23 (0.85, 1.79)	1.64 (0.74, 3.66)
Autre/Manquant	0.66 (0.41, 1.06)	0.50 (0.24, 1.06)	1.78 (1.00, 3.19)	1.83 (0.99, 3.41)
**Religion**				
Other Christians (Reference)	1.00	1.00	1.00	1.00
Islam	1.69 (0.96, 2.96)	0.35 (0.14, 0.84)	1.42 (1.01, 2.01)	1.48 (0.71, 3.07)
No Religion	1.01 (0.39, 2.62)	1.63 (0.44, 6.09)	1.03 (0.52, 2.05)	1.66 (0.76, 3.66)
**Parity**				
0 (Reference)	1.00	1.00	1.00	1.00
1	0.69 (0.48, 0.97)	0.80 (0.51, 1.24)	1.06 (0.76, 1.49)	0.76 (0.48, 1.21)
2	0.68 (0.47, 1.00)	0.83 (0.51, 1.36)	1.39 (1.03, 1.87)	0.77 (0.49, 1.23)
3	0.56 (0.36, 0.86)	0.69 (0.40, 1.19)	1.35 (0.96, 1.89)	0.56 (0.33, 0.95)
4	0.62 (0.40, 0.95)	0.74 (0.40, 1.35)	1.28 (0.84, 1.96)	0.50 (0.27, 0.92)
5	0.37 (0.21, 0.67)	0.40 (0.19, 0.83)	1.97 (1.35, 2.86)	0.79 (0.43, 1.45)
6+	0.62 (0.46, 0.83)	0.56 (0.34, 0.95)	1.06 (0.76, 1.49)	0.52 (0.30, 0.93)
**Marital Status**				
Single (Reference)	1.00	1.00	1.00	1.00
Married	0.65 (0.51, 0.83)	0.76 (0.53, 1.09)	1.56 (1.15, 2.12)	1.82 (1.12, 2.96)
Formerly Married	0.61 (0.30, 1.22)	0.76 (0.36, 1.60)	1.45 (0.82, 2.55)	1.02 (0.50, 2.09)
**Residential Setting**				
Rural (Reference)	1.00	1.00	1.00	1.00
Urban	0.91 (0.69, 1.19)	1.11 (0.68, 1.80)	3.39 (2.70, 4.25)	1.29 (0.92, 1.81)
**Region**				
Conakry (Reference)	1.00	1.00	1.00	1.00
Boké	0.97 (0.63, 1.48)	0.98 (0.56, 1.70)	0.51 (0.36, 0.73)	0.83 (0.56, 1.24)
Faranah	0.95 (0.59, 1.52)	0.90 (0.43, 1.86)	0.25 (0.16, 0.37)	0.60 (0.36, 0.99)
Kankan	1.12 (0.71, 1.77)	1.07 (0.52, 2.22)	0.31 (0.20, 0.49)	0.75 (0.45, 1.25)
Kindia	0.74 (0.46, 1.19)	0.90 (0.51, 1.60)	0.35 (0.23, 0.52)	0.49 (0.30, 0.79)
Labé	1.23 (0.83, 1.83)	1.00 (0.55, 1.82)	0.20 (0.13, 0.30)	0.61 (0.35, 1.05)
Mamou	1.63 (1.06, 2.51)	1.44 (0.79, 2.62)	0.25 (0.17, 0.37)	0.61 (0.36, 1.02)
N'Zérékoré	0.59 (0.36, 0.98)	0.67 (0.30, 1.49)	0.32 (0.23, 0.44)	0.74 (0.48, 1.15)
**Level of Education**				
No Education (Reference)	1.00	1.00	1.00	1.00
Primary	1.10 (0.81, 1.49)	1.02 (0.74, 1.42)	1.20 (0.93, 1.55)	1.07 (0.81, 1.42)
Secondary	1.12 (0.79, 1.60)	0.94 (0.63, 1.41)	1.84 (1.37, 2.48)	1.44 (1.03, 2.01)
Higher	0.71 (0.34, 1.48)	0.63 (0.24, 1.66)	1.66 (0.98, 2.84)	0.69 (0.34, 1.37)

VARIABLES	UNDERWEIGHT		OVERWEIGHT/OBESITY	
	Unadjusted RRR (95% CI)	Adjusted RRR (95% CI)	Unadjusted RRR (95% CI)	Adjusted RRR (95% CI)
**Wealth Quintile**				
Poorest (Reference)	1.00	1.00	1.00	1.00
Poorer	0.77 (0.58, 1.03)	1.04 (0.79, 1.37)	1.35 (0.80, 2.27)	1.18 (0.71, 1.97)
Middle	0.61 (0.44, 0.85)	0.73 (0.52, 1.01)	2.87 (1.81, 4.56)	2.35 (1.50, 3.69)
Richer	0.56 (0.41, 0.76)	0.62 (0.42, 0.92)	4.30 (2.70, 6.84)	2.58 (1.53, 4.35)
Richest	0.75 (0.53, 1.05)	0.88 (0.47, 1.65)	7.34 (4.62, 11.7)	2.65 (1.47, 4.77)
**Occupation**				
Not Working (Reference)	1.00	1.00	1.00	1.00
Nonmanual	0.52 (0.37, 0.74)	0.77 (0.54, 1.11)	1.94 (1.51, 2.49)	0.98 (0.72, 1.34)
Manual	0.66 (0.42, 1.05)	0.71 (0.44, 1.15)	0.74 (0.44, 1.24)	0.63 (0.37, 1.08)
Agricultural	0.86 (0.65, 1.12)	1.10 (0.80, 1.51)	0.49 (0.36, 0.65)	0.61 (0.43, 0.86)
**Media Exposure**				
Not Exposed (Reference)	1.00	1.00	1.00	1.00
Exposed to one media source	0.95 (0.73, 1.23)	0.88 (0.67, 1.15)	1.43 (1.06, 1.93)	1.25 (0.92, 1.70)
Exposed to two media sources	0.77 (0.57, 1.02)	0.74 (0.53, 1.03)	3.39 (2.63, 4.39)	1.72 (1.21, 2.44)
Exposed to three media sources	1.01 (0.62, 1.66)	0.83 (0.44, 1.57)	3.55 (2.18, 5.80)	1.57 (0.78, 3.16)
**Hormonal Contraceptive Use**				
No (Reference)	1.00	1.00	1.00	1.00
Yes	0.28 (0.11, 0.71)	0.35 (0.14, 0.91)	1.81 (1.13, 2.89)	1.22 (0.73, 2.03)

Overall Model Fit: F (88, 198) = 7.39, *P*=0.0000.

Table S46: Unadjusted and adjusted relative risk ratio, of underweight and overweight/obesity among women of child-bearing age in Senegal.

VARIABLES	UNDERWEIGHT		OVERWEIGHT/OBESITY	
	Unadjusted RRR (95% CI)	Adjusted RRR (95% CI)	Unadjusted RRR (95% CI)	Adjusted RRR (95% CI)
**Age**				
15-19 (Reference)	1.00	1.00	1.00	1.00
20-24	0.83 (0.64, 1.07)	1.05 (0.80, 1.39)	2.07 (1.30, 3.28)	1.93 (1.19, 3.12)
25-29	0.58 (0.42, 0.79)	0.88 (0.61, 1.27)	2.95 (1.74, 5.01)	2.60 (1.42, 4.76)
30-34	0.50 (0.36, 0.68)	0.84 (0.56, 1.24)	4.19 (2.87, 6.13)	3.48 (2.14, 5.65)
35-39	0.39 (0.27, 0.58)	0.71 (0.44, 1.14)	5.48 (3.66, 8.20)	4.97 (2.99, 8.25)
40-44	0.28 (0.18, 0.43)	0.57 (0.34, 0.97)	6.65 (4.29, 10.3)	5.39 (3.11, 9.32)
45-49	0.36 (0.23, 0.55)	0.70 (0.43, 1.15)	7.99 (4.81, 13.3)	6.63 (3.53, 12.4)
**Ethnicity**				
Wolof (Reference)	1.00	1.00	1.00	1.00
Poular	1.01 (0.81, 1.27)	1.05 (0.78, 1.42)	0.74 (0.59, 0.93)	0.72 (0.54, 0.98)
Serer	0.76 (0.55, 1.04)	0.86 (0.61, 1.23)	0.60 (0.45, 0.80)	0.65 (0.46, 0.92)
Mandingue	0.52 (0.34, 0.79)	0.53 (0.32, 0.89)	1.18 (0.71, 1.96)	1.12 (0.60, 2.07)
Diola	0.34 (0.16, 0.73)	0.45 (0.16, 1.29)	0.74 (0.45, 1.20)	0.47 (0.23, 0.95)
Soninke	0.64 (0.35, 1.18)	0.63 (0.31, 1.29)	1.08 (0.62, 1.86)	1.08 (0.63, 1.84)
Not a Senegalese	0.48 (0.17, 1.39)	0.52 (0.20, 1.39)	0.96 (0.49, 1.86)	0.97 (0.48, 1.97)
Others	0.67 (0.44, 1.03)	0.76 (0.48, 1.23)	0.96 (0.56, 1.65)	0.77 (0.42, 1.40)
**Religion**				
Islam (Reference)	1.00	1.00	1.00	1.00
Others	0.46 (0.30, 0.70)	0.54 (0.35, 0.84)	0.79 (0.48, 1.30)	0.81 (0.41, 1.58)
**Parity**				
0 (Reference)	1.00	1.00	1.00	1.00
1	0.75 (0.55, 1.03)	1.01 (0.70, 1.44)	1.86 (1.37, 2.52)	1.22 (0.78, 1.91)
2	0.69 (0.51, 0.93)	0.95 (0.68, 1.33)	1.46 (0.99, 2.16)	0.85 (0.50, 1.44)
3	0.62 (0.44, 0.87)	0.87 (0.57, 1.33)	2.34 (1.66, 3.30)	1.27 (0.73, 2.22)
4	0.51 (0.36, 0.72)	0.72 (0.44, 1.20)	2.00 (1.42, 2.82)	0.97 (0.55, 1.71)
5	0.43 (0.28, 0.67)	0.66 (0.38, 1.16)	2.54 (1.58, 4.09)	1.16 (0.61, 2.20)
6+	0.37 (0.28, 0.49)	0.58 (0.38, 0.89)	2.68 (2.00, 3.59)	1.33 (0.80, 2.21)
**Marital Status**				
Single (Reference)	1.00	1.00	1.00	1.00
Married	0.60 (0.50, 0.72)	0.67 (0.50, 0.89)	2.13 (1.64, 2.76)	1.20 (0.75, 1.92)
Formerly Married	0.45 (0.28, 0.71)	0.53 (0.30, 0.93)	2.56 (1.72, 3.81)	1.01 (0.54, 1.87)
**Residential Setting**				
Rural (Reference)	1.00	1.00	1.00	1.00
Urban	0.80 (0.64, 1.01)	0.88 (0.68, 1.13)	2.35 (1.91, 2.89)	1.60 (1.22, 2.09)
**Region**				
Dakar (Reference)	1.00	1.00	1.00	1.00
Ziguinchor	0.57 (0.35, 0.93)	0.63 (0.29, 1.33)	0.66 (0.43, 1.00)	1.63 (0.96, 2.76)
Diourbel	1.60 (1.01, 2.54)	1.22 (0.78, 1.92)	0.39 (0.27, 0.56)	0.61 (0.42, 0.89)
Saint-Louis	1.10 (0.70, 1.71)	0.73 (0.46, 1.16)	0.57 (0.39, 0.84)	0.94 (0.60, 1.46)
Tambacounda	1.40 (0.81, 2.42)	1.16 (0.66, 2.04)	0.54 (0.35, 0.83)	1.18 (0.68, 2.04)
Kaolack	0.90 (0.60, 1.34)	0.71 (0.46, 1.09)	0.37 (0.25, 0.55)	0.57 (0.36, 0.92)
Thiès	1.07 (0.66, 1.75)	0.80 (0.48, 1.34)	0.56 (0.40, 0.78)	0.81 (0.54, 1.21)
Louga	2.55 (1.67, 3.91)	1.89 (1.18, 3.00)	0.44 (0.28, 0.70)	0.78 (0.46, 1.31)
Fatick	0.74 (0.44, 1.25)	0.57 (0.32, 1.02)	0.43 (0.28, 0.67)	0.97 (0.60, 1.58)
Kolda	1.34 (0.82, 2.20)	1.06 (0.61, 1.84)	0.48 (0.31, 0.73)	1.13 (0.68, 1.88)
Matam	1.46 (0.96, 2.22)	1.01 (0.64, 1.61)	0.41 (0.28, 0.59)	0.77 (0.49, 1.22)
Kaffrine	1.19 (0.75, 1.87)	0.82 (0.49, 1.37)	0.32 (0.20, 0.50)	0.64 (0.37, 1.08)
Kedougou	0.71 (0.39, 1.30)	0.68 (0.34, 1.38)	0.21 (0.13, 0.34)	0.40 (0.22, 0.71)
Sedhiou	1.01 (0.63, 1.61)	1.06 (0.61, 1.86)	0.32 (0.21, 0.47)	0.62 (0.32, 1.22)

VARIABLES	UNDERWEIGHT		OVERWEIGHT/OBESITY	
	Unadjusted RRR (95% CI)	Adjusted RRR (95% CI)	Unadjusted RRR (95% CI)	Adjusted RRR (95% CI)
**Level of Education**				
No Education (Reference)	1.00	1.00	1.00	1.00
Primary	0.93 (0.73, 1.18)	0.99 (0.76, 1.29)	1.31 (1.03, 1.66)	1.17 (0.84, 1.62)
Secondary	1.11 (0.86, 1.45)	1.09 (0.73, 1.65)	0.96 (0.72, 1.27)	1.27 (0.89, 1.81)
Higher	0.10 (0.03, 0.38)	0.13 (0.03, 0.53)	1.32 (0.65, 2.68)	0.87 (0.36, 2.08)
**Wealth Quintile**				
Poorest (Reference)	1.00	1.00	1.00	1.00
Poorer	0.92 (0.71, 1.19)	0.99 (0.73, 1.34)	1.05 (0.79, 1.40)	0.98 (0.72, 1.32)
Middle	0.91 (0.70, 1.18)	0.98 (0.71, 1.35)	1.69 (1.26, 2.27)	1.22 (0.87, 1.72)
Richer	1.16 (0.88, 1.53)	1.37 (0.94, 1.99)	2.36 (1.79, 3.13)	1.38 (0.94, 2.01)
Richest	0.72 (0.52, 0.99)	0.84 (0.55, 1.28)	2.88 (2.05, 4.05)	1.67 (1.11, 2.51)
**Occupation**				
Not Working (Reference)	1.00	1.00	1.00	1.00
Nonmanual	0.65 (0.53, 0.79)	0.76 (0.61, 0.96)	2.58 (2.08, 3.20)	1.53 (1.17, 2.01)
Manual	0.48 (0.20, 1.15)	0.49 (0.21, 1.17)	1.18 (0.67, 2.10)	0.80 (0.42, 1.50)
Agricultural	0.81 (0.62, 1.04)	0.91 (0.69, 1.21)	1.08 (0.79, 1.48)	1.12 (0.78, 1.62)
**Media Exposure**				
Not Exposed (Reference)	1.00	1.00	1.00	1.00
Exposed to one media source	0.80 (0.58, 1.10)	0.76 (0.55, 1.04)	1.11 (0.79, 1.57)	0.95 (0.67, 1.35)
Exposed to two media sources	0.83 (0.61, 1.13)	0.68 (0.49, 0.92)	1.88 (1.34, 2.62)	1.22 (0.84, 1.77)
Exposed to three media sources	0.67 (0.45, 1.00)	0.51 (0.32, 0.82)	1.92 (1.24, 2.97)	1.08 (0.63, 1.86)
**Hormonal Contraceptive Use**				
No (Reference)	1.00	1.00	1.00	1.00
Yes	0.49 (0.28, 0.85)	0.72 (0.42, 1.25)	1.63 (1.20, 2.20)	1.04 (0.70, 1.54)

Overall Model Fit: F (100, 263) = 10.15, *P*=0.0000.

Table S47: Unadjusted and adjusted relative risk ratio, of underweight and overweight/obesity among women of child-bearing age in Namibia.

VARIABLES	UNDERWEIGHT		OVERWEIGHT/OBESITY	
	Unadjusted RRR (95% CI)	Adjusted RRR (95% CI)	Unadjusted RRR (95% CI)	Adjusted RRR (95% CI)
**Age**				
15-19 (Reference)	1.00	1.00	1.00	1.00
20-24	0.56 (0.42, 0.74)	0.84 (0.61, 1.15)	2.51 (1.66, 3.80)	1.53 (1.00, 2.33)
25-29	0.40 (0.27, 0.59)	0.74 (0.46, 1.19)	5.06 (3.33, 7.68)	2.53 (1.66, 3.87)
30-34	0.36 (0.23, 0.56)	0.67 (0.38, 1.18)	8.27 (5.55, 12.3)	4.32 (2.81, 6.63)
35-39	0.57 (0.38, 0.85)	0.99 (0.58, 1.70)	8.77 (5.80, 13.2)	4.43 (2.74, 7.14)
40-44	0.72 (0.47, 1.10)	1.05 (0.61, 1.81)	14.3 (9.14, 22.2)	7.65 (4.46, 13.1)
45-49	0.60 (0.39, 0.94)	0.70 (0.39, 1.27)	11.1 (7.21, 17.1)	5.76 (3.40, 9.75)
**Religion**				
Catholics (Reference)	1.00	1.00	1.00	1.00
Other Christians	0.74 (0.56, 0.97)	0.72 (0.53, 0.98)	0.98 (0.79, 1.22)	0.83 (0.64, 1.07)
Others	0.60 (0.35, 1.03)	0.66 (0.37, 1.15)	1.36 (1.00, 1.84)	0.83 (0.58, 1.19)
No Religion	0.46 (0.14, 1.49)	0.41 (0.12, 1.42)	1.35 (0.45, 4.03)	1.52 (0.54, 4.31)
**Parity**				
0 (Reference)	1.00	1.00	1.00	1.00
1	0.57 (0.41, 0.79)	0.71 (0.49, 1.03)	3.08 (2.28, 4.16)	2.00 (1.42, 2.82)
2	0.49 (0.35, 0.68)	0.58 (0.37, 0.90)	4.14 (3.14, 5.45)	1.93 (1.37, 2.73)
3	0.37 (0.24, 0.58)	0.44 (0.26, 0.75)	4.47 (3.32, 6.02)	1.59 (1.05, 2.42)
4	0.60 (0.37, 0.98)	0.64 (0.37, 1.10)	5.79 (4.13, 8.13)	2.17 (1.38, 3.41)
5	0.72 (0.41, 1.27)	0.62 (0.33, 1.19)	6.12 (4.14, 9.04)	2.81 (1.64, 4.83)
6+	1.08 (0.71, 1.64)	0.90 (0.48, 1.71)	3.51 (2.43, 5.08)	1.69 (0.97, 2.93)
**Marital Status**				
Single (Reference)	1.00	1.00	1.00	1.00
Married	0.65 (0.50, 0.83)	0.86 (0.63, 1.18)	2.79 (2.34, 3.32)	1.52 (1.20, 1.93)
Formerly Married	1.18 (0.75, 1.85)	1.46 (0.87, 2.46)	2.66 (1.96, 3.60)	1.33 (0.90, 1.96)
**Residential Setting**				
Rural (Reference)	1.00	1.00	1.00	1.00
Urban	0.75 (0.59, 0.97)	1.25 (0.91, 1.71)	2.23 (1.89, 2.63)	0.97 (0.76, 1.24)
**Region**				
Khomas (Reference)	1.00	1.00	1.00	1.00
Caprivi	0.85 (0.45, 1.59)	0.79 (0.40, 1.58)	0.48 (0.32, 0.71)	0.69 (0.45, 1.06)
Erongo	0.72 (0.37, 1.41)	0.78 (0.39, 1.55)	1.08 (0.78, 1.49)	0.93 (0.66, 1.31)
Hardap	1.83 (1.01, 3.30)	1.74 (0.91, 3.34)	1.38 (0.96, 1.98)	1.25 (0.83, 1.90)
Karas	0.90 (0.49, 1.67)	0.87 (0.45, 1.68)	1.24 (0.93, 1.66)	1.03 (0.72, 1.47)
Kavango	1.29 (0.74, 2.27)	0.88 (0.45, 1.72)	0.33 (0.23, 0.46)	0.51 (0.35, 0.76)
Kunene	1.43 (0.71, 2.85)	1.42 (0.66, 3.06)	1.36 (0.93, 1.98)	1.72 (1.09, 2.70)
Changwena	1.94 (1.12, 3.36)	1.55 (0.83, 2.90)	0.29 (0.20, 0.42)	0.50 (0.31, 0.81)
Omaheke	1.37 (0.75, 2.48)	1.23 (0.63, 2.39)	0.98 (0.67, 1.44)	1.05 (0.64, 1.71)
Omusati	1.48 (0.85, 2.58)	1.06 (0.56, 2.01)	0.36 (0.25, 0.53)	0.58 (0.35, 0.96)
Oshana	1.27 (0.71, 2.27)	1.12 (0.60, 2.06)	0.63 (0.44, 0.91)	0.77 (0.51, 1.17)
Oshikoto	1.14 (0.63, 2.05)	0.99 (0.51, 1.89)	0.57 (0.42, 0.78)	0.84 (0.58, 1.20)
Otjozondjupa	1.45 (0.76, 2.77)	1.29 (0.65, 2.57)	1.14 (0.83, 1.59)	0.96 (0.66, 1.41)
**Level of Education**				
No Education (Reference)	1.00	1.00	1.00	1.00
Primary	1.52 (0.96, 2.40)	1.47 (0.90, 2.41)	0.68 (0.45, 1.05)	0.87 (0.54, 1.41)
Secondary	0.92 (0.58, 1.46)	1.03 (0.60, 1.74)	0.86 (0.58, 1.27)	0.93 (0.58, 1.48)
Higher	0.64 (0.33, 1.26)	0.85 (0.41, 1.79)	1.19 (0.75, 1.90)	0.96 (0.53, 1.75)

VARIABLES	UNDERWEIGHT		OVERWEIGHT/OBESITY	
	Unadjusted RRR (95% CI)	Adjusted RRR (95% CI)	Unadjusted RRR (95% CI)	Adjusted RRR (95% CI)
**Wealth Quintile**				
Poorest (Reference)	1.00	1.00	1.00	1.00
Poorer	0.73 (0.53, 1.02)	0.82 (0.59, 1.16)	1.60 (1.12, 2.29)	1.33 (0.89, 1.99)
Middle	0.71 (0.50, 1.00)	0.81 (0.56, 1.19)	2.71 (1.94, 3.78)	2.08 (1.40, 3.09)
Richer	0.67 (0.48, 0.95)	0.82 (0.52, 1.31)	4.74 (3.46, 6.50)	3.58 (2.35, 5.44)
Richest	0.51 (0.34, 0.76)	0.62 (0.36, 1.07)	4.56 (3.30, 6.31)	3.52 (2.24, 5.53)
**Occupation**				
Not Working (Reference)	1.00	1.00	1.00	1.00
Nonmanual	0.55 (0.42, 0.71)	0.73 (0.54, 0.99)	2.86 (2.38, 3.42)	1.26 (1.01, 1.56)
Manual	0.51 (0.22, 1.21)	0.66 (0.29, 1.50)	2.74 (1.78, 4.21)	1.12 (0.68, 1.84)
Agricultural	0.82 (0.35, 1.91)	0.94 (0.40, 2.23)	1.70 (1.05, 2.78)	1.07 (0.60, 1.92)
**Media Exposure**				
Not Exposed (Reference)	1.00	1.00	1.00	1.00
Exposed to one media source	0.87 (0.62, 1.24)	0.96 (0.67, 1.37)	1.15 (0.78, 1.68)	1.20 (0.79, 1.83)
Exposed to two media sources	0.93 (0.67, 1.29)	1.08 (0.74, 1.57)	1.72 (1.20, 2.45)	1.45 (0.95, 2.22)
Exposed to three media sources	0.58 (0.40, 0.85)	0.75 (0.48, 1.19)	2.71 (1.96, 3.76)	1.68 (1.09, 2.59)
**Hormonal Contraceptive Use**				
No (Reference)	1.00	1.00	1.00	1.00
Yes	0.54 (0.41, 0.71)	0.72 (0.54, 0.95)	1.41 (1.18, 1.68)	1.11 (0.89, 1.38)

Overall Model Fit: F (88, 431) = 8.68, *P*=0.0000.

Table S48: Unadjusted and adjusted relative risk ratio, of underweight and overweight/obesity among women of child-bearing age in Congo.

VARIABLES	UNDERWEIGHT		OVERWEIGHT/OBESITY	
	Unadjusted RRR (95% CI)	Adjusted RRR (95% CI)	Unadjusted RRR (95% CI)	Adjusted RRR (95% CI)
**Age**				
15-19 (Reference)	1.00	1.00	1.00	1.00
20-24	0.56 (0.37, 0.84)	0.74 (0.45, 1.22)	2.95 (1.90, 4.57)	2.59 (1.55, 4.34)
25-29	0.49 (0.32, 0.74)	0.69 (0.39, 1.21)	5.16 (3.31, 8.04)	4.59 (2.53, 8.31)
30-34	0.60 (0.36, 1.00)	0.81 (0.44, 1.47)	9.56 (5.64, 16.2)	8.85 (4.61, 17.0)
35-39	0.56 (0.37, 0.83)	0.73 (0.40, 1.34)	8.96 (5.33, 15.1)	9.06 (4.51, 18.2)
40-44	0.85 (0.51, 1.42)	1.04 (0.57, 1.89)	9.50 (5.97, 15.1)	12.6 (6.29, 25.4)
45-49	0.42 (0.25, 0.70)	0.50 (0.23, 1.12)	7.96 (4.89, 12.9)	12.9 (6.45, 25.8)
**Ethnicity**				
Kongo (Reference)	1.00	1.00	1.00	1.00
Punu	1.11 (0.58, 2.14)	1.25 (0.60, 2.59)	0.88 (0.56, 1.38)	1.00 (0.59, 1.72)
Duma	0.84 (0.44, 1.60)	0.88 (0.49, 1.61)	0.40 (0.14, 1.09)	0.81 (0.28, 2.35)
Mbéré/Mbéti/Kélé	0.47 (0.20, 1.12)	0.56 (0.24, 1.32)	1.10 (0.55, 2.20)	1.30 (0.53, 3.19)
Téké	0.99 (0.70, 1.40)	1.16 (0.78, 1.73)	1.07 (0.76, 1.51)	1.32 (0.85, 2.06)
Mbochi	1.22 (0.76, 1.96)	1.46 (0.80, 2.67)	0.90 (0.66, 1.22)	0.93 (0.65, 1.33)
Sangha	0.85 (0.43, 1.71)	1.96 (0.67, 5.76)	1.19 (0.73, 1.95)	2.17 (1.13, 4.17)
Oubanguiens	0.45 (0.18, 1.15)	1.19 (0.38, 3.79)	0.84 (0.56, 1.26)	1.90 (1.03, 3.48)
Pygmée	1.54 (0.78, 3.04)	3.05 (1.39, 6.68)	0.15 (0.05, 0.46)	1.08 (0.32, 3.64)
Etranger	0.48 (0.23, 0.99)	0.65 (0.30, 1.38)	1.14 (0.81, 1.60)	1.45 (0.87, 2.45)
Others	1.22 (0.27, 5.42)	1.62 (0.28, 9.24)	1.49 (0.56, 3.97)	2.15 (0.91, 5.11)
**Religion**				
Catholics (Reference)	1.00	1.00	1.00	1.00
Other Christians	0.84 (0.63, 1.12)	0.84 (0.65, 1.10)	0.90 (0.73, 1.12)	0.97 (0.76, 1.23)
Islam	0.44 (0.09, 2.23)	1.01 (0.16, 6.33)	1.52 (0.76, 3.03)	1.37 (0.60, 3.11)
Traditionalist	1.39 (0.88, 2.19)	1.30 (0.84, 2.01)	0.49 (0.29, 0.82)	0.64 (0.35, 1.18)
Others	0.30 (0.08, 1.07)	0.29 (0.07, 1.21)	0.64 (0.16, 2.52)	0.83 (0.24, 2.90)
No Religion	0.85 (0.50, 1.45)	0.68 (0.39, 1.18)	0.43 (0.20, 0.90)	0.69 (0.35, 1.36)
**Parity**				
0 (Reference)	1.00	1.00	1.00	1.00
1	0.85 (0.58, 1.25)	1.13 (0.77, 1.65)	2.45 (1.62, 3.69)	1.58 (0.89, 2.80)
2	0.61 (0.41, 0.90)	0.86 (0.53, 1.37)	2.81 (1.87, 4.23)	1.35 (0.74, 2.47)
3	0.46 (0.30, 0.71)	0.69 (0.39, 1.21)	3.24 (2.11, 4.97)	1.30 (0.72, 2.34)
4	0.91 (0.57, 1.43)	1.32 (0.73, 2.40)	4.55 (2.97, 6.96)	1.69 (0.83, 3.41)
5	0.70 (0.40, 1.23)	1.10 (0.55, 2.23)	3.57 (2.20, 5.80)	1.27 (0.60, 2.69)
6+	0.70 (0.44, 1.10)	1.05 (0.56, 1.99)	2.77 (1.80, 4.26)	1.06 (0.53, 2.11)
**Marital Status**				
Single (Reference)	1.00	1.00	1.00	1.00
Married	0.58 (0.44, 0.76)	0.74 (0.49, 1.10)	2.84 (2.10, 3.84)	0.99 (0.63, 1.54)
Formerly Married	0.85 (0.56, 1.30)	0.99 (0.61, 1.60)	2.53 (1.84, 3.47)	0.85 (0.53, 1.37)
**Residential Setting**				
Rural (Reference)	1.00	1.00	1.00	1.00
Urban	1.13 (0.87, 1.47)	1.56 (0.90, 2.70)	2.81 (2.28, 3.45)	1.14 (0.65, 2.00)

VARIABLES	UNDERWEIGHT		OVERWEIGHT/OBESITY	
	Unadjusted RRR (95% CI)	Adjusted RRR (95% CI)	Unadjusted RRR (95% CI)	Adjusted RRR (95% CI)
**Region**				
Brazzaville (Reference)	1.00	1.00	1.00	1.00
Kouilou	0.87 (0.59, 1.27)	1.03 (0.55, 1.90)	0.37 (0.26, 0.53)	1.06 (0.54, 2.06)
Niari	0.97 (0.63, 1.47)	1.01 (0.58, 1.74)	0.51 (0.33, 0.79)	0.84 (0.49, 1.45)
Lekoumou	0.78 (0.50, 1.23)	0.89 (0.43, 1.85)	0.24 (0.15, 0.37)	0.52 (0.24, 1.12)
Bouenza	1.20 (0.79, 1.82)	1.43 (0.84, 2.41)	0.40 (0.26, 0.63)	0.87 (0.49, 1.56)
Pool	0.85 (0.55, 1.33)	1.02 (0.53, 1.98)	0.28 (0.19, 0.41)	0.65 (0.32, 1.32)
Plateaux	1.70 (1.13, 2.56)	1.81 (0.87, 3.78)	0.28 (0.17, 0.46)	0.66 (0.30, 1.45)
Cuvette	0.79 (0.47, 1.34)	0.89 (0.37, 2.15)	0.53 (0.36, 0.79)	0.83 (0.41, 1.68)
Cuvette - Ouest	0.99 (0.53, 1.86)	1.46 (0.60, 3.56)	0.25 (0.14, 0.43)	0.38 (0.16, 0.92)
Sangha	0.49 (0.27, 0.89)	0.34 (0.13, 0.88)	0.71 (0.49, 1.02)	0.87 (0.46, 1.66)
Likouala	0.49 (0.29, 0.82)	0.51 (0.19, 1.33)	0.43 (0.30, 0.61)	0.75 (0.36, 1.57)
Pointe-noire	0.98 (0.57, 1.68)	0.98 (0.59, 1.63)	1.09 (0.80, 1.47)	1.22 (0.85, 1.74)
**Level of Education**				
No Education (Reference)	1.00	1.00	1.00	1.00
Primary	1.17 (0.81, 1.71)	1.05 (0.69, 1.60)	1.19 (0.72, 1.97)	1.48 (0.83, 2.66)
Secondary	1.22 (0.82, 1.83)	1.05 (0.65, 1.69)	1.96 (1.22, 3.16)	1.60 (0.88, 2.90)
Higher	0.34 (0.11, 1.05)	0.39 (0.10, 1.46)	2.52 (1.36, 4.65)	1.27 (0.60, 2.69)
**Wealth Quintile**				
Poorest (Reference)	1.00	1.00	1.00	1.00
Poorer	1.18 (0.86, 1.62)	0.96 (0.66, 1.38)	1.81 (1.28, 2.58)	1.21 (0.88, 1.66)
Middle	1.46 (0.93, 2.28)	1.01 (0.56, 1.81)	2.52 (1.84, 3.45)	1.30 (0.81, 2.08)
Richer	0.81 (0.52, 1.26)	0.58 (0.29, 1.19)	3.74 (2.75, 5.07)	2.08 (1.23, 3.51)
Richest	0.69 (0.47, 1.02)	0.44 (0.22, 0.86)	5.27 (4.06, 6.85)	2.77 (1.68, 4.59)
**Occupation**				
Not Working (Reference)	1.00	1.00	1.00	1.00
Nonmanual	0.73 (0.50, 1.06)	0.83 (0.59, 1.16)	2.13 (1.62, 2.79)	1.15 (0.83, 1.61)
Manual	0.71 (0.37, 1.38)	0.88 (0.46, 1.69)	1.29 (0.75, 2.22)	0.81 (0.45, 1.47)
Agricultural	0.77 (0.58, 1.01)	0.84 (0.54, 1.30)	0.53 (0.40, 0.71)	0.55 (0.35, 0.85)
Others	0.44 (0.14, 1.39)	0.87 (0.28, 2.72)	0.58 (0.14, 2.44)	0.29 (0.07, 1.14)
**Media Exposure**				
Not Exposed (Reference)	1.00	1.00	1.00	1.00
Exposed to one media source	0.83 (0.63, 1.11)	0.92 (0.69, 1.23)	1.50 (1.10, 2.03)	0.88 (0.62, 1.25)
Exposed to two media sources	0.80 (0.56, 1.15)	0.90 (0.60, 1.34)	1.84 (1.36, 2.47)	0.85 (0.58, 1.24)
Exposed to three media sources	0.90 (0.63, 1.27)	1.20 (0.78, 1.87)	2.83 (2.03, 3.96)	1.05 (0.69, 1.60)
**Hormonal Contraceptives use**				
No (Reference)	1.00	1.00	1.00	1.00
Yes	1.01 (0.39, 2.65)	1.13 (0.42, 3.05)	1.86 (1.25, 2.77)	1.14 (0.72, 1.79)

Overall Model Fit: F (112, 248) = 6.76, *P*=0.0000

Table S49: Unadjusted and adjusted relative risk ratio, of underweight and overweight/obesity among women of child-bearing age in Nigeria.

VARIABLES	UNDERWEIGHT		OVERWEIGHT/OBESITY	
	Unadjusted RRR (95% CI)	Adjusted RRR (95% CI)	Unadjusted RRR (95% CI)	Adjusted RRR (95% CI)
**Age**				
15-19 (Reference)	1.00	1.00	1.00	1.00
20-24	0.53 (0.47, 0.59)	0.72 (0.64, 0.82)	2.15 (1.85, 2.51)	1.73 (1.47, 2.04)
25-29	0.43 (0.38, 0.49)	0.66 (0.56, 0.78)	4.21 (3.63, 4.89)	2.97 (2.48, 3.55)
30-34	0.36 (0.30, 0.42)	0.57 (0.46, 0.70)	6.01 (5.20, 6.94)	4.13 (3.42, 5.00)
35-39	0.35 (0.30, 0.42)	0.57 (0.44, 0.72)	8.88 (7.65, 10.3)	6.58 (5.39, 8.04)
40-44	0.34 (0.28, 0.41)	0.53 (0.41, 0.68)	8.72 (7.53, 10.1)	7.05 (5.74, 8.66)
45-49	0.40 (0.34, 0.49)	0.58 (0.44, 0.76)	9.02 (7.70, 10.6)	8.26 (6.70, 10.2)
**Ethnicity**				
Hausa (Reference)	1.00	1.00	1.00	1.00
Ekoi	0.19 (0.02, 1.70)	0.25 (0.02, 2.63)	2.03 (0.62, 6.61)	1.25 (0.40, 3.94)
Fulani	1.36 (1.12, 1.65)	1.33 (1.08, 1.64)	0.71 (0.53, 0.93)	0.78 (0.55, 1.11)
Ibibio	0.56 (0.42, 0.74)	0.84 (0.53, 1.31)	2.89 (2.31, 3.61)	1.65 (1.13, 2.40)
Igala	0.57 (0.29, 1.14)	0.69 (0.36, 1.34)	1.66 (1.27, 2.16)	1.20 (0.83, 1.73)
Igbo	0.47 (0.39, 0.56)	0.88 (0.59, 1.31)	2.45 (2.08, 2.88)	1.45 (1.06, 1.98)
Ijaw/Izon	0.56 (0.39, 0.81)	1.18 (0.64, 2.17)	2.53 (1.99, 3.20)	1.56 (1.11, 2.20)
Kanuri/Beriberi	0.86 (0.57, 1.30)	0.84 (0.54, 1.30)	0.90 (0.61, 1.32)	0.68 (0.46, 1.00)
Tiv	0.45 (0.29, 0.69)	0.88 (0.51, 1.51)	1.22 (0.93, 1.61)	1.47 (0.93, 2.33)
Yoruba	0.73 (0.63, 0.84)	0.99 (0.70, 1.40)	2.69 (2.34, 3.10)	1.11 (0.84, 1.47)
Others	0.55 (0.47, 0.63)	0.79 (0.63, 1.00)	1.71 (1.49, 1.97)	1.15 (0.92, 1.44)
**Religion**				
Other Christians (Reference)	1.00	1.00	1.00	1.00
Catholics	0.97 (0.78, 1.20)	1.11 (0.88, 1.40)	0.89 (0.80, 0.99)	0.99 (0.87, 1.12)
Islam	1.91 (1.71, 2.12)	1.33 (1.12, 1.59)	0.54 (0.49, 0.60)	1.07 (0.94, 1.23)
Traditionalists	1.12 (0.74, 1.68)	1.01 (0.61, 1.67)	0.39 (0.24, 0.61)	0.74 (0.48, 1.14)
**Parity**				
0 (Reference)	1.00	1.00	1.00	1.00
1	0.58 (0.50, 0.68)	0.76 (0.62, 0.93)	1.63 (1.42, 1.87)	0.93 (0.77, 1.14)
2	0.55 (0.47, 0.65)	0.84 (0.68, 1.05)	2.02 (1.76, 2.33)	0.87 (0.70, 1.09)
3	0.54 (0.45, 0.64)	0.87 (0.69, 1.10)	2.67 (2.33, 3.05)	0.99 (0.80, 1.23)
4	0.54 (0.45, 0.65)	0.92 (0.73, 1.17)	3.05 (2.69, 3.45)	0.98 (0.79, 1.20)
5	0.48 (0.39, 0.60)	0.81 (0.62, 1.05)	2.97 (2.57, 3.43)	0.99 (0.79, 1.24)
6+	0.50 (0.44, 0.57)	0.78 (0.62, 0.98)	2.48 (2.21, 2.78)	0.91 (0.74, 1.11)

VARIABLES	UNDERWEIGHT		OVERWEIGHT/OBESITY	
	Unadjusted RRR (95% CI)	Adjusted RRR (95% CI)	Unadjusted RRR (95% CI)	Adjusted RRR (95% CI)
**Marital Status**				
Single (Reference)	1.00	1.00	1.00	1.00
Married	0.58 (0.52, 0.65)	0.54 (0.44, 0.65)	2.51 (2.25, 2.80)	1.74 (1.45, 2.09)
Formerly Married	0.61 (0.49, 0.74)	0.75 (0.59, 0.96)	4.09 (3.53, 4.73)	2.02 (1.62, 2.53)
**Residential Setting**				
Rural (Reference)	1.00	1.00	1.00	1.00
Urban	0.90 (0.81, 1.01)	1.11 (0.97, 1.28)	2.15 (1.96, 2.36)	1.15 (1.02, 1.29)

VARIABLES	UNDERWEIGHT		OVERWEIGHT/OBESITY	
	Unadjusted RRR (95% CI)	Adjusted RRR (95% CI)	Unadjusted RRR (95% CI)	Adjusted RRR (95% CI)
**Region**				
Lagos (Reference)	1.00	1.00	1.00	1.00
Sokoto	1.82 (1.22, 2.71)	1.04 (0.63, 1.72)	0.21 (0.16, 0.27)	0.82 (0.56, 1.18)
Zamfara	1.38 (0.96, 1.99)	0.88 (0.54, 1.43)	0.18 (0.13, 0.24)	0.73 (0.49, 1.09)
Katsina	1.28 (0.87, 1.87)	0.84 (0.52, 1.37)	0.14 (0.10, 0.20)	0.51 (0.35, 0.75)
Jigawa	1.98 (1.36, 2.90)	1.21 (0.73, 2.00)	0.22 (0.14, 0.37)	0.97 (0.52, 1.81)
Yobe	1.10 (0.65, 1.86)	0.51 (0.26, 0.98)	0.34 (0.25, 0.48)	1.90 (1.24, 2.91)
Borno	1.39 (0.92, 2.12)	0.78 (0.48, 1.27)	0.27 (0.19, 0.37)	1.02 (0.73, 1.43)
Adamawa	1.49 (0.94, 2.36)	1.14 (0.72, 1.80)	0.40 (0.28, 0.56)	1.18 (0.83, 1.69)
Gombe	2.44 (1.61, 3.70)	1.68 (1.03, 2.74)	0.29 (0.21, 0.38)	0.92 (0.68, 1.23)
Bauchi	2.37 (1.64, 3.42)	1.39 (0.88, 2.18)	0.24 (0.17, 0.33)	0.97 (0.69, 1.37)
Kano	1.90 (1.35, 2.67)	1.08 (0.69, 1.69)	0.29 (0.22, 0.38)	0.82 (0.58, 1.14)
Kaduna	0.94 (0.61, 1.47)	0.80 (0.48, 1.34)	0.37 (0.28, 0.50)	0.73 (0.53, 1.02)
Kebbi	0.99 (0.66, 1.50)	0.59 (0.36, 0.96)	0.27 (0.19, 0.40)	1.10 (0.67, 1.81)
Niger	0.54 (0.26, 1.10)	0.38(0.21, 0.70)	0.37 (0.28, 0.49)	1.02 (0.71, 1.47)
Fct-abuja	0.61 (0.36, 1.06)	0.58 (0.34, 0.98)	0.92 (0.68, 1.25)	1.38 (1.04, 1.84)
Nasarawa	0.42 (0.16, 1.06)	0.33 (0.13, 0.82)	0.35 (0.28, 0.45)	0.97 (0.74, 1.29)
Plateau	0.44 (0.27, 0.71)	0.39 (0.23, 0.67)	0.44 (0.33, 0.58)	1.12 (0.85, 1.47)
Taraba	0.89 (0.51, 1.53)	0.58 (0.33, 1.03)	0.40 (0.31, 0.52)	1.43 (1.02, 2.02)
Benue	0.71 (0.47, 1.07)	0.61 (0.37, 1.01)	0.28 (0.20, 0.39)	0.88 (0.53, 1.47)
Kogi	0.93 (0.58, 1.50)	0.82 (0.54, 1.24)	0.48 (0.38, 0.60)	0.95 (0.71, 1.26)
Kwara	1.17 (0.79, 1.74)	0.80 (0.53, 1.21)	0.59 (0.47, 0.74)	0.90 (0.70, 1.14)
Oyo	1.49 (0.95, 2.34)	1.23 (0.83, 1.81)	0.53 (0.42, 0.66)	0.70 (0.54, 0.93)
Osun	0.93 (0.63, 1.38)	0.77 (0.52, 1.13)	0.49 (0.39, 0.63)	0.63 (0.49, 0.80)
Ekiti	0.72 (0.46, 1.11)	0.62 (0.40, 0.96)	0.53 (0.40, 0.71)	0.72 (0.51, 1.01)
Ondo	0.88 (0.56, 1.38)	0.74 (0.47, 1.15)	0.56 (0.44, 0.71)	0.88 (0.69, 1.12)
Edo	0.87 (0.54, 1.41)	0.79 (0.48, 1.32)	0.62 (0.50, 0.76)	1.09 (0.81, 1.46)
Anambra	0.33 (0.17, 0.66)	0.29 (0.14, 0.60)	0.65 (0.47, 0.92)	0.78 (0.49, 1.24)
Enugu	0.57 (0.35, 0.94)	0.43 (0.22, 0.82)	0.61 (0.46, 0.79)	1.11 (0.80, 1.53)
Ebonyi	1.16 (0.82, 1.65)	0.89 (0.54, 1.45)	0.26 (0.18, 0.40)	0.51 (0.34, 0.78)
Cross River	0.89 (0.57, 1.41)	0.97 (0.58, 1.61)	0.50 (0.38, 0.65)	0.97 (0.69, 1.37)
Akwa Ibom	0.86 (0.57, 1.30)	0.84 (0.50, 1.40)	0.58 (0.44, 0.77)	0.96 (0.66, 1.40)
Abia	0.71 (0.46, 1.10)	0.72 (0.41, 1.27)	0.53 (0.38, 0.73)	0.64 (0.44, 0.92)
Imo	0.63 (0.40, 1.00)	0.57 (0.31, 1.03)	0.70 (0.50, 0.99)	0.93 (0.61, 1.41)
Rivers	0.71 (0.42, 1.19)	0.70 (0.40, 1.23)	0.80 (0.61, 1.07)	1.24 (0.91, 1.68)
Bayelsa	0.61 (0.40, 0.93)	0.42 (0.21, 0.81)	0.55 (0.45, 0.68)	
Delta	0.72 (0.49, 1.06)	0.59 (0.39, 0.90)	0.46 (0.36, 0.58)	
Ogun	1.35 (0.89, 2.04)	1.41 (0.93, 2.13)	0.71 (0.54, 0.94)	

VARIABLES	UNDERWEIGHT		OVERWEIGHT/OBESITY	
	Unadjusted RRR (95% CI)	Adjusted RRR (95% CI)	Unadjusted RRR (95% CI)	Adjusted RRR (95% CI)
**Level of Education**				
No Education	1.00	1.00	1.00	1.00
Primary	0.82 (0.72, 0.93)	1.09 (0.94, 1.28)	2.13 (1.89, 2.40)	1.29 (1.14, 1.46)
Secondary	0.81 (0.72, 0.91)	0.80 (0.67, 0.96)	1.78 (1.59, 1.99)	1.39 (1.22, 1.58)
Higher	0.39 (0.31, 0.50)	0.61 (0.46, 0.82)	4.07 (3.55, 4.65)	1.68 (1.43, 1.97)
**Wealth Quintile**				
Poorest (Reference)	1.00	1.00	1.00	1.00
Poorer	0.80 (0.69, 0.92)	0.91 (0.78, 1.06)	1.43 (1.20, 1.70)	1.45 (1.22, 1.72)
Middle	0.72 (0.61, 0.84)	0.96 (0.80, 1.14)	2.22 (1.87, 2.63)	1.97 (1.65, 2.36)
Richer	0.67 (0.58, 0.78)	0.90 (0.74, 1.10)	3.49 (2.95, 4.14)	2.70 (2.23, 3.28)
Richest	0.49 (0.41, 0.59)	0.69 (0.54, 0.87)	5.58 (4.70, 6.62)	3.74 (3.04, 4.61)
**Occupation**				
Not Working (Reference)	1.00	1.00	1.00	1.00
Nonmanual	0.52 (0.46, 0.57)	0.76 (0.68, 0.85)	2.91 (2.65, 3.19)	1.27 (1.14, 1.41)
Manual	0.83 (0.70, 0.97)	0.94 (0.80, 1.11)	1.74 (1.52, 1.98)	1.08 (0.94, 1.24)
Agricultural	0.43 (0.36, 0.51)	0.85 (0.69, 1.04)	1.42 (1.22, 1.65)	0.75 (0.63, 0.89)
Others	1.69 (0.82, 3.50)	1.49 (0.78, 2.83)	1.16 (0.38, 3.61)	1.11 (0.45, 2.75)
**Media Exposure**				
Not Exposed (Reference)	1.00	1.00	1.00	1.00
Exposed to one media source	0.83 (0.74, 0.94)	0.87 (0.76, 0.99)	1.40 (1.25, 1.58)	1.19 (1.05, 1.34)
Exposed to two media sources	0.61 (0.53, 0.69)	0.67 (0.57, 0.79)	2.25 (2.01, 2.51)	1.15 (1.02, 1.30)
Exposed to three media sources	0.60 (0.51, 0.70)	0.65 (0.53, 0.79)	2.96 (2.61, 3.35)	1.34 (1.17, 1.54)
**Hormonal Contraceptive Use**				
No (Reference)	1.00	1.00	1.00	1.00
Yes	0.26 (0.19, 0.36)	0.53 (0.38, 0.74)	2.06 (1.81, 2.34)	1.06 (0.93, 1.22)

Overall Model Fit: F (158, 666) = 26.99, *P*=0.0000.

Table S50: Unadjusted and adjusted relative risk ratio, of underweight and overweight/obesity among women of child-bearing age in Zambia.

VARIABLES	UNDERWEIGHT		OVERWEIGHT/OBESITY	
	Unadjusted RRR (95% CI)	Adjusted RRR (95% CI)	Unadjusted RRR (95% CI)	Adjusted RRR (95% CI)

	UNDERWEIGHT		OVERWEIGHT/OBESITY	
**Age**				
15-19 (Reference)	1.00	1.00	1.00	1.00
20-24	0.58 (0.47, 0.72)	0.76 (0.60, 0.97)	1.97 (1.57, 2.48)	1.51 (1.17, 1.94)
25-29	0.51 (0.41, 0.63)	0.74 (0.55, 1.00)	3.00 (2.44, 3.68)	1.93 (1.48, 2.51)
30-34	0.53 (0.42, 0.67)	0.82 (0.58, 1.15)	4.04 (3.34, 4.90)	2.55 (1.90, 3.41)
35-39	0.72 (0.58, 0.89)	1.11 (0.80, 1.54)	4.53 (3.66, 5.61)	2.88 (2.08, 3.99)
40-44	0.69 (0.53, 0.90)	1.07 (0.72, 1.59)	5.50 (4.48, 6.75)	4.06 (2.97, 5.55)
45-49	0.83 (0.63, 1.09)	1.26 (0.84, 1.89)	6.19 (4.90, 7.82)	4.82 (3.42, 6.79)
**Ethnicity**				
Bemba (Reference)	1.00	1.00		1.00
Tonga	0.70 (0.55, 0.90)	0.62 (0.42, 0.91)	0.96 (0.78, 1.17)	1.01 (0.79, 1.28)
Chewa	0.76 (0.56, 1.03)	0.89 (0.59, 1.36)	1.21 (0.97, 1.51)	1.30 (0.97, 1.74)
Lozi	1.28 (0.95, 1.73)	0.84 (0.60, 1.19)	0.92 (0.73, 1.17)	1.14 (0.85, 1.52)
Nsenga	0.69 (0.47, 1.01)	0.79 (0.51, 1.21)	1.29 (1.03, 1.62)	1.21 (0.89, 1.66)
Tumbuka	0.69 (0.47, 1.02)	0.78 (0.51, 1.18)	1.16 (0.86, 1.55)	1.18 (0.86, 1.62)
Ngoni	0.73 (0.49, 1.10)	0.92 (0.58, 1.46)	1.59 (1.23, 2.07)	1.27 (0.94, 1.71)
Lala	0.74 (0.49, 1.13)	0.63 (0.39, 1.02)	0.89 (0.64, 1.25)	1.08 (0.77, 1.52)
Kaonde	0.61 (0.37, 0.99)	0.67 (0.39, 1.15)	1.15 (0.80, 1.64)	1.22 (0.84, 1.77)
Namwanga	1.08 (0.76, 1.54)	1.03 (0.72, 1.48)	0.99 (0.71, 1.38)	0.89 (0.60, 1.33)
Mambwe	0.77 (0.50, 1.17)	0.79 (0.51, 1.21)	0.97 (0.71, 1.32)	0.90 (0.66, 1.22)
Lunda(North-Western)	1.06 (0.75, 1.48)	1.29 (0.80, 2.06)	0.98 (0.66, 1.46)	1.47 (0.90, 2.41)
Luvale	0.91 (0.58, 1.42)	0.79 (0.49, 1.29)	1.00 (0.66, 1.52)	1.28 (0.82, 1.99)
Others	1.02 (0.85, 1.24)	0.85 (0.70, 1.05)	1.05 (0.88, 1.25)	1.18 (0.99, 1.42)
**Religion**				
Catholics (Reference)	1.00	1.00	1.00	1.00
Other Christians	1.00 (0.84, 1.20)	1.02 (0.84, 1.23)	1.01 (0.87, 1.17)	0.97 (0.82, 1.15)
Islam	1.62 (0.76, 3.48)	1.81 (0.84, 3.89)	3.26 (1.70, 6.24)	1.46 (0.80, 2.65)
Others	0.91 (0.43, 1.94)	0.76 (0.34, 1.71)	0.63 (0.32, 1.23)	0.74 (0.37, 1.47)
**Parity**				
0 (Reference)	1.00	1.00	1.00	1.00
1	0.64 (0.51, 0.79)	0.89 (0.70, 1.14)	1.46 (1.19, 1.80)	1.02 (0.79, 1.32)
2	0.61 (0.48, 0.76)	1.03 (0.75, 1.42)	2.22 (1.85, 2.66)	1.19 (0.90, 1.57)
3	0.56 (0.44, 0.71)	0.93 (0.65, 1.32)	2.78 (2.33, 3.33)	1.41 (1.03, 1.93)
4	0.76 (0.59, 0.98)	1.17 (0.79, 1.73)	2.69 (2.16, 3.34)	1.41 (1.03, 1.92)
5	0.50 (0.37, 0.67)	0.70 (0.46, 1.07)	2.65 (2.13, 3.28)	1.46 (1.03, 2.07)
6+	0.72 (0.60, 0.85)	0.83 (0.57, 1.21)	2.54 (2.14, 3.00)	1.50 (1.08, 2.08)
**Marital Status**				
Single (Reference)	1.00	1.00	1.00	1.00
Married	0.62 (0.54, 0.71)	0.61 (0.47, 0.80)	2.37 (2.04, 2.77)	1.36 (1.04, 1.77)
Formerly Married	0.64 (0.52, 0.80)	0.57 (0.41, 0.81)	2.32 (1.93, 2.78)	1.19 (0.90, 1.59)
**Residential Setting**				
Rural (Reference)	1.00	1.00	1.00	1.00
Urban	0.87 (0.75, 1.01)	1.22 (0.99, 1.49)	2.69 (2.39, 3.03)	1.12 (0.96, 1.30)
**Region**				
Lusaka (Reference)	1.00	1.00	1.00	1.00
Central	1.00 (0.75, 1.33)	0.88 (0.63, 1.21)	0.43 (0.35, 0.52)	0.75 (0.61, 0.92)
Copperbelt	1.03 (0.78, 1.38)	0.92 (0.68, 1.25)	0.81 (0.68, 0.96)	0.88 (0.73, 1.07)
Eastern	0.73 (0.55, 0.97)	0.51 (0.36, 0.74)	0.44 (0.37, 0.53)	0.90 (0.71, 1.14)
Luapula	0.98 (0.74, 1.29)	0.63 (0.45, 0.87)	0.27 (0.21, 0.35)	0.62 (0.47, 0.83)
Muchinga	1.33 (0.98, 1.79)	0.84 (0.61, 1.17)	0.29 (0.24, 0.37)	0.69 (0.53, 0.89)
Northern	1.25 (0.95, 1.65)	0.75 (0.54, 1.03)	0.27 (0.21, 0.35)	0.67 (0.51, 0.89)
North Western	0.85 (0.61, 1.17)	0.55 (0.36, 0.84)	0.32 (0.25, 0.42)	0.59 (0.41, 0.84)
Southern	0.94 (0.70, 1.25)	1.01 (0.68, 1.50)	0.52 (0.41, 0.64)	0.91 (0.72, 1.15)
Western	1.98 (1.47, 2.66)	1.31 (0.91, 1.89)	0.24 (0.18, 0.31)	0.51 (0.37, 0.69)

	UNDERWEIGHT		OVERWEIGHT/OBESITY	
**Level of Education**				
No Education (Reference)	1.00	1.00	1.00	1.00
Primary	0.99 (0.78, 1.24)	0.94 (0.75, 1.18)	1.06 (0.86, 1.31)	1.00 (0.80, 1.26)
Secondary	0.76 (0.59, 0.97)	0.66 (0.50, 0.86)	1.38 (1.12, 1.70)	1.23 (0.95, 1.58)
Higher	0.64 (0.41, 0.99)	0.76 (0.47, 1.24)	3.66 (2.74, 4.88)	1.67 (1.19, 2.36)
**Wealth Quintile**				
Poorest (Reference)	1.00	1.00	1.00	1.00
Poorer	0.82 (0.68, 0.99)	0.83 (0.69, 1.01)	1.43 (1.17, 1.74)	1.34 (1.09, 1.65)
Middle	0.65 (0.53, 0.79)	0.62 (0.49, 0.79)	2.15 (1.76, 2.62)	1.84 (1.48, 2.28)
Richer	0.56 (0.45, 0.70)	0.44 (0.33, 0.59)	4.16 (3.45, 5.03)	3.23 (2.49, 4.20)
Richest	0.67 (0.53, 0.84)	0.47 (0.33, 0.67)	5.84 (4.83, 7.05)	4.53 (3.42, 6.00)
**Occupation**				
Not Working (Reference)	1.00	1.00	1.00	1.00
Nonmanual	0.66 (0.55, 0.80)	0.79 (0.64, 0.96)	2.16 (1.91, 2.44)	1.15 (0.99, 1.32)
Manual	0.74 (0.36, 1.50)	0.86 (0.42, 1.78)	3.01 (1.95, 4.65)	1.47 (0.90, 2.41)
Agricultural	1.00 (0.87, 1.15)	0.98 (0.84, 1.15)	0.65 (0.57, 0.75)	0.86 (0.74, 1.00)
Others	0.59 (0.28, 1.26)	0.63 (0.29, 1.35)	3.17 (2.17, 4.64)	1.88 (1.26, 2.80)
**Media Exposure**				
Not Exposed (Reference)	1.00	1.00	1.00	1.00
Exposed to one media source	1.00 (0.84, 1.18)	1.10 (0.92, 1.32)	1.30 (1.12, 1.52)	0.99 (0.84, 1.16)
Exposed to two media sources	0.97 (0.80, 1.16)	1.23 (1.00, 1.53)	2.08 (1.77, 2.45)	1.03 (0.86, 1.24)
Exposed to three media sources	0.71 (0.57, 0.89)	0.94 (0.71, 1.25)	2.77 (2.37, 3.23)	1.01 (0.83, 1.24)
**Hormonal Contraceptive Use**				
No (Reference)	1.00	1.00	1.00	1.00
Yes	0.61 (0.51, 0.72)	0.80 (0.66, 0.97)	1.54 (1.39, 1.70)	1.27 (1.12, 1.44)

Overall Model Fit: F (110, 592) = 16.04, *P*=0.0000.

Table S51: Unadjusted and adjusted relative risk ratio, of underweight and overweight/obesity among women of child-bearing age in Sierra Leone.

VARIABLES	UNDERWEIGHT		OVERWEIGHT/OBESITY	
	Unadjusted RRR (95% CI)	Adjusted RRR (95% CI)	Unadjusted RRR (95% CI)	Adjusted RRR (95% CI)
**Age**				
15-19 (Reference)	1.00	1.00	1.00	1.00
20-24	0.51 (0.36, 0.72)	0.67 (0.46, 0.98)	1.74 (1.28, 2.38)	1.73 (1.17, 2.54)
25-29	0.37 (0.27, 0.52)	0.48 (0.30, 0.77)	2.42 (1.77, 3.30)	2.59 (1.71, 3.91)
30-34	0.58 (0.40, 0.83)	0.73 (0.43, 1.23)	3.41 (2.45, 4.74)	3.77 (2.40, 5.92)
35-39	0.55 (0.40, 0.76)	0.67 (0.40, 1.12)	3.60 (2.72, 4.76)	4.03 (2.66, 6.10)
40-44	0.63 (0.41, 0.98)	0.74 (0.39, 1.40)	4.92 (3.52, 6.89)	5.25 (3.28, 8.39)
45-49	0.40 (0.26, 0.61)	0.46 (0.25, 0.85)	4.62 (3.39, 6.30)	5.32 (3.30, 8.56)

	UNDERWEIGHT		OVERWEIGHT/OBESITY	
**Ethnicity**				
Temne (Reference)	1.00	1.00	1.00	1.00
Creole	1.27 (0.41, 3.91)	1.27 (0.38, 4.28)	3.11 (1.57, 6.16)	1.95 (1.05, 3.64)
Fullah	1.29 (0.82, 2.04)	1.47 (0.87, 2.47)	2.25 (1.43, 3.53)	1.38 (0.85, 2.23)
Kono	1.22 (0.82, 1.82)	1.04 (0.61, 1.77)	0.88 (0.60, 1.30)	0.89 (0.53, 1.51)
Limba	0.76 (0.47, 1.24)	0.69 (0.41, 1.16)	1.11 (0.77, 1.61)	1.04 (0.66, 1.62)
Loko	0.83 (0.33, 2.11)	0.78 (0.30, 2.03)	1.70 (0.78, 3.69)	1.42 (0.80, 2.50)
Mandigo	0.91 (0.46, 1.79)	0.94 (0.46, 1.93)	1.47 (0.90, 2.38)	0.92 (0.56, 1.52)
Mende	0.98 (0.74, 1.29)	0.97 (0.64, 1.49)	1.26 (1.01, 1.58)	1.11 (0.84, 1.47)
Sherbro	0.70 (0.33, 1.49)	0.68 (0.32, 1.44)	1.40 (0.89, 2.21)	1.19 (0.74, 1.92)
Koranko	0.93 (0.50, 1.74)	0.85 (0.44, 1.66)	0.61 (0.35, 1.07)	1.01 (0.62, 1.67)
Others	1.50 (0.97, 2.33)	1.46 (0.89, 2.37)	0.97 (0.64, 1.47)	1.04 (0.69, 1.57)
**Religion**				
Other Christians (Reference)	1.00	1.00	1.00	1.00
Islam	0.95 (0.76, 1.18)	0.89 (0.71, 1.12)	0.77 (0.63, 0.94)	0.95 (0.77, 1.19)
Other	0.46 (0.10, 2.27)	0.35 (0.07, 1.76)	0.36 (0.10, 1.28)	0.65 (0.18, 2.39)
**Parity**				
0 (Reference)	1.00	1.00	1.00	1.00
1	0.43 (0.30, 0.61)	0.62 (0.42, 0.92)	1.52 (1.17, 1.97)	1.09 (0.76, 1.55)
2	0.51 (0.36, 0.72)	0.90 (0.56, 1.44)	1.87 (1.39, 2.51)	1.05 (0.71, 1.54)
3	0.52 (0.36, 0.75)	0.94 (0.56, 1.57)	2.10 (1.62, 2.71)	1.18 (0.80, 1.74)
4	0.57 (0.39, 0.82)	1.03 (0.62, 1.70)	2.38 (1.82, 3.10)	1.19 (0.81, 1.75)
5	0.53 (0.34, 0.82)	0.91 (0.52, 1.57)	2.44 (1.80, 3.30)	1.17 (0.80, 1.73)
6+	0.61 (0.45, 0.82)	1.00 (0.58, 1.72)	2.35 (1.84, 3.01)	1.17 (0.78, 1.76)
**Marital Status**				
Single (Reference)	1.00	1.00	1.00	1.00
Married	0.60 (0.47, 0.76)	0.82 (0.53, 1.26)	1.79 (1.46, 2.19)	1.46 (1.11, 1.92)
Formerly Married	0.62 (0.40, 0.97)	0.94 (0.54, 1.62)	3.19 (2.42, 4.19)	1.97 (1.36, 2.86)
**Residential Setting**				
Rural (Reference)	1.00	1.00	1.00	1.00
Urban	0.85 (0.64, 1.12)	0.96 (0.62, 1.48)	2.33 (1.91, 2.83)	1.30 (0.97, 1.76)
**Region**				
Western (Reference)	1.00	1.00	1.00	1.00
Eastern	1.32 (0.81, 2.15)	1.10 (0.67, 1.81)	0.64 (0.47, 0.87)	1.27 (0.89, 1.83)
Northern	1.29 (0.84, 1.96)	1.14 (0.70, 1.85)	0.44 (0.33, 0.58)	1.06 (0.75, 1.49)
Southern	1.24 (0.80, 1.92)	1.05 (0.63, 1.77)	0.66 (0.50, 0.88)	1.46 (1.04, 2.06)
**Level of Education**				
No Education (Reference)	1.00	1.00	1.00	1.00
Primary	1.27 (0.98, 1.65)	1.02 (0.75, 1.40)	0.91 (0.71, 1.15)	1.05 (0.78, 1.39)
Secondary	1.28 (0.99, 1.65)	0.91 (0.62, 1.32)	1.07 (0.85, 1.34)	1.11 (0.84, 1.47)
Higher	0.73 (0.38, 1.42)	1.09 (0.42, 2.80)	1.59 (1.05, 2.40)	0.57 (0.32, 1.03)
**Wealth Quintile**				
Poorest (Reference)	1.00	1.00	1.00	1.00
Poorer	0.99 (0.71, 1.37)	0.97 (0.70, 1.34)	1.37 (1.00, 1.87)	1.42 (1.03, 1.95)
Middle	0.89 (0.65, 1.24)	0.88 (0.64, 1.23)	1.52 (1.11, 2.07)	1.54 (1.10, 2.16)
Richer	0.94 (0.67, 1.32)	0.94 (0.65, 1.36)	2.29 (1.73, 3.03)	1.99 (1.43, 2.77)
Richest	0.75 (0.51, 1.11)	0.71 (0.39, 1.29)	3.66 (2.78, 4.83)	2.89 (1.89, 4.40)
**Occupation**				
Not Working (Reference)	1.00	1.00	1.00	1.00
Nonmanual	0.25 (0.12, 0.52)	0.34 (0.15, 0.76)	1.81 (1.30, 2.52)	0.92 (0.64, 1.31)
Manual	0.44 (0.31, 0.60)	0.54 (0.38, 0.76)	1.33 (1.08, 1.63)	0.84 (0.67, 1.05)
Agricultural	0.72 (0.56, 0.93)	0.80 (0.60, 1.07)	0.61 (0.48, 0.77)	0.57 (0.44, 0.74)

	UNDERWEIGHT		OVERWEIGHT/OBESITY	
**Media Exposure**				
Not Exposed (Reference)	1.00	1.00	1.00	1.00
Exposed to one media source	1.14 (0.92, 1.43)	1.14 (0.90, 1.44)	1.32 (1.06, 1.64)	1.17 (0.94, 1.46)
Exposed to two media sources	1.00 (0.72, 1.39)	1.03 (0.72, 1.48)	2.06 (1.61, 2.64)	1.39 (1.04, 1.86)
Exposed to three media sources	1.04 (0.68, 1.58)	1.07 (0.69, 1.64)	2.60 (1.97, 3.45)	1.94 (1.39, 2.71)
**Hormonal Contraceptive Use**				
No (Reference)	1.00	1.00	1.00	1.00
Yes	0.82 (0.64, 1.06)	0.87 (0.66, 1.14)	1.14 (0.93, 1.40)	0.96 (0.76, 1.21)

Overall Model Fit: F (88, 320) = 7.57, *P*=0.0000.

Table S52: Unadjusted and adjusted relative risk ratio, of underweight and overweight/obesity among women of child-bearing age in Mozambique.

VARIABLES	UNDERWEIGHT		OVERWEIGHT/OBESITY	
	Unadjusted RRR (95% CI)	Adjusted RRR (95% CI)	Unadjusted RRR (95% CI)	Adjusted RRR (95% CI)
**Age**				
15-19 (Reference)	1.00	1.00	1.00	1.00
20-24	0.43 (0.34, 0.54)	0.63 (0.46, 0.86)	1.52 (1.20, 1.93)	1.46 (1.10, 1.94)
25-29	0.47 (0.34, 0.64)	0.71 (0.47, 1.06)	2.67 (2.15, 3.31)	2.67 (2.00, 3.58)
30-34	0.43 (0.32, 0.58)	0.65 (0.43, 0.99)	3.34 (2.66, 4.20)	3.87 (2.80, 5.33)
35-39	0.48 (0.34, 0.66)	0.69 (0.43, 1.11)	3.49 (2.84, 4.29)	4.71 (3.46, 6.41)
40-44	0.63 (0.45, 0.88)	0.90 (0.60, 1.36)	4.23 (3.30, 5.42)	6.53 (4.52, 9.43)
45-49	0.84 (0.61, 1.17)	1.20 (0.77, 1.87)	4.78 (3.82, 6.00)	8.84 (6.10, 12.8)
**Ethnicity**				
Xichangana (Reference)	1.00	1.00	1.00	1.00
Emakhuwa	1.21 (0.88, 1.68)	1.08 (0.63, 1.85)	0.31 (0.23, 0.40)	0.67 (0.45, 0.99)
Português	1.63 (1.18, 2.25)	1.34 (0.90, 2.00)	0.92 (0.75, 1.14)	0.88 (0.72, 1.08)
Cisena	1.99 (1.45, 2.72)	1.14 (0.70, 1.86)	0.13 (0.09, 0.19)	0.43 (0.29, 0.63)
Elomwe	2.81 (1.91, 4.11)	1.17 (0.68, 2.01)	0.11 (0.07, 0.18)	0.54 (0.29, 1.03)
Echuwabo	1.58 (0.95, 2.64)	0.79 (0.44, 1.43)	0.25 (0.16, 0.40)	0.77 (0.46, 1.29)
Cinyanja	1.39 (0.96, 2.03)	0.88 (0.52, 1.50)	0.21 (0.15, 0.30)	0.65 (0.41, 1.02)
Cindau	1.40 (0.99, 1.98)	0.93 (0.54, 1.58)	0.34 (0.26, 0.45)	0.88 (0.59, 1.31)
Xitswa	0.86 (0.54, 1.34)	1.01 (0.54, 1.89)	0.72 (0.54, 0.96)	0.84 (0.60, 1.17)
Cinyungwe	2.02 (1.33, 3.08)	1.35 (0.72, 2.53)	0.26 (0.15, 0.42)	0.50 (0.30, 0.85)
Ciyao	1.66 (0.93, 2.95)	1.10 (0.48, 2.55)	0.22 (0.14, 0.35)	0.59 (0.30, 1.14)
Others	1.36 (1.00, 1.84)	1.14 (0.75, 1.72)	0.63 (0.52, 0.76)	0.93 (0.76, 1.15)
**Religion**				
Catholics (Reference)	1.00	1.00	1.00	1.00
Other Christians	0.92 (0.73, 1.16)	1.00 (0.76, 1.31)	1.16 (0.98, 1.38)	0.95 (0.80, 1.13)
Islam	0.88 (0.65, 1.18)	1.00 (0.70, 1.43)	0.67 (0.54, 0.83)	0.77 (0.59, 1.01)
Others	1.17 (0.64, 2.14)	1.33 (0.69, 2.56)	1.43 (1.04, 1.95)	0.83 (0.60, 1.14)
No Religion	1.04 (0.78, 1.40)	1.02 (0.74, 1.41)	0.73 (0.56, 0.96)	0.93 (0.70, 1.24)
**Parity**				
0 (Reference)	1.00	1.00	1.00	1.00
1	0.51 (0.39, 0.68)	0.81 (0.59, 1.13)	1.11 (0.89, 1.39)	0.75 (0.57, 0.99)
2	0.49 (0.37, 0.65)	0.86 (0.58, 1.28)	1.64 (1.35, 1.99)	0.94 (0.71, 1.23)
3	0.47 (0.34, 0.65)	0.80 (0.52, 1.23)	2.10 (1.70, 2.60)	1.03 (0.76, 1.40)
4	0.48 (0.35, 0.66)	0.79 (0.53, 1.18)	1.81 (1.47, 2.23)	0.88 (0.64, 1.21)
5	0.59 (0.41, 0.84)	0.85 (0.53, 1.37)	1.91 (1.53, 2.37)	0.87 (0.63, 1.19)
6+	0.59 (0.45, 0.78)	0.71 (0.48, 1.06)	1.46 (1.22, 1.75)	0.76 (0.57, 1.02)
**Marital Status**				
Single (Reference)	1.00	1.00	1.00	1.00
Married	0.51 (0.42, 0.62)	0.57 (0.44, 0.75)	1.44 (1.24, 1.68)	1.33 (1.05, 1.67)
Formerly Married	0.50 (0.38, 0.65)	0.55 (0.40, 0.76)	1.51 (1.24, 1.83)	0.97 (0.72, 1.31)
**Residential Setting**				
Rural (Reference)	1.00	1.00	1.00	1.00
Urban	0.77 (0.63, 0.93)	0.99 (0.77, 1.28)	3.02 (2.56, 3.56)	0.99 (0.79, 1.24)

	UNDERWEIGHT		OVERWEIGHT/OBESITY	
**Region**				
Maputo Provincia (Reference)	1.00	1.00	1.00	1.00
Niassa	1.39 (0.90, 2.14)	1.49 (0.79, 2.80)	0.26 (0.19, 0.37)	1.21 (0.73, 1.99)
Cabo Delgado	1.32 (0.87, 2.00)	1.03 (0.56, 1.91)	0.26 (0.19, 0.35)	1.17 (0.76, 1.79)
Nampula	0.83 (0.51, 1.34)	0.67 (0.35, 1.29)	0.30 (0.20, 0.44)	1.25 (0.78, 2.00)
Zambezia	1.99 (1.39, 2.86)	1.63 (0.95, 2.79)	0.12 (0.08, 0.18)	0.51 (0.31, 0.83)
Tete	1.35 (0.91, 1.99)	1.32 (0.74, 2.38)	0.21 (0.16, 0.28)	0.80 (0.53, 1.21)
Manica	1.27 (0.84, 1.90)	1.29 (0.78, 2.15)	0.31 (0.24, 0.41)	0.73 (0.54, 1.01)
Sofala	1.43 (1.00, 2.04)	1.14 (0.68, 1.89)	0.22 (0.15, 0.33)	0.65 (0.42, 1.01)
Inhambane	0.71 (0.46, 1.09)	0.61 (0.34, 1.10)	0.61 (0.47, 0.80)	1.24 (0.92, 1.68)
Gaza	0.83 (0.55, 1.26)	0.90 (0.57, 1.40)	0.52 (0.40, 0.68)	0.83 (0.64, 1.08)
Maputo Cidade	1.06 (0.74, 1.53)	1.00 (0.67, 1.49)	1.15 (0.94, 1.42)	0.96 (0.80, 1.16)
**Level of Education**				
No Education (Reference)	1.00	1.00	1.00	1.00
Primary	1.05 (0.85, 1.28)	0.97 (0.79, 1.18)	1.63 (1.40, 1.91)	1.25 (1.03, 1.50)
Secondary	0.79 (0.62, 1.00)	0.67 (0.49, 0.93)	2.79 (2.32, 3.35)	1.38 (1.08, 1.76)
Higher	1.14 (0.62, 2.12)	1.05 (0.55, 2.03)	8.23 (5.82, 11.6)	1.91 (1.17, 3.10)
**Wealth Quintile**				
Poorest (Reference)	1.00	1.00	1.00	1.00
Poorer	0.91 (0.72, 1.15)	0.94 (0.74, 1.20)	1.17 (0.77, 1.77)	0.99 (0.65, 1.52)
Middle	0.59 (0.45, 0.77)	0.66 (0.51, 0.86)	2.02 (1.42, 2.86)	1.56 (1.07, 2.26)
Richer	0.53 (0.39, 0.71)	0.71 (0.52, 0.96)	3.49 (2.46, 4.95)	2.27 (1.56, 3.30)
Richest	0.60 (0.46, 0.77)	0.76 (0.51, 1.14)	9.76 (6.89, 13.8)	5.17 (3.41, 7.85)
**Occupation**				
Not Working (Reference)	1.00	1.00	1.00	1.00
Nonmanual	0.65 (0.50, 0.84)	0.92 (0.69, 1.22)	2.73 (2.35, 3.17)	1.07 (0.91, 1.26)
Manual	0.54 (0.21, 1.41)	0.78 (0.30, 1.97)	2.63 (1.73, 4.02)	1.14 (0.73, 1.80)
Agricultural	1.16 (0.97, 1.38)	1.36 (1.13, 1.65)	0.69 (0.58, 0.84)	0.83 (0.68, 1.02)
**Media Exposure**				
Not Exposed (Reference)	1.00	1.00	1.00	1.00
Exposed to one media source	0.79 (0.64, 0.97)	0.75 (0.59, 0.94)	1.12 (0.94, 1.32)	1.05 (0.87, 1.27)
Exposed to two media sources	0.66 (0.51, 0.87)	0.76 (0.55, 1.06)	2.43 (2.02, 2.92)	1.18 (0.94, 1.49)
Exposed to three media sources	0.75 (0.56, 1.01)	0.85 (0.56, 1.29)	3.69 (3.00, 4.53)	1.25 (0.97, 1.62)
**Hormonal Contraceptive Use**				
No (Reference)	1.00	1.00	1.00	1.00
Yes	0.54 (0.38, 0.77)	0.86 (0.60, 1.24)	2.41 (2.09, 2.77)	1.35 (1.16, 1.58)

Overall Model Fit: F (108, 482) = 13.93, *P*=0.0000.

Table S53: Unadjusted and adjusted relative risk ratio, of underweight and overweight/obesity among women of child-bearing age in Malawi.

VARIABLES	UNDERWEIGHT		OVERWEIGHT/OBESITY	
	Unadjusted RRR (95% CI)	Adjusted RRR (95% CI)	Unadjusted RRR (95% CI)	Adjusted RRR (95% CI)
**Age**				
15-19 (Reference)	1.00	1.00	1.00	1.00
20-24	0.39 (0.27, 0.56)	0.76 (0.49, 1.18)	2.03 (1.45, 2.84)	2.18 (1.43, 3.33)
25-29	0.45 (0.30, 0.67)	1.02 (0.58, 1.81)	3.61 (2.63, 4.96)	4.41 (2.69, 7.25)
30-34	0.61 (0.41, 0.92)	1.40 (0.78, 2.53)	5.27 (4.01, 6.95)	7.58 (4.73, 12.1)
35-39	0.49 (0.33, 0.74)	1.22 (0.66, 2.23)	5.66 (4.06, 7.89)	8.98 (5.38, 15.0)
40-44	0.60 (0.39, 0.93)	1.34 (0.72, 2.49)	4.63 (3.28, 6.55)	7.47 (4.45, 12.5)
45-49	0.55 (0.33, 0.91)	1.20 (0.59, 2.44)	5.37 (3.84, 7.49)	8.81 (5.14, 15.1)
**Ethnicity**				
Chewa (Reference)	1.00	1.00	1.00	1.00
Tombuka	0.88 (0.55, 1.42)	0.88 (0.44, 1.79)	1.43 (1.07, 1.90)	1.01 (0.64, 1.61)
Lomwe	1.37 (1.02, 1.85)	1.30 (0.84, 2.00)	1.12 (0.90, 1.40)	0.95 (0.72, 1.25)
Tonga	1.30 (0.69, 2.46)	1.31 (0.58, 2.97)	1.42 (0.88, 2.29)	0.94 (0.50, 1.75)
Yao	1.08 (0.73, 1.58)	0.66 (0.40, 1.09)	1.17 (0.89, 1.54)	1.05 (0.72, 1.54)
Sena	0.80 (0.45, 1.42)	0.77 (0.40, 1.48)	0.98 (0.65, 1.48)	0.75 (0.48, 1.16)
Nkhonde	1.01 (0.45, 2.28)	0.96 (0.38, 2.47)	1.93 (0.97, 3.83)	1.15 (0.55, 2.38)
Ngoni	1.28 (0.87, 1.90)	1.34 (0.89, 2.02)	1.73 (1.37, 2.19)	1.45 (1.13, 1.86)
Mang'anja	1.30 (0.69, 2.46)	1.27 (0.62, 2.61)	1.39 (0.78, 2.48)	0.88 (0.50, 1.53)
Nyanja	0.64 (0.22, 1.90)	0.62 (0.21, 1.84)	1.42 (0.80, 2.55)	1.07 (0.54, 2.12)
Others	0.25 (0.10, 0.65)	0.29 (0.10, 0.84)	1.91 (1.23, 2.98)	1.36 (0.80, 2.30)
**Religion**				
Catholics (Reference)	1.00	1.00	1.00	1.00
Other Christians	0.86 (0.67, 1.11)	0.94 (0.72, 1.21)	1.04 (0.87, 1.25)	1.03 (0.85, 1.25)
Islam	1.11 (0.75, 1.64)	1.63 (1.01, 2.62)	0.87 (0.62, 1.21)	0.95 (0.63, 1.45)
Others	0.89 (0.10, 8.37)	0.99 (0.09, 11.1)	0.87 (0.15, 4.96)	0.84 (0.18, 3.82)
No Religion	1.84 (0.57, 6.00)	2.08 (0.63, 6.95)	1.36 (0.39, 4.68)	1.57 (0.45, 5.50)
**Parity**				
0 (Reference)	1.00	1.00	1.00	1.00
1	0.45 (0.31, 0.66)	0.91 (0.57, 1.44)	1.32 (1.00, 1.72)	0.61 (0.39, 0.94)
2	0.36 (0.24, 0.55)	0.76 (0.43, 1.36)	1.91 (1.44, 2.54)	0.58 (0.35, 0.96)
3	0.54 (0.35, 0.83)	0.97 (0.51, 1.84)	2.23 (1.68, 2.97)	0.50 (0.29, 0.83)
4	0.63 (0.40, 0.99)	0.96 (0.49, 1.88)	2.36 (1.78, 3.12)	0.47 (0.28, 0.80)
5	0.45 (0.29, 0.70)	0.63 (0.31, 1.27)	1.84 (1.35, 2.50)	0.37 (0.21, 0.65)
6+	0.49 (0.34, 0.71)	0.64 (0.33, 1.22)	2.44 (1.89, 3.14)	0.50 (0.29, 0.86)
**Marital Status**				
Single (Reference)	1.00	1.00	1.00	1.00
Married	0.43 (0.33, 0.56)	0.50 (0.32, 0.80)	2.13 (1.71, 2.67)	1.31 (0.83, 2.07)
Formerly Married	0.56 (0.39, 0.82)	0.53 (0.32, 0.88)	2.05 (1.55, 2.72)	1.35 (0.84, 2.16)
**Residential Setting**				
Rural (Reference)	1.00	1.00	1.00	1.00
Urban	1.11 (0.78, 1.59)	1.25 (0.81, 1.93)	2.78 (2.35, 3.29)	1.41 (1.13, 1.76)
**Region**				
Central (Reference)	1.00	1.00	1.00	1.00
Northern	0.89 (0.62, 1.29)	1.09 (0.58, 2.05)	1.23 (0.99, 1.52)	1.11 (0.73, 1.68)
Southern	1.21 (0.95, 1.55)	1.08 (0.74, 1.58)	1.00 (0.85, 1.18)	1.02 (0.82, 1.26)
**Level of Education**				
No Education (Reference)	1.00	1.00	1.00	1.00
Primary	1.04 (0.75, 1.45)	0.95 (0.67, 1.36)	0.70 (0.56, 0.86)	0.77 (0.60, 0.99)
Secondary	0.95 (0.64, 1.42)	0.75 (0.45, 1.24)	1.21 (0.95, 1.53)	0.86 (0.63, 1.17)
Higher	0.29 (0.05, 1.60)	0.24 (0.04, 1.43)	2.21 (1.42, 3.45)	0.76 (0.42, 1.37)

	UNDERWEIGHT		OVERWEIGHT/OBESITY	
**Wealth Quintile**				
Poorest (Reference)	1.00	1.00	1.00	1.00
Poorer	0.79 (0.56, 1.12)	0.77 (0.54, 1.11)	1.05 (0.75, 1.46)	1.07 (0.77, 1.50)
Middle	0.87 (0.63, 1.20)	0.83 (0.59, 1.17)	1.57 (1.13, 2.18)	1.51 (1.08, 2.11)
Richer	0.96 (0.68, 1.35)	0.92 (0.63, 1.34)	2.23 (1.62, 3.07)	1.96 (1.42, 2.71)
Richest	0.94 (0.66, 1.35)	0.94 (0.60, 1.47)	4.20 (3.11, 5.67)	2.95 (2.08, 4.19)
**Occupation**				
Not Working (Reference)	1.00	1.00	1.00	1.00
Nonmanual	0.57 (0.37, 0.89)	0.85 (0.52, 1.39)	2.28 (1.79, 2.90)	1.08 (0.83, 1.42)
Manual	0.87 (0.60, 1.24)	1.16 (0.80, 1.69)	1.53 (1.23, 1.91)	1.15 (0.90, 1.48)
Agricultural	0.78 (0.60, 1.01)	1.04 (0.77, 1.39)	0.73 (0.61, 0.89)	0.71 (0.58, 0.88)
**Media Exposure**				
Not Exposed (Reference)	1.00	1.00	1.00	1.00
Exposed to one media source	0.72 (0.54, 0.97)	0.76 (0.56, 1.04)	1.32 (1.11, 1.57)	1.04 (0.87, 1.26)
Exposed to two media sources	0.75 (0.53, 1.07)	0.74 (0.51, 1.09)	1.96 (1.58, 2.42)	1.17 (0.91, 1.49)
Exposed to three media sources	0.66 (0.41, 1.06)	0.64 (0.37, 1.09)	2.41 (1.90, 3.07)	1.05 (0.77, 1.43)
**Hormonal Contraceptives use**				
No (Reference)	1.00	1.00	1.00	1.00
Yes	0.49 (0.38, 0.64)	0.67 (0.50, 0.90)	1.23 (1.06, 1.44)	1.32 (1.10, 1.60)

Overall Model Fit: F (90, 705) = 9.22, *P*=0.0000.

Table S54: Unadjusted and adjusted relative risk ratio, of underweight and overweight/obesity among women of child-bearing age in Ivory Coast.

VARIABLES	UNDERWEIGHT		OVERWEIGHT/OBESITY	
	Unadjusted RRR (95% CI)	Adjusted RRR (95% CI)	Unadjusted RRR (95% CI)	Adjusted RRR (95% CI)
**Age**				
15-19 (Reference)	1.00	1.00	1.00	1.00
20-24	0.38 (0.25, 0.57)	0.65 (0.40, 1.05)	1.88 (1.31, 2.71)	1.84 (1.28, 2.64)
25-29	0.34 (0.23, 0.52)	0.88 (0.49, 1.59)	2.74 (1.88, 3.99)	2.80 (1.83, 4.31)
30-34	0.37 (0.21, 0.66)	1.20 (0.58, 2.48)	4.98 (3.38, 7.35)	5.24 (3.15, 8.71)
35-39	0.38 (0.23, 0.65)	1.47 (0.74, 2.94)	4.44 (3.05, 6.46)	5.14 (3.10, 8.53)
40-44	0.41 (0.24, 0.70)	1.68 (0.78, 3.65)	5.11 (3.58, 7.28)	6.78 (4.08, 11.3)
45-49	0.37 (0.21, 0.65)	1.53 (0.71, 3.28)	5.13 (3.62, 7.26)	6.42 (3.83, 10.8)
**Ethnicity**				
Akan (Reference)	1.00	1.00	1.00	1.00
Krou	0.95 (0.58, 1.55)	0.90 (0.48, 1.67)	0.91 (0.65, 1.28)	0.80 (0.55, 1.17)
Mandé du nord	1.13 (0.70, 1.81)	1.29 (0.68, 2.46)	1.58 (1.15, 2.15)	0.92 (0.57, 1.49)
Mandé du sud	0.77 (0.44, 1.35)	0.74 (0.38, 1.45)	0.80 (0.54, 1.20)	0.84 (0.50, 1.41)
Voltaïque/Gur	1.23 (0.79, 1.94)	1.25 (0.77, 2.03)	0.78 (0.54, 1.12)	0.77 (0.48, 1.26)
Autres nationalités	1.14 (0.75, 1.72)	1.30 (0.80, 2.10)	1.04 (0.77, 1.39)	0.84 (0.57, 1.24)
**Religion**				
Catholics (Reference)	1.00	1.00	1.00	1.00
Other Christians	1.07 (0.67, 1.71)	1.21 (0.76, 1.94)	0.98 (0.73, 1.30)	1.01 (0.74, 1.36)
Islam	1.14 (0.74, 1.77)	1.23 (0.78, 1.94)	1.26 (0.97, 1.63)	1.33 (0.92, 1.93)
Traditionalist	1.04 (0.43, 2.52)	1.37 (0.50, 3.76)	0.77 (0.42, 1.39)	1.39 (0.69, 2.79)
Others	0.80 (0.18, 3.46)	1.05 (0.21, 5.21)	0.94 (0.38, 2.34)	1.28 (0.54, 3.02)
No Religion	0.81 (0.45, 1.47)	1.01 (0.54, 1.88)	0.53 (0.37, 0.75)	0.93 (0.63, 1.36)
**Parity**				
0 (Reference)	1.00	1.00	1.00	1.00
1	0.61 (0.39, 0.95)	0.87 (0.53, 1.43)	1.33 (0.95, 1.85)	0.94 (0.60, 1.47)
2	0.36 (0.23, 0.57)	0.62 (0.35, 1.11)	1.36 (0.93, 1.98)	0.82 (0.50, 1.34)
3	0.34 (0.17, 0.68)	0.57 (0.27, 1.23)	1.96 (1.42, 2.69)	1.05 (0.64, 1.73)
4	0.56 (0.32, 0.99)	0.86 (0.41, 1.82)	3.13 (2.10, 4.66)	1.91 (1.03, 3.56)
5	0.26 (0.14, 0.47)	0.39 (0.18, 0.85)	1.52 (1.04, 2.24)	0.69 (0.38, 1.24)
6+	0.30 (0.19, 0.48)	0.38 (0.19, 0.77)	1.91 (1.45, 2.51)	1.02 (0.59, 1.76)
**Marital Status**				
Single (Reference)	1.00	1.00	1.00	1.00
Married	0.36 (0.27, 0.47)	0.46 (0.29, 0.73)	1.76 (1.44, 2.15)	1.11 (0.77, 1.61)
Formerly Married	0.47 (0.25, 0.88)	0.57 (0.26, 1.25)	2.66 (1.85, 3.83)	1.29 (0.82, 2.03)
**Residential Setting**				
Rural (Reference)	1.00	1.00	1.00	1.00
Urban	1.31 (0.98, 1.76)	1.59 (0.82, 3.09)	2.73 (2.23, 3.35)	1.67 (1.15, 2.43)
**Region**				
Ville d'Abidjan (Reference)	1.00	1.00	1.00	1.00
Centre	0.77 (0.48, 1.23)	0.82 (0.47, 1.45)	0.40 (0.25, 0.63)	0.73 (0.45, 1.17)
Centre-Est	1.20 (0.68, 2.12)	1.29 (0.69, 2.42)	0.46 (0.30, 0.70)	0.84 (0.52, 1.34)
Centre-Nord	0.69 (0.38, 1.25)	0.65 (0.36, 1.17)	0.46 (0.31, 0.66)	0.68 (0.46, 0.99)
Centre-Ouest	0.84 (0.46, 1.54)	1.02 (0.53, 1.95)	0.53 (0.38, 0.75)	1.07 (0.72, 1.58)
Nord	1.05 (0.60, 1.82)	1.42 (0.77, 2.60)	0.26 (0.17, 0.40)	0.50 (0.31, 0.81)
Nord-est	1.24 (0.79, 1.93)	1.08 (0.61, 1.92)	0.21 (0.14, 0.34)	0.49 (0.28, 0.85)
Nord-Ouest	1.03 (0.65, 1.63)	1.33 (0.73, 2.42)	0.36 (0.24, 0.54)	0.71 (0.42, 1.22)
Ouest	0.77 (0.42, 1.41)	1.00 (0.48, 2.08)	0.40 (0.27, 0.58)	0.88 (0.56, 1.37)
Sud sans Abidjan	0.75 (0.41, 1.38)	0.76 (0.38, 1.49)	0.54 (0.37, 0.79)	0.94 (0.61, 1.46)
Sud-ouest	0.58 (0.30, 1.13)	0.63 (0.33, 1.21)	0.31 (0.20, 0.50)	0.76 (0.47, 1.24)

	UNDERWEIGHT		OVERWEIGHT/OBESITY	
**Level of Education**				
No Education (Reference)	1.00	1.00	1.00	1.00
Primary	1.53 (1.05, 2.23)	1.51 (1.02, 2.24)	1.32 (1.08, 1.61)	1.33 (1.04, 1.71)
Secondary	1.80 (1.25, 2.59)	1.35 (0.79, 2.31)	1.20 (0.93, 1.55)	1.10 (0.76, 1.60)
Higher	0.05 (0.01, 0.40)	0.04 (0.01, 0.38)	2.02 (1.02, 4.00)	1.24 (0.60, 2.56)
**Wealth Quintile**				
Poorest (Reference)	1.00	1.00	1.00	1.00
Poorer	1.00 (0.65, 1.56)	0.88 (0.56, 1.39)	1.55 (1.06, 2.27)	1.33 (0.86, 2.05)
Middle	0.92 (0.58, 1.45)	0.54 (0.30, 0.96)	1.95 (1.33, 2.87)	1.44 (0.91, 2.29)
Richer	0.87 (0.56, 1.37)	0.41 (0.21, 0.83)	3.41 (2.42, 4.79)	1.71 (1.08, 2.71)
Richest	1.23 (0.82, 1.85)	0.51 (0.25, 1.05)	4.07 (2.89, 5.74)	2.16 (1.28, 3.63)
**Occupation**				
Not Working (Reference)	1.00	1.00	1.00	1.00
Nonmanual	0.49 (0.33, 0.72)	0.56 (0.39, 0.82)	2.10 (1.67, 2.64)	1.31 (1.01, 1.70)
Manual	1.02 (0.55, 1.88)	1.02 (0.53, 1.97)	1.29 (0.84, 2.00)	1.21 (0.74, 1.97)
Agricultural	0.58 (0.41, 0.84)	0.82 (0.52, 1.28)	0.64 (0.47, 0.86)	0.74 (0.51, 1.07)
**Media Exposure**				
Not Exposed (Reference)	1.00	1.00	1.00	1.00
Exposed to one media source	1.11 (0.74, 1.66)	0.98 (0.63, 1.54)	1.68 (1.29, 2.18)	1.10 (0.81, 1.49)
Exposed to two media sources	1.08 (0.74, 1.58)	0.82 (0.52, 1.29)	2.21 (1.73, 2.83)	1.23 (0.91, 1.66)
Exposed to three media sources	1.01 (0.60, 1.69)	0.69 (0.34, 1.39)	2.53 (1.80, 3.54)	1.27 (0.79, 2.03)
**Hormonal Contraceptive Use**				
No (Reference)	1.00	1.00	1.00	1.00
Yes	0.61 (0.33, 1.14)	0.83 (0.43, 1.59)	1.84 (1.41, 2.41)	1.38 (0.99, 1.90)

Overall Model Fit: F (98, 233) = 6.43, *P*=0.0000.

Table S55: Unadjusted and adjusted relative risk ratio, of underweight and overweight/obesity among women of child-bearing age in Rwanda.

VARIABLES	UNDERWEIGHT		OVERWEIGHT/OBESITY	
	Unadjusted RRR (95% CI)	Adjusted RRR (95% CI)	Unadjusted RRR (95% CI)	Adjusted RRR (95% CI)
**Age**				
15-19 (Reference)	1.00	1.00	1.00	1.00
20-24	0.30 (0.21, 0.43)	0.35 (0.24, 0.52)	1.47 (1.18, 1.83)	1.32(1.01, 1.73)
25-29	0.45 (0.31, 0.65)	0.60 (0.37, 0.99)	1.66 (1.30, 2.10)	1.23 (0.91, 1.66)
30-34	0.54 (0.37, 0.80)	0.77 (0.43, 1.38)	1.92 (1.53, 2.40)	1.28 (0.92, 1.79)
35-39	0.61 (0.42, 0.91)	0.92 (0.50, 1.71)	2.29 (1.80, 2.91)	1.37 (0.94, 2.00)
40-44	0.55 (0.36, 0.83)	0.79 (0.42, 1.51)	1.95 (1.51, 2.51)	1.29 (0.86, 1.95)
45-49	1.02 (0.71, 1.46)	1.42 (0.76, 2.66)	1.67 (1.26, 2.21)	1.25 (0.81, 1.93)
**Religion**				
Catholics (Reference)	1.00	1.00	1.00	1.00
Other Christians	0.83 (0.66, 1.04)	0.88 (0.69, 1.12)	1.22 (1.06, 1.40)	1.18 (1.02, 1.35)
Islam	0.65 (0.29, 1.45)	0.58 (0.24, 1.42)	1.94 (1.21, 3.12)	1.07 (0.68, 1.67)
No Religion	1.62 (0.34, 7.63)	1.78 (0.38, 8.36)	2.05 (0.73, 5.81)	2.41 (0.82, 7.07)
**Parity**				
0 (Reference)	1.00	1.00	1.00	1.00
1	0.53 (0.36, 0.77)	0.84 (0.48, 1.48)	1.22 (0.99, 1.52)	0.87 (0.64, 1.19)
2	0.64 (0.45, 0.92)	0.91 (0.46, 1.82)	1.57 (1.26, 1.97)	1.11 (0.77, 1.60)
3	0.51 (0.33, 0.79)	0.60 (0.28, 1.27)	1.62 (1.29, 2.03)	1.21 (0.80, 1.81)
4	0.87 (0.59, 1.29)	0.91 (0.42, 1.97)	1.73 (1.38, 2.16)	1.39 (0.93, 2.08)
5	1.07 (0.70, 1.63)	1.02 (0.47, 2.21)	1.94 (1.48, 2.55)	1.59 (1.01, 2.49)
6+	0.69 (0.47, 1.00)	0.60 (0.27, 1.37)	1.60 (1.29, 1.99)	1.47 (0.93, 2.31)
**Marital Status**				
Single (Reference)	1.00	1.00	1.00	1.00
Married	0.63 (0.50, 0.80)	0.97 (0.56, 1.69)	1.76 (1.52, 2.05)	1.58 (1.19, 2.11)
Formerly Married	1.14 (0.82, 1.58)	1.10 (0.62, 1.98)	1.32 (1.05, 1.65)	1.46 (1.02, 2.08)
**Residential Setting**				
Rural (Reference)	1.00	1.00	1.00	1.00
Urban	1.05 (0.81, 1.35)	1.24 (0.88, 1.74)	2.86 (2.43, 3.37)	1.45 (1.14, 1.85)
**Region**				
Kigali City (Reference)	1.00	1.00	1.00	1.00
South	1.39 (0.99, 1.94)	1.31 (0.87, 1.97)	0.38 (0.31, 0.47)	1.02 (0.77, 1.37)
West	0.73 (0.49, 1.09)	0.65 (0.41, 1.03)	0.46 (0.37, 0.59)	1.23 (0.90, 1.70)
North	0.70 (0.45, 1.08)	0.64 (0.39, 1.06)	0.49 (0.38, 0.62)	1.34 (0.98, 1.83)
East	1.15 (0.80, 1.65)	1.24 (0.82, 1.88)	0.48 (0.39, 0.60)	1.26 (0.93, 1.70)
**Level of Education**				
No Education (Reference)	1.00	1.00	1.00	1.00
Primary	0.86 (0.62, 1.20)	0.95 (0.67, 1.34)	1.27 (1.02, 1.58)	1.23 (0.98, 1.56)
Secondary	0.84 (0.58, 1.23)	0.83 (0.52, 1.34)	1.67 (1.30, 2.13)	1.33 (1.00, 1.77)
Higher	1.94 (1.02, 3.68)	2.58 (1.19, 5.57)	4.39 (2.87, 6.74)	1.77 (1.12, 2.80)
**Wealth Quintile**				
Poorest (Reference)	1.00	1.00	1.00	1.00
Poorer	0.68 (0.49, 0.94)	0.68 (0.49, 0.94)	1.42 (1.09, 1.85)	1.33 (1.00, 1.76)
Middle	0.67 (0.48, 0.93)	0.69 (0.50, 0.95)	1.55 (1.21, 1.99)	1.42 (1.09, 1.85)
Richer	0.50 (0.35, 0.73)	0.45 (0.31, 0.67)	2.32 (1.78, 3.01)	2.03 (1.51, 2.72)
Richest	0.65 (0.48, 0.88)	0.43 (0.28, 0.64)	4.54 (3.56, 5.79)	2.72 (1.93, 3.85)
**Occupation**				
Not Working (Reference)	1.00	1.00	1.00	1.00
Nonmanual	0.47 (0.32, 0.69)	0.55 (0.36, 0.83)	2.07 (1.67, 2.55)	1.26 (0.98, 1.61)
Manual	0.71 (0.39, 1.29)	0.79 (0.44, 1.43)	1.27 (0.87, 1.85)	0.97 (0.67, 1.39)
Agricultural	0.54 (0.41, 0.70)	0.51 (0.37, 0.71)	0.68 (0.56, 0.81)	0.67 (0.52, 0.87)

	UNDERWEIGHT		OVERWEIGHT/OBESITY	
**Media Exposure**				
Not Exposed (Reference)	1.00	1.00	1.00	1.00
Exposed to one media source	0.70 (0.52, 0.95)	0.78 (0.56, 1.07)	1.70 (1.32, 2.18)	1.41 (1.08, 1.84)
Exposed to two media sources	0.62 (0.45, 0.85)	0.66 (0.46, 0.95)	2.35 (1.83, 3.00)	1.53 (1.15, 2.03)
Exposed to three media sources	0.86 (0.61, 1.21)	0.88 (0.58, 1.32)	2.71 (2.10, 3.50)	1.51 (1.12, 2.04)
**Hormonal Contraceptive Use**				
No (Reference)	1.00	1.00	1.00	1.00
Yes	0.48 (0.35, 0.65)	0.63 (0.45, 0.88)	1.55 (1.34, 1.80)	1.33 (1.11, 1.61)

Overall Model Fit: F (72, 361) = 8.40, *P*=0.0000.

Table S56: Unadjusted and adjusted relative risk ratio, of underweight and overweight/obesity among women of child-bearing age in Liberia.

VARIABLES	UNDERWEIGHT		OVERWEIGHT/OBESITY	
	Unadjusted RRR (95% CI)	Adjusted RRR (95% CI)	Unadjusted RRR (95% CI)	Adjusted RRR (95% CI)
**Age**				
15-19 (Reference)	1.00	1.00	1.00	1.00
20-24	0.36 (0.22, 0.58)	0.56 (0.32, 1.00)	2.18 (1.17, 4.05)	1.40 (0.67, 2.89)
25-29	0.29 (0.16, 0.53)	0.47 (0.22, 1.01)	4.18 (2.31, 7.55)	2.25 (1.08, 4.69)
30-34	0.45 (0.27, 0.78)	0.66 (0.34, 1.28)	6.13 (3.47, 10.8)	3.00 (1.36, 6.61)
35-39	0.32 (0.16, 0.65)	0.54 (0.18, 1.59)	8.60 (4.97, 14.9)	4.11 (1.86, 9.08)
40-44	0.69 (0.37, 1.29)	1.22 (0.45, 3.33)	10.4 (5.94, 18.2)	5.61 (2.47, 12.7)
45-49	0.86 (0.45, 1.63)	1.53 (0.54, 4.35)	10.8 (6.05, 19.3)	6.38 (2.92, 13.9)
**Ethnicity**				
Kpelle (Reference)	1.00	1.00	1.00	1.00
Bassa	1.29 (0.81, 2.06)	2.10 (1.09, 4.02)	0.78 (0.57, 1.05)	0.59 (0.38, 0.93)
Gio	1.52 (0.73, 3.13)	2.77 (0.50, 15.5)	0.65 (0.34, 1.24)	0.72 (0.35, 1.45)
Gola	0.77 (0.39, 1.52)	1.24 (0.59, 2.59)	1.23 (0.83, 1.84)	0.84 (0.47, 1.51)
Grebo	0.85 (0.47, 1.54)	1.86 (0.61, 5.64)	1.27 (0.78, 2.07)	1.01 (0.44, 2.32)
Kissi	0.77 (0.37, 1.63)	1.01 (0.44, 2.30)	1.47 (0.75, 2.88)	1.28 (0.63, 2.64)
Krahn	0.49 (0.25, 0.94)	0.76 (0.33, 1.72)	1.28 (0.79, 2.06)	1.13 (0.47, 2.75)
Kru	0.85 (0.38, 1.90)	2.03 (0.64, 6.38)	1.89 (1.21, 2.97)	1.15 (0.52, 2.52)
Lorma	0.99 (0.40, 2.44)	1.53 (0.59, 3.94)	1.22 (0.69, 2.15)	1.07 (0.51, 2.25)
Mano	0.26 (0.11, 0.60)	0.48 (0.12, 2.01)	0.81 (0.57, 1.17)	0.90 (0.46, 1.75)
Vai	1.03 (0.33, 3.17)	2.01 (0.57, 7.14)	1.91 (1.13, 3.24)	1.41 (0.70, 2.83)
Others	1.30 (0.71, 2.38)	2.08 (1.08, 4.01)	1.02 (0.70, 1.49)	0.82 (0.50, 1.36)
**Religion**				
Other Christians (Reference)	1.00	1.00	1.00	1.00
Islam	0.80 (0.39, 1.64)	0.67 (0.30, 1.50)	1.23 (0.93, 1.64)	1.07 (0.70, 1.65)
No Religion	0.76 (0.29, 1.98)	0.69 (0.30, 1.57)	0.53 (0.28, 0.98)	0.94 (0.41, 2.13)
**Parity**				
0 (Reference)	1.00	1.00	1.00	1.00
1	0.33 (0.20, 0.54)	0.52 (0.31, 0.87)	2.21 (1.45, 3.36)	1.62 (0.94, 2.80)
2	0.40 (0.25, 0.64)	0.67 (0.40, 1.14)	3.81 (2.38, 6.11)	2.07 (1.08, 3.98)
3	0.40 (0.19, 0.86)	0.64 (0.28, 1.49)	3.78 (2.32, 6.14)	1.92 (0.97, 3.79)
4	0.54 (0.28, 1.03)	0.76 (0.34, 1.66)	4.89 (2.93, 8.18)	2.29 (1.08, 4.86)
5	0.67 (0.37, 1.22)	0.76 (0.33, 1.75)	8.63 (5.69, 13.1)	3.31 (1.64, 6.67)
6+	0.48 (0.30, 0.74)	0.39 (0.17, 0.92)	5.92 (3.77, 9.28)	2.20 (1.05, 4.60)
**Marital Status**				
Single (Reference)	1.00	1.00	1.00	1.00
Married	0.66 (0.46, 0.96)	0.90 (0.55, 1.50)	3.93 (2.98, 5.17)	1.66 (1.10, 2.51)
Formerly Married	0.38 (0.22, 0.66)	0.45 (0.24, 0.86)	4.14 (2.77, 6.19)	1.57 (0.96, 2.55)
**Residential Setting**				
Rural (Reference)	1.00	1.00	1.00	1.00
Urban	0.93 (0.67, 1.29)	1.21 (0.81, 1.81)	1.50 (1.20, 1.88)	1.15 (0.84, 1.56)

	UNDERWEIGHT		OVERWEIGHT/OBESITY	
**Region**				
Montserrado (Reference)	1.00	1.00	1.00	1.00
Bomi	1.05 (0.52, 2.10)	1.12 (0.47, 2.68)	0.98 (0.67, 1.44)	1.67 (0.99, 2.83)
Bong	1.31 (0.80, 2.15)	1.47 (0.72, 3.00)	0.47 (0.29, 0.73)	0.77 (0.41, 1.45)
Gbarpolu	1.60 (0.91, 2.79)	1.23 (0.59, 2.58)	0.79 (0.48, 1.32)	1.38 (0.77, 2.46)
Grand Basa	1.21 (0.66, 2.24)	0.76 (0.34, 1.68)	0.56 (0.41, 0.77)	1.13 (0.72, 1.78)
Grand Cape Mount	0.48 (0.20, 1.17)	0.35 (0.11, 1.19)	0.96 (0.62, 1.46)	1.18 (0.65, 2.14)
Grand Gedeh	0.70 (0.36, 1.37)	0.95 (0.44, 2.06)	0.62 (0.42, 0.93)	0.76 (0.31, 1.85)
Grand Kru	0.43 (0.15, 1.25)	0.21 (0.05, 0.88)	0.71 (0.44, 1.14)	1.21 (0.50, 2.94)
Lofa	0.99 (0.53, 1.83)	0.88 (0.39, 2.02)	0.35 (0.24, 0.51)	0.57 (0.32, 1.04)
Margibi	1.31 (0.74, 2.32)	1.14 (0.54, 2.41)	0.72 (0.52, 0.98)	1.00 (0.66, 1.49)
Maryland	0.70 (0.35, 1.38)	0.40 (0.13, 1.21)	0.66 (0.39, 1.11)	0.88 (0.35, 2.25)
Nimba	0.87 (0.48, 1.59)	0.70 (0.15, 3.21)	0.44 (0.28, 0.69)	0.71 (0.28, 1.81)
River Cess	0.93 (0.53, 1.64)	0.41 (0.17, 0.98)	0.41 (0.28, 0.61)	1.04 (0.56, 1.93)
River Gee	0.83 (0.43, 1.61)	0.60 (0.21, 1.73)	0.63 (0.33, 1.22)	0.92 (0.35, 2.41)
Sinoe	0.93 (0.45, 1.92)	0.56 (0.20, 1.59)	0.84 (0.53, 1.33)	1.06 (0.50, 2.25)
**Level of Education**				
No Education (Reference)	1.00	1.00	1.00	1.00
Primary	1.39 (0.95, 2.04)	0.99 (0.64, 1.52)	0.61 (0.49, 0.77)	1.04 (0.76, 1.44)
Secondary	0.70 (0.40, 1.22)	0.47 (0.23, 0.96)	0.86 (0.66, 1.12)	1.13 (0.77, 1.67)
Higher	0.37 (0.06, 2.43)	0.28 (0.04, 2.00)	2.15 (1.13, 4.06)	1.94 (0.90, 4.18)
**Wealth Quintile**				
Poorest (Reference)	1.00	1.00	1.00	1.00
Poorer	0.91 (0.64, 1.28)	0.86 (0.58, 1.29)	1.22 (0.93, 1.60)	1.27 (0.96, 1.70)
Middle	0.63 (0.42, 0.93)	0.57 (0.36, 0.90)	1.38 (1.05, 1.82)	1.35 (0.98, 1.86)
Richer	0.57 (0.33, 1.00)	0.38 (0.21, 0.70)	1.94 (1.35, 2.77)	1.94 (1.30, 2.89)
Richest	0.92 (0.55, 1.56)	0.54 (0.25, 1.19)	2.51 (1.90, 3.31)	2.60 (1.50, 4.49)
**Occupation**				
Not Working (Reference)	1.00	1.00	1.00	1.00
Nonmanual	0.67 (0.44, 1.01)	0.95 (0.59, 1.52)	2.24 (1.69, 2.97)	1.04 (0.73, 1.47)
Manual	1.51 (0.68, 3.36)	1.60 (0.82, 3.13)	2.35 (1.39, 3.96)	1.24 (0.74, 2.08)
Agricultural	1.04 (0.74, 1.46)	0.97 (0.64, 1.48)	0.88 (0.67, 1.16)	0.67 (0.49, 0.91)
Others	1.47 (0.48, 4.46)	1.00 (0.34, 3.00)	0.77 (0.18, 3.20)	2.14 (0.57, 7.99)
**Media Exposure**				
Not Exposed (Reference)	1.00	1.00	1.00	1.00
Exposed to one media source	1.31 (0.91, 1.89)	1.50 (1.01, 2.20)	1.45 (1.16, 1.80)	1.23 (0.95, 1.59)
Exposed to two media sources	1.03 (0.63, 1.68)	1.31 (0.68, 2.52)	1.78 (1.39, 2.27)	1.37 (0.99, 1.88)
Exposed to three media sources	1.15 (0.65, 2.04)	1.67 (0.79, 3.55)	1.66 (1.22, 2.27)	1.17 (0.67, 2.05)
**Hormonal Contraceptive Use**				
No (Reference)	1.00	1.00	1.00	1.00
Yes	0.52 (0.32, 0.84)	0.71 (0.42, 1.20)	1.13 (0.92, 1.39)	1.09 (0.84, 1.42)

Overall Model Fit: F (114, 179) = 13.28, *P*=0.0000.

Table S57: Unadjusted and adjusted relative risk ratio, of underweight and overweight/obesity among women of child-bearing age in Benin.

VARIABLES	UNDERWEIGHT		OVERWEIGHT/OBESITY	
	Unadjusted RRR (95% CI)	Adjusted RRR (95% CI)	Unadjusted RRR (95% CI)	Adjusted RRR (95% CI)
**Age**				
15-19 (Reference)	1.00	1.00	1.00	1.00
20-24	0.57 (0.46, 0.70)	0.68 (0.53, 0.86)	1.46 (1.21, 1.78)	1.37 (1.09, 1.73)
25-29	0.32 (0.24, 0.41)	0.45 (0.32, 0.62)	2.24 (1.86, 2.70)	2.09 (1.62, 2.69)
30-34	0.39 (0.31, 0.50)	0.58 (0.41, 0.82)	3.06 (2.55, 3.66)	3.03 (2.30, 3.98)
35-39	0.39 (0.29, 0.51)	0.59 (0.39, 0.88)	4.27 (3.57, 5.10)	4.32 (3.27, 5.72)
40-44	0.38 (0.28, 0.51)	0.56 (0.36, 0.86)	4.41 (3.68, 5.28)	4.74 (3.55, 6.33)
45-49	0.41 (0.27, 0.60)	0.59 (0.36, 0.95)	4.81 (3.98, 5.81)	5.28 (3.93, 7.09)
**Ethnicity**				
Fon (Reference)	1.00	1.00	1.00	1.00
Adja	0.92 (0.74, 1.15)	0.91 (0.60, 1.37)	0.83 (0.72, 0.96)	1.23 (0.97, 1.56)
Bariba	0.73 (0.50, 1.04)	1.38 (0.69, 2.73)	0.47 (0.39, 0.57)	0.72 (0.52, 1.00)
Dendi	0.66 (0.38, 1.14)	1.16 (0.59, 2.30)	0.72 (0.57, 0.91)	0.92 (0.67, 1.26)
Yoa	0.53 (0.32, 0.87)	1.35 (0.69, 2.67)	0.41 (0.32, 0.53)	0.84 (0.59, 1.21)
Betamaribe	1.04 (0.78, 1.38)	1.24 (0.60, 2.58)	0.21 (0.16, 0.28)	0.76 (0.53, 1.10)
Peulh	0.80 (0.49, 1.32)	1.56 (0.78, 3.10)	0.42 (0.31, 0.56)	0.70 (0.46, 1.04)
Yoruba	1.11 (0.85, 1.44)	1.11 (0.78, 1.56)	0.85 (0.72, 1.00)	0.79 (0.65, 0.95)
Other Béninois	1.08 (0.29, 4.01)	1.96 (0.51, 7.50)	0.76 (0.31, 1.85)	0.95 (0.44, 2.03)
Other Nationalities	1.07 (0.47, 2.43)	1.33 (0.55, 3.19)	1.54 (1.10, 2.15)	1.27 (0.90, 1.79)
**Religion**				
Catholics (Reference)	1.00	1.00	1.00	1.00
Other Christians	1.08 (0.86, 1.37)	1.12 (0.89, 1.41)	0.89 (0.78, 1.00)	0.95 (0.83, 1.09)
Islam	0.78 (0.60, 1.02)	0.92 (0.66, 1.29)	0.68 (0.60, 0.78)	1.24 (1.03, 1.50)
Traditionalists	1.46 (1.14, 1.85)	1.76 (1.37, 2.27)	0.51 (0.43, 0.61)	0.77 (0.64, 0.94)
Others	0.97 (0.54, 1.73)	0.97 (0.55, 1.71)	0.94 (0.69, 1.28)	1.13 (0.80, 1.58)
No Religion	1.07 (0.78, 1.48)	1.13 (0.82, 1.57)	0.51 (0.40, 0.64)	0.78 (0.61, 1.00)
**Parity**				
0 (Reference)	1.00	1.00	1.00	1.00
1	0.64 (0.50, 0.82)	1.09 (0.79, 1.50)	1.51 (1.28, 1.79)	1.14 (0.87, 1.50)
2	0.53 (0.42, 0.67)	1.08 (0.75, 1.57)	1.90 (1.63, 2.23)	1.10 (0.84, 1.45)
3	0.39 (0.30, 0.52)	0.86 (0.57, 1.28)	2.14 (1.84, 2.49)	1.19 (0.89, 1.58)
4	0.41 (0.30, 0.54)	0.89 (0.57, 1.37)	2.52 (2.15, 2.95)	1.29 (0.97, 1.73)
5	0.44 (0.32, 0.61)	0.96 (0.62, 1.49)	2.12 (1.78, 2.53)	1.10 (0.82, 1.49)
6+	0.45 (0.35, 0.58)	0.97 (0.63, 1.51)	2.09 (1.79, 2.43)	1.09 (0.81, 1.47)
**Marital Status**				
Single (Reference)	1.00	1.00	1.00	1.00
Married	0.47 (0.40, 0.55)	0.77 (0.56, 1.05)	1.97 (1.74, 2.23)	0.96 (0.73, 1.28)
Formerly Married	0.37 (0.23, 0.60)	0.57 (0.32, 1.01)	2.54 (2.08, 3.12)	0.88 (0.63, 1.23)
**Residential Setting**				
Rural (Reference)	1.00	1.00	1.00	1.00
Urban	1.03 (0.86, 1.23)	1.08 (0.88, 1.34)	1.92 (1.73, 2.13)	1.26 (1.12, 1.43)

	UNDERWEIGHT		OVERWEIGHT/OBESITY	
**Region**				
Ouémé (Reference)	1.00	1.00	1.00	1.00
Alibori	0.73 (0.43, 1.24)	0.66 (0.33, 1.32)	0.45 (0.36, 0.56)	0.69 (0.49, 0.98)
Atacora	0.99 (0.69, 1.43)	0.71 (0.32, 1.57)	0.20 (0.16, 0.25)	0.35 (0.25, 0.49)
Atlantique	1.15 (0.82, 1.62)	1.06 (0.75, 1.48)	0.84 (0.69, 1.01)	0.89 (0.71, 1.10)
Borgou	0.56 (0.37, 0.86)	0.45 (0.25, 0.82)	0.56 (0.45, 0.69)	0.71 (0.52, 0.97)
Collines	0.93 (0.64, 1.35)	0.94 (0.61, 1.45)	0.79 (0.64, 0.96)	1.14 (0.90, 1.43)
Couffo	0.72 (0.49, 1.05)	0.65 (0.38, 1.13)	0.33 (0.25, 0.44)	0.41 (0.29, 0.60)
Donga	0.38 (0.24, 0.61)	0.35 (0.19, 0.65)	0.30 (0.23, 0.38)	0.33 (0.23, 0.47)
Littoral	0.73 (0.48, 1.10)	0.57 (0.37, 0.89)	1.38 (1.14, 1.66)	1.01 (0.81, 1.26)
Mono	1.21 (0.83, 1.76)	1.08 (0.63, 1.88)	0.96 (0.79, 1.17)	1.05 (0.77, 1.44)
Pateau	1.38 (0.96, 1.98)	1.35 (0.90, 2.05)	0.72 (0.55, 0.95)	1.03 (0.76, 1.40)
Zou	1.08 (0.80, 1.46)	0.95 (0.69, 1.31)	0.74 (0.61, 0.90)	0.92 (0.74, 1.13)
**Level of Education**				
No Education (Reference)	1.00	1.00	1.00	1.00
Primary	1.26 (1.01, 1.57)	1.10 (0.86, 1.42)	1.62 (1.45, 1.81)	1.26 (1.10, 1.43)
Secondary	1.62 (1.34, 1.95)	1.11 (0.85, 1.46)	1.07 (0.95, 1.20)	1.06 (0.90, 1.25)
Higher	0.88 (0.42, 1.85)	0.90 (0.40, 2.03)	1.66 (1.20, 2.28)	0.92 (0.64, 1.33)
**Wealth Quintile**				
Poorest (Reference)	1.00	1.00	1.00	1.00
Poorer	0.82 (0.64, 1.05)	0.85 (0.66, 1.09)	1.40 (1.18, 1.67)	1.36 (1.15, 1.62)
Middle	0.72 (0.55, 0.94)	0.70 (0.53, 0.93)	1.86 (1.57, 2.19)	1.63 (1.37, 1.95)
Richer	0.80 (0.61, 1.04)	0.79 (0.58, 1.07)	2.78 (2.36, 3.28)	2.04 (1.70, 2.45)
Richest	0.96 (0.73, 1.27)	1.04 (0.72, 1.49)	3.72 (3.16, 4.37)	2.40 (1.94, 2.98)
**Occupation**				
Not Working (Reference)	1.00	1.00	1.00	1.00
Nonmanual	0.54 (0.45, 0.65)	0.78 (0.63, 0.97)	2.06 (1.85, 2.28)	1.23 (1.09, 1.38)
Manual	0.96 (0.73, 1.28)	1.14 (0.84, 1.54)	1.24 (1.01, 1.51)	0.90 (0.73, 1.12)
Agricultural	0.64 (0.50, 0.81)	0.81 (0.63, 1.05)	0.88 (0.75, 1.03)	0.96 (0.81, 1.13)
Others	0.60 (0.39, 0.92)	0.85 (0.54, 1.35)	2.17 (1.75, 2.69)	1.15 (0.92, 1.43)
**Media Exposure**				
Not Exposed (Reference)	1.00	1.00	1.00	1.00
Exposed to one media source	0.84 (0.66, 1.07)	0.82 (0.64, 1.07)	1.04 (0.92, 1.18)	0.82 (0.72, 0.94)
Exposed to two media sources	0.87 (0.71, 1.06)	0.80 (0.63, 1.02)	1.66 (1.48, 1.87)	0.93 (0.81, 1.06)
Exposed to three media sources	1.23 (0.93, 1.64)	0.84 (0.60, 1.19)	1.42 (1.23, 1.65)	0.88 (0.72, 1.08)
**Hormonal Contraceptive Use**				
No (Reference)	1.00	1.00	1.00	1.00
Yes	0.58 (0.37, 0.92)	0.80 (0.50, 1.29)	1.65 (1.36, 2.00)	1.19 (0.95, 1.48)

Overall Model Fit: F (110, 506) = 13.85, *P*=0.0000.

Table S58: Unadjusted and adjusted relative risk ratio, of underweight and overweight/obesity among women of child-bearing age in Kenya.

VARIABLES	UNDERWEIGHT		OVERWEIGHT/OBESITY	
	Unadjusted RRR (95% CI)	Adjusted RRR (95% CI)	Unadjusted RRR (95% CI)	Adjusted RRR (95% CI)
**Age**				
15-19 (Reference)	1.00	1.00	1.00	1.00
20-24	0.59 (0.47, 0.75)	0.83 (0.63, 1.09)	1.82 (1.43, 2.32)	1.08 (0.80, 1.46)
25-29	0.58 (0.46, 0.73)	0.93 (0.66, 1.32)	3.51 (2.78, 4.42)	1.73 (1.25, 2.39)
30-34	0.42 (0.32, 0.55)	0.67 (0.45, 1.00)	5.21 (4.11, 6.60)	2.63 (1.82, 3.79)
35-39	0.55 (0.42, 0.71)	0.79 (0.53, 1.18)	4.83 (3.85, 6.06)	2.85 (1.97, 4.14)
40-44	0.50 (0.37, 0.68)	0.70 (0.44, 1.10)	5.93 (4.70, 7.48)	3.82 (2.66, 5.49)
45-49	0.63 (0.46, 0.86)	0.84 (0.52, 1.34)	6.18 (4.80, 7.94)	4.35 (2.97, 6.37)
**Ethnicity**				
Kikuyu (Reference)	1.00	1.00	1.00	1.00
Embu	0.93 (0.39, 2.21)	1.06 (0.44, 2.57)	0.46 (0.28, 0.75)	0.50 (0.29, 0.86)
Kalenjin	1.45 (1.07, 1.96)	1.10 (0.71, 1.70)	0.26 (0.22, 0.31)	0.42 (0.33, 0.54)
Kamba	1.28 (0.91, 1.81)	1.24 (0.70, 2.20)	0.54 (0.44, 0.67)	0.68 (0.48, 0.96)
Kisii	0.55 (0.32, 0.95)	0.54 (0.26, 1.12)	0.49 (0.40, 0.60)	0.75 (0.54, 1.04)
Luhya	0.85 (0.61, 1.18)	0.63 (0.36, 1.12)	0.44 (0.35, 0.55)	0.77 (0.58, 1.02)
Luo	0.84 (0.60, 1.16)	0.79 (0.45, 1.37)	0.35 (0.29, 0.43)	0.58 (0.42, 0.80)
Maasai	4.04 (2.80, 5.83)	2.34 (1.40, 3.91)	0.37 (0.25, 0.56)	0.76 (0.47, 1.22)
Meru	1.22 (0.80, 1.86)	1.23 (0.64, 2.35)	0.40 (0.31, 0.53)	0.47 (0.31, 0.71)
Mijikenda/Swahili	1.48 (1.03, 2.12)	0.51 (0.28, 0.91)	0.33 (0.25, 0.42)	0.56 (0.38, 0.84)
Somali	4.80 (3.39, 6.78)	1.22 (0.45, 3.31)	0.42 (0.30, 0.60)	1.27 (0.58, 2.79)
Taita/Taveta	0.76 (0.32, 1.79)	0.55 (0.20, 1.46)	1.06 (0.73, 1.54)	0.90 (0.56, 1.45)
Turkana	7.05 (4.85, 10.2)	2.95 (1.75, 4.99)	0.09 (0.05, 0.20)	0.22 (0.10, 0.48)
Samburu	6.66 (4.19, 10.6)	3.18 (1.86, 5.43)	0.06 (0.02, 0.13)	0.10 (0.04, 0.26)
Other	1.94 (1.32, 2.84)	0.85 (0.48, 1.51)	0.39 (0.29, 0.53)	0.72 (0.47, 1.09)
**Religion**				
Catholics (Reference)	1.00	1.00	1.00	1.00
Other Christians	0.81 (0.66, 0.98)	0.89 (0.72, 1.09)	0.93 (0.80, 1.08)	0.94 (0.82, 1.09)
Islam	2.35 (1.81, 3.03)	1.70 (1.11, 2.59)	0.90 (0.72, 1.13)	1.19 (0.84, 1.67)
Others	1.38 (0.42, 4.54)	1.41 (0.51, 3.94)	1.06 (0.54, 2.10)	0.65 (0.36, 1.18)
No Religion	1.46 (0.94, 2.27)	1.19 (0.75, 1.89)	0.36 (0.23, 0.55)	0.62 (0.38, 1.02)
**Parity**				
0 (Reference)	1.00	1.00	1.00	1.00
1	0.59 (0.45, 0.77)	0.93 (0.67, 1.28)	2.22 (1.83, 2.68)	1.23 (0.93, 1.63)
2	0.54 (0.42, 0.71)	0.90 (0.63, 1.28)	3.48 (2.92, 4.16)	1.57 (1.16, 2.13)
3	0.65 (0.50, 0.86)	0.98 (0.66, 1.45)	3.64 (2.97, 4.45)	1.54 (1.11, 2.14)
4	0.66 (0.50, 0.87)	0.85 (0.57, 1.28)	3.15 (2.57, 3.86)	1.40 (0.98, 1.98)
5	0.67 (0.50, 0.91)	0.99 (0.63, 1.56)	3.01 (2.42, 3.74)	1.38 (0.95, 2.00)
6+	0.74 (0.59, 0.93)	0.81 (0.53, 1.25)	1.99 (1.66, 2.38)	1.09 (0.76, 1.57)
**Marital Status**				
Single (Reference)	1.00	1.00	1.00	1.00
Married	0.65 (0.56, 0.76)	0.74 (0.57, 0.96)	2.74 (2.39, 3.14)	1.42 (1.13, 1.79)
Formerly Married	0.66 (0.51, 0.86)	0.78 (0.55, 1.12)	2.59 (2.14, 3.15)	1.16 (0.88, 1.54)
**Residential Setting**				
Rural (Reference)	1.00	1.00	1.00	1.00
Urban	0.60 (0.50, 0.72)	1.03 (0.84, 1.27)	2.06 (1.82, 2.34)	1.13 (0.98, 1.31)

	UNDERWEIGHT		OVERWEIGHT/OBESITY	
**Region**				
Nairobi (Reference)	1.00	1.00	1.00	1.00
Coast	3.53 (1.97, 6.31)	2.08 (1.10, 3.95)	0.61 (0.45, 0.82)	1.27 (0.86, 1.88)
North Eastern	10.1 (5.56, 18.4)	1.24 (0.44, 3.53)	0.39 (0.25, 0.59)	0.69 (0.33, 1.45)
Eastern	2.98 (1.67, 5.30)	1.40 (0.75, 2.62)	0.54 (0.40, 0.71)	1.17 (0.79, 1.74)
Central	2.43 (1.33, 4.44)	1.95 (0.96, 3.96)	1.08 (0.82, 1.42)	1.10 (0.80, 1.52)
Rift Valley	3.58 (2.04, 6.29)	1.59 (0.85, 2.95)	0.50 (0.39, 0.66)	1.15 (0.85, 1.55)
Western	2.31 (1.27, 4.20)	1.94 (0.96, 3.92)	0.39 (0.26, 0.57)	0.85 (0.55, 1.32)
Nyanza	1.67 (0.93, 3.01)	1.37 (0.71, 2.65)	0.42 (0.32, 0.56)	0.93 (0.65, 1.33)
**Level of Education**				
No Education (Reference)	1.00	1.00	1.00	1.00
Primary	0.34 (0.28, 0.42)	0.82 (0.63, 1.07)	1.60 (1.24, 2.05)	1.18 (0.85, 1.63)
Secondary	0.25 (0.20, 0.31)	0.64 (0.46, 0.89)	1.93 (1.50, 2.49)	1.29 (0.91, 1.81)
Higher	0.24 (0.16, 0.36)	0.86 (0.54, 1.38)	2.96 (2.20, 3.99)	1.27 (0.86, 1.87)
**Wealth Quintile**				
Poorest (Reference)	1.00	1.00	1.00	1.00
Poorer	0.52 (0.43, 0.63)	0.86 (0.69, 1.08)	1.70 (1.39, 2.07)	1.50 (1.21, 1.87)
Middle	0.37 (0.30, 0.46)	0.63 (0.50, 0.80)	2.29 (1.89, 2.78)	1.83 (1.47, 2.28)
Richer	0.30 (0.23, 0.39)	0.58 (0.43, 0.78)	4.12 (3.39, 5.00)	2.82 (2.24, 3.56)
Richest	0.28 (0.21, 0.37)	0.55 (0.39, 0.77)	5.92 (4.88, 7.19)	3.75 (2.88, 4.89)
**Occupation**				
Not Working (Reference)	1.00	1.00	1.00	1.00
Nonmanual	0.41 (0.33, 0.50)	0.67 (0.53, 0.84)	2.62 (2.27, 3.01)	1.25 (1.04, 1.50)
Manual	0.38 (0.27, 0.52)	0.54 (0.39, 0.75)	1.71 (1.37, 2.14)	1.05 (0.81, 1.37)
Agricultural	0.55 (0.45, 0.66)	0.78 (0.63, 0.98)	1.63 (1.40, 1.89)	1.00 (0.82, 1.22)
**Media Exposure**				
Not Exposed (Reference)	1.00	1.00	1.00	1.00
Exposed to one media source	0.47 (0.39, 0.57)	0.73 (0.59, 0.90)	1.70 (1.40, 2.06)	1.26 (1.02, 1.55)
Exposed to two media sources	0.34 (0.28, 0.42)	0.61 (0.48, 0.78)	2.31 (1.91, 2.79)	1.21 (0.97, 1.50)
Exposed to three media sources	0.32 (0.25, 0.41)	0.58 (0.42, 0.80)	3.12 (2.53, 3.84)	1.56 (1.22, 1.99)
**Hormonal Contraceptive Use**				
No (Reference)	1.00	1.00	1.00	1.00
Yes	0.44 (0.36, 0.54)	0.70 (0.56, 0.87)	1.55 (1.39, 1.73)	1.14 (1.00, 1.31)

Overall Model Fit: F (108, 1393) = 16.96, *P*=0.0000.

Table S59: Unadjusted and adjusted relative risk ratio, of underweight and overweight/obesity among women of child-bearing age in Sao Tome and Principe.

VARIABLES	UNDERWEIGHT		OVERWEIGHT/OBESITY	
	Unadjusted RRR (95% CI)	Adjusted RRR (95% CI)	Unadjusted RRR (95% CI)	Adjusted RRR (95% CI)
**Age**				
15-19 (Reference)	1.00	1.00	1.00	1.00
20-24	0.99 (0.63, 1.55)	1.36 (0.73, 2.55)	1.98 (1.30, 3.04)	1.35 (0.78, 2.33)
25-29	0.42 (0.19, 0.92)	0.52 (0.15, 1.78)	3.03 (1.88, 4.89)	1.64 (0.92, 2.94)
30-34	0.36 (0.17, 0.76)	0.45 (0.12, 1.69)	4.31 (2.69, 6.90)	2.14 (1.12, 4.11)
35-39	0.53 (0.22, 1.29)	0.63 (0.15, 2.65)	6.37 (3.96, 10.24)	3.17 (1.62, 6.20)
40-44	0.27 (0.11, 0.65)	0.32 (0.08, 1.24)	5.29 (3.15, 8.89)	2.65 (1.27, 5.52)
45-49	0.63 (0.24, 1.70)	0.60 (0.23, 1.59)	7.17 (4.79, 10.75)	3.70 (1.86, 7.34)
**Religion**				
Catholics (Reference)	1.00	1.00	1.00	1.00
Other Christians	0.43 (0.20, 0.95)	0.35 (0.15, 0.80)	0.86 (0.62, 1.20)	1.12 (0.76, 1.64)
Others	0.17 (0.03, 0.88)	0.14 (0.02, 0.77)	1.08 (0.62, 1.88)	1.19 (0.68, 2.09)
None	0.69 (0.37, 1.29)	0.57 (0.30, 1.08)	0.61 (0.37, 0.99)	0.76 (0.45, 1.29)
**Parity**				
0 (Reference)	1.00	1.00	1.00	1.00
1	0.58 (0.30, 1.14)	0.92 (0.40, 2.09)	2.73 (1.59, 4.71)	0.84 (0.36, 1.96)
2	0.42 (0.21, 0.81)	0.79 (0.23, 2.73)	2.43 (1.52, 3.90)	0.55 (0.21, 1.42)
3	0.42 (0.20, 0.90)	1.15 (0.32, 4.12)	3.36 (2.09, 5.41)	0.66 (0.26, 1.64)
4	0.47(0.19, 1.17)	1.30 (0.27, 6.29)	4.74 (2.92, 7.68)	0.86 (0.32, 2.33)
5	0.36 (0.14, 0.91)	1.11 (0.27, 4.53)	5.33 (3.34, 8.50)	0.83 (0.31, 2.24)
6+	0.31 (0.12, 0.75)	0.91 (0.24, 3.50)	4.16 (2.64, 6.54)	0.63 (0.23, 1.72)
**Marital Status**				
Single (Reference)	1.00	1.00	1.00	1.00
Married	0.40 (0.25, 0.62)	0.59 (0.24, 1.48)	4.46 (3.13, 6.36)	3.57 (1.78, 7.18)
Formerly Married	0.55 (0.29, 1.04)	0.71 (0.25, 2.04)	3.04 (1.98, 4.66)	2.26 (1.21, 4.21)
**Residential Setting**				
Rural (Reference)	1.00	1.00	1.00	1.00
Urban	1.77 (1.02, 3.06)	1.33 (0.82, 2.14)	1.36 (1.09, 1.70)	1.36 (1.03, 1.79)
**Region**				
Região Centro (Reference)	1.00	1.00	1.00	1.00
Região Sul	0.73 (0.42, 1.26)	0.89 (0.51, 1.57)	0.89 (0.68, 1.16)	1.22 (0.84, 1.76)
Região Norte	0.54 (0.27, 1.09)	0.65 (0.33, 1.28)	0.79 (0.60, 1.03)	1.04 (0.75, 1.45)
Região do Príncipe	0.93 (0.43, 2.02)	1.22 (0.51, 2.89)	1.01 (0.77, 1.33)	1.33 (0.88, 2.01)
**Level of Education**				
No Education (Reference)	1.00	1.00	1.00	1.00
Primary	1.21 (0.51, 2.87)	1.01 (0.38, 2.67)	1.32 (0.80, 2.17)	1.38 (0.89, 2.13)
Secondary	1.42 (0.55, 3.68)	0.49 (0.16, 1.58)	0.93 (0.57, 1.51)	1.15 (0.70, 1.90)
**Wealth Quintile**				
Poorest (Reference)	1.00	1.00	1.00	1.00
Poorer	0.72 (0.33, 1.57)	0.66 (0.29, 1.50)	1.07 (0.71, 1.59)	1.03 (0.69, 1.55)
Middle	0.79 (0.43, 1.46)	0.66 (0.33, 1.32)	1.21 (0.79, 1.85)	1.19 (0.76, 1.87)
Richer	0.54 (0.28, 1.03)	0.42 (0.20, 0.89)	1.77 (1.20, 2.61)	1.89 (1.25, 2.84)
Richest	1.65 (0.89, 3.06)	1.21 (0.56, 2.62)	1.81 (1.28, 2.56)	2.03 (1.39, 2.98)
**Occupation**				
Not Working (Reference)	1.00	1.00	1.00	1.00
Nonmanual	0.96 (0.70, 1.31)	1.22 (0.80, 1.84)	2.26 (1.80, 2.83)	1.46 (1.13, 1.89)
Manual	0.54 (0.21, 1.40)	0.71 (0.25, 1.99)	2.06 (1.29, 3.28)	1.20 (0.73, 2.00)
**Media Exposure**				
Not Exposed (Reference)	1.00	1.00	1.00	1.00
Exposed to one media source	0.39 (0.09, 1.71)	0.38 (0.08, 1.89)	1.31 (0.60, 2.87)	1.51 (0.67, 3.44)
Exposed to two media sources	0.97 (0.28, 3.33)	0.92 (0.25, 3.52)	1.33 (0.74, 2.39)	1.40 (0.75, 2.63)
Exposed to three media sources	1.34 (0.40, 4.55)	1.10 (0.28, 4.31)	1.32 (0.77, 2.28)	1.36 (0.73, 2.54)

	UNDERWEIGHT		OVERWEIGHT/OBESITY	
**Hormonal Contraceptive Use**				
No (Reference)	1.00	1.00	1.00	1.00
Yes	0.42 (0.21, 0.86)	0.63 (0.31, 1.28)	1.10 (0.82, 1.48)	0.87 (0.64, 1.18)

Overall Model Fit: F (66, 21) = 8.15, P=0.0000.

Table S60: Unadjusted and adjusted relative risk ratio, of underweight and overweight/obesity among women of child-bearing age in Togo.

VARIABLES	UNDERWEIGHT		OVERWEIGHT/OBESITY	
	Unadjusted RRR (95% CI)	Adjusted RRR (95% CI)	Unadjusted RRR (95% CI)	Adjusted RRR (95% CI)

	UNDERWEIGHT		OVERWEIGHT/OBESITY	
**Age**				
15-19 (Reference)	1.00	1.00	1.00	1.00
20-24	0.58 (0.40, 0.85)	0.94 (0.60, 1.48)	1.83 (1.33, 2.51)	1.29 (0.93, 1.81)
25-29	0.55 (0.35, 0.86)	1.13 (0.59, 2.16)	3.35 (2.48, 4.52)	2.17 (1.46, 3.24)
30-34	0.62 (0.39, 0.97)	1.41 (0.67, 2.97)	4.96 (3.63, 6.78)	3.56 (2.32, 5.47)
35-39	0.76 (0.47, 1.22)	1.94 (0.91, 4.12)	6.31 (4.70, 8.46)	5.20 (3.38, 8.00)
40-44	0.77 (0.49, 1.23)	1.98 (0.85, 4.62)	7.36 (5.34, 10.2)	6.71 (4.30, 10.5)
45-49	0.82 (0.50, 1.34)	2.12 (0.93, 4.82)	6.35 (4.55, 8.84)	6.87 (4.25, 11.1)
**Ethnicity**				
Adja-Ewé/Mina (Reference)	1.00	1.00	1.00	1.00
Kabye/Tem	0.68 (0.49, 0.95)	0.55 (0.34, 0.90)	0.70 (0.56, 0.86)	0.93 (0.71, 1.22)
Akposso/Akebou	0.36 (0.12, 1.05)	0.39 (0.12, 1.27)	0.89 (0.60, 1.33)	1.34 (0.89, 2.01)
Ana-Ife	0.82 (0.23, 2.87)	0.92 (0.25, 3.38)	0.77 (0.52, 1.15)	1.21 (0.77, 1.92)
Para-Gourma/Akan	1.35 (1.01, 1.79)	0.67 (0.39, 1.17)	0.33 (0.25, 0.45)	0.70 (0.44, 1.11)
Other Togolese	1.31 (0.61, 2.83)	1.04 (0.34, 3.16)	0.31 (0.15, 0.65)	0.40 (0.16, 0.95)
Stranger	0.95 (0.50, 1.80)	1.21 (0.59, 2.48)	1.98 (1.45, 2.72)	1.66 (1.17, 2.36)
**Religion**				
Other Christians (Reference)	1.00	1.00	1.00	1.00
Islam	0.92 (0.64, 1.31)	0.89 (0.57, 1.41)	1.12 (0.91, 1.38)	1.61 (1.22, 2.12)
Traditionalists	1.66 (1.23, 2.23)	1.32 (0.91, 1.91)	0.35 (0.27, 0.47)	0.68 (0.48, 0.96)
No Religion	1.05 (0.69, 1.60)	0.87 (0.56, 1.35)	0.61 (0.45, 0.81)	0.99 (0.70, 1.39)
**Parity**				
0 (Reference)	1.00	1.00	1.00	1.00
1	0.61 (0.40, 0.95)	0.88 (0.49, 1.57)	1.66 (1.28, 2.17)	0.75 (0.51, 1.10)
2	0.56 (0.35, 0.92)	0.82 (0.41, 1.63)	2.85 (2.15, 3.77)	0.86 (0.57, 1.30)
3	0.66 (0.40, 1.07)	0.82 (0.39, 1.71)	3.00 (2.31, 3.90)	0.86 (0.55, 1.37)
4	0.67 (0.38, 1.16)	0.78 (0.35, 1.76)	2.91 (2.21, 3.85)	0.77 (0.47, 1.26)
5	0.92 (0.54, 1.59)	0.87 (0.38, 2.02)	2.57 (1.86, 3.57)	0.92 (0.55, 1.53)
6+	0.80 (0.56, 1.15)	0.58 (0.26, 1.33)	1.84 (1.42, 2.38)	0.77 (0.48, 1.24)
**Marital Status**				
Single (Reference)	1.00	1.00	1.00	1.00
Married	0.73 (0.56, 0.95)	0.73 (0.41, 1.30)	2.87 (2.35, 3.49)	2.03 (1.35, 3.06)
Formerly Married	0.45 (0.24, 0.86)	0.40 (0.16, 0.99)	3.76 (2.71, 5.20)	2.01 (1.22, 3.32)
**Residential Setting**				
Rural (Reference)	1.00	1.00	1.00	1.00
Urban	0.71 (0.52, 0.97)	1.73 (0.87, 3.42)	2.80 (2.37, 3.31)	0.94 (0.64, 1.39)
**Region**				
Grande Agglomération de Lomé (Reference)	1.00	1.00	1.00	1.00
Maritime (Sans Agglomération de Lomé)	1.82 (1.16, 2.86)	1.47 (0.71, 3.05)	0.56 (0.43, 0.74)	0.99 (0.70, 1.39)
Plateaux	0.87 (0.53, 1.44)	0.82 (0.40, 1.68)	0.42 (0.33, 0.52)	0.72 (0.52, 1.01)
Centrale	0.97 (0.58, 1.60)	1.31 (0.61, 2.84)	0.45 (0.36, 0.57)	0.70 (0.49, 0.98)
Kara	1.65 (1.04, 2.63)	1.82 (0.88, 3.78)	0.28 (0.21, 0.37)	0.59 (0.40, 0.87)
Savenes	2.23 (1.46, 3.39)	2.15 (1.00, 4.66)	0.14 (0.09, 0.20)	0.46 (0.25, 0.81)
**Level of Education**				
No Education (Reference)	1.00	1.00	1.00	1.00
Primary	0.92 (0.67, 1.25)	1.27 (0.89, 1.83)	1.59 (1.31, 1.93)	1.38 (1.11, 1.71)
Secondary	0.97 (0.70, 1.34)	1.28 (0.82, 2.01)	1.28 (1.03, 1.60)	1.46 (1.06, 2.01)
Higher	0.75 (0.33, 1.70)	1.18 (0.45, 3.13)	1.83 (1.20, 2.78)	1.70 (0.99, 2.93)

	UNDERWEIGHT		OVERWEIGHT/OBESITY	
**Wealth Quintile**				
Poorest (Reference)	1.00	1.00	1.00	1.00
Poorer	0.69 (0.49, 0.97)	0.81 (0.56, 1.17)	1.80 (1.26, 2.57)	1.16 (0.80, 1.69)
Middle	0.69 (0.50, 0.97)	0.70 (0.46, 1.06)	2.59 (1.91, 3.52)	1.41 (0.98, 2.04)
Richer	0.45 (0.28, 0.72)	0.32 (0.14, 0.70)	4.50 (3.34, 6.07)	2.11 (1.29, 3.46)
Richest	0.53 (0.36, 0.79)	0.35 (0.15, 0.84)	6.75 (5.09, 8.94)	3.12 (1.82, 5.35)
**Occupation**				
Not Working (Reference)	1.00	1.00	1.00	1.00
Nonmanual	0.52 (0.37, 0.74)	0.57 (0.39, 0.84)	3.54 (2.84, 4.41)	1.59 (1.24, 2.03)
Manual	0.82 (0.53, 1.28)	0.98 (0.60, 1.60)	1.93 (1.47, 2.54)	1.16 (0.84, 1.59)
Agricultural	0.93 (0.67, 1.29)	0.81 (0.53, 1.26)	0.88 (0.65, 1.18)	0.94 (0.67, 1.31)
**Media Exposure**				
Not Exposed (Reference)	1.00	1.00	1.00	1.00
Exposed to one media source	0.79 (0.59, 1.05)	0.89 (0.65, 1.22)	1.36 (1.10, 1.68)	1.13 (0.91, 1.41)
Exposed to two media sources	0.65 (0.47, 0.89)	0.90 (0.62, 1.32)	2.53 (2.04, 3.13)	1.36 (1.04, 1.77)
Exposed to three media sources	0.68 (0.41, 1.11)	0.89 (0.48, 1.65)	2.50 (1.87, 3.33)	1.39 (0.95, 2.04)
**Hormonal Contraceptive Use**				
No (Reference)	1.00	1.00	1.00	1.00
Yes	0.37 (0.21, 0.66)	0.44 (0.24, 0.80)	1.08 (0.87, 1.34)	0.98 (0.76, 1.28)

Overall Model Fit: $F(86, 234) = 9.74$, P=0.0000.

Table S61: Unadjusted and adjusted relative risk ratio, of underweight and overweight/obesity among women of child-bearing age in Cameroon.

VARIABLES	UNDERWEIGHT		OVERWEIGHT/OBESITY	
	Unadjusted RRR (95% CI)	Adjusted RRR (95% CI)	Unadjusted RRR (95% CI)	Adjusted RRR (95% CI)
**Age**				
15-19 (Reference)	1.00	1.00	1.00	1.00
20-24	0.85 (0.64, 1.12)	0.81 (0.56, 1.16)	1.65 (1.35, 2.02)	1.34 (1.03, 1.72)
25-29	0.91 (0.63, 1.32)	0.84 (0.52, 1.37)	2.43 (2.01, 2.95)	1.96 (1.49, 2.59)
30-34	0.60 (0.38, 0.93)	0.63 (0.37, 1.08)	3.53 (2.82, 4.43)	2.92 (2.13, 4.02)
35-39	1.23 (0.85, 1.78)	1.19 (0.70, 2.00)	5.01 (4.01, 6.26)	4.52 (3.19, 6.40)
40-44	0.98 (0.64, 1.51)	1.05 (0.58, 1.90)	5.20 (4.13, 6.55)	5.57 (3.89, 7.98)
45-49	0.91 (0.58, 1.43)	1.01 (0.55, 1.86)	4.52 (3.57, 5.73)	4.92 (3.35, 7.24)
**Ethnicity**				
Bamilike/Bamoun (Reference)	1.00	1.00	1.00	1.00
Arabes-Choa/Peulh/Haoussa/Kanuri	16.1 (9.41, 27.4)	6.48 (2.65, 15.9)	0.23 (0.16, 0.31)	0.31 (0.19, 0.50)
Biu-Mandara	5.49 (3.19, 9.44)	2.42 (0.97, 6.03)	0.15 (0.10, 0.21)	0.44 (0.28, 0.70)
Adamaoua-Oubangui	5.65 (3.23, 9.88)	2.62 (1.10, 6.24)	0.21 (0.15, 0.30)	0.40 (0.27, 0.59)
Bantoïde Sud-Ouest	3.65 (1.20, 11.1)	3.16 (0.85, 11.8)	0.61 (0.33, 1.14)	0.51 (0.29, 0.89)
Grassfields	1.26 (0.65, 2.43)	1.10 (0.43, 2.81)	0.62 (0.51, 0.77)	0.70 (0.49, 0.98)
Côtier/Ngoe/Oroko	2.62 (1.18, 5.79)	2.89 (1.26, 6.60)	0.55 (0.41, 0.73)	0.45 (0.31, 0.64)
Beti/Bassa/Mbam	2.96 (1.69, 5.17)	3.52 (1.92, 6.47)	0.50 (0.41, 0.60)	0.42 (0.33, 0.54)
Kako/Meka/Pygmé	4.24 (2.18, 8.27)	2.46 (0.98, 6.19)	0.31 (0.23, 0.43)	0.26 (0.15, 0.45)
Stranger/Others	8.50 (3.90, 18.6)	5.09 (1.89, 13.7)	0.58 (0.37, 0.90)	0.68 (0.44, 1.05)
**Religion**				
Catholics (Reference)	1.00	1.00	1.00	1.00
Other Christians	1.28 (0.97, 1.69)	1.24 (0.93, 1.66)	1.01 (0.88, 1.16)	1.01 (0.87, 1.18)
Islam	3.64 (2.55, 5.19)	1.31 (0.82, 2.09)	0.66 (0.54, 0.81)	1.10 (0.83, 1.47)
Traditionalists	1.95 (1.12, 3.40)	1.04 (0.52, 2.06)	0.27 (0.14, 0.52)	0.54 (0.30, 0.98)
Others	1.53 (0.53, 4.45)	1.56 (0.51, 4.76)	0.88 (0.50, 1.56)	0.68 (0.36, 1.30)
No Religion	1.66 (0.72, 3.84)	1.30 (0.56, 3.03)	0.81 (0.54, 1.21)	0.94 (0.61, 1.45)
**Parity**				
0 (Reference)	1.00	1.00	1.00	1.00
1	1.02 (0.70, 1.48)	1.24 (0.82, 1.87)	1.78 (1.46, 2.16)	1.30 (1.00, 1.69)
2	1.38 (0.86, 2.21)	1.70 (1.03, 2.81)	2.21 (1.78, 2.74)	1.44 (1.06, 1.96)
3	1.20 (0.82, 1.74)	1.49 (0.89, 2.50)	2.04 (1.63, 2.55)	1.24 (0.91, 1.71)
4	1.07 (0.66, 1.75)	1.37 (0.77, 2.44)	2.87 (2.27, 3.63)	1.45 (1.02, 2.05)
5	1.27 (0.80, 2.02)	1.52 (0.83, 2.78)	2.90 (2.22, 3.78)	1.57 (1.04, 2.35)
6+	1.42 (1.02, 1.98)	1.18 (0.70, 2.00)	2.24 (1.87, 2.68)	1.44 (1.02, 2.04)
**Marital Status**				
Single (Reference)	1.00	1.00	1.00	1.00
Married	1.31 (1.00, 1.72)	0.63 (0.42, 0.96)	2.04 (1.77, 2.35)	1.37 (1.08, 1.75)
Formerly Married	0.62 (0.38, 1.01)	0.33 (0.18, 0.60)	2.16 (1.72, 2.71)	1.01 (0.73, 1.40)
**Residential Setting**				
Rural (Reference)	1.00	1.00	1.00	1.00
Urban	0.66 (0.50, 0.85)	1.28 (0.88, 1.88)	2.32 (2.01, 2.67)	0.89 (0.70, 1.12)

	UNDERWEIGHT		OVERWEIGHT/OBESITY	
**Region**				
Yaoundé (Reference)	1.00	1.00	1.00	1.00
Adamaoua	4.69 (2.47, 8.91)	2.37 (0.88, 6.40)	0.40 (0.29, 0.55)	0.75 (0.49, 1.13)
Centre (sans Yaoundé)	0.86 (0.44, 1.72)	0.77 (0.38, 1.58)	0.45 (0.33, 0.62)	0.65 (0.43, 0.97)
Douala	1.01 (0.46, 2.20)	1.13 (0.52, 2.46)	1.01 (0.79, 1.28)	0.82 (0.62, 1.07)
Est	2.81 (1.50, 5.23)	2.09 (0.94, 4.62)	0.50 (0.37, 0.68)	1.25 (0.81, 1.94)
Extrême-Nord	3.81 (2.09, 6.93)	2.01 (0.76, 5.31)	0.11 (0.07, 0.17)	0.31 (0.17, 0.58)
Littoral (sans Douala)	0.69 (0.26, 1.83)	0.68 (0.25, 1.85)	0.89 (0.65, 1.22)	1.04 (0.73, 1.48)
Nord	2.88 (1.59, 5.21)	1.58 (0.61, 4.07)	0.29 (0.20, 0.41)	0.71 (0.43, 1.19)
Nord-Ouest	0.68 (0.32, 1.44)	1.20 (0.40, 3.65)	0.62 (0.47, 0.81)	0.93 (0.60, 1.45)
Ouest	0.53 (0.22, 1.27)	1.06 (0.42, 2.68)	0.93 (0.73, 1.19)	0.91 (0.66, 1.27)
Sud	1.86 (0.92, 3.78)	1.44 (0.66, 3.15)	0.50 (0.37, 0.67)	0.81 (0.58, 1.12)
Sud-Ouest	0.94 (0.43, 2.07)	1.06 (0.43, 2.60)	0.91 (0.68, 1.21)	1.20 (0.83, 1.74)
**Level of Education**				
No Education (Reference)	1.00	1.00	1.00	1.00
Primary	0.49 (0.38, 0.64)	0.94 (0.67, 1.31)	2.40 (1.89, 3.06)	1.07 (0.78, 1.46)
Secondary	0.28 (0.21, 0.38)	0.82 (0.51, 1.32)	2.89 (2.31, 3.62)	1.21 (0.86, 1.70)
Higher	0.20 (0.10, 0.40)	0.84 (0.35, 2.02)	4.12 (3.04, 5.58)	1.05 (0.67, 1.65)
**Wealth Quintile**				
Poorest (Reference)	1.00	1.00	1.00	1.00
Poorer	0.54 (0.39, 0.76)	0.73 (0.49, 1.10)	2.52 (1.83, 3.47)	1.44 (1.01, 2.05)
Middle	0.40 (0.28, 0.58)	0.50 (0.31, 0.81)	4.64 (3.35, 6.44)	2.21 (1.50, 3.26)
Richer	0.44 (0.30, 0.66)	0.63 (0.35, 1.15)	5.77 (4.22, 7.87)	2.50 (1.61, 3.90)
Richest	0.32 (0.21, 0.48)	0.51 (0.26, 1.00)	8.03 (5.91, 10.9)	3.51 (2.24, 5.50)
**Occupation**				
Not Working (Reference)	1.00	1.00	1.00	1.00
Nonmanual	0.70 (0.51, 0.96)	0.81 (0.58, 1.12)	2.43 (2.08, 2.85)	1.20 (0.99, 1.46)
Manual	0.70 (0.42, 1.17)	0.79 (0.52, 1.19)	1.72 (1.40, 2.12)	1.18 (0.94, 1.48)
Agricultural	0.75 (0.56, 1.00)	0.76 (0.52, 1.10)	0.88 (0.73, 1.06)	0.61 (0.48, 0.78)
**Media Exposure**				
Not Exposed (Reference)	1.00	1.00	1.00	1.00
Exposed to one media source	0.72 (0.56, 0.94)	1.15 (0.87, 1.52)	2.16 (1.73, 2.69)	1.08 (0.84, 1.39)
Exposed to two media sources	0.49 (0.37, 0.66)	0.96 (0.68, 1.34)	2.64 (2.16, 3.22)	1.08 (0.83, 1.41)
Exposed to three media sources	0.33 (0.22, 0.50)	0.79 (0.48, 1.31)	3.21 (2.62, 3.94)	1.24 (0.93, 1.66)
**Hormonal Contraceptive Use**				
No (Reference)	1.00	1.00	1.00	1.00
Yes	0.65 (0.35, 1.21)	0.91 (0.50, 1.69)	1.33 (1.03, 1.72)	0.79 (0.59, 1.06)

Overall Model Fit: F (108, 447) = 11.56, *P*=0.0000.

Table S62: Unadjusted and adjusted relative risk ratio, of underweight and overweight/obesity among women of child-bearing age in Comoros.

VARIABLES	UNDERWEIGHT		OVERWEIGHT/OBESITY	
	Unadjusted RRR (95% CI)	Adjusted RRR (95% CI)	Unadjusted RRR (95% CI)	Adjusted RRR (95% CI)
**Age**				
15-19 (Reference)	1.00	1.00	1.00	1.00
20-24	0.70 (0.46, 1.06)	0.81 (0.54, 1.21)	2.10 (1.56, 2.82)	1.69 (1.25, 2.28)
25-29	0.55 (0.35, 0.87)	0.78 (0.48, 1.28)	4.28 (3.26, 5.62)	2.71 (1.99, 3.69)
30-34	0.39 (0.22, 0.71)	0.66 (0.34, 1.26)	5.81 (4.49, 7.53)	3.47 (2.39, 5.03)
35-39	0.31 (0.16, 0.60)	0.53 (0.24, 1.17)	5.77 (4.43, 7.51)	3.14 (2.24, 4.40)
40-44	0.62 (0.34, 1.16)	1.04 (0.50, 2.19)	5.81 (4.32, 7.81)	2.97 (2.01, 4.39)
45-49	0.73 (0.35, 1.51)	1.22 (0.44, 3.38)	8.23 (5.75, 11.79)	4.31 (2.79, 6.66)
**Parity**				
0 (Reference)	1.00	1.00	1.00	1.00
1	0.78 (0.44, 1.38)	1.19 (0.60, 2.36)	2.65 (2.04, 3.45)	1.45 (1.03, 2.06)
2	0.65 (0.36, 1.16)	1.08 (0.51, 2.31)	2.63 (1.97, 3.51)	1.27 (0.89, 1.85)
3	0.40 (0.20, 0.82)	0.68 (0.32, 1.47)	3.64 (2.76, 4.81)	1.76 (1.25, 2.49)
4	0.30 (0.13, 0.69)	0.53 (0.20, 1.46)	3.86 (2.77, 5.37)	1.74 (1.11, 2.71)
5	0.50 (0.24, 1.05)	0.94 (0.36, 2.43)	2.99 (2.15, 4.15)	1.27 (0.82, 1.96)
6+	0.63 (0.35, 1.11)	1.05 (0.48, 2.31)	3.73 (2.93, 4.75)	1.75 (1.17, 2.64)
**Marital Status**				
Single (Reference)	1.00	1.00	1.00	1.00
Married	0.51 (0.39, 0.67)	0.75 (0.45, 1.25)	3.84 (3.11, 4.76)	1.42 (1.02, 1.96)
Formerly Married	0.93 (0.54, 1.59)	1.25 (0.67, 2.34)	2.59 (1.89, 3.55)	0.97 (0.66, 1.44)
**Residential Setting**				
Rural (Reference)	1.00	1.00	1.00	1.00
Urban	0.94 (0.68, 1.30)	0.87 (0.60, 1.27)	1.55 (1.27, 1.89)	1.28 (1.03, 1.60)
**Region**				
Ngazidja (Reference)	1.00	1.00	1.00	1.00
Mwali	1.34 (0.86, 2.07)	1.31 (0.72, 2.37)	1.33 (1.07, 1.67)	1.85 (1.37, 2.50)
Ndzuwani	1.07 (0.76, 1.51)	1.12 (0.71, 1.74)	1.03 (0.84, 1.27)	1.27 (0.98, 1.64)
**Level of Education**				
No Education (Reference)	1.00	1.00	1.00	1.00
Primary	1.38 (0.87, 2.17)	1.27 (0.76, 2.11)	0.92 (0.71, 1.20)	1.15 (0.87, 1.53)
Secondary	1.86 (1.27, 2.72)	1.43 (0.90, 2.28)	0.59 (0.49, 0.71)	0.96 (0.75, 1.21)
Higher	1.28 (0.67, 2.44)	1.25 (0.61, 2.56)	1.05 (0.81, 1.35)	0.88 (0.65, 1.21)
**Wealth Quintile**				
Poorest (Reference)	1.00	1.00	1.00	1.00
Poorer	1.47 (0.94, 2.28)	1.38 (0.87, 2.19)	1.34 (0.99, 1.81)	1.60 (1.19, 2.16)
Middle	1.11 (0.68, 1.80)	1.08 (0.61, 1.91)	1.67 (1.24, 2.26)	2.00 (1.43, 2.79)
Richer	0.89 (0.55, 1.42)	0.83 (0.47, 1.46)	1.65 (1.23, 2.20)	1.95 (1.40, 2.71)
Richest	1.23 (0.74, 2.04)	1.08 (0.57, 2.07)	2.18 (1.63, 2.91)	2.63 (1.87, 3.70)
**Occupation**				
Not Working (Reference)	1.00	1.00	1.00	1.00
Nonmanual	0.72 (0.48, 1.06)	0.82 (0.52, 1.29)	2.17 (1.77, 2.67)	1.32 (1.07, 1.64)
Manual	0.76 (0.47, 1.23)	0.85 (0.53, 1.37)	0.99 (0.72, 1.36)	0.86 (0.61, 1.22)
Agricultural	0.68 (0.41, 1.14)	0.92 (0.50, 1.68)	1.63 (1.20, 2.23)	1.15 (0.79, 1.68)
**Media Exposure**				
Not Exposed (Reference)	1.00	1.00	1.00	1.00
Exposed to one media source	1.23 (0.79, 1.92)	1.09 (0.69, 1.72)	1.18 (0.91, 1.54)	1.15 (0.87, 1.51)
Exposed to two media sources	1.08 (0.75, 1.56)	0.97 (0.67, 1.41)	1.29 (1.01, 1.66)	1.28 (0.96, 1.72)
Exposed to three media sources	1.55 (1.00, 2.41)	1.24 (0.77, 2.00)	1.19 (0.92, 1.53)	1.61 (1.13, 2.28)
**Hormonal Contraceptive Use**				
No (Reference)	1.00	1.00	1.00	1.00
Yes	0.47 (0.22, 0.98)	0.68 (0.32, 1.47)	2.04 (1.56, 2.65)	1.15 (0.87, 1.52)

Overall Model Fit: F (62, 185) = 12.61, *P*=0.0000.

Table S63: Unadjusted and adjusted relative risk ratio, of underweight and overweight/obesity among women of child-bearing age in Tanzania.

VARIABLES	UNDERWEIGHT		OVERWEIGHT/OBESITY	
	Unadjusted RRR (95% CI)	Adjusted RRR (95% CI)	Unadjusted RRR (95% CI)	Adjusted RRR (95% CI)
**Age**				
15-19 (Reference)	1.00	1.00	1.00	1.00
20-24	0.44 (0.35, 0.56)	0.67 (0.52, 0.87)	1.85 (1.47, 2.34)	1.70 (1.27, 2.27)
25-29	0.35 (0.27, 0.46)	0.65 (0.46, 0.92)	3.22 (2.53, 4.11)	3.12 (2.24, 4.36)
30-34	0.48 (0.37, 0.64)	1.00 (0.69, 1.47)	4.52 (3.61, 5.67)	4.94 (3.58, 6.81)
35-39	0.49 (0.37, 0.65)	1.08 (0.73, 1.59)	4.75 (3.81, 5.93)	5.86 (4.19, 8.21)
40-44	0.59 (0.45, 0.77)	1.29 (0.87, 1.91)	5.63 (4.53, 6.99)	8.44 (6.05, 11.8)
45-49	0.59 (0.44, 0.79)	1.27 (0.82, 1.97)	5.48 (4.46, 6.73)	8.99 (6.51, 12.4)
**Parity**				
0 (Reference)	1.00	1.00	1.00	1.00
1	0.51 (0.39, 0.66)	0.84 (0.62, 1.16)	1.65 (1.37, 1.98)	1.07 (0.84, 1.38)
2	0.39 (0.30, 0.51)	0.70 (0.47, 1.05)	2.42 (2.02, 2.89)	1.06 (0.80, 1.41)
3	0.47 (0.35, 0.62)	0.79 (0.51, 1.23)	2.53 (2.11, 3.04)	0.92 (0.69, 1.24)
4	0.53 (0.40, 0.72)	0.79 (0.53, 1.18)	2.29 (1.82, 2.88)	0.92 (0.66, 1.30)
5	0.51 (0.37, 0.71)	0.66 (0.41, 1.05)	1.96 (1.54, 2.48)	0.80 (0.57, 1.12)
6+	0.52 (0.42, 0.64)	0.56 (0.38, 0.85)	1.81 (1.49, 2.20)	0.78 (0.57, 1.08)
**Marital Status**				
Single (Reference)	1.00	1.00	1.00	1.00
Married	0.49 (0.42, 0.57)	0.62 (0.48, 0.81)	2.01 (1.76, 2.30)	1.32 (1.06, 1.64)
Formerly Married	0.52 (0.40, 0.68)	0.66 (0.47, 0.93)	2.03 (1.72, 2.40)	1.07 (0.84, 1.35)
**Residential Setting**				
Rural (Reference)	1.00	1.00	1.00	1.00
Urban	0.93 (0.78, 1.10)	1.07 (0.85, 1.35)	2.66 (2.33, 3.05)	1.05 (0.88, 1.25)

	UNDERWEIGHT		OVERWEIGHT/OBESITY	
**Region**				
Dar es salaam (Reference)	1.00	1.00	1.00	1.00
Dodoma	1.35 (0.89, 2.06)	1.07 (0.66, 1.72)	0.35 (0.24, 0.51)	0.85 (0.58, 1.25)
Arusha	1.52 (1.01, 2.30)	1.28 (0.81, 2.03)	0.56 (0.41, 0.77)	0.94 (0.71, 1.23)
Kilimanjaro	0.92 (0.54, 1.59)	0.78 (0.44, 1.39)	0.82 (0.55, 1.23)	1.20 (0.82, 1.74)
Tanga	1.01 (0.62, 1.64)	0.82 (0.47, 1.43)	0.67 (0.46, 0.99)	1.22 (0.87, 1.71)
Morogoro	0.34 (0.20, 0.59)	0.33 (0.19, 0.58)	0.60 (0.42, 0.86)	1.17 (0.83, 1.64)
Pwani	1.13 (0.75, 1.70)	1.04 (0.67, 1.64)	0.70 (0.47, 1.03)	1.35 (0.87, 2.10)
Lindi	0.68 (0.40, 1.14)	0.62 (0.34, 1.15)	0.42 (0.31, 0.58)	1.06 (0.72, 1.58)
Mtwara	0.93 (0.57, 1.52)	0.86 (0.50, 1.47)	0.46 (0.34, 0.62)	1.12 (0.82, 1.53)
Ruvuma	0.63 (0.36, 1.12)	0.59 (0.33, 1.08)	0.29 (0.19, 0.47)	0.63 (0.42, 0.96)
Iringa	0.79 (0.52, 1.20)	0.68 (0.43, 1.06)	0.43 (0.29, 0.64)	0.72 (0.49, 1.07)
Mbeya	0.40 (0.22, 0.71)	0.31 (0.17, 0.57)	0.54 (0.36, 0.80)	1.19 (0.80, 1.75)
Singida	1.63 (1.08, 2.46)	1.42 (0.91, 2.23)	0.35 (0.25, 0.51)	0.97 (0.66, 1.41)
Tabora	1.02 (0.65, 1.60)	0.83 (0.49, 1.41)	0.38 (0.29, 0.52)	1.34 (0.97, 1.85)
Rukwa	0.46 (0.26, 0.80)	0.39 (0.22, 0.71)	0.29 (0.20, 0.42)	0.78 (0.52, 1.16)
Kigoma	0.96 (0.63, 1.46)	0.81 (0.50, 1.32)	0.25 (0.19, 0.35)	0.78 (0.54, 1.14)
Shinyanga	0.77 (0.50, 1.18)	0.64 (0.40, 1.04)	0.35 (0.25, 0.48)	0.90 (0.67, 1.21)
Kagera	1.16 (0.73, 1.86)	1.02 (0.61, 1.71)	0.19 (0.13, 0.28)	0.53 (0.35, 0.79)
Mwanza	0.82 (0.51, 1.33)	0.65 (0.40, 1.03)	0.31 (0.21, 0.45)	0.68 (0.48, 0.98)
Mara	0.89 (0.58, 1.36)	0.79 (0.50, 1.26)	0.21 (0.13, 0.32)	0.46 (0.31, 0.68)
Manyara	1.53 (0.99, 2.36)	1.14 (0.71, 1.82)	0.30 (0.22, 0.42)	0.91 (0.62, 1.32)
Njombe	0.47 (0.25, 0.86)	0.44 (0.23, 0.85)	0.44 (0.34, 0.58)	0.81 (0.59, 1.12)
Katavi	0.70 (0.41, 1.22)	0.61 (0.34, 1.10)	0.28 (0.20, 0.40)	0.86 (0.62, 1.20)
Simiyu	1.24 (0.81, 1.91)	0.98 (0.60, 1.60)	0.19 (0.13, 0.28)	0.67 (0.47, 0.96)
geita	1.17 (0.75, 1.82)	1.01 (0.61, 1.66)	0.28 (0.19, 0.41)	0.85 (0.61, 1.19)
Kaskazini Unguja	1.58 (0.98, 2.52)	1.29 (0.75, 2.22)	0.71 (0.55, 0.92)	1.38 (0.94, 2.03)
	1.51 (0.97, 2.34)	1.37 (0.84, 2.23)	0.81 (0.57, 1.15)	1.32 (0.88, 1.97)
Kusini Unguja	1.78 (1.29, 2.48)	1.63 (1.16, 2.29)	0.97 (0.76, 1.24)	1.05 (0.78, 1.42)
Mjini Magharibi	1.34 (0.89, 2.02)	0.96 (0.61, 1.51)	0.46 (0.30, 0.71)	0.95 (0.60, 1.49)
Kaskazini Pemba	0.89 (0.57, 1.40)	0.61 (0.37, 1.01)	0.59 (0.40, 0.87)	1.36 (0.94, 1.96)
Kusini Pemba				
**Level of Education**				
No Education (Reference)	1.00	1.00	1.00	1.00
Primary	1.02 (0.81, 1.28)	1.05 (0.83, 1.33)	1.44 (1.23, 1.69)	1.04 (0.88, 1.24)
Secondary	1.22 (0.95, 1.57)	0.99 (0.72, 1.36)	1.79 (1.48, 2.15)	1.14 (0.91, 1.44)
Higher	0.59 (0.17, 2.02)	0.73 (0.20, 2.67)	6.09 (3.96, 9.36)	1.55 (1.04, 2.31)
**Wealth Quintile**				
Poorest (Reference)	1.00	1.00	1.00	1.00
Poorer	0.98 (0.79, 1.23)	1.06 (0.86, 1.32)	1.38 (1.11, 1.72)	1.33 (1.06, 1.67)
Middle	0.72 (0.57, 0.91)	0.75 (0.60, 0.96)	1.79 (1.45, 2.23)	1.71 (1.36, 2.14)
Richer	0.82 (0.65, 1.04)	0.74 (0.57, 0.96)	3.52 (2.88, 4.32)	2.92 (2.32, 3.68)
Richest	0.79 (0.63, 1.00)	0.57 (0.41, 0.81)	6.19 (5.07, 7.56)	4.57 (3.42, 6.12)
**Occupation**				
Not Working (Reference)	1.00	1.00	1.00	1.00
Nonmanual	0.49 (0.38, 0.63)	0.67 (0.50, 0.89)	2.80 (2.40, 3.27)	1.30 (1.08, 1.57)
Manual	0.54 (0.42, 0.70)	0.81 (0.61, 1.06)	2.19 (1.86, 2.58)	1.09 (0.91, 1.30)
Agricultural	0.58 (0.49, 0.68)	0.74 (0.60, 0.92)	0.78 (0.67, 0.92)	0.79 (0.66, 0.95)
**Media Exposure**				
Not Exposed (Reference)	1.00	1.00	1.00	1.00
Exposed to one media source	0.74 (0.59, 0.93)	0.74 (0.59, 0.93)	1.26 (1.03, 1.54)	0.97 (0.79, 1.18)
Exposed to two media sources	0.79 (0.62, 1.00)	0.74 (0.58, 0.94)	1.94 (1.60, 2.36)	1.18 (0.98, 1.43)
Exposed to three media sources	0.72 (0.58, 0.91)	0.67 (0.52, 0.88)	2.80 (2.29, 3.42)	1.30 (1.05, 1.62)

	UNDERWEIGHT		OVERWEIGHT/OBESITY	
**Hormonal Contraceptive Use**				
No (Reference)	1.00	1.00	1.00	1.00
Yes	0.54 (0.44, 0.67)	0.83 (0.66, 1.05)	1.23 (1.08, 1.39)	1.10 (0.97, 1.26)

Overall Model Fit: F (116, 434) = 16.49, *P*=0.0000.

Table S64: Unadjusted and adjusted relative risk ratio, of underweight and overweight/obesity among women of child-bearing age in Zimbabwe.

VARIABLES	UNDERWEIGHT		OVERWEIGHT/OBESITY	
	Unadjusted RRR (95% CI)	Adjusted RRR (95% CI)	Unadjusted RRR (95% CI)	Adjusted RRR (95% CI)
**Age**				
15-19 (Reference)	1.00	1.00	1.00	1.00
20-24	0.55 (0.42, 0.72)	0.89 (0.65, 1.23)	1.94 (1.58, 2.38)	1.42 (1.11, 1.82)
25-29	0.28 (0.19, 0.42)	0.64 (0.40, 1.03)	3.06 (2.48, 3.79)	2.08 (1.55, 2.80)
30-34	0.48 (0.35, 0.66)	1.21 (0.72, 2.02)	5.19 (4.21, 6.40)	3.28 (2.44, 4.42)
35-39	0.53 (0.36, 0.77)	1.29 (0.72, 2.31)	5.67 (4.52, 7.12)	3.60 (2.62, 4.93)
40-44	0.42 (0.27, 0.65)	0.92 (0.49, 1.73)	7.09 (5.71, 8.81)	4.56 (3.33, 6.26)
45-49	0.64 (0.40, 1.04)	1.31 (0.67, 2.57)	6.95 (5.35, 9.03)	4.77 (3.28, 6.93)
**Religion**				
Catholics (Reference)	1.00	1.00	1.00	1.00
Other Christians	1.04 (0.71, 1.51)	1.09 (0.74, 1.60)	0.83 (0.68, 1.02)	0.95 (0.76, 1.19)
Islam	0.77 (0.09, 6.49)	0.91 (0.14, 6.07)	1.01 (0.35, 2.92)	1.13 (0.35, 3.63)
Traditionalist	0.55 (0.08, 3.83)	0.66 (0.10, 4.53)	1.09 (0.57, 2.08)	1.26 (0.63, 2.54)
No Religion	0.89 (0.46, 1.71)	0.91 (0.47, 1.78)	0.75 (0.56, 0.99)	1.05 (0.77, 1.43)
**Parity**				
0 (Reference)	1.00	1.00	1.00	1.00
1	0.65 (0.49, 0.86)	0.83 (0.57, 1.21)	1.68 (1.42, 1.98)	0.86 (0.65, 1.13)
2	0.43 (0.31, 0.59)	0.58 (0.34, 0.97)	2.58 (2.14, 3.12)	0.96 (0.71, 1.32)
3	0.37 (0.26, 0.52)	0.48 (0.27, 0.85)	3.18 (2.66, 3.79)	1.04 (0.75, 1.45)
4	0.42 (0.29, 0.63)	0.46 (0.24, 0.89)	3.01 (2.44, 3.71)	1.00 (0.70, 1.42)
5	0.43 (0.25, 0.74)	0.45 (0.21, 0.95)	3.00 (2.28, 3.93)	1.11 (0.74, 1.66)
6+	0.61 (0.39, 0.95)	0.55 (0.27, 1.12)	3.43 (2.73, 4.32)	1.37 (0.93, 2.01)
**Marital Status**				
Single (Reference)	1.00	1.00	1.00	1.00
Married	0.42 (0.34, 0.53)	0.69 (0.48, 0.99)	2.88 (2.48, 3.33)	1.80 (1.38, 2.35)
Formerly Married	0.68 (0.52, 0.90)	1.10 (0.72, 1.67)	2.95 (2.48, 3.52)	1.55 (1.14, 2.12)
**Residential Setting**				
Rural (Reference)	1.00	1.00	1.00	1.00
Urban	0.73 (0.58, 0.93)	1.78 (1.04, 3.07)	2.16 (1.88, 2.49)	0.93 (0.72, 1.21)
**Region**				
Harare (Reference)	1.00	1.00	1.00	1.00
Manicaland	1.07 (0.68, 1.70)	0.81 (0.46, 1.41)	0.50 (0.39, 0.65)	0.88 (0.67, 1.17)
Mashonaland Central	1.15 (0.70, 1.88)	0.88 (0.49, 1.58)	0.45 (0.35, 0.58)	0.87 (0.65, 1.16)
Mashonaland East	2.69 (1.68, 4.31)	2.22 (1.29, 3.82)	0.61 (0.48, 0.78)	0.93 (0.71, 1.22)
Mashonaland West	1.98 (1.25, 3.12)	1.64 (0.94, 2.85)	0.54 (0.43, 0.68)	0.83 (0.62, 1.12)
Mashonaland North	2.85 (1.70, 4.76)	1.87 (1.01, 3.45)	0.48 (0.35, 0.65)	1.05 (0.77, 1.43)
Mashonaland South	2.99 (1.86, 4.79)	2.07 (1.17, 3.63)	0.50 (0.39, 0.65)	0.93 (0.68, 1.26)
Midlands	1.84 (1.16, 2.94)	1.37 (0.76, 2.46)	0.54 (0.40, 0.72)	0.93 (0.67, 1.30)
Masvingo	0.94 (0.55, 1.61)	0.66 (0.36, 1.20)	0.51 (0.39, 0.66)	0.91 (0.69, 1.20)
Bulawayo	1.59 (0.99, 2.57)	1.44 (0.87, 2.38)	0.95 (0.76, 1.19)	1.07 (0.82, 1.39)
**Level of Education**				
No Education (Reference)	1.00	1.00	1.00	1.00
Primary	1.03 (0.38, 2.82)	0.95 (0.35, 2.57)	0.77 (0.48, 1.25)	1.01 (0.57, 1.78)
Secondary	1.04 (0.39, 2.79)	0.91 (0.34, 2.42)	1.07 (0.67, 1.72)	1.15 (0.65, 2.04)
Higher	0.65 (0.22, 1.97)	0.99 (0.32, 3.08)	2.48 (1.51, 4.09)	1.29 (0.71, 2.36)

	UNDERWEIGHT		OVERWEIGHT/OBESITY	
**Wealth Quintile**				
Poorest (Reference)	1.00	1.00	1.00	1.00
Poorer	1.07 (0.79, 1.44)	1.04 (0.78, 1.38)	1.39 (1.12, 1.73)	1.44 (1.16, 1.80)
Middle	1.10 (0.82, 1.47)	0.94 (0.68, 1.28)	1.87 (1.54, 2.28)	1.92 (1.55, 2.39)
Richer	0.80 (0.58, 1.11)	0.48 (0.29, 0.78)	2.83 (2.30, 3.48)	2.89 (2.21, 3.78)
Richest	0.56 (0.40, 0.78)	0.26 (0.14, 0.48)	3.94 (3.26, 4.76)	4.16 (3.02, 5.75)
**Occupation**				
Not Working (Reference)	1.00	1.00	1.00	1.00
Nonmanual	0.58 (0.46, 0.74)	0.75 (0.57, 1.00)	2.16 (1.92, 2.44)	1.17 (1.02, 1.36)
Manual	0.58 (0.30, 1.13)	0.79 (0.39, 1.59)	1.85 (1.37, 2.50)	0.89 (0.64, 1.24)
Agricultural	0.59 (0.38, 0.91)	0.76 (0.48, 1.21)	1.24 (1.01, 1.52)	0.91 (0.73, 1.13)
Others	1.28 (0.56, 2.97)	1.62 (0.70, 3.72)	2.24 (1.32, 3.80)	1.07 (0.62, 1.84)
**Media Exposure**				
Not Exposed (Reference)	1.00	1.00	1.00	1.00
Exposed to one media source	0.89 (0.69, 1.17)	1.01 (0.77, 1.32)	1.18 (1.01, 1.37)	0.97 (0.83, 1.14)
Exposed to two media sources	0.85 (0.63, 1.15)	1.03 (0.75, 1.41)	1.70 (1.44, 2.00)	1.09 (0.91, 1.30)
Exposed to three media sources	0.61 (0.45, 0.84)	0.87 (0.60, 1.26)	2.45 (2.08, 2.89)	1.29 (1.06, 1.58)
**Hormonal Contraceptive Use**				
No (Reference)	1.00	1.00	1.00	1.00
Yes	0.53 (0.42, 0.65)	0.87 (0.64, 1.18)	1.49 (1.34, 1.67)	1.04 (0.90, 1.19)

Overall Model Fit: F (86, 296) = 6.49, *P*=0.0000.

Table S65: Unadjusted and adjusted relative risk ratio, of underweight and overweight/obesity among women of child-bearing age in Gabon.

VARIABLES	UNDERWEIGHT		OVERWEIGHT/OBESITY	
	Unadjusted RRR (95% CI)	Adjusted RRR (95% CI)	Unadjusted RRR (95% CI)	Adjusted RRR (95% CI)
**Age**				
15-19 (Reference)	1.00	1.00	1.00	1.00
20-24	0.61 (0.37, 0.99)	0.77 (0.40, 1.46)	1.88 (1.26, 2.80)	1.83 (1.16, 2.89)
25-29	0.40 (0.22, 0.73)	0.55 (0.22, 1.40)	3.43 (2.29, 5.14)	2.93 (1.77, 4.84)
30-34	0.30 (0.14, 0.64)	0.41 (0.13, 1.23)	6.49 (4.20, 10.0)	5.42 (3.18, 9.23)
35-39	0.38 (0.16, 0.91)	0.58 (0.15, 2.21)	7.25 (4.98, 10.5)	6.42 (3.96, 10.4)
40-44	0.20 (0.09, 0.43)	0.32 (0.09, 1.22)	7.99 (5.04, 12.7)	7.02 (4.12, 12.0)
45-49	0.24 (0.11, 0.51)	0.48 (0.13, 1.69)	9.04 (5.81, 14.0)	9.10 (5.03, 16.5)
**Ethnicity**				
Shira-Punu/Vili (Reference)	1.00	1.00	1.00	1.00
Fang	0.48 (0.28, 0.83)	0.46 (0.25, 0.84)	1.31 (0.90, 1.91)	1.37 (0.87, 2.17)
Kota-Kele	0.57 (0.31, 1.03)	0.51 (0.21, 1.23)	0.78 (0.51, 1.19)	0.98 (0.58, 1.64)
Mbede-Teke	0.67 (0.36, 1.26)	0.61 (0.26, 1.47)	1.12 (0.73, 1.71)	1.04 (0.62, 1.76)
Myene	1.20 (0.59, 2.44)	0.89 (0.39, 2.05)	1.68 (1.04, 2.73)	1.80 (1.05, 3.06)
Nzabi-Duma	1.06 (0.67, 1.68)	1.02 (0.66, 1.59)	1.07 (0.78, 1.47)	1.10 (0.77, 1.57)
Okande-Tsogho	0.64 (0.36, 1.13)	0.65 (0.36, 1.17)	0.74 (0.45, 1.20)	1.29 (0.70, 2.36)
Pygmee	0.22 (0.04, 1.33)	0.28 (0.04, 1.86)	0.08 (0.03, 0.23)	0.10 (0.02, 0.52)
Other	0.43 (0.24, 0.80)	0.44 (0.23, 0.83)	0.88 (0.60, 1.30)	0.93 (0.57, 1.53)
**Religion**				
Catholics (Reference)	1.00	1.00	1.00	1.00
Other Christians	1.72 (1.18, 2.53)	1.53 (0.99, 2.35)	1.02 (0.82, 1.27)	1.19 (0.91, 1.54)
Others	1.08 (0.36, 3.27)	0.83 (0.25, 2.74)	1.05 (0.52, 2.14)	1.18 (0.54, 2.60)
No Religion	1.45 (0.74, 2.85)	1.39 (0.65, 2.99)	0.82 (0.56, 1.19)	1.39 (0.82, 2.34)
**Parity**				
0 (Reference)	1.00	1.00	1.00	1.00
1	0.47 (0.30, 0.72)	0.75 (0.48, 1.15)	1.57 (1.05, 2.35)	0.89 (0.55, 1.44)
2	0.40 (0.19, 0.84)	0.83 (0.34, 2.06)	3.54 (2.39, 5.24)	1.42 (0.84, 2.43)
3	0.68 (0.31, 1.47)	1.80 (0.73, 4.45)	3.00 (1.88, 4.78)	0.89 (0.50, 1.59)
4	0.25 (0.11, 0.57)	0.81 (0.27, 2.43)	3.55 (2.40, 5.25)	0.98 (0.57, 1.66)
5	0.55 (0.25, 1.22)	1.71 (0.49, 6.00)	4.31 (2.75, 6.77)	1.12 (0.62, 2.03)
6+	0.21 (0.12, 0.37)	0.82 (0.29, 2.37)	4.21 (2.80, 6.33)	1.13 (0.66, 1.92)
**Marital Status**				
Single (Reference)	1.00	1.00	1.00	1.00
Married	0.38 (0.24, 0.59)	0.61 (0.35, 1.05)	2.72 (2.15, 3.44)	1.29 (0.96, 1.73)
Formerly Married	0.44 (0.24, 0.79)	0.89 (0.46, 1.71)	4.13 (2.80, 6.09)	1.70 (1.07, 2.72)
**Residential Setting**				
Rural (Reference)	1.00	1.00	1.00	1.00
Urban	1.13 (0.84, 1.52)	0.70 (0.48, 1.03)	1.49 (1.16, 1.89)	1.10 (0.80, 1.51)
**Region**				
Estuaire (Reference)	1.00	1.00	1.00	1.00
Libreville-Port-Gentil	1.42 (0.92, 2.20)	1.69 (1.01, 2.82)	1.23 (0.86, 1.76)	1.04 (0.69, 1.59)
Haut-Ogooué	1.28 (0.80, 2.07)	1.51 (0.73, 3.14)	0.89 (0.60, 1.31)	1.04 (0.62, 1.75)
Moyen-Ogooué	2.02 (1.23, 3.32)	2.40 (1.26, 4.55)	0.97 (0.66, 1.44)	1.08 (0.71, 1.65)
Ngounié	1.19 (0.75, 1.87)	1.27 (0.76, 2.12)	0.46 (0.31, 0.70)	0.58 (0.35, 0.95)
Nyanga	0.93 (0.56, 1.53)	0.93 (0.53, 1.64)	0.66 (0.43, 1.01)	0.86 (0.50, 1.49)
Ogooué Maritime	1.13 (0.62, 2.06)	1.09 (0.55, 2.14)	0.98 (0.64, 1.51)	0.88 (0.54, 1.44)
Ogooué-Ivindo	1.08 (0.71, 1.66)	1.96 (1.01, 3.82)	0.56 (0.38, 0.83)	0.92 (0.58, 1.46)
Ogooué-Lolo	1.09 (0.64, 1.86)	1.37 (0.73, 2.56)	0.73 (0.47, 1.13)	0.99 (0.60, 1.63)
Woleu-N’tem	0.56 (0.22, 1.44)	1.05 (0.39, 2.84)	0.88 (0.62, 1.26)	0.92 (0.58, 1.44)

	UNDERWEIGHT		OVERWEIGHT/OBESITY	
**Level of Education**				
No Education (Reference)	1.00	1.00	1.00	1.00
Primary	4.23 (1.01, 17.8)	3.57 (0.77, 16.6)	2.95 (1.46, 5.95)	2.89 (1.47, 5.68)
Secondary	8.31 (2.01, 34.4)	5.22 (1.15, 23.7)	2.96 (1.55, 5.63)	3.39 (1.74, 6.63)
Higher	4.06 (0.83, 20.0)	3.04 (0.56, 16.5)	3.85 (1.75, 8.47)	2.48 (1.10, 5.61)
**Wealth Quintile**				
Poorest (Reference)	1.00	1.00	1.00	1.00
Poorer	0.95 (0.61, 1.48)	0.73 (0.45, 1.19)	1.33 (1.01, 1.77)	1.35 (0.91, 1.99)
Middle	1.26 (0.80, 2.00)	1.00 (0.58, 1.75)	1.85 (1.43, 2.39)	1.85 (1.19, 2.87)
Richer	0.98 (0.61, 1.58)	0.66 (0.37, 1.17)	1.93 (1.41, 2.64)	1.96 (1.21, 3.17)
Richest	1.65 (1.01, 2.69)	1.22 (0.70, 2.12)	2.45 (1.75, 3.41)	2.57 (1.59, 4.15)
**Occupation**				
Not Working (Reference)	1.00	1.00	1.00	1.00
Nonmanual	0.45 (0.27, 0.75)	0.67 (0.39, 1.17)	2.16 (1.71, 2.73)	1.01 (0.78, 1.32)
Manual	0.56 (0.16, 1.87)	0.71 (0.20, 2.58)	3.40 (1.97, 5.85)	1.82 (1.04, 3.18)
Agricultural	0.55 (0.34, 0.88)	1.01 (0.61, 1.69)	1.13 (0.73, 1.74)	0.93 (0.58, 1.48)
**Media Exposure**				
Not Exposed (Reference)	1.00	1.00	1.00	1.00
Exposed to one media source	1.62 (0.96, 2.73)	1.51 (0.83, 2.73)	1.35 (0.83, 2.21)	0.98 (0.55, 1.75)
Exposed to two media sources	1.33 (0.79, 2.23)	1.24 (0.67, 2.28)	1.58 (0.97, 2.57)	0.91 (0.52, 1.61)
Exposed to three media sources	1.67 (1.01, 2.77)	1.49 (0.75, 2.95)	1.60 (1.02, 2.51)	0.77 (0.42, 1.40)
**Hormonal Contraceptive Use**				
No (Reference)	1.00	1.00	1.00	1.00
Yes	1.28 (0.46, 3.60)	1.77 (0.67, 4.72)	1.85 (0.97, 3.56)	1.45 (0.70, 2.99)

Overall Model Fit: $F(98, 218) = 13.70$, P=0.0000.

Table S66: Unadjusted and adjusted relative risk ratio, of underweight and overweight/obesity among women of child-bearing age in Ghana.

VARIABLES	UNDERWEIGHT		OVERWEIGHT/OBESITY	
	Unadjusted RRR (95% CI)	Adjusted RRR (95% CI)	Unadjusted RRR (95% CI)	Adjusted RRR (95% CI)
**Age**				
15-19 (Reference)	1.00	1.00	1.00	1.00
20-24	0.47 (0.29, 0.76)	0.67 (0.41, 1.10)	3.63 (2.55, 5.18)	3.04 (2.01, 4.60)
25-29	0.56 (0.34, 0.91)	1.03 (0.57, 1.84)	7.91 (5.62, 11.1)	5.51 (3.53, 8.61)
30-34	0.58 (0.35, 0.96)	1.04 (0.49, 2.20)	11.1 (7.71, 16.1)	8.56 (5.19, 14.1)
35-39	0.57 (0.34, 0.97)	1.06 (0.44, 2.52)	11.1 (7.76, 15.8)	9.00 (5.33, 15.2)
40-44	0.31 (0.16, 0.64)	0.54 (0.19, 1.56)	13.6 (9.39, 19.8)	12.0 (7.02, 20.6)
45-49	0.42 (0.24, 0.75)	0.65 (0.26, 1.58)	10.7 (7.27, 15.6)	10.2 (5.92, 17.4)
**Ethnicity**	.			
Akan (Reference)	1.00	1.00	1.00	1.00
Ga/Dangme	0.91 (0.48, 1.73)	0.74 (0.36, 1.52)	1.11 (0.83, 1.50)	1.00 (0.65, 1.53)
Ewe	0.89 (0.53, 1.47)	0.59 (0.30, 1.18)	0.72 (0.53, 0.98)	0.79 (0.56, 1.11)
Guan	0.77 (0.38, 1.56)	0.51 (0.24, 1.09)	0.86 (0.58, 1.25)	1.00 (0.66, 1.50)
Mole-Dagbani	1.12 (0.79, 1.58)	0.68 (0.34, 1.36)	0.36 (0.26, 0.49)	0.71 (0.49, 1.02)
Grusi	0.57 (0.30, 1.07)	0.39 (0.19, 0.80)	0.38 (0.24, 0.62)	0.79 (0.51, 1.22)
Gurma	1.11 (0.74, 1.67)	0.57 (0.27, 1.18)	0.21 (0.13, 0.33)	0.78 (0.45, 1.36)
Mande	0.35 (0.07, 1.77)	0.14 (0.02, 0.89)	0.67 (0.28, 1.60)	1.27 (0.42, 3.85)
Others	3.03 (1.34, 6.83)	2.08 (0.91, 4.77)	1.49 (0.85, 2.61)	1.54 (0.75, 3.16)
**Religion**				
Catholics (Reference)	1.00	1.00	1.00	1.00
Other Christians	1.09 (0.69, 1.74)	1.14 (0.68, 1.90)	1.31 (1.01, 1.70)	0.86 (0.64, 1.15)
Islam	1.44 (0.86, 2.39)	1.51 (0.85, 2.69)	0.97 (0.66, 1.43)	1.38 (0.91, 2.08)
Traditionalist	1.76 (0.78, 3.95)	1.52 (0.67, 3.46)	0.61 (0.32, 1.16)	1.69 (0.88, 3.21)
No Religion	0.33 (0.10, 1.12)	0.37 (0.11, 1.30)	0.65 (0.39, 1.07)	0.78 (0.42, 1.42)
**Parity**				
0 (Reference)	1.00	1.00	1.00	1.00
1	0.42 (0.25, 0.72)	0.60 (0.34, 1.05)	2.09 (1.59, 2.74)	0.94 (0.65, 1.36)
2	0.71 (0.40, 1.27)	1.03 (0.50, 2.11)	3.87 (2.94, 5.10)	1.34 (0.90, 1.99)
3	0.51 (0.29, 0.90)	0.72 (0.36, 1.45)	3.65 (2.80, 4.75)	1.22 (0.80, 1.86)
4	0.66 (0.38, 1.17)	0.92 (0.42, 1.98)	3.25 (2.37, 4.46)	0.98 (0.60, 1.60)
5	0.29 (0.13, 0.67)	0.39 (0.12, 1.20)	2.21 (1.57, 3.11)	0.83 (0.51, 1.36)
6+	0.59 (0.38, 0.94)	0.85 (0.36, 2.02)	2.23 (1.67, 2.96)	1.30 (0.78, 2.16)
**Marital Status**				
Single (Reference)	1.00	1.00	1.00	1.00
Married	0.54 (0.39, 0.75)	0.83 (0.50, 1.37)	3.14 (2.58, 3.81)	1.48 (1.06, 2.06)
Formerly Married	0.75 (0.42, 1.33)	1.26 (0.59, 2.68)	4.55 (3.47, 5.96)	1.74 (1.14, 2.66)
**Residential Setting**				
Rural (Reference)	1.00	1.00	1.00	1.00
Urban	0.96 (0.72, 1.28)	1.12 (0.76, 1.66)	2.30 (1.91, 2.78)	0.71 (0.55, 0.92)
**Region**				
Greater Accra (Reference)	1.00	1.00	1.00	1.00
Western	0.83 (0.45, 1.52)	0.72 (0.35, 1.48)	0.55 (0.42, 0.72)	0.81 (0.59, 1.12)
Central	0.55 (0.27, 1.12)	0.47 (0.21, 1.06)	0.50 (0.38, 0.66)	0.73 (0.51, 1.03)
Volta	1.01 (0.50, 2.06)	1.18 (0.50, 2.78)	0.35 (0.23, 0.51)	0.79 (0.51, 1.23)
Eastern	1.04 (0.56, 1.94)	0.93 (0.44, 1.93)	0.47 (0.35, 0.62)	0.72 (0.49, 1.04)
Ashanti	1.01 (0.54, 1.86)	0.81 (0.38, 1.73)	0.59 (0.44, 0.79)	0.77 (0.54, 1.10)
Brong Ahafo	0.92 (0.51, 1.67)	0.72 (0.33, 1.59)	0.39 (0.27, 0.55)	0.85 (0.57, 1.27)
Northern	1.24 (0.74, 2.07)	1.04 (0.47, 2.32)	0.11 (0.08, 0.18)	0.41 (0.22, 0.75)
Upper East	1.12 (0.66, 1.89)	0.94 (0.40, 2.20)	0.18 (0.12, 0.26)	0.73 (0.45, 1.19)
Upper West	0.82 (0.46, 1.46)	0.73 (0.31, 1.75)	0.19 (0.13, 0.27)	0.71 (0.41, 1.24)

	UNDERWEIGHT		OVERWEIGHT/OBESITY	
**Level of Education**				
No Education (Reference)	1.00	1.00	1.00	1.00
Primary	1.52 (1.04, 2.23)	1.67 (1.06, 2.64)	1.70 (1.33, 2.17)	1.44 (1.10, 1.88)
Secondary	1.22 (0.90, 1.66)	1.26 (0.81, 1.96)	2.06 (1.67, 2.54)	1.45 (1.11, 1.89)
Higher	1.36 (0.62, 3.02)	1.93 (0.74, 5.06)	3.91 (2.70, 5.66)	1.38 (0.82, 2.31)
**Wealth Quintile**				
Poorest (Reference)	1.00	1.00	1.00	1.00
Poorer	0.84 (0.57, 1.23)	0.89 (0.57, 1.40)	2.42 (1.84, 3.20)	1.65 (1.20, 2.26)
Middle	0.57 (0.38, 0.85)	0.62 (0.34, 1.15)	4.50 (3.40, 5.95)	2.73 (1.90, 3.93)
Richer	0.79 (0.51, 1.23)	0.76 (0.38, 1.51)	7.89 (5.88, 10.6)	4.41 (2.87, 6.75)
Richest	0.74 (0.47, 1.16)	0.55 (0.25, 1.23)	10.7 (8.15, 14.1)	6.47 (4.00, 10.5)
**Occupation**				
Not Working (Reference)	1.00	1.00	1.00	1.00
Nonmanual	0.51 (0.34, 0.78)	0.62 (0.38, 1.02)	3.70 (2.97, 4.60)	1.24 (0.96, 1.60)
Manual	0.42 (0.24, 0.74)	0.55 (0.29, 1.03)	2.50 (1.88, 3.32)	1.14 (0.83, 1.57)
Agricultural	0.74 (0.52, 1.06)	1.07 (0.64, 1.78)	0.79 (0.61, 1.03)	0.57 (0.39, 0.81)
**Media Exposure**				
Not Exposed (Reference)	1.00	1.00	1.00	1.00
Exposed to one media source	0.86 (0.55, 1.33)	0.89 (0.55, 1.46)	2.49 (1.75, 3.53)	1.75 (1.16, 2.65)
Exposed to two media sources	0.73 (0.46, 1.16)	0.79 (0.45, 1.40)	4.42 (3.14, 6.22)	1.88 (1.24, 2.87)
Exposed to three media sources	0.84 (0.50, 1.41)	0.78 (0.39, 1.56)	4.89 (3.26, 7.34)	2.09 (1.25, 3.48)
**Hormonal Contraceptive Use**				
No (Reference)	1.00	1.00	1.00	1.00
Yes	0.36 (0.21, 0.61)	0.44 (0.25, 0.76)	0.96 (0.78, 1.19)	0.85 (0.65, 1.11)

Overall Model Fit: F (100, 308) = 9.39, *P*=0.0000.

Table S67: Unadjusted and adjusted relative risk ratio, of underweight and overweight/obesity among women of child-bearing age in Lesotho.

VARIABLES	UNDERWEIGHT		OVERWEIGHT/OBESITY	
	Unadjusted RRR (95% CI)	Adjusted RRR (95% CI)	Unadjusted RRR (95% CI)	Adjusted RRR (95% CI)
**Age**				
15-19 (Reference)	1.00	1.00	1.00	1.00
20-24	0.85 (0.50, 1.45)	1.29 (0.72, 2.28)	2.12 (1.54, 2.90)	1.61 (1.10, 2.37)
25-29	0.33 (0.16, 0.69)	0.63 (0.28, 1.44)	4.28 (3.02, 6.05)	2.96 (1.90, 4.60)
30-34	0.55 (0.26, 1.16)	1.33 (0.56, 3.14)	4.73 (3.41, 6.57)	3.12 (2.04, 4.77)
35-39	0.18 (0.05, 0.58)	0.42 (0.11, 1.64)	6.48 (4.52, 9.28)	4.32 (2.61, 7.16)
40-44	0.37 (0.14, 0.99)	0.91 (0.34, 2.43)	8.09 (5.91, 11.1)	5.93 (3.72, 9.46)
45-49	0.37 (0.16, 0.89)	0.92 (0.27, 3.12)	8.75 (5.73, 13.4)	6.99 (3.94, 12.4)
**Religion**				
Catholics (Reference)	1.00	1.00	1.00	1.00
Other Christians	0.70 (0.47, 1.04)	0.72 (0.48, 1.10)	0.96 (0.79, 1.16)	0.91 (0.74, 1.13)
Others	0.28 (0.04, 2.17)	0.29 (0.04, 1.91)	0.73 (0.40, 1.35)	0.78 (0.36, 1.68)
**Parity**				
0 (Reference)	1.00	1.00	1.00	1.00
1	0.61 (0.37, 1.00)	0.71 (0.36, 1.38)	2.26 (1.73, 2.95)	1.14 (0.79, 1.65)
2	0.34 (0.16, 0.72)	0.43 (0.15, 1.22)	3.45 (2.60, 4.58)	1.33 (0.84, 2.09)
3	0.16 (0.05, 0.54)	0.20 (0.05, 0.81)	3.55 (2.60, 4.84)	1.23 (0.74, 2.02)
4	0.43 (0.18, 1.01)	0.59 (0.17, 2.11)	4.90 (3.26, 7.36)	1.47 (0.84, 2.56)
5	0.22 (0.05, 0.98)	0.27 (0.04, 1.72)	4.59 (2.82, 7.46)	1.56 (0.84, 2.88)
6+	0.34 (0.10, 1.11)	0.42 (0.09, 1.90)	3.42 (2.29, 5.09)	1.11 (0.60, 2.07)
**Marital Status**				
Single (Reference)	1.00	1.00	1.00	1.00
Married	0.46 (0.29, 0.72)	0.80 (0.41, 1.55)	3.11 (2.51, 3.86)	1.50 (1.08, 2.08)
Formerly Married	0.33 (0.15, 0.73)	0.64 (0.24, 1.73)	2.71 (2.03, 3.60)	1.03 (0.70, 1.51)
**Residential Setting**				
Rural (Reference)	1.00	1.00	1.00	1.00
Urban	1.15 (0.75, 1.78)	1.27 (0.70, 2.31)	1.42 (1.14, 1.77)	0.92 (0.70, 1.20)
**Region**				
Maseru (Reference)	1.00	1.00	1.00	1.00
Botha-Bothe	0.58 (0.25, 1.33)	0.65 (0.27, 1.55)	0.97 (0.69, 1.36)	1.33 (0.91, 1.95)
Leribe	0.75 (0.35, 1.63)	0.83 (0.39, 1.78)	1.03 (0.73, 1.45)	1.14 (0.79, 1.65)
Berea	1.46 (0.72, 2.93)	1.48 (0.72, 3.04)	1.09 (0.80, 1.49)	1.04 (0.74, 1.47)
Mafeteng	1.42 (0.69, 2.92)	1.56 (0.78, 3.10)	1.10 (0.76, 1.60)	1.18 (0.80, 1.73)
Mohale’s Hoek	0.75 (0.35, 1.62)	0.79 (0.35, 1.75)	0.95 (0.67, 1.34)	1.29 (0.92, 1.79)
Quthing	1.15 (0.58, 2.28)	1.24 (0.61, 2.50)	0.90 (0.60, 1.35)	1.19 (0.82, 1.74)
Qacha’s-Nek	1.11 (0.56, 2.20)	1.06 (0.52, 2.19)	0.91 (0.62, 1.33)	1.28 (0.86, 1.92)
Mokhotlong	0.81 (0.36, 1.83)	0.75 (0.31, 1.81)	0.44 (0.31, 0.62)	0.74 (0.51, 1.08)
Thaba Tseka	1.12 (0.57, 2.20)	1.25 (0.61, 2.56)	0.54 (0.36, 0.81)	0.79 (0.53, 1.20)
**Level of Education**				
No Education (Reference)	1.00	1.00	1.00	1.00
Primary	1.87 (0.36, 9.76)	1.00 (0.20, 4.99)	1.27 (0.52, 3.08)	1.35 (0.55, 3.31)
Secondary	1.85 (0.35, 9.70)	0.67 (0.12, 3.63)	1.33 (0.53, 3.33)	1.76 (0.70, 4.43)
Higher	1.68 (0.26, 10.8)	0.63 (0.09, 4.19)	2.31 (0.90, 5.92)	1.78 (0.69, 4.61)
**Wealth Quintile**				
Poorest (Reference)	1.00	1.00	1.00	1.00
Poorer	0.99 (0.52, 1.87)	0.94 (0.45, 1.96)	1.88 (1.38, 2.55)	1.80 (1.31, 2.47)
Middle	0.82 (0.45, 1.51)	0.73 (0.35, 1.50)	2.32 (1.75, 3.06)	2.16 (1.55, 3.00)
Richer	0.76 (0.41, 1.40)	0.58 (0.26, 1.29)	2.87 (2.14, 3.86)	2.76 (1.92, 3.97)
Richest	1.09 (0.58, 2.03)	0.65 (0.23, 1.86)	3.75 (2.84, 4.96)	3.65 (2.42, 5.51)

	UNDERWEIGHT		OVERWEIGHT/OBESITY	
**Media Exposure**				
Not Exposed (Reference)	1.00	1.00	1.00	1.00
Exposed to one media source	0.75 (0.46, 1.22)	0.82 (0.48, 1.41)	1.54 (1.20, 1.97)	1.02 (0.76, 1.36)
Exposed to two media sources	1.07 (0.64, 1.78)	1.16 (0.63, 2.13)	2.01 (1.54, 2.61)	1.10 (0.79, 1.52)
Exposed to three media sources	1.10 (0.61, 1.98)	1.07 (0.49, 2.35)	1.99 (1.51, 2.61)	1.13 (0.77, 1.64)
**Hormonal Contraceptive Use**				
No (Reference)	1.00	1.00	1.00	1.00
Yes	0.53 (0.31, 0.91)	0.85 (0.45, 1.58)	1.59 (1.32, 1.92)	1.21 (0.97, 1.50)

Overall Model Fit: F (74, 306) = 6.81, *P*=0.0000.

Table S68: Unadjusted and adjusted relative risk ratio, of underweight and overweight/obesity among women of child-bearing age in Eswatini.

VARIABLES	UNDERWEIGHT		OVERWEIGHT/OBESITY	
	Unadjusted RRR (95% CI)	Adjusted RRR (95% CI)	Unadjusted RRR (95% CI)	Adjusted RRR (95% CI)
**Age**				
15-19 (Reference)	1.00	1.00	1.00	1.00
20-24	0.54 (0.32, 0.92)	0.82 (0.44, 1.53)	1.99 (1.62, 2.46)	1.46 (1.13, 1.90)
25-29	0.36 (0.16, 0.79)	0.80 (0.27, 2.33)	3.38 (2.74, 4.18)	2.06 (1.54, 2.75)
30-34	0.29 (0.12, 0.71)	0.77 (0.25, 2.38)	5.68 (4.53, 7.11)	3.10 (2.18, 4.40)
35-39	0.70 (0.30, 1.66)	2.05 (0.68, 6.23)	7.99 (6.28, 10.2)	4.39 (3.08, 6.26)
40-44	0.48 (0.18, 1.28)	1.54 (0.40, 6.00)	10.6 (7.88, 14.3)	5.81 (3.68, 9.17)
45-49	0.50 (0.20, 1.28)	1.42 (0.47, 4.32)	8.20 (6.25, 10.7)	4.89 (3.29, 7.26)
**Religion**				
Catholics (Reference)	1.00	1.00	1.00	1.00
Other Christians	0.59 (0.28, 1.23)	0.59 (0.28, 1.26)	1.10 (0.79, 1.53)	1.38 (0.99, 1.92)
Traditionalists	0.80 (0.09, 7.47)	0.74 (0.07, 8.35)	0.63 (0.28, 1.41)	0.73 (0.34, 1.58)
No Religion	0.66 (0.20, 2.17)	0.67 (0.20, 2.30)	1.01 (0.62, 1.63)	1.07 (0.67, 1.70)
**Parity**				
0 (Reference)	1.00	1.00	1.00	1.00
1	0.67 (0.40, 1.10)	0.98 (0.55, 1.74)	2.22 (1.83, 2.70)	1.53 (1.20, 1.94)
2	0.35 (0.17, 0.71)	0.59 (0.26, 1.35)	3.07 (2.49, 3.79)	1.62 (1.24, 2.12)
3	0.67 (0.33, 1.34)	1.12 (0.44, 2.82)	4.28 (3.32, 5.51)	1.79 (1.27, 2.53)
4	0.33 (0.09, 1.29)	0.48 (0.12, 1.96)	5.64 (4.28, 7.43)	2.19 (1.49, 3.21)
5	0.17 (0.02, 1.22)	0.21 (0.03, 1.76)	6.72 (4.84, 9.34)	2.35 (1.56, 3.55)
6+	0.37 (0.15, 0.92)	0.37 (0.10, 1.38)	6.66 (5.41, 8.19)	2.40 (1.66, 3.48)
**Marital Status**				
Single (Reference)	1.00	1.00	1.00	1.00
Married	0.30 (0.17, 0.54)	0.41 (0.21, 0.78)	2.85 (2.47, 3.28)	1.08 (0.88, 1.31)
Formerly Married	0.92 (0.45, 1.91)	1.14 (0.48, 2.71)	3.20 (2.60, 3.95)	0.99 (0.77, 1.28)
**Residential Setting**				
Rural (Reference)	1.00	1.00	1.00	1.00
Urban	1.10 (0.74, 1.64)	1.49 (0.88, 2.54)	1.30 (1.11, 1.52)	0.85 (0.70, 1.05)
**Region**				
Hhohho (Reference)	1.00	1.00	1.00	1.00
Manzini	1.82 (1.00, 3.31)	1.59 (0.86, 2.95)	1.02 (0.85, 1.21)	1.10 (0.90, 1.34)
Shiselweni	1.76 (0.96, 3.22)	1.52 (0.80, 2.88)	0.93 (0.78, 1.11)	1.19 (0.99, 1.44)
Lubombo	1.75 (0.94, 3.28)	1.59 (0.87, 2.93)	0.88 (0.72, 1.07)	1.01 (0.81, 1.27)
**Level of Education**				
No Education (Reference)	1.00	1.00	1.00	1.00
Primary	1.15 (0.56, 2.36)	0.76 (0.36, 1.62)	0.81 (0.62, 1.05)	1.18 (0.86, 1.62)
Secondary	0.94 (0.47, 1.87)	0.57 (0.26, 1.27)	0.88 (0.70, 1.11)	1.36 (1.00, 1.86)
Higher	0.44 (0.14, 1.44)	0.38 (0.11, 1.40)	1.64 (1.19, 2.26)	1.11 (0.72, 1.72)
**Wealth Quintile**				
Poorest (Reference)	1.00	1.00	1.00	1.00
Poorer	0.94 (0.57, 1.55)	0.88 (0.53, 1.48)	1.25 (1.00, 1.57)	1.28 (0.97, 1.68)
Middle	0.70 (0.40, 1.24)	0.67 (0.36, 1.26)	1.40 (1.10, 1.79)	1.52 (1.11, 2.07)
Richer	0.78 (0.48, 1.27)	0.66 (0.35, 1.24)	1.74 (1.38, 2.19)	2.02 (1.47, 2.77)
Richest	0.73 (0.44, 1.22)	0.63 (0.28, 1.45)	2.23 (1.76, 2.82)	2.55 (1.79, 3.63)
**Occupation**				
Not Working (Reference)	1.00	1.00	1.00	1.00
Nonmanual	0.40 (0.24, 0.67)	0.49 (0.29, 0.83)	2.46 (2.15, 2.82)	1.23 (1.03, 1.47)
Manual	0.84 (0.34, 2.06)	0.90 (0.33, 2.43)	2.33 (1.75, 3.11)	1.21 (0.91, 1.60)
Agricultural	0.11 (0.02, 0.48)	0.10 (0.02, 0.48)	1.78 (1.30, 2.43)	1.00 (0.73, 1.36)

	UNDERWEIGHT		OVERWEIGHT/OBESITY	
**Media Exposure**				
Not Exposed (Reference)	1.00	1.00	1.00	1.00
Exposed to one media source	1.30 (0.71, 2.39)	1.29 (0.67, 2.46)	0.99 (0.77, 1.27)	1.07 (0.80, 1.43)
Exposed to two media sources	0.97 (0.51, 1.83)	0.93 (0.45, 1.92)	1.07 (0.85, 1.34)	1.16 (0.85, 1.57)
Exposed to three media sources	1.11 (0.60, 2.03)	1.21 (0.54, 2.67)	1.35 (1.06, 1.71)	1.30 (0.91, 1.86)
**Hormonal Contraceptive Use**				
No (Reference)	1.00	1.00	1.00	1.00
Yes	0.43 (0.22, 0.86)	0.72 (0.35, 1.47)	1.46 (1.22, 1.74)	1.14 (0.94, 1.38)

Overall Model Fit: F (70, 70) = 13.75, *P*=0.0000.
